# Dynamic Kinetic Resolution of Indole-Based Sulfenylated Heterobiaryls by Rhodium-Catalyzed Atroposelective Reductive Aldol Reaction

**DOI:** 10.1021/acscatal.3c03422

**Published:** 2023-08-30

**Authors:** Carlos Rodríguez-Franco, Abel Ros, Pedro Merino, Rosario Fernández, José M. Lassaletta, Valentín Hornillos

**Affiliations:** † Instituto de Investigaciones Químicas (CSIC-US) and Centro de Innovación en Química Avanzada (ORFEO−CINQA), Avda. Américo Vespucio, 49, 41092 Sevilla, Spain; ‡ Instituto de Biocomputación y Física de Sistemas Complejos (BIFI), Universidad de Zaragoza, 50009 Zaragoza, Spain; § Departamento de Química Orgánica, Universidad de Sevilla and Centro de Innovación en Química Avanzada (ORFEO−CINQA), C/Prof. García González, 1, 41012 Sevilla, Spain

**Keywords:** asymmetric catalysis, axial chirality, 3-aryl indoles, dynamic kinetic resolution, rhodium

## Abstract

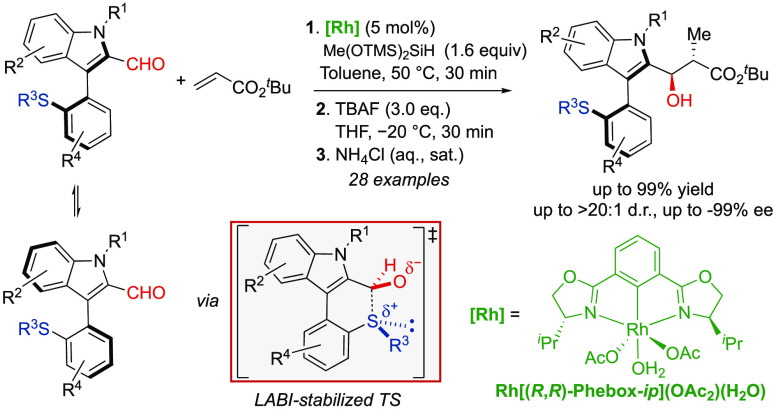

A highly enantio- and diastereoselective dynamic kinetic resolution (DKR) of configurationally labile 3-aryl indole-2-carbaldehydes is described. The DKR proceeds via a Rh-catalyzed intermolecular asymmetric reductive aldol reaction with acrylate esters, with simultaneous generation of three stereogenic elements. The strategy relies on the labilization of the stereogenic axis that takes place thanks to a transient Lewis acid–base interaction (LABI) between the formyl group and a thioether moiety strategically located at the *ortho′* position. The atropisomeric indole products present a high degree of functionalization and can be further converted to a series of axially chiral derivatives, thereby expanding their potential application in drug discovery and asymmetric catalysis.

Enantioenriched axially chiral compounds constitute important scaffolds present in natural products,^1^ drug discovery,^2^ and material science.^3^ Furthermore, axially chiral skeletons widely exist in privileged chiral ligands^4^ and organocatalysts^5^ that play crucial roles in the preparation of chiral compounds. Despite the large development of catalytic asymmetric methodologies for the synthesis of these motives, most prepared axially chiral scaffolds consist of biaryls based on six-membered rings.^6^ In contrast, the construction of biaryl compounds based on five-membered rings has been less explored and is more challenging, as the large spatial distance between the *ortho* substituents of both aryl rings requires higher degrees of steric constraints to stabilize the stereogenic axis.^7^ An outstanding member of this family of atropisomers comprises C3 chiral indole derivatives and analogues.^8^ Their core structure is present in a number of natural products,^9^ bioactive compounds^10^ and chiral ligands,^11^ and organocatalysts^12^ (Figure 1). Furthermore, the indole ring has a unique reactivity that can serve to change the electron density, modulate the steric congestion, and allow participation as a hydrogen bond donor.^13^ Because of the interest in this class of compounds, a variety of catalytic enantioselective methods have recently been reported for their synthesis, including atroposelective arylation reactions,^14^ de novo construction of the indole ring,^15^ atroposelective desymmetrization based on C–H functionalizations,^10d,16^ and central-to-axial chirality transfer processes.^17^ Despite these developments, limitations exist regarding structural variability and the strategic introduction of functional groups. Additionally, the catalytic asymmetric synthesis of atropisomers bearing multiple stereogenic elements is underdeveloped. Therefore, novel synthetic strategies for the preparation of axially chiral aryl indole scaffolds are still needed.

**Figure 1:**
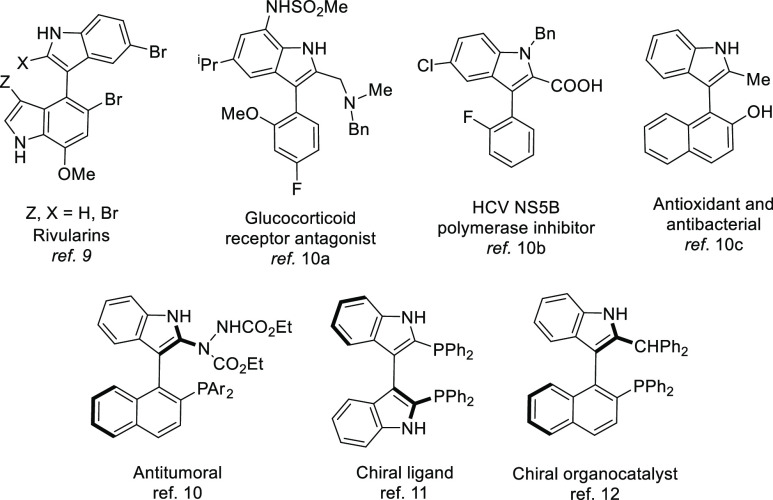
Selected 3-aryl indole frameworks.

In the frame of our interest in atroposelective catalysis, we recently developed a dynamic kinetic resolution (DKR) strategy for the synthesis of axially chiral biaryls on the basis of the labilization of the stereogenic axis facilitated by transient Lewis acid–base interaction (LABI) between functional groups strategically located in the molecule. Such interaction overrides the steric repulsion in the transition structure and acts as a “lubricant” that substantially reduces the rotational barrier in the substrate. The axis could then be stabilized by any asymmetric transformation in which the interaction is disabled, thereby enabling a DKR scenario. This strategy was first demonstrated in the atroposelective zinc-catalyzed hydrosilylation of heterobiaryl ketones to obtain enantioenriched carbinols with central and axial chirality (Scheme 1A).^18^ A related DKR strategy was also reported by Clayden and Turner for the biocatalytic asymmetric reduction of heterobiaryl aldehydes, which showed a similar bonding interaction between a *N*-oxide and an aldehyde (Scheme 1B).^19^ More recently, LABI N···CHO interactions have been exploited for the biocatalytic dynamic resolution of *N*-aryl indoles^20^ and the allylation of 8-aryl quinoline derivatives^21^ (Scheme 1C).

**Scheme 1:**
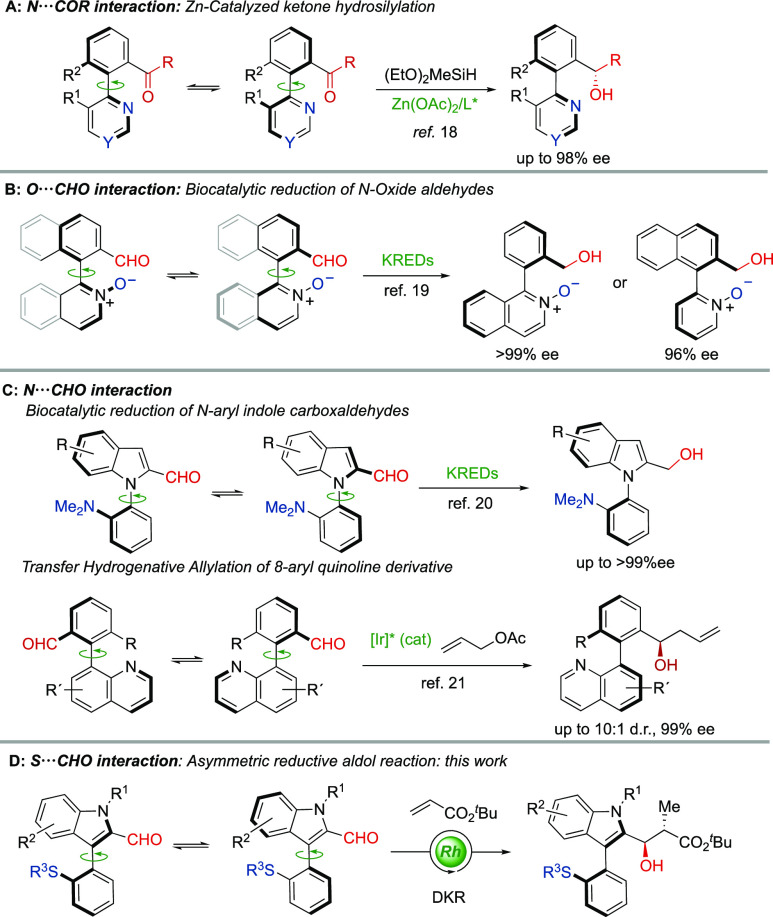
Atropisomerization via Lewis Acid/Base Interactions

However, axially chiral biaryls bearing thioether moieties are rare in the literature compared with phosphine or amine analogues^22^ but hold appealing structures for their application in asymmetric catalysis^23^ and feature interesting bioactivities.^24^ With this motivation, we wondered whether a thioether as the Lewis base and a carbonyl group as a weak Lewis acid would be a competent Lewis pair able to promote the labilization of a stereogenic axis on the basis of the discussed strategy. According to a recent report,^22i^ however, trisubstituted biphenyl derivatives bearing CHO and SMe groups in *ortho*,*ortho′* positions exhibit configurational stability, which suggests that the steric repulsion dominates the LABI SMe···CHO interaction in this system. However, we speculated whether the repulsive/attractive force balance could be reverted in a sterically more relaxed heterobiaryl scaffold featuring a five-membered ring.^7a^ Considering that the introduction of a thioether moiety in functionalized axially chiral 3-aryl indoles could open new avenues for not only chiral ligands^23^ but also drug candidates,^24^ we decided to explore the suitability of the LABI-based DKR strategy to the resolution of trisubstituted 3-aryl indole scaffolds **1** bearing an aldehyde and a thioether groups at *ortho*, *ortho′* positions (Scheme 1D).

The HPLC analysis of *N*-benzyl-3-[2-(methylthio)phenyl]-1*H*-indole-2-carbaldehyde **1a**, chosen as a model substrate, showed a unique narrow peak on a variety of chiral stationary phases, thereby pointing to its configurational lability in solution. Additionally, DFT computational studies at the ωB97XD/def2tzvp/smd=toluene level of theory were conducted to estimate the barrier of racemization in the 3-aryl indole system and, for the sake of comparison, in the aforementioned biphenyl derivatives. Transition structures **TSA**_***cis***_ and **TSB**_***cis***_ were located for the 3-aryl indole system **A** and the biaryl analogue **B**, respectively (Figure 2). A barrier of 24.1 kcal mol^–1^ was calculated for the racemization of **A**, which demonstrates that this system is stable enough to detect atropisomers, but at ambient temperature and above, the racemization rate enables a dynamic kinetic resolution.^25^ In sharp contrast, the barrier of 37.9 kcal mol^–1^ calculated for **B** grants its configurational stability with a half-life (*t*_1/2_) of 2 × 10^7^ years at 25 °C. At first sight, these values are surprisingly high in comparison with those calculated for similar transition structures featuring O (*N*-oxide, 18.4 kcal mol^–1^)^19^ or N (NMe_2_, 24.2 kcal mol^–1^)^20^ basic functionalities. Reasonably, however, the barrier correlates well with the interaction distance (1.96 Å for O···CHO,^19^ 2.15 Å for N···CHO,^20^ and 2.64 Å for S···CHO), which causes severe distortions in **TSB**_***cis***_. Among the two possible transition structures for the racemization, those facing the carbonyl group and the sulfur atom are the most stable in both cases (by 10.4 and 6.6 kcal mol^–1^ for **A** and **B**, respectively) because of the designed noncovalent interaction between the sulfur atom and the carbonyl carbon. The key role of the LABI interaction was further demonstrated by replacing the formyl group in **A** or **B** by a methyl group, which resulted in much higher barriers of racemization (for details, see the Supporting Information). Two relative configurations are possible for these transition structures, depending on the orientation of the methyl group with respect to the carbonyl group. Interestingly, those structures with a *cis* orientation (**TSA**_***cis***_ and **TSB**_***cis***_) feature a CH···O hydrogen bond that provides a significant extra stabilization with respect to the *trans* isomers **TSA**_***trans***_ and **TSB**_***trans***_ (1.3 and 1.0 kcal mol^–1^ for **A** and **B**, respectively). This interaction can be rationalized by considering the enhanced basicity of the oxygen and the enhanced acidity of the methyl group in the transition state as a consequence of the developing negative and positive charges in the oxygen and sulfur atoms, respectively. All these interactions were confirmed by a noncovalent interactions (NCI) analysis.

**Figure 2:**
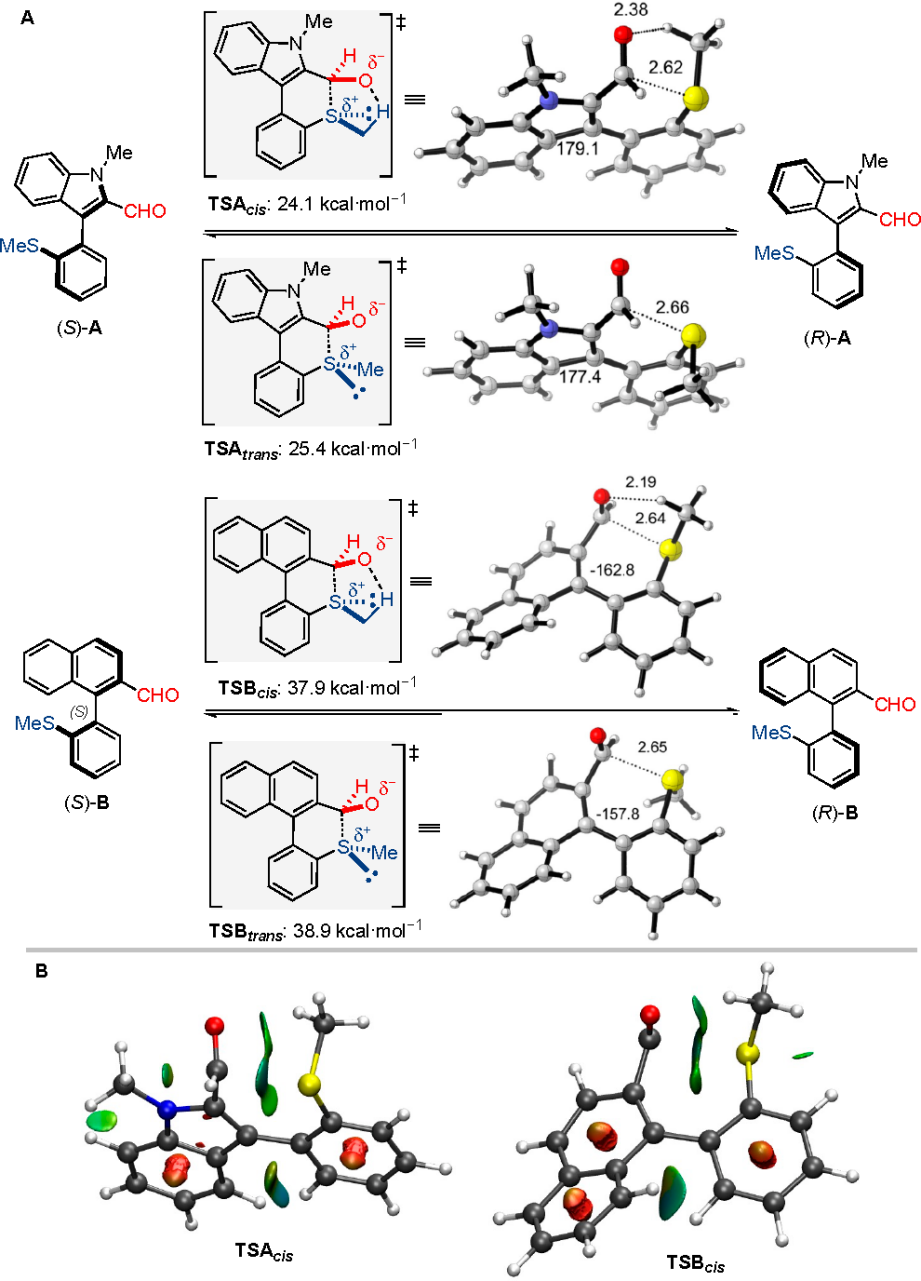
(A) Transition structures for the racemization of 3-aryl indole **A** and biaryl **B**. (B) NCI analysis. Thin, delocalized green surfaces indicate van der Waals interactions. Small, lenticular, and bluish surfaces indicate strong interactions. Steric clashes are shown as red isosurfaces.

On this basis, we envisioned quaternization of the carbonyl group of indole derivatives **1** (e.g., by nucleophilic addition) to destroy its Lewis acidic character, thereby increasing the rotational barrier in the resulting product to make it configurationally stable. Among the countless possible catalytic enantioselective transformations, we selected the rhodium-catalyzed reductive aldol reaction as an appealing option that provides densely functionalized products.^26^ The designed atroposelective version of this transformation represents a major challenge for two main reasons: First, an exquisite level of stereocontrol is required because the reaction could potentially afford up to eight different stereoisomers derived from the simultaneous installation of two stereocenters and a stereogenic axis. Second, conditions have to be found to avoid catalyst deactivation or undesirable side reactions caused by coordination of the thioether functional group in the substrate. Inspired by the Nishiyama’s enantioselective reductive coupling of α,β-unsaturated esters catalyzed by chiral rhodium(bisoxazolinylphenyl) complexes [Rh(Phebox)],^26a^ we first examined the reaction of **1a** with *tert*-butyl acrylate (1.5 equiv) in toluene using 6 mol % of catalyst. The desired coupling product **2a** was efficiently formed in 98% conversion and 90% ee with a 6:1 diastereoselectivity (Table 1, entry 1). A significant influence by the solvent on reactivity and stereoselectivity was found (entries 2–5 and Supporting Information), with toluene still giving the best results. No benefits were observed at lower temperatures: dr and ee values were essentially maintained at 40 or 30 °C (entries 6 and 7), but longer reaction times were required and a moderate conversion was observed in the latter case. A systematic study of hydrosilanes (entries 8–14 and Supporting Information) revealed that sterically hindered Me(OTMS)_2_SiH is the optimal reagent, which affords excellent 99% ee with full conversion and 9:1 diastereoselectivity (entry 14). Et_3_SiH was also found to perform well and afforded a similar diastereoselectivity, although at the expense of conversion and enantioselectivity (entry 13). The reaction proceeds with an excellent *anti* selectivity and, remarkably, the absolute (2*S*, 3*R*) configuration of the newly created stereocenters suggests that the sulfenyl group of the substrate causes no interferences with the catalytic cycle and the stereochemical model.^26f^ This statement applies also to the minor diastereomers and epimers in the stereogenic axis.

**Table 1:**
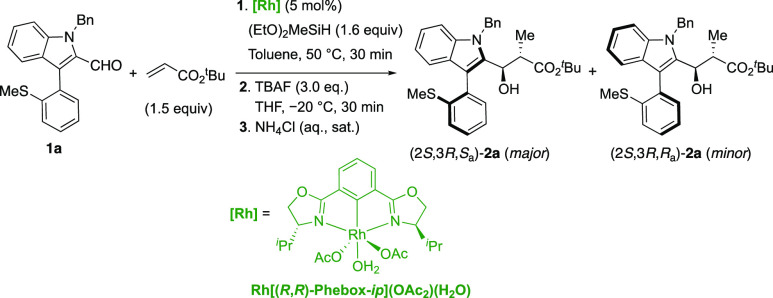
Variation of Reaction Parameters^a^

entry	variation of standard conditions	conversion (%)^b^	dr^b^	ee (%)^c^
1	none	98	6:1	90
2	in 1,4-dioxane	89	7:2	89
3	in THF	91	7:2	89
4	in DMSO	79	3:1	67
5	in MeCN	58	5:2	83
6	*T* = 40 °C^d^	97	6:1	90
7	*T* = 30 °C^e^	45	7:1	91
8	with Me_3_SiH	<5	n.d.	n.d.
9	with (EtO)_3_SiH	40	5:1	70
10	with Me_2_PhSiH	72	6:1	10
11	with (MeO)_3_SiH	60	5:1	75
12	with PMHS	78	7:3	85
13	with Et_3_SiH	75	9:1	95
14	with Me(OTMS)_2_SiH	98	9:1	99

a Conditions: 0.1 mmol of **1a**.

b Determined by ^1^H NMR.

c Determined by HPLC analysis on chiral stationary phases.

d Reaction time of 1.5 h.

e Reaction time of 4.5 h.

With the optimal conditions in hand, we next investigated the substrate scope of the methodology (Scheme 2). A wide range of 2-formyl aryl indoles smoothly reacted with *tert*-butyl acrylate to deliver the corresponding axially chiral products (**2a**–**2z**) in good to excellent yields and diastereomeric ratios (up to 99% yield, >20:1 dr) and with excellent enantioselectivities (up to 99% ee). We first investigated the effect of aryl substituents at the benzyl group. Both electron-withdrawing (e.g., Br, CF_3_, and OCF_3_; **1b**–**1d, 1o**, **1p**), and electron-donating groups (**1q**, **1r**) were tolerated and afforded the corresponding products in good yields with high selectivity. Other nitrogen substituents, including methyl and allyl groups, were also amenable to give the desired products in good yields and similar enantiocontrol (**2e**, **2f**, **2s**). Different aryl substitutions on the indole ring were also examined. A series of functional groups, such as halogen (**1j**–**1l** and **1v**–**1y**), benzyl (**2i** and **2u**), and methyl ethers (**1g**, **1h**, and **1t**), could be well tolerated under the optimal reaction conditions. The excellent enantiocontrol was unperturbed by C-5 or C-6 substitution at the indole ring. It is worth noting that near perfect diastereoselectivities (>20:1 dr) were regularly observed in the reaction of substrates containing ethylsufinyl and benzylsulfinyl substituents (**2m**–**2z**) while maintaining high yields and excellent enantioselectivities.^27^ Aldehyde **1a** was also made to react with *n*-butyl acrylate under the optimized conditions to afford the expected aldol product **2aa** in good yield but with decreased diastereo- and enantioselectivity. This result highlights the importance of the bulkier *tert-*butyl ester to afford a highly selective DKR process. Conversely, products **2ab** and **2ac** featuring benzothiophenyl and dibenzothiophenyl rings at the bottom fragment of the heterobiaryl skeleton were obtained in excellent yields and enantioselectivity but were configurationally labile. The wider SC_*ortho*_C_*ipso*_ angles associated with the benzothiophene ring facilitate the rotation about the stereogenic axis in these compounds. Thus, the observed 1.7:1 and 1.2:1 diastereomeric ratios for **2ab** and **2ac**, respectively, correspond to the relative stability (conformational equilibrium) of the resulting atropisomers.

**Scheme 2:**
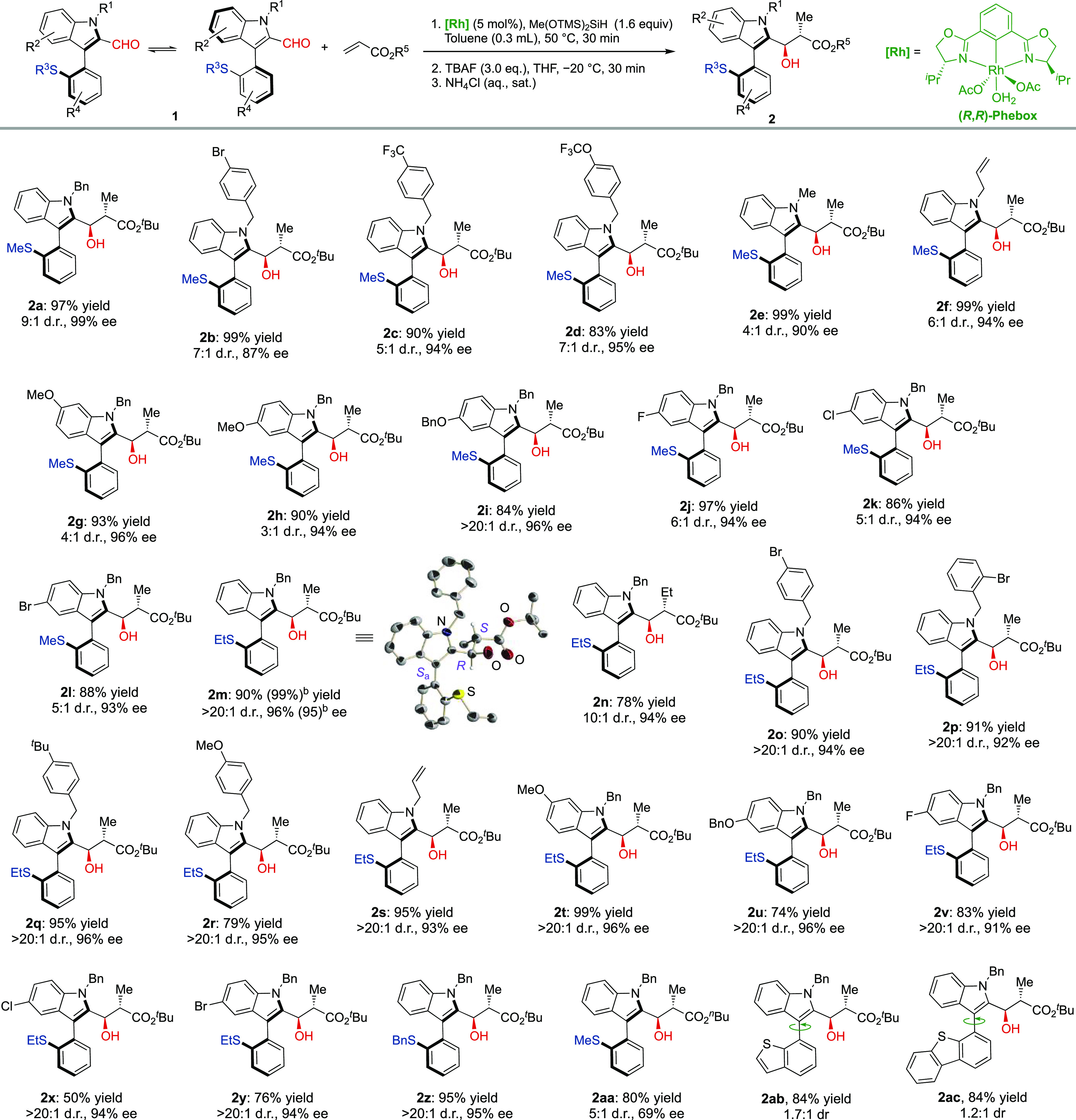
Reaction Scope Reactions performed at 0.1 mmol scale in 0.3 mL of toluene. Compounds **1a**–**w** (1 equiv), *tert*-butyl acrylate (1.5 equiv). Isolated yields after chromatography. HPLC on chiral stationary phases determined the ee values. Reaction performed at 1.2 mmol scale.

The absolute (2*S*,3*R*,*S*_a_) configuration of product **2m** was determined by X-ray crystallographic analysis.^28^ The absolute (2*S*,3*R*,*R*_a_) configuration of the minor diastereomer was determined after thermal equilibration: heating of the enantiopure major (2*S*,3*R*,*S*_a_)-**2m** epimer at 80 °C for 12 h in toluene afforded a 2:1 mixture of (*S*_a_/*R*_a_) atropoisomers.^29^ The absolute configuration of other products **2** was assigned by analogy and assuming a uniform reaction pathway.

Importantly, the catalyst loading could be reduced to 2 mol % when the reaction of **1m** was performed at 1.2 mol scale, which improved the isolated yield of the product while maintaining the stereochemical efficiency (>20:1 dr and 96% ee).

Further derivatizations were performed using ethyl thioether derivative **2m** as a representative example (Scheme 3). The carboxylate group could be reduced using LiAlH_4_ to afford diol **3** in 99% yield with 95% ee. This product was further transformed into acetal derivative **4** in 84% yield. The thioether group could also be oxidized to the corresponding sulfoxide derivative **6**(28) with excellent diastereoselectivity (>20:1 dr) using 1.0 equiv of *m*-CPBA at −78 °C. The oxidation to the sulfone **5** was also achieved in 97% yield by increasing the amount of *m*-CPBA (2.5 equiv) and performing the reaction at room temperature. Importantly, both oxidation reactions proceeded without noticeable racemization.

**Scheme 3:**
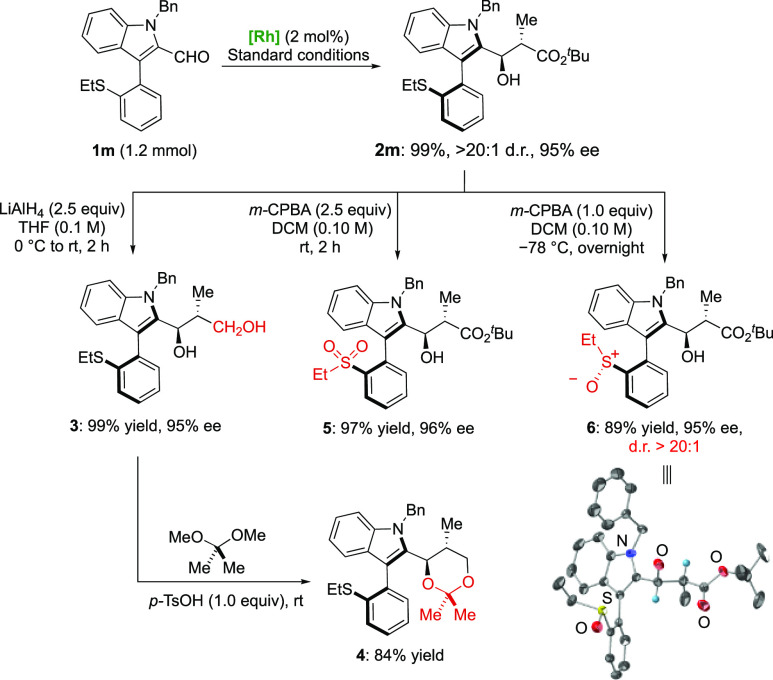
Representative Transformations from **2m**

In summary, the labilization of trisubstituted *ortho′*-sulfenyl 3-aryl indole 2-carbaldehydes by means of an attractive SR···CHO LABI interaction can be exploited to perform a Rh-catalyzed dynamic kinetic resolution via reductive aldol reaction with *t*-butyl acrylate. The reaction proceeds with the generation of two stereocenters and a stereogenic axis to afford densely functionalized, axially chiral indole derivatives in good to excellent yields and high diastereo- and enantioselectivities.

## Supporting Information Available

Experimental procedures, characterization data for new compounds, crystallographic data for (2*R*,3*S*,*S*_a_)-**2m**, DFT calculations, NMR spectra, and HPLC traces (PDF)
Crystallographic data for **2m** (CIF)
Crystallographic data for **6** (CIF)

## Supplementary Material

cs3c03422_si_001.pdf

cs3c03422_si_002.cif

cs3c03422_si_003.cif
