# Supplementary material for: Dynamic Kinetic Resolution of Indole-Based Sulfenylated Heterobiaryls by Rhodium-Catalyzed Atroposelective Reductive Aldol Reaction
Source: ACS Catal. 2023 Aug 30;13(18):12134–41. doi: 10.1021/acscatal.3c03422 (PMC10513111; doi:10.1021/acscatal.3c03422)

Supporting information

Dynamic Kinetic Resolution of Indole-Based Sulfenylated Heterobiaryls by Rhodium-Catalyzed Atroposelective Reductive Aldol Reaction.

Carlos Rodríguez-Franco,[§] Abel Ros,[§] Pedro Merino,[*,‡] Rosario Fernández,[*,∫] José M. Lassaletta[*,§] and Valentín Hornillos[*,∫,§]

[§] Instituto de Investigaciones Químicas (CSIC-US) and Centro de Innovación en Química Avanzada (ORFEO-CINQA), Avda. Américo Vespucio, 49, 41092 Sevilla, Spain.

[‡] Instituto de Biocomputación y Física de Sistemas Complejos (BIFI). Universidad de Zaragoza, 50009 Zaragoza, Spain

[∫] Departamento de Química Orgánica, Universidad de Sevilla and Centro de Innovación en Química Avanzada (ORFEO-CINQA), C/ Prof. García González, 1, 41012 Sevilla, Spain.

*pmerino@unizar.es *ffernan@us.es *jmlassa@iiq.csic.es *vhornillos@iiq.csic.es

Index

1. General information........S2
2. Complete optimization studies........S2
3. Synthesis of starting materials and precursors........S4
4. General procedure for the catalytic asymmetric reductive aldol reaction........S18
5. Representative transformations........S30
6. Computational Studies........S32
7. References........S61
8. NMR Spectra and HPLC traces........S63

1. General information

^{1}H-NMR spectra were recorded at 400 MHz; ^{13}C-NMR spectra were recorded at 100 MHz; with the solvent peak used as the internal reference (7.26 and 77.0 ppm for ^{1}H and ^{13}C respectively for $CDCl_3$); column chromatography was performed on silica gel (Merck Kieselgel 60). Analytical TLC was performed on aluminium backed plates (1.5 × 5 cm) pre-coated (0.25 mm) with silica gel (Merck, Silica Gel 60 F_{254}). Compounds were visualized by exposure to UV light or by dipping the plates in a solution of 5% $(NH_4)_2Mo_7O_{24}\cdot4\ H_2O$ in 95% EtOH (w/v) and heating.

Anhydrous 1,4-dioxane and THF were obtained by distillation from sodium using benzophenone as indicator. Dichloromethane, toluene and MeCN were dried by passage through solvent-purification columns containing activated alumina. Anhydrous DMF, $LiAlH_4$, MnO_2, I_2, KOH, K_2CO_3, benzyl bromide, 4-bromobenzyl bromide, 2-bromobenzyl bromide 4-*tert*-butylbenzyl bromide, 4-trifluoromethylbenzyl bromide, 4-trifluoromethoxybenzyl bromide, MeI, allyl chloride, [Rh((*R*,*R*)-Phebox)$(OAc)_2(H_2O)$], $RhCl(PPh_3)_3$, $ZnCl_2$, *tert*-butyl acrylate, *n*-butyl acrylate, *c*, diethoxymethylsilane, trimethylsilane, triethylsilane, chlorodimethylsilane, 1,1,1,3,5,5,5-Heptamethyltrisiloxane, trimethylsilane, triethoxysilane, dimethylphenylsilane, PMHS, trimethoxysilane, triisopropylsilane, 2,2-dimethoxypropane, *p*-toluenesulfonic acid monohydrate and *m*CPBA were purchased from Sigma-Aldrich. Indole-2-carboxylic acid, 5-methoxyindole-2-carboxylic acid, 6-methoxyindole-2-carboxylic acid, 5-fluoroindole-2-carboxylic acid, 5-chloroindole-2-carboxylic acid, 5-bromoindole-2-carboxylic acid, $Pd(PPh_3)_4$, 4-methoxybenzyl bromide and methyl diphenylsilane were purchased from Fluorochem. 5-Benzyloxyindole-2-carboxylic acid was purchased from TCI.

2. Complete optimization studies

Table S1. *Solvent screening for the atroposelective reductive aldol reaction via DKR of* ***1a*** *with tert-butyl acrylate.*

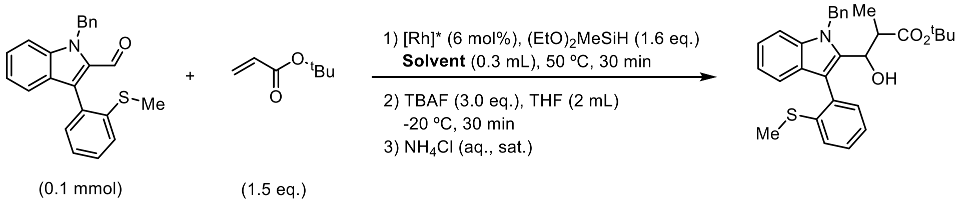

Entry	Solvent	Conv.[a]	*ee*[b]	d.r.[a]
**1**	Toluene	98%	90%	6:1
**2**	1,2-DCE	62% (3 h)	60%	5:2
**3**	1,4-Dioxane	89%	89%	7:2
**4**	THF	91%	89%	7:2
**5**	DMSO	79%	67%	3:1
**6**	MeCN	58%	83%	5:2
**7**	DMF	89%	89%	7:2
**8**	Toluene	98%	90%	6:1
**9**	Trifluorotoluene	62%	89%	6:1
**10**	Xylenes	98%	89%	6:1

[a] Determined by ^{1}H NMR spectroscopy of the crude reaction mixture. [b] Determined by HPLC analysis on chiral stationary phases.

Table S2. *Temperature effect for the atroposelective reductive aldol reaction of* ***1a*** *in toluene.*

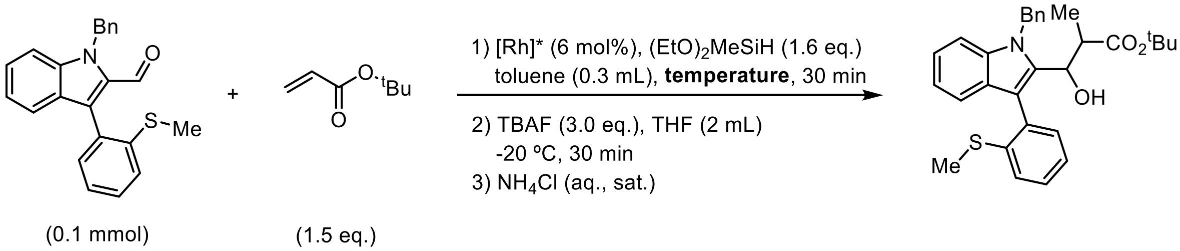

Entry	Temperature	Time	Conv.[a]	*ee*[b]	d.r.[a]
**1**	50 ºC	30 min	98%	90%	6:1
**2**	40ºC	1.5 h	97%	90%	6:1
**3**	30ºC	4.5 h	45%	91%	7:1

[a] Determined by ^{1}H NMR spectroscopy of the crude reaction mixture. [b] Determined by HPLC analysis on chiral stationary phases.

Table S3. *Silane screening for the atroposelective reductive aldol reaction of* ***1a.***

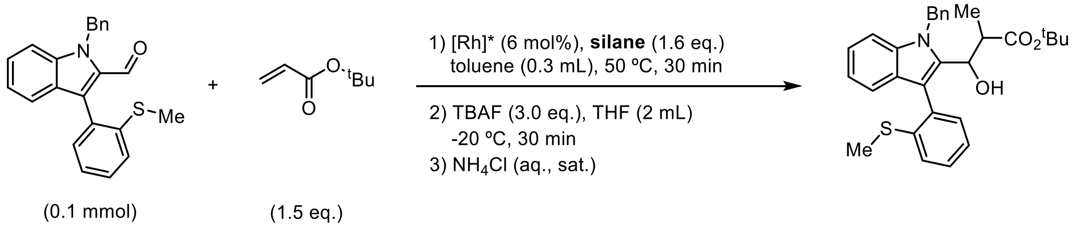

Entry	Silane	Conv.[a]	*ee*[b]	d.r.[a]
**1**	$(EtO)_2MeSiH$	98%	90%	6:1
**2**	Et_3SiH	75%	95%	9:1
**3**	$Cl(Me)_2SiH$	n.r.	-	-
**4**	$Me(OTMS)_2SiH$	98%	99%	9:1
**5**	Me_3SiH	n.r.	-	-
**6**	$(EtO)_3SiH$	40%	70%	5:1
**7**	Me_2PhSiH	72%	10%	6:1
**8**	$MePh_2SiH$	70%	90%	5:1
**9**	PMHS	78%	85%	7:3
**10**	$(MeO)_3SiH$	60%	75%	5:1
**11**	$^iPr_3SiH$	n.r.	-	-

[a] Determined by ^{1}H NMR spectroscopy of the crude reaction mixture. [b] Determined by HPLC analysis on chiral stationary phases.

3. Synthesis of starting materials and precursors

Synthesis of 3-iodo-1*H*-indole-2-carbaldehyde derivatives (7a-l)

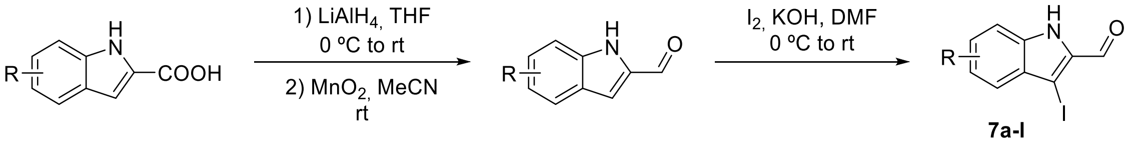

7a-l

3-iodo-1*H*-indole-2-carbaldehyde derivatives were prepared from commercially available carboxylic acid precursors by reduction to the aldehyde[1] followed by iodination:[2a] **7a**,[2a] **7g**,[2b] **7h,j,l**,[2c] **7i**,[2d] **7k**.[2e]

Cross-coupling reactions for the synthesis of precursors 8

A Schlenk tube was charged, under inert atmosphere, with iodide (1.0 equiv), boronic acid (1.2 equiv), K_2CO_3 (8.3 equiv) and $Pd(PPh_3)_4$ (5 mol%). Toluene/ethanol (1:1) (8 mL/mmol iodide) and water (3.3 mL/mmol iodide) were added, and reaction mixture was stirred at 70 °C for overnight. After cooling to room temperature, the rection mixture was quenched with brine and extracted with EtOAc. The combined organic phases were dried over $MgSO_4$, concentrated, and the residue was purified by silica gel column chromatography using different cyclohexane/EtOAc mixtures (typically cyclohexane/EtOAc 6:1).

3-(2-(Methylthio)phenyl)-1*H*-indole-2-carbaldehyde (8a)

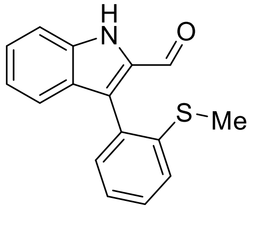

Following the general procedure from 3-iodo-1*H*-indole-2-carbaldehyde (3.0 mmol, 813 mg) and 2-(methylthio)phenylboronic acid (3.6 mmol, 637 mg), purification by column chromatography afforded **8a** as a pale-yellow solid (802 mg, 99%). ^{1}H-NMR (400 MHz, $CDCl_3$) δ 9.82 - 9.65 (m, 2H), 7.58 – 7.50 (m, 2H), 7.51 – 7.33 (m, 4H), 7.28 (t, J = 7.5 Hz, 1H), 7.17 (t, J = 7.5 Hz, 1H), 2.37 (s, 3H). ^{13}C-NMR (100 MHz, $CDCl_3$) δ 182.8, 139.8, 137.5, 132.2, 132.1, 130.2, 129.1, 127.6, 127.3, 127.2, 125.2, 124.6, 122.6, 121.3, 112.7, 15.8. HRMS (ESI) calcd. for $C_{16}H_{13}ONNaS$ ($M + Na^+$) 290.0610. Found 290.0607.

6-Methoxy-3-(2-(methylthio)phenyl)-1*H*-indole-2-carbaldehyde (8g)

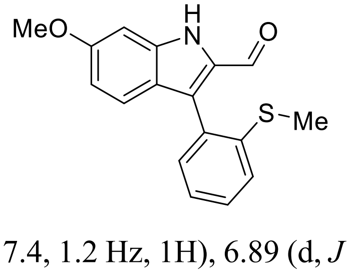

Following the general procedure from 3-iodo-6-methoxi-1*H*-indole-2-carbaldehyde (1.0 mmol, 301 mg) and 2-(methylthio)phenylboronic acid (1.2 mmol, 212 mg), purification by column chromatography afforded **8g** as a pale-yellow solid (287 mg, 97%). ^{1}H-NMR (400 MHz, $CDCl_3$) δ 9.66 (br, 1H), 9.57 (s, 1H), 7.45 (ddd, J = 8.1, 7.3, 1.5 Hz, 1H), 7.38 (dt, J = 8.9, 0.6 Hz, 1H), 7.37 – 7.32 (m, 2H), 7.26 (td, J = 7.4, 1.2 Hz, 1H), 6.89 (d, J = 2.0 Hz, 1H), 6.82 (dd, J = 8.9, 2.2 Hz, 1H), 3.88 (s, 3H), 2.36 (s, 3H). ^{13}C-NMR (100 MHz, $CDCl_3$) δ 181.6, 160.6, 139.7, 138.9, 131.9, 131.6, 130.2, 129.0, 128.1, 125.0,

124.4, 123.5, 121.7, 113.4, 93.7, 55.6, 15.7. HRMS (ESI) calcd. for $C_{17}H_{15}O_2NNaS$ (M + Na^+) 320.0716. Found 320.0713.

5-Methoxy-3-(2-(methylthio)phenyl)-1*H*-indole-2-carbaldehyde (8h)

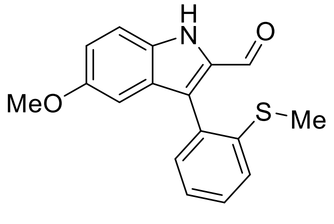

Following the general procedure from 3-iodo-5-methoxi-1*H*-indole-2-carbaldehyde (1.0 mmol, 301 mg) and 2-(methylthio)phenylboronic acid (1.2 mmol, 212 mg), purification by column chromatography afforded **8h** as a pale-yellow solid (288 mg, 97%). ^{1}H-NMR (400 MHz, DMSO-d_6) δ 12.01 (s, 1H), 9.51 (s, 1H), 7.48 (t, *J* = 7.5 Hz, 1H), 7.44 – 7.36 (m, 3H), 7.29 (t, *J* = 7.3 Hz, 1H), 7.04 (dd, *J* = 9.0, 2.1 Hz, 1H), 6.70 (d, *J* = 1.4 Hz, 1H), 3.68 (s, 3H), 2.36 (s, 3H). ^{13}C-NMR (100 MHz, DMSO-d_6) δ 182.1, 154.8, 139.7, 133.6, 133.0, 132.1, 130.4, 129.3, 127.0, 125.3, 124.9, 119.1, 114.6, 101.3, 55.7, 15.2. HRMS (ESI) calcd. for $C_{17}H_{15}O_2NNaS$ (M + Na^+) 320.0716. Found 320.0715.

5-(Benzyloxy)-3-(2-(methylthio)phenyl)-1*H*-indole-2-carbaldehyde (8i)

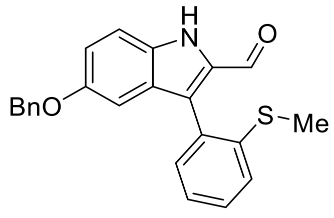

Following the general procedure from 5-(benzyloxy)-3-iodo-1*H*-indole-2-carbaldehyde (1.0 mmol, 377 mg) and 2-(methylthio)phenylboronic acid (1.2 mmol, 212 mg), purification by column chromatography afforded **8i** as a yellow solid (292 mg, 78%). ^{1}H-NMR (400 MHz, $CDCl_3$) δ 9.65 (s, 1H), 9.49 (br, 1H), 7.53 – 7.27 (m, 10H), 7.18 (d, *J* = 8.9 Hz, 1H), 6.97 (s, 1H), 5.07 – 4.95 (m, 2H), 2.37 (s, 3H). ^{13}C-NMR (100 MHz, $CDCl_3$) δ 182.4, 154.2, 139.8, 137.1, 132.9, 132.7, 132.0, 130.2, 129.0, 128.5, 127.9, 127.7, 127.5, 126.4, 125.1, 124.6, 120.1, 113.6, 103.3, 70.6, 15.7. HRMS (ESI) calcd. for $C_{23}H_{19}O_2NNaS$ (M + Na^+) 396.1029. Found 396.1025.

5-Fluoro-3-(2-(methylthio)phenyl)-1*H*-indole-2-carbaldehyde (8j)

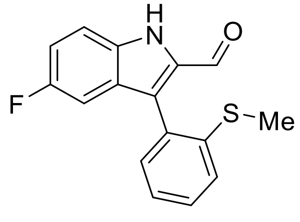

Following the general procedure from 5-chloro-3-iodo-1*H*-indole-2-carbaldehyde (1.0 mmol, 289 mg) and 2-(methylthio)phenylboronic acid (1.2 mmol, 212 mg), purification by column chromatography afforded **8j** as a pale-yellow solid (257 mg, 90%). ^{1}H-NMR (400 MHz, DMSO-d_6) δ 12.25 (s, 1H), 9.57 (s, 1H), 7.54 – 7.47 (m, 2H), 7.41 (dd, *J* = 8.0, 1.1 Hz, 1H), 7.38 (dd, *J* = 7.5, 1.5 Hz, 1H), 7.30 (dd, *J* = 7.4, 1.3 Hz, 1H), 7.25 (td, *J* = 9.0, 2.6 Hz, 1H), 7.04 (dd, *J* = 9.4, 2.6 Hz, 1H), 2.37 (s, 3H). ^{13}C-NMR (100 MHz, DMSO-d_6) δ 182.0, 157.5 (d, *J* = 236 Hz), 139.1, 134.3, 133.5, 131.6, 129.2, 129.1, 126.3 (d, *J* = 10 Hz), 125.0 (d, *J* = 6 Hz), 124.9, 124.5, 115.8 (d, *J* = 27 Hz), 114.6 (d, *J* = 10 Hz), 105.4 (d, *J* = 23.4 Hz), 14.63. ^{19}F-NMR (376 MHz, DMSO-d_6) δ -122.0. HRMS (ESI) calcd. for $C_{16}H_{12}ONFNaS$ (M + Na^+) 308.0516. Found 308.0515.

5-Chloro-3-(2-(methylthio)phenyl)-1*H*-indole-2-carbaldehyde (8k)

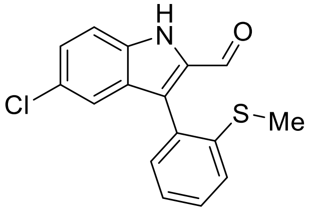

Following the general procedure from 5-chloro-3-iodo-1*H*-indole-2-carbaldehyde (1.0 mmol, 305 mg) and 2-(methylthio)phenylboronic acid (1.2 mmol, 212 mg), purification by column chromatography afforded **8k** as a salmon colour solid (260 mg, 86%). ^{1}H-NMR (400 MHz, $CDCl_3$) δ 9.68 (s, 1H), 9.34 (br, 1H), 7.52 – 7.41 (m, 3H), 7.38 – 7.31 (m, 3H), 7.29 (d, *J* = 7.4 Hz, 1H), 2.37 (s, 3H). ^{13}C-NMR (100 MHz, $CDCl_3$) δ 182.6, 139.8, 135.5, 133.2, 132.0, 129.5, 129.4, 128.3, 128.1, 127.2, 126.0, 125.2, 124.8, 121.7, 113.8, 15.7. HRMS (ESI) calcd. for $C_{16}H_{12}ONClNaS$ (M + Na^+) 324.0220. Found 324.0219.

3-(2-(Ethylthio)phenyl)-1*H*-indole-2-carbaldehyde (8m)

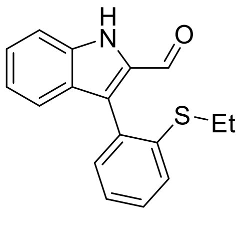

Following the general procedure from 3-iodo-1*H*-indole-2-carbaldehyde (1.0 mmol, 271 mg) and 2-(ethylthio)phenylboronic acid (1.2 mmol, 218 mg), purification by column chromatography afforded **8m** as an orange solid (275 mg, 98%). ^{1}H-NMR (400 MHz, $CDCl_3$) δ 9.73 (s, 1H), 9.51 (br, 1H), 7.59 – 7.37 (m, 6H), 7.34 – 7.29 (m, 1H), 7.18 (t, *J* = 7.5 Hz, 1H), 2.83 (q, *J* = 7.3 Hz, 2H), 1.23 (t, *J* = 7.4 Hz, 3H). ^{13}C-NMR (100 MHz, $CDCl_3$) δ 182.8, 138.3, 137.4, 132.3, 132.2, 131.4, 129.0, 127.6, 127.5, 127.4, 125.2, 122.6, 121.3, 112.6, 27.0, 13.9. HRMS (ESI) calcd. for $C_{17}H_{15}ONNaS$ (M + Na^+) 304.0767. Found 304.0766.

3-(2-(Ethylthio)phenyl)-6-methoxy-1*H*-indole-2-carbaldehyde (8t)

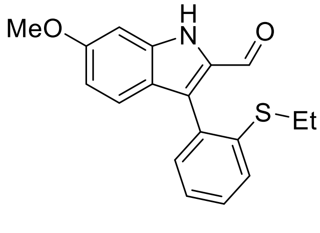

Following the general procedure from 3-iodo-6-methoxy-1*H*-indole-2-carbaldehyde (0.5 mmol, 151 mg) and 2-(ethylthio)phenylboronic acid (0.6 mmol, 109 mg), purification by column chromatography afforded **8t** as a pale-orange solid (146 mg, 94%). ^{1}H-NMR (400 MHz, $CDCl_3$) δ 9.83 (br, 1H), 9.58 (s, 1H), 7.47 – 7.35 (m, 4H), 7.30 – 7.24 (m, 1H), 6.91 (d, *J* = 1.9 Hz, 1H), 6.82 (dd, *J* = 8.9, 2.1 Hz, 1H), 3.88 (s, 3H), 2.81 (q, *J* = 7.3 Hz, 2H), 1.21 (t, *J* = 7.4 Hz, 3H). ^{13}C-NMR (100 MHz, $CDCl_3$) δ 181.8, 160.7, 139.0, 138.2, 132.3, 131.7, 131.5, 128.9, 128.6, 127.4, 125.1, 123.6, 121.9, 113.5, 93.8, 55.7, 27.0, 13.9. HRMS (ESI) calcd. for $C_{18}H_{17}O_2NNaS$ (M + Na^+) 334.0872. Found 334.0872.

5-(Benzyloxy)-3-(2-(ethylthio)phenyl)-1*H*-indole-2-carbaldehyde (8u)

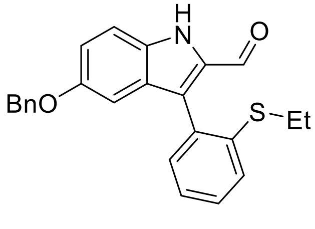

Following the general procedure from 5-benzyloxy-3-iodo-1*H*-indole-2-carbaldehyde (0.8 mmol, 302 mg) and 2-(ethylthio)phenylboronic acid (0.96 mmol, 174 mg), purification by column chromatography afforded **8u** as an orange solid (310 mg, 99%). ^{1}H-NMR (400 MHz, $CDCl_3$) δ 9.65 (s, 1H), 9.28 (br, 1H), 7.48 – 7.27 (m, 10H), 7.17 (dd, *J* = 9.0, 2.3 Hz, 1H), 6.96 (d, *J* = 2.0 Hz, 1H), 5.05 – 4.96 (m, 2H), 2.81

(q, J = 7.1 Hz, 2H), 1.22 (t, J = 7.3 Hz, 3H). ^{13}C-NMR (100 MHz, $CDCl_3$) δ 182.5, 154.3, 138.4, 137.2, 132.9, 132.7, 132.3, 131.4, 129.0, 128.6, 128.1, 127.8, 127.8, 127.4, 126.7, 125.2, 120.1, 113.6, 103.5, 70.7, 26.9, 13.9. HRMS (ESI) calcd. for $C_{24}H_{21}O_2NNaS$ (M + Na^+) 410.1185. Found 410.1178.

3-(2-(Ethylthio)phenyl)-5-fluoro-1*H*-indole-2-carbaldehyde (8v)

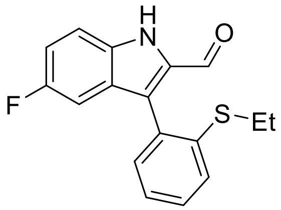

Following the general procedure from 5-fluor-3-iodo-1*H*-indole-2-carbaldehyde (0.8 mmol, 231 mg) and 2-(ethylthio)phenylboronic acid (0.96 mmol, 174 mg), purification by column chromatography afforded **8v** as a brown solid (209 mg, 88%). ^{1}H-NMR (400 MHz, $CDCl_3$) δ 9.70 (s, 2H), 7.49 – 7.41 (m, 3H), 7.36 (d, J = 7.3 Hz, 1H), 7.29 (ddd, J = 7.9, 5.6, 3.1 Hz, 1H), 7.22 – 7.12 (m, 2H), 2.82 (q, J = 7.4 Hz, 2H), 1.22 (t, J = 7.4 Hz, 3H). ^{13}C-NMR (100 MHz, $CDCl_3$) δ 182.8, 158.4 (d, J = 238 Hz), 138.2, 133.9, 133.4, 132.1, 130.7, 129.1, 127.6 (d, J = 10 Hz), 127.3, 127.0 (br s), 125.2, 116.8 (d, J = 27 Hz), 113.7 (d, J = 9 Hz), 106.6 (d, J = 24 Hz), 26.8, 13.7. ^{19}F-NMR (376 MHz, $CDCl_3$) δ -121.5. HRMS (ESI) calcd. for $C_{17}H_{14}ONFNaS$ (M + Na^+) 322.0672. Found 322.0672.

5-Chloro-3-(2-(ethylthio)phenyl)-1*H*-indole-2-carbaldehyde (8x)

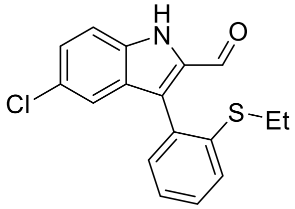

Following the general procedure from 5-chloro3-iodo-1*H*-indole-2-carbaldehyde (0.5 mmol, 153 mg) and 2-(ethylthio)phenylboronic acid (0.6 mmol, 109 mg), purification by column chromatography afforded **8x** as a pale-yellow solid (157 mg, 99%). ^{1}H-NMR (400 MHz, $CDCl_3$) δ 9.70 (s, 1H), 9.65 (s, 1H), 7.50 (d, J = 2.0 Hz, 1H), 7.47 – 7.42 (m, 3H), 7.39 – 7.33 (m, 2H), 7.32 – 7.27 (m, 1H), 2.82 (q, J = 7.3 Hz, 2H), 1.22 (t, J = 7.4 Hz, 3H). ^{13}C-NMR (100 MHz, $CDCl_3$) δ 182.9, 138.3, 135.7, 133.1, 132.2, 130.5, 129.3, 128.3, 128.1, 127.4, 127.1, 126.5, 125.3, 121.7, 113.9, 27.0, 13.8. HRMS (ESI) calcd. for $C_{17}H_{14}ONClNaS$ (M + Na^+) 338.0377. Found 338.0377.

3-(2-(Benzylthio)phenyl)-1*H*-indole-2-carbaldehyde (8z)

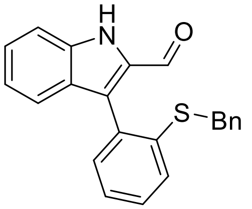

Following the general procedure from 3-iodo-1*H*-indole-2-carbaldehyde (1.0 mmol, 271 mg) and 2-(benzylthio)phenylboronic acid (1.2 mmol, 293 mg), purification by column chromatography afforded **8z** as a pale-yellow solid (329 mg, 96%). ^{1}H-NMR (400 MHz, DMSO-d_6) δ 12.12 (s, 1H), 9.51 (s, 1H), 7.59 (d, J = 7.9 Hz, 1H), 7.50 (d, J = 8.2 Hz, 1H), 7.44 (t, J = 7.6 Hz, 1H), 7.41 – 7.17 (m, 9H), 7.09 (t, J = 7.5 Hz, 1H), 4.12 (s, 2H). ^{13}C-NMR (100 MHz, DMSO-d_6) δ 181.83, 137.48, 137.42, 136.77, 132.06, 131.95, 130.86, 128.77, 128.28, 127.53, 127.05, 126.66, 126.44, 125.54, 125.35, 121.54, 120.66, 113.00, 36.29. HRMS (ESI) calcd. for $C_{22}H_{17}ONNaS$ (M + Na^+) 366.0923. Found 366.0918.

3-(Benzo[*b*]thiophen-7-yl)-1*H*-indole-2-carbaldehyde (8ab)

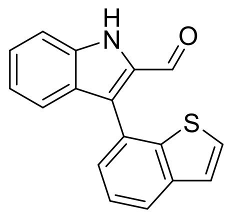

Following the general procedure from 3-iodo-1*H*-indole-2-carbaldehyde (1.5 mmol, 405 mg) and benzo[*b*]thiophen-7-ylboronic acid (1.8 mmol, 327 mg), afforded **8ab** which was used in the next step without further purification.

3-(Dibenzo[*b*,*d*]thiophen-4-yl)-1*H*-indole-2-carbaldehyde (8ac)

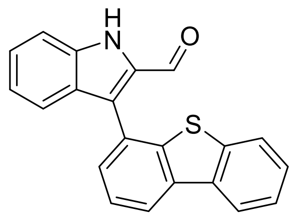

Following the general procedure from 3-iodo-1*H*-indole-2-carbaldehyde (1.5 mmol, 405 mg) and dibenzo[*b*,*d*]thiophen-4-ylboronic acid, (1.8 mmol, 420 mg) purification by column chromatography afforded **8ac** as a pale-yellow solid (480 mg, 98%). ^{1}H-NMR (400 MHz, $CDCl_3$) δ 9.85 (s, 1H), 9.51 (s, 1H), 8.27 (d, *J* = 7.6 Hz, 1H), 8.23 (d, *J* = 7.1 Hz, 1H), 7.79 (d, *J* = 7.1 Hz, 1H), 7.68 – 7.43 (m, 7H), 7.18 (t, *J* = 7.5 Hz, 1H). ^{13}C-NMR (100 MHz, $CDCl_3$) δ 182.4, 141.4, 139.6, 137.5, 136.5, 135.8, 132.0, 129.5, 128.0, 127.3, 127.3, 127.2, 126.7, 124.9, 124.8, 122.9, 122.8, 122.0, 121.6, 121.5, 112.8. HRMS (ESI) calcd. for $C_{21}H_{13}ONNaS$ (M + Na^+) 350.0610. Found 350.0608.

Cross-coupling reactions for the synthesis of precursors 8l and 8y

A Schlenk tube was charged, under inert atmosphere, with iodide (1.0 equiv), boronic acid (1.0 equiv), K_2CO_3 (8.3 equiv) and $Pd(PPh_3)_4$ (5 mol%). Toluene/ethanol (1:1) (8 mL/mmol iodide) and water (3.3 mL/mmol iodide) were added, and reaction mixture was stirred at 50 °C for 3 hours. After cooling to room temperature, the rection mixture was quenched with brine and extracted with EtOAc. The combined organic phases were dried over $MgSO_4$, concentrated, and the residue was purified by silica gel column chromatography using different cyclohexane/EtOAc mixtures (typically cyclohexane/EtOAc 6:1).

5-bromo-3-(2-(methylthio)phenyl)-1*H*-indole-2-carbaldehyde (8l)

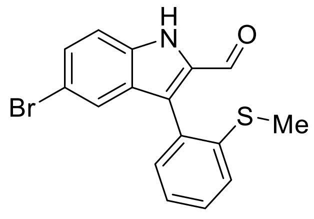

Following the procedure described above from 3-iodo-1*H*-indole-2-carbaldehyde (0.5 mmol, 175 mg) and 2-(methylthio)phenylboronic acid (0.5 mmol, 88 mg), purification by column chromatography afforded **8l** as a salmon colour solid (171 mg, 99%). ^{1}H-NMR (400 MHz, DMSO-d_6) δ 12.36 (s, 1H), 9.59 (s, 1H), 7.54 – 7.37 (m, 6H), 7.30 (t, *J* = 7.4 Hz, 1H), 2.37 (s, 3H). ^{13}C-NMR (100 MHz, DMSO-d_6) δ 182.1, 139.1, 136.1, 133.0, 131.7, 129.2, 128.9, 127.9, 124.9, 124.5, 124.5, 124.3, 123.4, 115.2, 113.1, 14.6. HRMS (ESI) calcd. for $C_{16}H_{12}ONBrNaS$ (M + Na^+) 367.9715. Found 367.9712.

5-Bromo-3-(2-(ethylthio)phenyl)-1*H*-indole-2-carbaldehyde (8y)

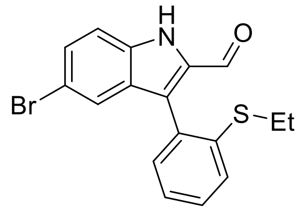

Following the procedure described above from 5-bromo-3-iodo-1*H*-indole-2-carbaldehyde (0.5 mmol, 175 mg) and 2-(ethylthio)phenylboronic acid (0.5 mmol, 91 mg), purification by column chromatography afforded **8y** as a pale-green solid (180 mg, 99%). ^{1}H-NMR (400 MHz, $CDCl_3$) δ 9.69 (s, 1H), 9.49 (br, 1H), 7.66 (d, *J* = 1.9 Hz, 1H), 7.48 (dd, *J* = 8.8, 1.9 Hz, 1H), 7.46 – 7.34 (m, 4H), 7.32 – 7.28 (m, 1H), 2.82 (q, *J* = 7.4 Hz, 2H), 1.22 (t, *J* = 7.4 Hz, 3H). ^{13}C-NMR (100 MHz, $CDCl_3$) δ 182.8, 138.4, 135.8, 132.9, 132.2, 130.5, 129.3, 129.0, 127.4, 126.2, 125.3, 124.9, 114.6, 114.2, 27.0, 13.9. HRMS (ESI) calcd. for $C_{17}H_{14}ONBrNaS$ (M + Na^+) 381.9872. Found 381.9869.

***N*-benzylation reactions for the synthesis of substrates 1**

Over a solution of 3-aryl-1*H*-indole-2-carbaldehyde derivative **8a-z** (1 equiv) and DMSO (4 mL/mmol) in a Schlenk tube, were added, under air, KOH (2 equiv) and benzyl bromide derivative (1.2-2 eq.), and the reaction mixture was stirred at room temperature until total consumption of starting material, monitored by TLC (typically 2-6 h). The reaction mixture was then quenched by addition of water and extracted with EtOAc (x3). The combined organic phases were dried over $MgSO_4$, concentrated, and the residue was purified by silica gel column chromatography using different cyclohexane/EtOAc mixtures (typically cyclohexane/EtOAc 12:1).

1-Benzyl-3-(2-(methylthio)phenyl)-1*H*-indole-2-carbaldehyde (1a)

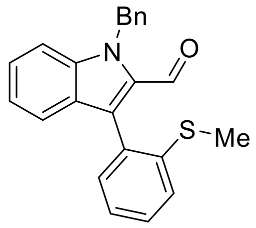

Following the general procedure from **8a** (1.9 mmol, 508 mg) and benzyl bromide (3.8 mmol, 461 μL), purification by column chromatography afforded **1a** as a pale-yellow solid (679 mg, 99%). ^{1}H-NMR (400 MHz, $CDCl_3$) δ 9.71 (s, 1H), 7.51 (d, *J* = 8.1 Hz, 1H), 7.45 (td, *J* = 7.6, 1.3 Hz, 1H), 7.42 – 7.12 (m, 11H), 5.98 – 5.87 (m, 2H), 2.35 (s, 3H). ^{13}C-NMR (100 MHz, $CDCl_3$) δ 183.6, 140.2, 139.6, 138.0, 132.1, 131.0, 130.3, 129.8, 129.1, 128.7, 127.6, 127.4, 126.7, 126.5, 124.9, 124.5, 122.5, 121.3, 111.2, 48.2, 15.7. HRMS (ESI) calcd. for $C_{23}H_{19}ONNaS$ (M + Na^+) 380.1080. Found 380.1075.

1-(4-Bromobenzyl)-3-(2-(methylthio)phenyl)-1*H*-indole-2-carbaldehyde (1b)

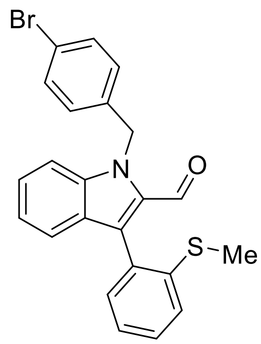

Following the general procedure from **8a** (0.40 mmol, 107 mg) and 4-bromobenzyl bromide (0.80 mmol, 204 mg), purification by column chromatography afforded **1b** as a pale-yellow solid (103 mg, 60%). ^{1}H-NMR (400 MHz, $CDCl_3$) δ 9.72 (s, 1H), 7.53 (d, *J* = 8.1 Hz, 1H), 7.50 – 7.32 (m, 7H), 7.29 (d, *J* = 7.4 Hz, 1H), 7.18 (t, *J* = 6.9 Hz, 1H), 7.05 (d, *J* = 8.0 Hz, 2H), 5.90 (d, *J* = 16.1 Hz, 1H), 5.83 (d, *J* = 16.2 Hz, 1H), 2.37 (s, 3H). ^{13}C-NMR (100 MHz, $CDCl_3$) δ 183.5, 140.1, 139.4, 137.1, 132.0, 131.8, 130.9,

130.0, 130.0, 129.2, 128.4, 127.7, 126.5, 124.8, 124.4, 122.6, 121.5, 121.3, 110.8, 47.6, 15.6.

3-(2-(Methylthio)phenyl)-1-(4-(trifluoromethyl)benzyl)-1*H*-indole-2-carbaldehyde (1c)

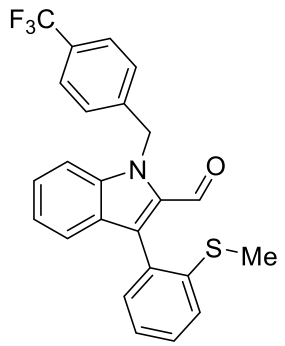

Following the general procedure from **8a** (0.40 mmol, 107 mg) and 4-(trifluoromethyl)benzyl bromide (0.80 mmol, 126 μL), purification by column chromatography afforded **1c** as a pale-yellow solid (151 mg, 90%). ^{1}H-NMR (400 MHz, $CDCl_3$) δ 9.70 (s, 1H), 7.64 (d, *J* = 8.1 Hz, 1H), 7.57 – 7.32 (m, 7H), 7.29 (d, *J* = 7.4 Hz, 1H), 7.24 (d, *J* = 8.1 Hz, 2H), 7.19 (t, *J* = 7.4 Hz, 1H), 6.02 (d, *J* = 16.5 Hz, 1H), 5.92 (d, *J* = 16.5 Hz, 1H), 2.38 (s, 3H). ^{13}C-NMR (100 MHz, $CDCl_3$) δ 183.5, 142.0, 140.1, 139.4, 132.0, 130.8, 130.1, 129.8, 129.6 (q, *J* = 32 Hz), 129.2, 127.8, 126.6 (q, *J* = 218 Hz), 128.0, 126.5, 125.7, 125.7, 124.7, 124.4, 122.6, 121.5, 110.6, 47.7, 15.5. ^{19}F -NMR (376 MHz, $CDCl_3$) δ -62.5. HRMS (ESI) calcd. for $C_{24}H_{18}ONF_3NaS$ (M + Na^+) 448.0953. Found 448.0949.

3-(2-(methylthio)phenyl)-1-(4-(trifluoromethoxy)benzyl)-1*H*-indole-2-carbaldehyde (1d)

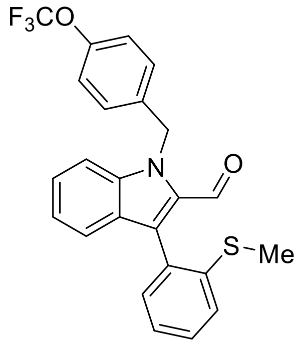

Following the general procedure from **8a** (0.40 mmol, 107 mg) and 4-(trifluoromethoxi)benzyl bromide (0.80 mmol, 132 μL), purification by column chromatography afforded **1d** as a pale-yellow solid (168 mg, 95%). ^{1}H-NMR (400 MHz, $CDCl_3$) δ 9.71 (s, 1H), 7.53 (d, *J* = 8.1 Hz, 1H), 7.50 – 7.33 (m, 5H), 7.30 – 7.25 (m, 1H), 7.22 – 7.11 (m, 5H), 5.95 (d, *J* = 16.2 Hz, 1H), 5.87 (d, *J* = 16.2 Hz, 1H), 2.37 (s, 3H). ^{13}C-NMR (100 MHz, $CDCl_3$) δ 183.5, 148.4, 140.1, 139.4, 136.7, 132.0, 130.8, 130.0, 129.9, 129.2, 128.0, 127.7, 126.5, 124.7, 124.4, 122.6, 121.4, 121.2, 120.5 (q, *J* = 255 Hz), 47.4, 15.5. ^{19}F -NMR (376 MHz, $CDCl_3$) δ -57.8. HRMS (ESI) calcd. for $C_{24}H_{18}O_2NF_3NaS$ (M + Na^+) 464.0903. Found 464.0898.

1-Benzyl-6-methoxy-3-(2-(methylthio)phenyl)-1*H*-indole-2-carbaldehyde (1g)

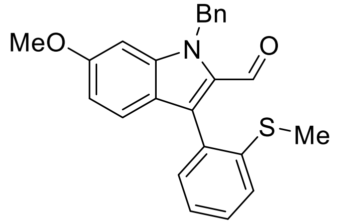

Following the general procedure from **8g** (0.40 mmol, 120 mg) and benzyl bromide (0.80 mmol, 97 μL), purification by column chromatography afforded **1g** as a pale-yellow foam (120 mg, 77%). ^{1}H-NMR (400 MHz, $CDCl_3$) δ 9.60 (s, 1H), 7.45 (t, *J* = 7.6 Hz, 1H), 7.41 – 7.22 (m, 7H), 7.19 (d, *J* = 7.4 Hz, 2H), 6.83 (dd, *J* = 8.9, 1.7 Hz, 1H), 6.75 (d, *J* = 2.2 Hz, 1H), 5.95 – 5.84 (m, 2H), 3.82 (s, 3H), 2.37 (s, 3H). ^{13}C-NMR (100 MHz, $CDCl_3$) δ 182.5, 160.6, 141.0, 140.1, 137.9, 132.1, 130.7, 130.6, 130.3, 129.1, 128.7, 127.3, 126.7, 124.8, 124.4, 123.5, 121.0, 113.1, 92.5, 55.6, 48.2, 15.6. HRMS (ESI) calcd. for $C_{24}H_{21}O_2NNaS$ (M + Na^+) 410.1185. Found 410.1180.

1-Benzyl-5-methoxy-3-(2-(methylthio)phenyl)-1*H*-indole-2-carbaldehyde (1h)

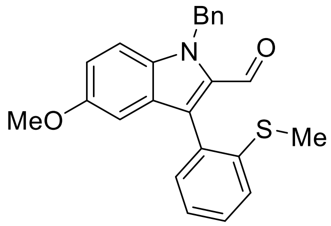

Following the general procedure from **8h** (0.40 mmol, 120 mg) and benzyl bromide (0.80 mmol, 97 μL), purification by column chromatography afforded **1h** as a pale-orange solid (112 mg, 72%). ^{1}H-NMR (400 MHz, $CDCl_3$) δ 9.66 (s, 1H), 7.47 (t, *J* = 7.6 Hz, 1H), 7.41 – 7.20 (m, 7H), 7.18 – 7.12 (m, 2H), 7.07 (dd, *J* = 9.1, 2.3 Hz, 1H), 6.83 (d, *J* = 2.1 Hz, 1H), 5.92 (d, *J* = 16.5 Hz, 1H), 5.88 (d, *J* = 16.3 Hz, 1H), 3.76 (s, 3H), 2.38 (s, 3H). ^{13}C-NMR (100 MHz, $CDCl_3$) δ 183.3, 155.2, 140.2, 138.0, 135.1, 132.0, 131.2, 130.3, 129.0, 128.9, 128.7, 127.3, 126.7, 126.6, 124.8, 124.4, 119.6, 112.1, 101.5, 55.7, 48.2, 15.6. HRMS (ESI) calcd. for $C_{24}H_{21}O_2NNaS$ (M + Na^+) 410.1185. Found 410.1182.

1-Benzyl-5-(benzyloxy)-3-(2-(methylthio)phenyl)-1*H*-indole-2-carbaldehyde (1i)

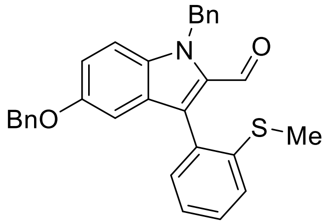

Following the general procedure from **8i** (0.40 mmol, 149 mg) and benzyl bromide (0.80 mmol, 97 μL), purification by column chromatography afforded **1i** as a pale-yellow solid (142mg, 78%). ^{1}H-NMR (400 MHz, $CDCl_3$) δ 9.65 (s, 1H), 7.46 (td, *J* = 7.8, 1.4 Hz, 1H), 7.43 – 7.20 (m, 12H), 7.17 – 7.11 (m, 3H), 6.92 (d, *J* = 2.4 Hz, 1H), 5.94 – 5.83 (m, 2H), 5.05 – 4.92 (m, 2H), 2.36 (s, 3H). ^{13}C-NMR (100 MHz, $CDCl_3$) δ 183.4, 154.4, 140.3, 138.1, 137.1, 135.3, 132.1, 131.4, 130.4, 129.1, 129.0, 128.8, 128.6, 128.1, 127.8, 127.4, 126.7, 126.7, 124.8, 124.5, 120.1, 112.2, 103.1, 70.7, 48.3, 15.7. HRMS (ESI) calcd. for $C_{30}H_{25}O_2NNaS$ (M + Na^+) 486.1498. Found 486.1491.

1-Benzyl-5-fluoro-3-(2-(methylthio)phenyl)-1*H*-indole-2-carbaldehyde (1j)

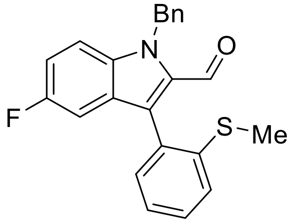

Following the general procedure from **8j** (0.40 mmol, 114 mg) and benzyl bromide (0.80 mmol, 97 μL), purification by column chromatography afforded **1j** as a pale-yellow solid (123 mg, 83%). ^{1}H-NMR (400 MHz, $CDCl_3$) δ 9.71 (s, 1H), 7.46 (t, *J* = 7.6 Hz, 1H), 7.38 – 7.22 (m, 7H), 7.19 – 7.10 (m, 4H), 5.94 (d, *J* = 16.1 Hz, 1H), 5.89 (d, *J* = 16.1 Hz, 1H), 2.38 (s, 3H). ^{13}C-NMR (100 MHz, $CDCl_3$) δ 183.5, 158.5 (d, *J* = 239 Hz), 140.1, 137.6, 136.1, 132.1, 131.9, 129.6, 129.3, 129.1 (d, *J* = 6 Hz), 128.7, 127.4, 126.7, 126.5, 124.8, 124.5, 116.7 (d, *J* = 27 Hz), 112.2 (d, *J* = 9 Hz), 106.5 (d, *J* = 24 Hz), 48.3, 15.5. ^{19}F-NMR (376 MHz, $CDCl_3$) δ -121.56. HRMS (ESI) calcd. for $C_{23}H_{18}ONFNaS$ (M + Na^+) 398.0985. Found 398.0977.

1-Benzyl-5-chloro-3-(2-(methylthio)phenyl)-1*H*-indole-2-carbaldehyde (1k)

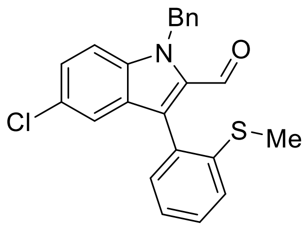

Following the general procedure from **8k** (0.40 mmol, 122 mg) and benzyl bromide (0.80 mmol, 97 μL), purification by column chromatography afforded **1k** as a pale-yellow solid (70 mg, 44%). ^{1}H-NMR (400 MHz, $CDCl_3$) δ 9.71 (s, 1H), 7.51 – 7.44 (m, 2H), 7.42 – 7.21 (m, 8H), 7.19 – 7.10 (m, 2H), 5.93 (d, *J* = 16.1 Hz, 1H), 5.88 (d, *J* = 16.1 Hz, 1H), 2.38 (s, 3H). ^{13}C-NMR (100 MHz, $CDCl_3$) δ 174.5, 139.4, 137.9, 136.8, 136.4, 132.2, 131.7, 128.8, 128.5, 128.4, 127.3, 126.1, 125.9, 124.7, 124.5, 123.1, 119.1, 114.8, 111.6, 81.0, 70.2, 48.6, 45.6, 28.1, 15.3, 14.7. HRMS (ESI) calcd. for $C_{23}H_{18}ONClNaS$ (M + Na^+) 414.0690. Found 414.0685.

1-Benzyl-5-bromo-3-(2-(methylthio)phenyl)-1*H*-indole-2-carbaldehyde (1l)

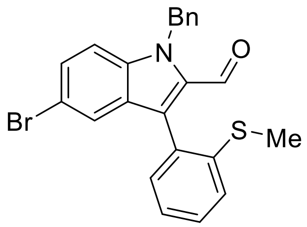

Following the general procedure from **8l** (0.5 mmol, 171 mg) and benzyl bromide (1.0 mmol, 121 μL), purification by column chromatography afforded **1l** as a pale-yellow solid (157 mg, 72%). ^{1}H-NMR (400 MHz, $CDCl_3$) δ 9.70 (s, 1H), 7.63 (dd, *J* = 1.9, 0.6 Hz, 1H), 7.50 – 7.43 (m, 2H), 7.36 – 7.21 (m, 7H), 7.15 – 7.09 (m, 2H), 5.92 (d, *J* = 16.1 Hz, 1H), 5.87 (d, *J* = 16.1 Hz, 1H), 2.37 (s, 3H). ^{13}C-NMR (100 MHz, $CDCl_3$) δ 183.6, 140.2, 138.1, 137.6, 132.0, 131.7, 130.4, 129.4, 128.9, 128.7, 128.0, 127.6, 126.6, 124.9, 124.7, 124.6, 114.7, 112.8, 48.4, 15.6. HRMS (ESI) calcd. for $C_{23}H_{18}ONBrNaS$ (M + Na^+) 458.0185. Found 458.0183.

1-Benzyl-3-(2-(ethylthio)phenyl)-1*H*-indole-2-carbaldehyde (1m)

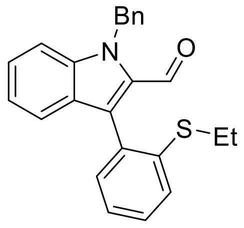

Following the general procedure from **8m** (0.40 mmol, 113 mg) and benzyl bromide (0.80 mmol, 97 μL), purification by column chromatography afforded **1m** as a pale-yellow foam (121 mg, 83%). ^{1}H-NMR (400 MHz, $CDCl_3$) δ 9.71 (s, 1H), 7.50 (d, *J* = 8.1 Hz, 1H), 7.45 – 7.36 (m, 5H), 7.31 – 7.21 (m, 4H), 7.18 – 7.12 (m, 3H), 5.93 (s, 2H), 2.80 (q, *J* = 7.4 Hz, 2H), 1.21 (t, *J* = 7.4 Hz, 3H). ^{13}C-NMR (100 MHz, $CDCl_3$) δ 183.6, 139.5, 138.6, 138.0, 132.3, 131.4, 130.9, 130.1, 128.9, 128.6, 127.4, 127.3, 127.2, 126.6, 125.0, 122.4, 121.2, 111.0, 48.1, 26.8, 13.8. HRMS (ESI) calcd. for $C_{24}H_{21}ONNaS$ (M + Na^+) 394.1236. Found 394.1236.

1-(4-Bromobenzyl)-3-(2-(ethylthio)phenyl)-1*H*-indole-2-carbaldehyde (1o)

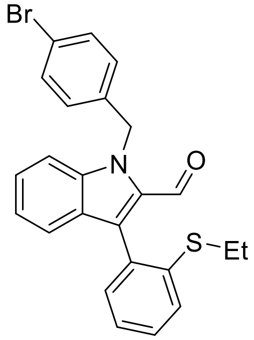

Following the general procedure from **8m** (0.40 mmol, 113 mg) and 4-bromobenzyl bromide (0.48 mmol, 122 mg), purification by column chromatography afforded **1o** as an orange oil (140 mg, 78%). ^{1}H-NMR (400 MHz, $CDCl_3$) δ 9.69 (s, 1H), 7.50 (dt, *J* = 8.1, 0.8 Hz, 1H), 7.45 – 7.35 (m, 7H), 7.31 – 7.27 (m, 1H), 7.17 (ddd, *J* = 8.0, 6.5, 1.4 Hz, 1H), 7.06 – 7.00 (m, 2H), 5.94 – 5.73 (m, 2H), 2.80 (q, *J* = 7.4 Hz, 2H), 1.21 (t, *J* = 7.4 Hz, 3H). ^{13}C-NMR (100 MHz, $CDCl_3$) δ 183.7, 139.5, 138.7, 137.2, 132.4, 131.9, 131.3, 130.9, 130.5, 129.1, 128.5, 127.7, 127.2, 126.8, 125.1, 122.7, 121.5, 121.3, 110.9, 47.7, 26.9, 13.9. HRMS (ESI) calcd. for $C_{24}H_{20}ONBrNaS$ (M + Na^+) 472.0341. Found 472.0334.

1-(2-Bromobenzyl)-3-(2-(ethylthio)phenyl)-1*H*-indole-2-carbaldehyde (1p)

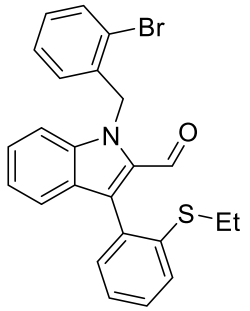

Following the general procedure from **8m** (0.40 mmol, 113 mg) and 4-bromobenzyl bromide (0.48 mmol, 122 mg), purification by column chromatography afforded **1p** as a yellow foam (140 mg, 78%). ^{1}H-NMR (400 MHz, $CDCl_3$) δ 9.71 (s, 1H), 7.63 – 7.59 (m, 1H), 7.53 (dt, *J* = 8.1, 0.9 Hz, 1H), 7.46 – 7.40 (m, 3H), 7.40 – 7.36 (m, 1H), 7.34 – 7.27 (m, 2H), 7.18 (ddd, *J* = 8.0, 6.9, 0.9 Hz, 1H), 7.12 – 7.06 (m, 2H), 6.51 – 6.38 (m, 1H), 6.02 (d, *J* = 17.3 Hz, 1H), 5.92 (d, *J* = 17.3 Hz, 1H), 2.83 (q, *J* = 7.4 Hz, 2H), 1.23 (t, *J* = 7.4 Hz, 3H). ^{13}C-NMR (100 MHz, $CDCl_3$) δ 183.5, 139.5, 138.8, 137.2, 132.8, 132.3, 131.3, 131.2, 130.2, 129.1, 128.7, 127.9, 127.8, 127.2, 126.9, 126.7, 125.1, 122.6, 122.0, 121.6, 110.9, 48.7, 27.0, 13.9. HRMS (ESI) calcd. for $C_{24}H_{20}ONBrNaS$ (M + Na^+) 472.0341. Found 472.0339.

1-(4-(*Tert*-butyl)benzyl)-3-(2-(ethylthio)phenyl)-1*H*-indole-2-carbaldehyde (1q)

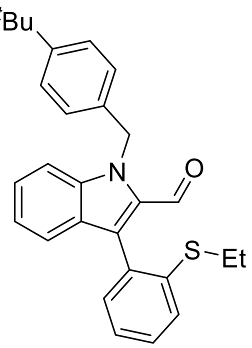

Following the general procedure from **8m** (0.40 mmol, 113 mg) and 4-tert-butylbenzyl bromide (0.48 mmol, 91 μL), purification by column chromatography afforded **1q** as an orange oil (120 mg, 70%). ^{1}H-NMR (400 MHz, $CDCl_3$) δ 9.72 (s, 1H), 7.50 (dt, *J* = 8.1, 1.0 Hz, 1H), 7.46 – 7.37 (m, 5H), 7.31 – 7.27 (m, 3H), 7.15 (ddd, *J* = 8.0, 6.7, 1.2 Hz, 1H), 7.12 – 7.09 (m, 2H), 5.90 (s, 2H), 2.80 (q, *J* = 7.4 Hz, 2H), 1.28 (s, 9H), 1.21 (t, *J* = 7.4 Hz, 3H). ^{13}C-NMR (100 MHz, $CDCl_3$) δ 183.7, 150.2, 139.6, 138.7, 135.1, 132.5, 131.6, 131.0, 130.1, 129.0, 127.4, 127.3, 126.7, 126.5, 125.6, 125.1, 122.5, 121.2, 111.2, 47.9, 34.6, 31.5, 26.9, 13.9. HRMS (ESI) calcd. for $C_{28}H_{29}ONNaS$ (M + Na^+) 450.1862. Found 450.1865.

3-(2-(Ethylthio)phenyl)-1-(4-methoxybenzyl)-1*H*-indole-2-carbaldehyde (1r)

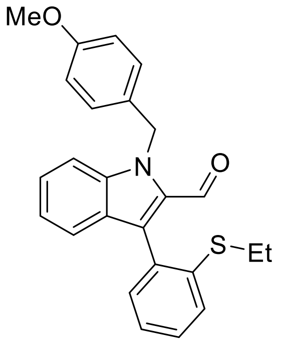

Following the general procedure from **8m** (0.40 mmol, 113 mg) and 4-methoxybenzyl bromide (0.80 mmol, 117 µL), purification by column chromatography afforded **1r** as a yellow oil (83 mg, 52%). ^{1}H-NMR (400 MHz, $CDCl_3$) δ 9.70 (s, 1H), 7.48 (dt, *J* = 8.1, 0.9 Hz, 1H), 7.46 – 7.35 (m, 5H), 7.30 – 7.24 (m, 1H), 7.18 – 7.10 (m, 3H), 6.85 – 6.78 (m, 2H), 5.85 (s, 2H), 3.76 (s, 3H), 2.79 (q, *J* = 7.4 Hz, 2H), 1.20 (t, *J* = 7.4 Hz, 3H). ^{13}C-NMR (100 MHz, $CDCl_3$) δ 183.7, 159.0, 139.5, 138.7, 132.4, 131.5, 130.9, 130.2, 129.0, 128.2, 127.5, 127.2, 126.7, 125.1, 122.5, 121.3, 114.1, 111.2, 55.4, 47.7, 26.9, 13.9. HRMS (ESI) calcd. for $C_{25}H_{23}O_2NNaS$ (M + Na^+) 424.1342. Found 424.1338.

1-Benzyl-3-(2-(ethylthio)phenyl)-6-methoxy-1*H*-indole-2-carbaldehyde (1t)

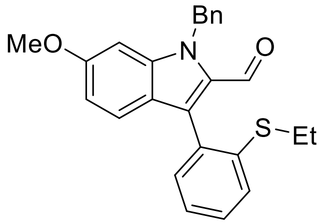

Following the general procedure from **8t** (0.40 mmol, 125 mg) and benzyl bromide (0.60 mmol, 73 µL), purification by column chromatography afforded **1t** as a yellow oil (136 mg, 85%). ^{1}H-NMR (400 MHz, $CDCl_3$) δ 9.56 (s, 1H), 7.44 – 7.39 (m, 2H), 7.35 (d, *J* = 7.6 Hz, 2H), 7.31 – 7.21 (m, 4H), 7.16 (d, *J* = 7.2 Hz, 2H), 6.80 (d, *J* = 8.8 Hz, 1H), 6.72 (s, 1H), 5.88 (s, 2H), 3.81 (s, 3H), 2.79 (q, *J* = 7.4 Hz, 2H), 1.20 (t, *J* = 7.3 Hz, 3H). ^{13}C-NMR (100 MHz, $CDCl_3$) δ 182.5, 160.6, 140.9, 138.6, 138.0, 132.4, 131.6, 131.1, 130.6, 129.0, 128.7, 127.4, 127.2, 126.7, 125.0, 123.6, 121.2, 113.1, 92.5, 55.7, 48.2, 26.9, 13.9. HRMS (ESI) calcd. for $C_{25}H_{23}O_2NNaS$ (M + Na^+) 424.1342. Found 424.1336.

1-Benzyl-5-benzyloxy-3-(2-(ethylthio)phenyl)-1*H*-indole-2-carbaldehyde (1u)

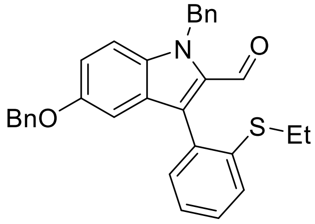

Following the general procedure from **8u** (0.42 mmol, 165 mg) and benzyl bromide (0.50 mmol, 61 µL), purification by column chromatography afforded **1u** as a brown foam (69 mg, 36%). ^{1}H-NMR (400 MHz, $CDCl_3$) δ 9.69 (s, 1H), 7.48 – 7.21 (m, 13H), 7.18 – 7.14 (m, 3H), 6.95 (d, *J* = 2.2 Hz, 1H), 5.91 (s, 2H), 4.99 (s, 2H), 2.83 (q, *J* = 7.3 Hz, 2H), 1.24 (t, *J* = 7.4 Hz, 3H). ^{13}C-NMR (100 MHz, $CDCl_3$) δ 183.4, 154.4, 138.8, 138.1, 137.1, 135.2, 132.4, 131.5, 131.4, 129.4, 128.9, 128.7, 128.6, 128.1, 127.8, 127.4, 127.1, 126.9, 126.7, 125.0, 120.0, 112.2, 103.2, 70.7, 48.3, 26.8, 13.9. HRMS (ESI) calcd. for $C_{31}H_{27}O_2NNaS$ (M + Na^+) 500.1655. Found 500.1649.

1-Benzyl-3-(2-(ethylthio)phenyl)-5-fluor-1*H*-indole-2-carbaldehyde (1v)

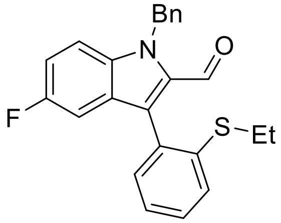

Following the general procedure from **8v** (0.21 mmol, 62 mg) and benzyl bromide (0.25 mmol, 31 μL), purification by column chromatography afforded **1v** as a yellow foam (65 mg, 80%). ^{1}H-NMR (400 MHz, $CDCl_3$) δ 9.72 (s, 1H), 7.44 (d, J = 3.9 Hz, 2H), 7.40 – 7.20 (m, 6H), 7.18 – 7.12 (m, 3H), 5.92 (s, 2H), 2.83 (q, J = 7.3 Hz, 2H), 1.24 (t, J = 7.4 Hz, 3H). ^{13}C-NMR (100 MHz, $CDCl_3$) δ 183.6, 158.5 (d, J = 239 Hz), 138.6, 137.7, 136.1, 132.2, 132.0, 130.8, 129.5 (d, J = 6 Hz), 129.1, 128.7, 127.4, 127.1, 126.7 (d, J = 10 Hz), 126.5, 125.0, 116.7 (d, J = 27 Hz), 112.2 (d, J = 9 Hz), 106.5 (d, J = 24 Hz), 48.3, 26.8, 13.8. ^{19}F-NMR (376 MHz, $CDCl_3$) δ -121.6.

1-Benzyl-5-chloro-3-(2-(ethylthio)phenyl)-1*H*-indole-2-carbaldehyde (1x)

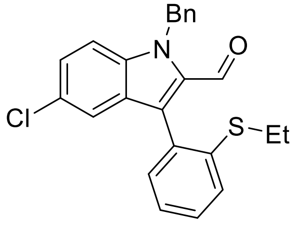

Following the general procedure from **8x** (0.40 mmol, 126 mg) and benzyl bromide (0.48 mmol, 58 μL), purification by column chromatography afforded **1x** as a yellow oil (48 mg, 30%). ^{1}H-NMR (400 MHz, $CDCl_3$) δ 9.69 (s, 1H), 7.46 (s, 1H), 7.44 – 7.27 (m, 8H), 7.11 (d, J = 7.1 Hz, 2H), 5.90 (s, 2H), 2.81 (q, J = 7.4 Hz, 2H), 1.23 (t, J = 7.4 Hz, 3H). ^{13}C-NMR (100 MHz, $CDCl_3$) δ 183.7, 138.8, 137.8, 137.6, 132.3, 131.8, 130.6, 129.3, 129.1, 128.8, 128.0, 127.6, 127.5, 127.2, 126.6, 125.2, 121.6, 112.4, 48.4, 26.9, 13.9. HRMS (ESI) calcd. for $C_{24}H_{20}ONClNaS$ (M + Na^+) 428.0846. Found 428.0843.

1-Benzyl-5-bromo-3-(2-(ethylthio)phenyl)-1*H*-indole-2-carbaldehyde (1y)

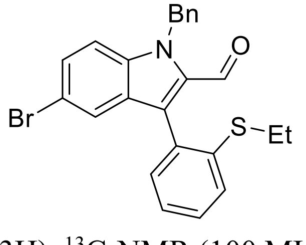

Following the general procedure from **8y** (0.40 mmol, 144 mg) and benzyl bromide (0.48 mmol, 58 μL), purification by column chromatography afforded **1y** as a pale-yellow foam (125 mg, 70%). ^{1}H-NMR (400 MHz, $CDCl_3$) δ 9.71 (s, 1H), 7.63 (d, J = 1.6 Hz, 1H), 7.47 – 7.41 (m, 3H), 7.36 (d, J = 7.2 Hz, 1H), 7.31 – 7.21 (m, 4H), 7.12 (d, J = 7.1 Hz, 2H), 5.90 (s, 2H), 2.82 (q, J = 7.4 Hz, 2H), 1.23 (t, J = 7.4 Hz, 3H). ^{13}C-NMR (100 MHz, $CDCl_3$) δ 183.6, 138.8, 138.1, 137.6, 132.3, 131.7, 130.6, 130.4, 129.3, 129.0, 128.8, 128.1, 127.6, 127.1, 126.6, 125.2, 124.7, 114.7, 112.8, 48.3, 26.9, 13.9. HRMS (ESI) calcd. for $C_{24}H_{20}ONBrNaS$ (M + Na^+) 472.0341. Found 472.0336.

1-Benzyl-3-(2-(benzylthio)phenyl)-1*H*-indole-2-carbaldehyde (1z)

Following the general procedure from **8z** (0.40 mmol, 140 mg) and benzyl bromide (0.64 mmol, 77 μL), purification by column chromatography afforded **1z** as a pale-yellow foam (112 mg, 65%).

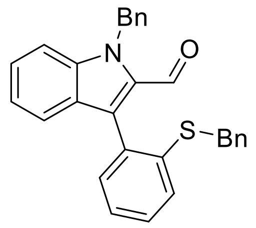

^{1}H-NMR (400 MHz, $CDCl_3$) δ 9.60 (s, 1H), 7.52 – 7.45 (m, 2H), 7.44 – 7.36 (m, 4H), 7.33 – 7.12 (m, 12H), 5.95 (d, *J* = 16.1 Hz, 1H), 5.88 (d, *J* = 16.1 Hz, 1H), 4.01 – 3.91 (m, 2H). ^{13}C-NMR (100 MHz, $CDCl_3$) δ 183.5, 139.5, 138.1, 136.8, 132.5, 132.2, 131.0, 130.1, 129.1, 129.0, 128.9, 128.7, 128.5, 127.5, 127.4, 127.3, 126.8, 126.7, 125.8, 122.5, 121.4, 111.1, 48.2, 38.5. HRMS (ESI) calcd. for $C_{29}H_{23}ONNaS$ (M + Na^+) 456.1393. Found 456.1389.

3-(Benzo[*b*]thiophen-7-yl)-1-benzyl-1H-indole-2-carbaldehyde (1ab)

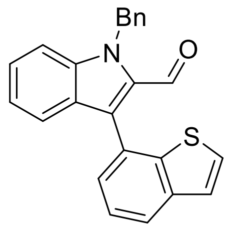

Following the general procedure from **8ab** (0.43 mmol, 118 mg) and benzyl bromide (0.86 mmol, 104 μL), purification by column chromatography afforded **1ab** as a pale-yellow foam (130 mg, 82%). ^{1}H-NMR (400 MHz, $CDCl_3$) δ 9.81 (s, 1H), 7.89 (dd, *J* = 7.8, 1.1 Hz, 1H), 7.56 (d, *J* = 8.2 Hz, 1H), 7.52 – 7.37 (m, 6H), 7.31 – 7.10 (m, 6H), 5.94 (s, 2H). ^{13}C-NMR (100 MHz, $CDCl_3$) δ 183.3, 142.1, 140.3, 139.7, 138.0, 130.8, 130.4, 128.8, 127.9, 127.5, 127.3, 127.2, 127.0, 126.8, 125.9, 124.6, 124.3, 123.8, 122.7, 121.4, 111.3, 48.3. HRMS (ESI) calcd. for $C_{24}H_{17}ONNaS$ (M + Na^+) 390.0923. Found 390.0923.

1-Benzyl-3-(dibenzo[*b*,*d*]thiophen-4-yl)-1*H*-indole-2-carbaldehyde (1ac)

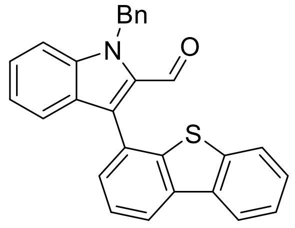

Following the general procedure from **8ac** (0.42 mmol, 138 mg) and benzyl bromide (0.84 mmol, 102 μL), purification by column chromatography afforded **1ac** as a pale-yellow foam (131 mg, 75%). ^{1}H-NMR (400 MHz, $CDCl_3$) δ 9.90 (s, 1H), 8.29 (dd, *J* = 7.6, 1.5 Hz, 1H), 8.27 – 8.24 (m, 1H), 7.84 – 7.80 (m, 1H), 7.68 – 7.63 (m, 2H), 7.61 (dd, *J* = 7.3, 1.5 Hz, 1H), 7.56 – 7.45 (m, 4H), 7.38 – 7.17 (m, 6H), 6.01 (s, 2H). ^{13}C-NMR (100 MHz, $CDCl_3$) δ 183.3, 141.8, 139.8, 139.6, 137.9, 136.3, 135.8, 130.8, 130.1, 129.7, 128.8, 127.9, 127.5, 127.3, 127.2, 126.8, 125.9, 124.8, 124.8, 122.9, 122.7, 122.0, 121.6, 121.5, 111.3, 48.3. HRMS (ESI) calcd. for $C_{28}H_{19}ONNaS$ (M + Na^+) 440.1080. Found 440.1074.

***N*-methylation for the synthesis of 1e**

3-(2-(methylthio)phenyl)-1*H*-indole-2-carbaldehyde **8a** (0.40 mmol, 107 mg) was dissolved in dry THF (1.3 mL) under inert atmosphere and, at 0 ºC, sodium hydride (60% suspension in mineral oil, 0.60 mmol, 24 mg) was slowly added. The reaction was stirred at 0 ºC for 15 min, then it was allowed to warm to r.t. and stirred for 1.5 h. The mixture was then cooled back to 0 ºC, methyl iodide (0.52 mmol, 32 μL) was added and the reaction was stirred at r.t., overnight. After cooling again to 0 ºC, the reaction was quenched by addition of water and extracted with EtOAc (x3). The combined organic phases were dried over $MgSO_4$, concentrated, and the residue was purified by silica gel column chromatography using cyclohexane/EtOAc (9:1) as eluent.

1-Methyl-3-(2-(methylthio)phenyl)-1*H*-indole-2-carbaldehyde (1e)

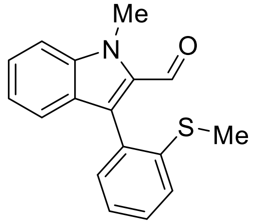

Following the procedure described above, purification by column chromatography afforded **1e** as a white solid (95 mg, 85%). ^{1}H-NMR (400 MHz, $CDCl_3$) δ 9.73 (s, 1H), 7.50 (d, *J* = 8.1 Hz, 1H), 7.48 – 7.43 (m, 2H), 7.38 – 7.30 (m, 2H), 7.26 (t, *J* = 7.4 Hz, 1H), 7.17 (ddd, *J* = 8.0, 5.3, 2.5 Hz, 1H), 4.18 (s, 3H), 2.37 (s, 3H). ^{13}C-NMR (100 MHz, $CDCl_3$) δ 183.8, 140.1, 139.8, 132.2, 131.4, 130.4, 129.1, 129.0, 127.3, 126.2, 124.8, 124.4, 122.4, 121.0, 110.5, 31.9, 15.6. HRMS (ESI) calcd. for $C_{17}H_{15}ONNaS$ (M + Na^+) 304.0767. Found 304.0766.

***N*-allylation reactions for the synthesis of substrates 1f and 1s**

Over a solution of 3-aryl-1-*H*-indole-2-carbaldehyde derivative (0.40 mmol) and DMSO (0.80 mL) in a Schlenk tube, were added, under air, KOH (0.80 mmol, 45 mg) and allyl chloride (0.80 mmol, 65 µL), and reaction mixture was stirred at room temperature for 2 h. The reaction was then quenched by addition of water and extracted with EtOAc (x3). The combined organic phases were dried over $MgSO_4$, concentrated, and the residue was purified by silica gel column chromatography using cyclohexane/CH_2Cl_2 (2:1) as eluent.

1-Allyl-3-(2-(methylthio)phenyl)-1*H*-indole-2-carbaldehyde (1f)

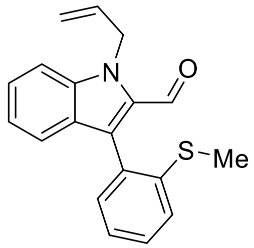

Following the procedure described above from **8a**, purification by column chromatography afforded **1f** as a pale-yellow solid (123 mg, 99%). ^{1}H-NMR (400 MHz, $CDCl_3$) δ 9.69 (s, 1H), 7.49 (d, *J* = 8.1 Hz, 1H), 7.49 – 7.39 (m, 3H), 7.38 – 7.29 (m, 2H), 7.26 (dd, *J* = 7.6, 1.0 Hz, 1H), 7.16 (ddd, *J* = 8.0, 4.7, 3.1 Hz, 1H), 6.06 (ddt, *J* = 16.9, 10.1, 5.0 Hz, 1H), 5.34 – 5.28 (m, 2H), 5.15 (dd, *J* = 10.3, 1.1 Hz, 1H), 4.99 (dd, *J* = 17.1, 1.1 Hz, 1H), 2.35 (s, 3H). ^{13}C-NMR (100 MHz, $CDCl_3$) δ 183.5, 140.2, 139.3, 133.7, 132.1, 130.9, 130.31, 129.6, 129.1, 127.4, 126.4, 124.8, 124.4, 122.5, 121.2, 116.4, 110.2, 47.1, 15.6.

1-Allyl-3-(2-(ethylthio)phenyl)-1*H*-indole-2-carbaldehyde (1s)

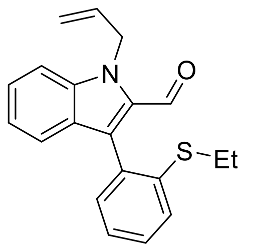

Following the procedure described above from **8m**, purification by column chromatography afforded **1s** as a pale-yellow solid (127 mg, 99%). ^{1}H-NMR (400 MHz, $CDCl_3$) δ 9.69 (s, 1H), 7.49 (d, *J* = 8.1 Hz, 1H), 7.46 – 7.38 (m, 4H), 7.36 (d, *J* = 7.5 Hz, 1H), 7.30 – 7.23 (m, 1H), 7.16 (ddd, *J* = 8.0, 4.8, 3.0 Hz, 1H), 6.06 (ddt, *J* = 15.3, 10.1, 4.9 Hz, 1H), 5.40 – 5.23 (m, 2H), 5.15 (d, *J* = 10.9 Hz, 1H), 4.99 (d, *J* = 17.1 Hz, 1H), 2.80 (q, *J* = 7.3 Hz, 2H), 1.21 (t, *J* = 7.4 Hz, 3H). ^{13}C-NMR (100 MHz, $CDCl_3$) δ 183.5, 139.2, 138.6, 133.7, 132.3, 131.4, 130.7, 129.8, 128.8, 127.3, 127.0, 126.5, 124.9, 122.4, 121.1, 116.3, 110.8, 47.0, 26.8, 13.8. HRMS (ESI) calcd. for $C_{20}H_{19}ONNaS$ (M + Na^+) 344.1080. Found 344.1080.

4. General procedure for the catalytic asymmetric reductive aldol reaction

Over a flame dried Schlenk, under inert atmosphere, containing aldehyde **1a-z** (0.10 mmol) and [Rh((*R*,*R*)-Phebox)(OAc)$_2$(H_2O)] (0.006 mmol, 3.2 mg) in dry toluene (0.30 mL), *tert*-butyl acrylate (0.15 mmol, 23 µL) and 1,1,1,3,5,5,5-heptamethyltrisiloxane (0.16 mmol, 44 µL) were added, and the reaction mixture was stirred at 50 ºC until complete conversion of the starting material (typically 30 min as monitored by TLC). The reaction was then allowed to cool to room temperature and 2 mL of THF were added, followed by cooling to -20 ºC. TBAF (0.2 mL, 1.0 M in THF) was then added and the reaction was allowed to stir at this temperature for 15 min. Brine was then added and the resulting mixture was extracted with ethyl acetate (x3). The combined organic phases were dried over $MgSO_4$, concentrated and the residue was purified by silica gel column chromatography, tipically using cyclohexane/EtOAc (12:1) as eluent.

*<u>Note 1</u>. Racemic products were obtained following the method described by Ando et al.[3] Over a solution of RhCl(PPh_3)$_3$ (3.0 mg, 5 mol%) in THF (0.3 mL), at rt under inert atmosphere, tert-butyl acrylate (16 µL, 0.1 mmol), aldehyde **1a-z** (0.1 mmol) and Et_2Zn (0.12 mmol, 120 µL, 1.0 M in hexane) were added and the reaction mixture was stirred for 2 h. The mixture was then quenched by addition of 10% HCl and extracted with AcOEt. The organic layer was dried over $MgSO_4$, and concentrated under reduced pressure, affording the racemic mixture of reductive aldol products that was isolated from the reaction crude by preparative TLC prior to HPLC analysis.*

*<u>Note 2</u>. The reaction between **1m** and tert-butyl acrylate at 1.2 mmol scale was performed following the general procedure using 1.2 mmol **1m**, 1.5 equiv of tert-butyl acrylate, 2 mol% of [Rh((R,R)-Phebox)(OAc)$_2$(H_2O)] and 1.6 equiv of 1,1,1,3,5,5,5-heptamethyltrisiloxane in 3.6 mL of toluene, affording **2m** (596 mg, 99%) after column chromatography purification. In this case, the reaction requires 40 min for completion.*

tert-Butyl (2*S*,3*R*,*S*$_a$)-3-(1-benzyl-3-(2-(methylthio)phenyl)-1*H*-indol-2-yl)-3-hydroxy-2-methylpropanoate (2a)

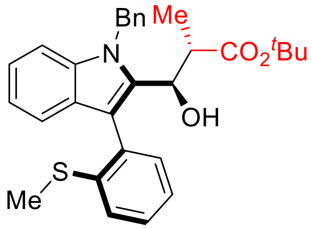

Following the general procedure from **1a** (0.10 mmol, 36 mg), purification by column chromatography afforded **2a** as a pale-yellow foam (47 mg, 97%). d.r. 9:1. Major diastereomer: 99% *ee*. ^{1}H-NMR (400 MHz, $CDCl_3$) δ 7.47 – 7.05 (m, 13H), 5.86 (d, *J* = 17.1 Hz, 1H), 5.70 (d, *J* = 17.0 Hz, 1H), 4.98 (d, *J* = 11.0 Hz, 1H), 3.09 (s, 1H), 2.76 (dq, *J* = 11.0, 7.0 Hz, 1H), 2.36 (s, 3H), 1.41 (s, 9H), 0.70 (d, *J* = 6.9 Hz, 3H). ^{13}C-NMR (100 MHz, $CDCl_3$) δ 174.7, 139.4, 138.4, 138.1, 135.3, 132.6, 132.3, 128.7, 128.2, 127.4, 127.1, 126.0, 124.6, 124.5, 122.8, 120.2, 119.7, 115.3, 110.4, 80.8, 70.3, 48.4, 45.6, 28.1, 27.0, 15.3, 14.7. HRMS (ESI) calcd. for $C_{30}H_{33}O_3NNaS$ (M + Na$^+$) 510.2073. Found 510.2066. HPLC (IA column, 95:5 n-Hex/iPrOH, 30 °C, 1.0 mL/min): t_R 13.33 min (major) and 27.55 min (minor).

tert-Butyl (2*S*,3*R*,*S*$_a$)-3-(1-(4-bromobenzyl)-3-(2-(methylthio)phenyl)-1*H*-indol-2-yl)-3-hydroxy-2-methylpropanoate (2b)

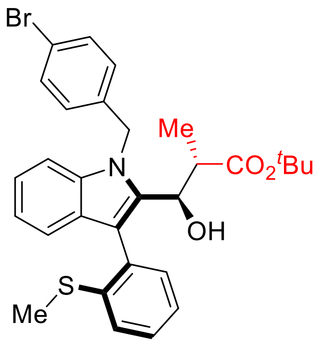

Following the general procedure from **1b** (0.10 mmol, 44 mg), purification by column chromatography afforded **2b** as a pale-yellow oil (57 mg, 99%). d.r. 7:1. Major diastereomer: $[\alpha]^{20}_{D}$ +26.5 (*c* 0.53, $CHCl_3$) for 87% *ee*. ^{1}H-NMR (400 MHz, $CDCl_3$) δ 7.46 – 7.06 (m, 9H), 6.96 (d, *J* = 8.4 Hz, 2H), 5.78 (d, *J* = 17.2 Hz, 1H), 5.64 (d, *J* = 17.2 Hz, 1H), 4.97 (d, *J* = 10.9 Hz, 1H), 3.10 (br, 1H), 2.72 (dq, *J* = 10.9, 7.0 Hz, 1H), 2.36 (s, 3H), 1.42 (s, 9H), 0.71 (d, *J* = 7.0 Hz, 3H). ^{13}C-NMR (100 MHz, $CDCl_3$) δ 174.5, 139.2, 137.8, 137.4, 135.1, 132.2, 132.2, 131.8, 128.2, 127.7, 127.4, 124.6, 124.4, 122.8, 120.9, 120.3, 119.7, 115.4, 110.1, 80.9, 70.2, 47.8, 45.7, 28.0, 15.2, 14.6. HRMS (ESI) calcd. for $C_{30}H_{32}O_3NBrNaS$ (M + Na$^+$) 588.1178. Found 588.1173. HPLC (IA column, 95:5 n-Hex/iPrOH, 30 °C, 1.0 mL/min): t_R 9.94 min (minor) and 15.95 min (major).

tert-Butyl (2*S*,3*R*,*S*$_a$)-3-hydroxy-2-methyl-3-(3-(2-(methylthio)phenyl)-1-(4-(trifluoromethyl)benzyl)-1*H*-indol-2-yl)propanoate (2c)

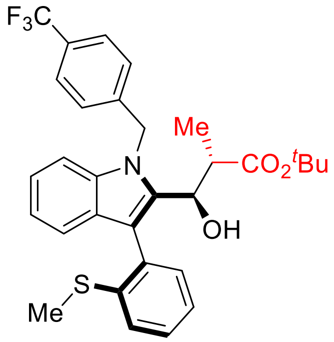

Following the general procedure from **1c** (0.10 mmol, 43 mg), purification by column chromatography afforded **2c** as a pale-yellow oil (50 mg, 90%). d.r. 5:1. Major diastereomer: $[\alpha]^{20}_{D}$ +28.6 (*c* 0.35, $CHCl_3$) for 94% *ee*. ^{1}H-NMR (400 MHz, $CDCl_3$) δ 7.55 (d, *J* = 8.0 Hz, 2H), 7.43 – 7.06 (m, 10H), 5.86 (d, *J* = 17.4 Hz, 1H), 5.76 (d, *J* = 17.4 Hz, 1H), 4.98 (dd, *J* = 10.9, 3.2 Hz, 1H), 3.09 (d, *J* = 3.3 Hz, 1H), 2.71 (dq, *J* = 14.1, 7.0 Hz, 1H), 2.37 (s, 3H), 1.41 (s, 9H), 0.72 (d, *J* = 7.0 Hz, 3H). ^{13}C-NMR (100 MHz, $CDCl_3$) δ 174.4, 142.5, 139.2, 137.8, 137.7, 135.1, 132.1, 129.3 (q, *J* = 32 Hz), 128.2, 127.4, 126.2, 125.7, 125.6, 124.6, 124.4, 122.9, 121.6 (q, *J* = 279 Hz), 120.4, 119.8, 115.5, 110.0, 80.9, 70.2, 48.0, 45.8, 28.0, 15.2, 14.6. ^{19}F NMR (376 MHz, $CDCl_3$) δ -62.4. HRMS (ESI) calcd. for $C_{31}H_{32}O_3NF_3NaS$ (M + Na$^+$) 578.1947. Found 578.1941. HPLC (IA column, 95:5 n-Hex/iPrOH, 30 °C, 1.0 mL/min): t_R 6.78 min (minor) and 10.62 min (major).

***tert*-Butyl (2*S*,3*R*,*S*$_a$)-3-hydroxy-2-methyl-3-(3-(2-(methylthio)phenyl)-1-(4-(trifluoromethoxy)benzyl)-1*H*-indol-2-yl)propanoate (2d)**

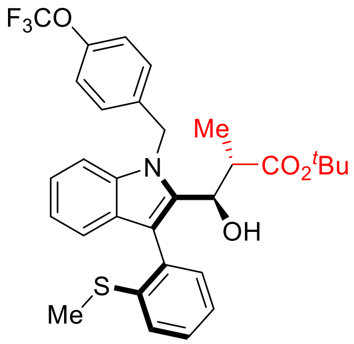

Following the general procedure from **1d** (0.10 mmol, 44 mg), purification by column chromatography afforded **2d** as a pale-yellow oil (47 mg, 83%). d.r. 7:1. Major diastereomer: $[\alpha]^{20}_{D}$ +35.6 (*c* 0.72, $CHCl_3$) for 95% *ee*. ^{1}H-NMR (400 MHz, $CDCl_3$) δ 7.43 – 7.22 (m, 6H), 7.19 – 7.09 (m, 6H), 5.80 (d, *J* = 17.1 Hz, 1H), 5.71 (d, *J* = 16.9 Hz, 1H), 4.97 (dd, *J* = 10.9, 3.5 Hz, 1H), 3.10 (d, *J* = 3.5 Hz, 1H), 2.71 (dq, *J* = 10.9, 7.0 Hz, 1H), 2.37 (s, 3H), 1.41 (s, 9H), 0.70 (d, *J* = 7.1 Hz, 3H). ^{13}C-NMR (100 MHz, $CDCl_3$) δ 174.4, 148.2, 139.2, 137.8, 137.1, 135.1, 132.2, 132.1, 128.2, 127.3, 124.5, 124.3, 122.8, 121.2, 120.3, 119.7, 120.3, 119.7, 115.4, 110.1, 80.8, 70.1, 47.7, 45.7, 28.0, 15.2, 14.6. CF_3 and CF_3O-*C*$_{ipso}$ are missing. ^{19}F NMR (376 MHz, $CDCl_3$) δ -57.9. HRMS (ESI) calcd. for $C_{31}H_{32}O_4NF_3NaS$ (M + Na$^+$) 594.1896. Found 594.1898. HPLC (IA column, 95:5 n-Hex/iPrOH, 30 °C, 1.0 mL/min): t_R 8.21 min (minor) and 14.46 min (major).

***tert*-Butyl (2*S*,3*R*,*S*$_a$)-3-(1-methyl-3-(2-(methylthio)phenyl)-1*H*-indol-2-yl)-3-hydroxy-2-methylpropanoate (2e)**

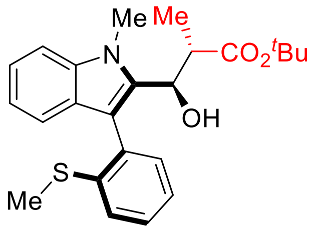

Following the general procedure from **1e** (0.10 mmol, 28 mg), purification by column chromatography afforded **2e** as a pale-yellow oil (41 mg, 99%). d.r. 4:1. Major diastereomer: $[\alpha]^{20}_{D}$ +77.2 (*c* 0.53, $CHCl_3$) for 90% *ee*. ^{1}H-NMR (400 MHz, $CDCl_3$) δ 7.40 – 7.19 (m, 7H), 7.09 (t, *J* = 7.4 Hz, 1H), 4.97 (d, *J* = 10.8 Hz, 1H), 4.02 (s, 3H), 3.01 (dq, *J* = 10.8, 7.1 Hz, 1H), 2.33 (s, 3H), 1.47 (s, 9H), 0.76 (d, *J* = 7.1 Hz, 3H). ^{13}C-NMR (100 MHz, $CDCl_3$) δ 174.7, 139.23 138.23 135.12 132.6, 132.3, 128.1, 127.0, 124.6, 124.4, 122.5, 119.9, 119.6, 114.8, 109.2, 80.9, 70.1, 45.6, 31.6, 28.2, 15.3, 14.7. HRMS (ESI) calcd. for $C_{24}H_{29}O_3NNaS$ (M + Na$^+$) 434.1760. Found 434.1756. HPLC (IC column, 99:1 n-Hex/iPrOH, 30 °C, 1.0 mL/min): t_R 7.96 min (minor) and 10.32 min (major).

***tert*-Butyl (2*S*,3*R*,*S*$_a$)-3-(1-allyl-3-(2-(methylthio)phenyl)-1*H*-indol-2-yl)-3-hydroxy-2-methylpropanoate (2f)**

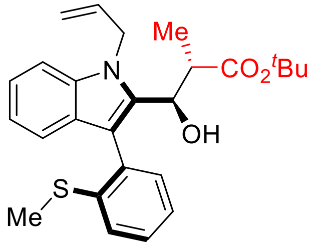

Following the general procedure from **1f** (0.10 mmol, 31 mg), purification by column chromatography afforded **2f** as a pale-yellow oil (44 mg, 99%). d.r. 6:1. Major diastereomer: $[\alpha]^{20}_{D}$ +58.7 (*c* 0.50, $CHCl_3$) for 94% *ee*. ^{1}H-NMR (400 MHz, $CDCl_3$) δ 7.38 (ddd, *J* = 8.1, 7.2, 1.6 Hz, 1H), 7.34 – 7.19 (m, 6H), 7.09 (ddd, *J* = 8.0, 7.0, 1.0 Hz, 1H), 6.08 (ddt, *J* = 17.0, 10.5, 4.6 Hz, 1H), 5.34 (ddt, *J* = 17.6, 4.8, 1.9 Hz, 1H), 5.22 (dd, *J* = 10.5, 1.3 Hz, 1H), 5.08 – 4.93 (m, 3H), 2.94 (dq, *J* = 10.9, 7.1 Hz, 1H), 2.33 (s, 3H), 1.46 (s, 9H), 0.76 (d, *J* = 7.1 Hz, 3H). ^{13}C-NMR (100 MHz, $CDCl_3$) δ

174.8, 139.3, 137.9, 134.9, 134.0, 132.6, 132.3, 128.2, 127.3, 124.6, 124.4, 122.6, 120.1, 119.6, 116.2, 115.0, 110.2, 80.8, 70.2, 47.1, 45.5, 28.2, 15.3, 14.8. HRMS (ESI) calcd. for $C_{26}H_{31}O_3NNaS$ (M + Na^+) 460.1917. Found 460.1914. HPLC (IA column, 95:5 n-Hex/iPrOH, 30 °C, 1.0 mL/min): t_R 7.80 min (minor) and 13.12 min (major).

***tert*-Butyl (2*S*,3*R*,*S*$_a$)-3-(1-benzyl-6-methoxy-3-(2-(methylthio)phenyl)-1*H*-indol-2-yl)-3-hydroxy-2-methylpropanoate (2g)**

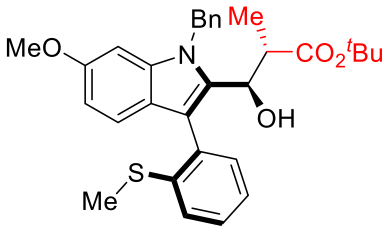

Following the general procedure from **1g** (0.10 mmol, 39 mg), purification by column chromatography afforded **2g** as a pale-yellow foam (48 mg, 93%). d.r. 4:1. Major diastereomer: $[\alpha]^{20}_D$ +24.2 (*c* 0.52, $CHCl_3$) for 96% *ee*. ^{1}H-NMR (400 MHz, $CDCl_3$) δ 7.38 (t, *J* = 7.5 Hz, 1H), 7.27 (dt, *J* = 38.0, 7.7 Hz, 7H), 7.12 (d, *J* = 7.2 Hz, 2H), 6.75 (dd, *J* = 8.6, 2.1 Hz, 1H), 6.61 (d, *J* = 1.9 Hz, 1H), 5.80 (d, *J* = 17.2 Hz, 1H), 5.62 (d, *J* = 17.2 Hz, 1H), 4.91 (d, *J* = 11.0 Hz, 1H), 3.73 (s, 3H), 3.05 (br, 1H), 2.72 (dq, *J* = 11.1, 7.0 Hz, 1H), 2.35 (s, 3H), 1.40 (s, 9H), 0.69 (d, *J* = 7.0 Hz, 3H). ^{13}C-NMR (100 MHz, $CDCl_3$) δ 174.7, 157.0, 139.3, 138.8, 138.1, 134.0, 132.5, 132.1, 128.7, 128.1, 127.1, 126.0, 124.5, 124.3, 121.6, 120.2, 115.2, 109.8, 94.1, 80.6, 70.3, 55.7, 48.3, 45.6, 28.0, 15.2, 14.6. HRMS (ESI) calcd. for $C_{31}H_{35}O_4NNaS$ (M + Na^+) 540.2179. Found 540.2170. HPLC (IA column, 95:5 n-Hex/iPrOH, 30 °C, 1.0 mL/min): t_R 9.12 min (minor) and 17.60 min (major).

***tert*-Butyl (2*S*,3*R*,*S*$_a$)-3-(1-benzyl-5-methoxy-3-(2-(methylthio)phenyl)-1*H*-indol-2-yl)-3-hydroxy-2-methylpropanoate (2h)**

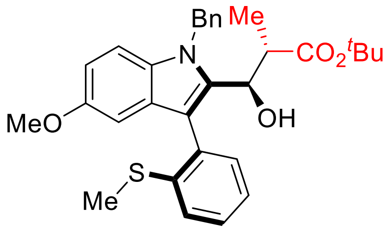

Following the general procedure from **1h** (0.10 mmol, 39 mg), purification by column chromatography afforded **2h** as a pale-yellow foam (47 mg, 90%). d.r. 3:1. Major diastereomer: $[\alpha]^{20}_D$ +19.4 (*c* 0.56, $CHCl_3$) for 94% *ee*. ^{1}H-NMR (400 MHz, $CDCl_3$) δ 7.40 (t, *J* = 7.5 Hz, 1H), 7.35 – 7.22 (m, 6H), 7.11 – 7.07 (m, 2H), 7.02 (d, *J* = 8.9 Hz, 1H), 6.80 (dd, *J* = 8.8, 2.4 Hz, 1H), 6.76 (d, *J* = 2.2 Hz, 1H), 5.80 (d, *J* = 17.1 Hz, 1H), 5.66 (d, *J* = 17.1 Hz, 1H), 4.93 (d, *J* = 10.9 Hz, 1H), 3.75 (s, 3H), 3.05 (br, 1H), 2.74 (dq, *J* = 11.1, 7.0 Hz, 1H), 2.37 (s, 3H), 1.40 (s, 9H), 0.70 (d, *J* = 7.0 Hz, 3H). ^{13}C-NMR (100 MHz, $CDCl_3$) δ 174.7, 154.7, 139.5, 138.5, 135.9, 133.2, 132.7, 132.3, 128.8, 128.2, 127.1, 126.2, 126.0, 124.7, 124.4, 114.9, 113.1, 111.3, 101.0, 80.8, 70.3, 55.9, 48.5, 45.7, 28.1, 15.3, 14.7. HRMS (ESI) calcd. for $C_{31}H_{35}O_4NNaS$ (M + Na^+) 540.2179. Found 540.2171. HPLC (IA column, 95:5 n-Hex/iPrOH, 30 °C, 1.0 mL/min): t_R 9.81 min (minor) and 13.80 min (major).

tert-Butyl (2*S*,3*R*,*S*$_a$)-3-(1-benzyl-5-(benzyloxy)-3-(2-(methylthio)phenyl)-1*H*-indol-2-yl)-3-hydroxy-2-methylpropanoate (2i)

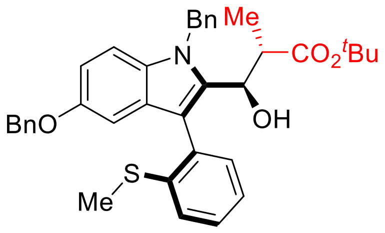

Following the general procedure from **1i** (0.10 mmol, 46 mg), purification by column chromatography afforded **2i** as a pale-yellow foam (50 mg, 84%). d.r. > 20:1. Major diastereomer: $[\alpha]^{20}_{D}$ +12.7 (*c* 0.55, $CHCl_3$) for 96% *ee*. ^{1}H-NMR (400 MHz, $CDCl_3$) 7.45 – 7.27 (m, 12H), 7.11 (d, *J* = 7.0 Hz, 2H), 7.08 – 7.03 (m, 1H), 6.93 – 6.88 (m, 2H), 5.82 (d, *J* = 17.1 Hz, 1H), 5.68 (d, *J* = 17.1 Hz, 1H), 5.01 – 4.94 (m, 3H), 3.10 (d, *J* = 3.3 Hz, 1H), 2.76 (dq, *J* = 10.9, 7.0 Hz, 1H), 2.38 (s, 3H), 1.42 (s, 9H), 0.72 (d, *J* = 7.1 Hz, 3H). δ ^{13}C-NMR (100 MHz, $CDCl_3$) δ 174.7, 154.0, 139.5, 138.4, 137.7, 136.0, 133.4, 132.6, 132.3, 128.7, 128.5, 128.2, 127.8, 127.8, 127.1, 126.0, 124.7, 124.5, 114.9, 113.5, 111.3, 102.5, 80.8, 70.8, 70.3, 48.5, 45.7, 28.1, 15.3, 14.7. HRMS (ESI) calcd. for $C_{37}H_{39}O_4NNaS$ (M + Na^+) 616.2492. Found 616.2490. HPLC (IA column, 95:5 n-Hex/iPrOH, 30 °C, 1.0 mL/min): t_R 15.30 min (minor) and 25.69 min (major).

tert-Butyl (2*S*,3*R*,*S*$_a$)-3-(1-benzyl-5-fluoro-3-(2-(methylthio)phenyl)-1*H*-indol-2-yl)-3-hydroxy-2-methylpropanoate (2j)

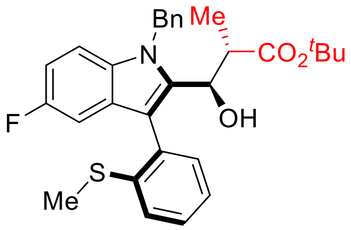

Following the general procedure from **1j** (0.10 mmol, 38 mg), purification by column chromatography afforded **2j** as a white foam (49 mg, 97%). d.r. 6:1. Major diastereomer: $[\alpha]^{20}_{D}$ +43.2 (*c* 0.50, $CHCl_3$) for 94% *ee*. ^{1}H-NMR (400 MHz, $CDCl_3$) δ 7.39 (ddd, *J* = 8.5, 7.2, 1.8 Hz, 1H), 7.34 – 7.21 (m, 6H), 7.07 (d, *J* = 7.2 Hz, 2H), 7.03 (dd, *J* = 8.9, 4.3 Hz, 1H), 6.96 (dd, *J* = 9.3, 2.5 Hz, 1H), 6.87 (td, *J* = 9.0, 2.5 Hz, 1H), 5.82 (d, *J* = 17.1 Hz, 1H), 5.67 (d, *J* = 17.1 Hz, 1H), 4.95 (d, *J* = 11.0 Hz, 1H), 2.73 (dq, *J* = 10.9, 7.0 Hz, 1H), 2.37 (s, 3H), 1.40 (s, 9H), 0.70 (d, *J* = 7.1 Hz, 3H). ^{13}C-NMR (100 MHz, $CDCl_3$) δ 174.6, 158.4 (d, *J* = 236 Hz), 139.4, 138.1, 137.1, 134.5, 132.2, 132.0, 128.8, 128.4, 127.7 (d, *J* = 10 Hz), 127.3, 126.0, 124.7, 124.5, 115.2 (d, *J* = 5 Hz), 111.2 (d, *J* = 10 Hz), 111.0, 104.5 (d, *J* = 24 Hz), 80.9, 70.3, 48.6, 45.6, 28.1, 15.3, 14.7. ^{19}F-NMR (376 MHz, $CDCl_3$) δ -124.07. HRMS (ESI) calcd. for $C_{30}H_{32}O_3NFNaS$ (M + Na^+) 528.1979. Found 528.1968. HPLC (IA column, 95:5 n-Hex/iPrOH, 30 °C, 1.0 mL/min): t_R 8.84 min (minor) and 12.91 min (major).

tert-Butyl (2*S*,3*R*,*S*$_a$)-3-(1-benzyl-5-chloro-3-(2-(methylthio) phenyl)-1*H*-indol-2-yl)-3-hydroxy-2-methylpropanoate (2k)

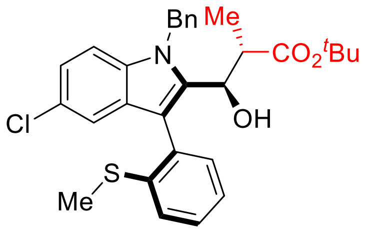

Following the general procedure from **1k** (0.10 mmol, 39 mg), purification by column chromatography afforded **2k** as a pale-yellow foam (45 mg, 86%). d.r. 5:1. Major diastereomer: $[\alpha]^{20}_{D}$ +21.1 (*c* 0.52, $CHCl_3$) for 94% *ee*. ^{1}H-NMR (400 MHz, $CDCl_3$) δ 7.43 – 7.37 (m, 1H), 7.34 – 7.22 (m, 7H), 7.06 (d, *J* = 9.0 Hz, 4H), 5.82 (d, *J* = 17.1 Hz, 1H), 5.68 (d, *J* = 17.1 Hz, 1H), 4.95 (d, *J* =

10.9 Hz, 1H), 2.98 – 2.67 (m, 2H), 2.37 (s, 3H), 1.40 (s, 9H), 0.70 (d, *J* = 7.1 Hz, 3H). ^{13}C-NMR (100 MHz, CDCl$_3$) δ 174.5, 139.4, 137.9, 136.8, 136.4, 132.2, 131.8, 128.9, 128.5, 128.4, 127.3, 126.1, 126.0, 124.8, 124.5, 123.1, 119.1, 114.8, 111.6, 81.0, 70.2, 48.6, 45.6, 28.1, 15.3, 14.7. HRMS (ESI) calcd. for $C_{30}H_{32}O_3NClNaS$ (M + Na$^+$) 544.1684. Found 544.1687. HPLC (ID column, 98:2 n-Hex/iPrOH, 30 °C, 1.0 mL/min): t_R 9.53 min (minor) and 11.32 min (major).

***tert*-Butyl (2*S*,3*R*,*S*$_a$)-3-(1-benzyl-5-bromo-3-(2-(methylthio)phenyl)-1*H*-indol-2-yl)-3-hydroxy-2-methylpropanoate (2l)**

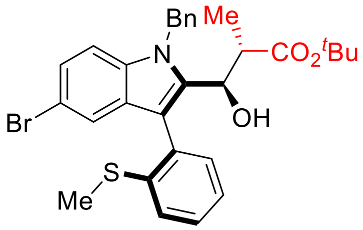

Following the general procedure from **1l** (0.10 mmol, 44 mg), purification by column chromatography afforded **2l** as a pale-yellow oil (50 mg, 88%). d.r. 5:1. Major diastereomer: $[\alpha]^{20}_D$ +18.5 (*c* 0.49, CHCl$_3$) for 94% *ee*. ^{1}H-NMR (400 MHz, CDCl$_3$) δ 7.44 (d, *J* = 1.9 Hz, 1H), 7.40 (ddd, *J* = 7.9, 7.1, 1.8 Hz, 1H), 7.33 – 7.26 (m, 5H), 7.21 (dd, *J* = 8.7, 1.9 Hz, 1H), 7.12 – 7.04 (m, 3H), 7.00 (d, *J* = 8.7 Hz, 1H), 5.81 (d, *J* = 17.1 Hz, 1H), 5.67 (d, *J* = 17.1 Hz, 1H), 4.95 (d, *J* = 10.9 Hz, 1H), 2.72 (dq, *J* = 10.9, 7.1 Hz, 1H), 2.37 (s, 3H), 1.40 (s, 9H), 0.69 (d, *J* = 7.1 Hz, 3H). ^{13}C-NMR (100 MHz, CDCl$_3$) δ 174.5, 139.4, 137.9, 136.7, 132.2, 131.7, 129.0, 128.9, 128.8, 128.5, 127.3, 125.9, 125.6, 124.7, 124.5, 122.2, 114.7, 113.6, 112.0, 81.0, 70.2, 48.5, 45.6, 28.1, 15.3, 14.7. HRMS (ESI) calcd. for $C_{30}H_{32}O_3NBrNaS$ (M + Na$^+$) 588.1178. Found 588.1177. HPLC (IA column, 95:5 n-Hex/iPrOH, 30 °C, 1.0 mL/min): t_R 14.66 min (minor) and 18.03 min (major).

***tert*-Butyl (2*S*,3*R*,*S*$_a$)-3-(1-benzyl-3-(2-(ethylthio)phenyl)-1*H*-indol-2-yl)-3-hydroxy-2-methylpropanoate (2m)**

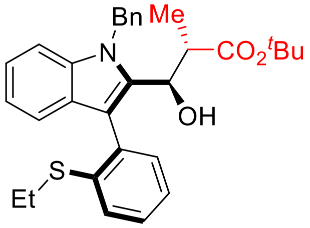

Following the general procedure from **1m** (0.10 mmol, 37 mg), purification by column chromatography afforded **2m** as a white foam (45 mg, 90%). The pure product was crystallized by slow diffusion of *n*-hexane into a solution of the product in acetone to give **2m** as white crystals suitable for X-Ray analysis. d.r. > 20:1. Major diastereomer: $[\alpha]^{20}_D$ +37.0 (*c* 0.48, CHCl$_3$) for 96% *ee*. ^{1}H-NMR (400 MHz, CDCl$_3$) δ 7.41 – 7.33 (m, 4H), 7.32 – 7.21 (m, 4H), 7.15 (d, *J* = 3.4 Hz, 2H), 7.12 – 7.05 (m, 3H), 5.85 (d, *J* = 17.2 Hz, 1H), 5.69 (d, *J* = 17.2 Hz, 1H), 4.98 (dd, *J* = 11.0, 3.3 Hz, 1H), 3.11 (d, *J* = 3.4 Hz, 1H), 2.89 – 2.69 (m, 3H), 1.40 (s, 9H), 1.24 (t, *J* = 7.4 Hz, 3H), 0.67 (d, *J* = 7.0 Hz, 3H). ^{13}C-NMR (100 MHz, CDCl$_3$) δ 174.6, 138.4, 138.1, 137.8, 135.2, 133.7, 132.6, 128.7, 128.0, 127.5, 127.1, 126.9, 126.0, 125.3, 122.7, 120.2, 119.6, 115.5, 110.4, 80.7, 70.4, 48.4, 45.7, 28.1, 26.3, 14.7, 13.8. HRMS (ESI) calcd. for $C_{31}H_{35}O_3NNaS$ (M + Na$^+$) 524.2230. Found 524.2222. HPLC (IA column, 95:5 n-Hex/iPrOH, 30 °C, 1.0 mL/min): t_R 7.29 min (minor) and 9.66 min (major).

***tert*-Butyl (2*S*,3*R*,*S*$_a$)-(1-benzyl-3-(2-(ethylthio)phenyl)-1*H*-indol-2-yl)(hydroxy)methyl)butanoate (2n)**

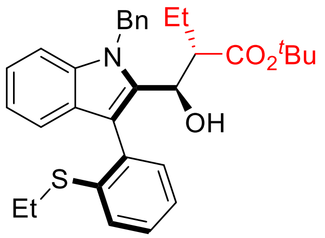

Following the general procedure from **1m** (0.10 mmol, 37 mg) and *tert*-butyl crotonate (0.15 mmol, 25 μL), purification by column chromatography afforded **2n** as a pale-yellow oil (40 mg, 78%). d.r. 10:1. Major diastereomer: $[\alpha]^{20}_D$ +44.6 (*c* 0.53, $CHCl_3$) for 94% *ee*. ^{1}H-NMR (400 MHz, $CDCl_3$) δ 7.42 – 7.22 (m, 8H), 7.18 (d, *J* = 6.0 Hz, 2H), 7.10 (t, *J* = 6.8 Hz, 3H), 5.90 (d, *J* = 17.3 Hz, 1H), 5.67 (d, *J* = 17.2 Hz, 1H), 4.96 (dd, *J* = 11.2, 3.2 Hz, 1H), 3.03 (d, *J* = 3.4 Hz, 1H), 2.89 – 2.68 (m, 2H), 2.58 (td, *J* = 11.0, 3.8 Hz, 1H), 1.41 (s, 9H), 1.23 (t, *J* = 7.4 Hz, 3H), 1.07 (dp, *J* = 10.8, 7.1 Hz, 1H), 0.94 (ddq, *J* = 14.7, 7.2, 3.6 Hz, 1H), 0.42 (t, *J* = 7.4 Hz, 3H). ^{13}C-NMR (100 MHz, $CDCl_3$) δ 173.8, 138.4, 138.2, 137.6, 135.5, 133.7, 132.6, 128.8, 128.0, 127.4, 127.2, 126.9, 126.0, 125.4, 122.8, 120.2, 119.6, 115.4, 110.3, 80.6, 69.7, 53.1, 48.2, 28.2, 26.3, 22.8, 13.8, 11.1. HRMS (ESI) calcd. for $C_{32}H_{37}O_3NNaS$ (M + Na$^+$) 538.2386. Found 538.2378. HPLC (IA column, 95:5 n-Hex/iPrOH, 30 °C, 1.0 mL/min): t_R 5.38 min (minor) and 6.86 min (major).

***tert*-Butyl (2*S*,3*R*,*S*$_a$)-3-(1-(4-bromobenzyl)-3-(2-(ethylthio)phenyl)-1*H*-indol-2-yl)-3-hydroxy-2-methylpropanoate (2o)**

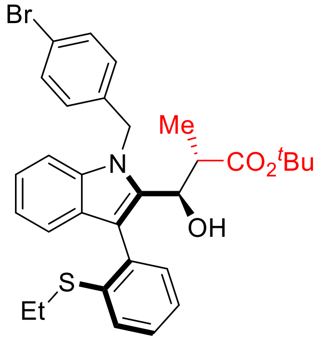

Following the general procedure from **1o** (0.10 mmol, 45 mg), purification by column chromatography afforded **2o** as a pale-yellow foam (52 mg, 90%). d.r. > 20:1. Major diastereomer: $[\alpha]^{20}_D$ +43.7 (*c* 0.51, $CHCl_3$) for 94% *ee*. ^{1}H-NMR (400 MHz, $CDCl_3$) δ 7.44 – 7.31 (m, 6H), 7.29 – 7.23 (m, 1H), 7.19 – 7.14 (m, 1H), 7.12 – 7.07 (m, 2H), 6.96 (d, *J* = 8.4 Hz, 2H), 5.78 (d, *J* = 17.3 Hz, 1H), 5.64 (d, *J* = 17.2 Hz, 1H), 4.98 (dd, *J* = 11.0, 3.4 Hz, 1H), 3.12 (d, *J* = 3.4 Hz, 1H), 2.90 – 2.66 (m, 3H), 1.41 (s, 9H), 1.24 (t, *J* = 7.4 Hz, 3H), 0.69 (d, *J* = 7.0 Hz, 3H). ^{13}C-NMR (100 MHz, $CDCl_3$) δ 174.4, 137.8, 137.7, 137.5, 135.1, 133.5, 132.5, 131.8, 128.1, 127.8, 127.6, 126.8, 125.3, 122.8, 120.9, 120.4, 119.8, 115.7, 110.2, 80.8, 70.3, 47.9, 45.8, 28.1, 26.3, 14.7, 13.8. HRMS (ESI) calcd. for $C_{31}H_{34}O_3NBrNaS$ (M + Na$^+$) 602.1335. Found 602.1331. HPLC (IA column, 95:5 n-Hex/iPrOH, 30 °C, 1.0 mL/min): t_R 7.73 min (minor) and 10.39 min (major).

***tert*-Butyl (2*S*,3*R*,*S*$_a$)-3-(1-(2-bromobenzyl)-3-(2-(ethylthio)phenyl)-1*H*-indol-2-yl)-3-hydroxy-2-methylpropanoate (2p)**

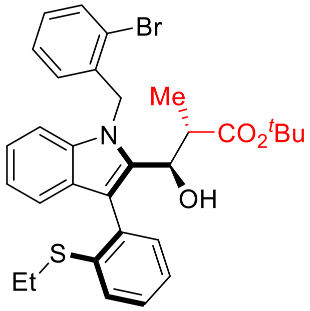

Following the general procedure from **1p** (0.10 mmol, 45 mg), purification by column chromatography afforded **2p** as a pale-yellow foam (53 mg, 91%). d.r. > 20:1. Major diastereomer: $[\alpha]^{20}_D$ +7.4 (*c* 0.55, $CHCl_3$) for 92% *ee*. ^{1}H-NMR (400 MHz, $CDCl_3$) δ 7.66 – 7.59 (m, 1H), 7.37 (q, *J* = 7.7, 6.5 Hz, 4H), 7.26 (dd, *J* = 14.1, 1.6 Hz, 1H), 7.22 – 7.14 (m, 1H), 7.11 (dt, *J* = 6.9, 3.8 Hz, 4H), 6.53 – 6.49 (m, 1H), 5.93 (d, *J* = 17.8 Hz, 1H), 5.61 (d, *J* = 17.8 Hz, 1H), 4.96 (dd, *J* = 10.9, 3.2 Hz, 1H), 3.06 (d, *J* = 3.2 Hz, 1H), 2.91 – 2.66 (m, 3H), 1.42 (s, 9H), 1.24 (t, *J* = 7.3 Hz, 3H), 0.71 (d, *J* = 7.0 Hz, 3H). ^{13}C-NMR (100 MHz, $CDCl_3$) δ 174.5, 137.9, 137.8, 137.3, 135.3, 133.4, 132.8, 132.5, 128.7, 128.1, 127.7, 127.6, 127.4, 126.8, 125.2, 122.9, 121.8, 120.4, 119.7, 115.8, 110.1, 80.8, 70.3, 48.9, 45.6, 28.1, 26.3, 14.6, 13.8. HRMS (ESI) calcd. for $C_{31}H_{34}O_3NBrNaS$ (M + Na^+) 602.1335. Found 602.1336. HPLC (IB column, 98:2 n-Hex/iPrOH, 30 °C, 1.0 mL/min): t_R 5.44 min (minor) and 6.42 min (major).

***tert*-Butyl (2*S*,3*R*,*S*$_a$)-3-(1-(4-(*tert*-butyl)benzyl)-3-(2-(ethylthio)phenyl)-1*H*-indol-2-yl)-3-hydroxy-2-methylpropanoate (2q)**

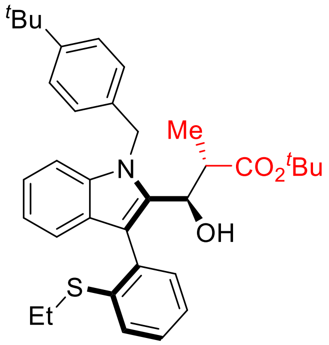

Following the general procedure from **1q** (0.10 mmol, 43 mg), purification by column chromatography afforded **2q** as a white foam (53 mg, 95%). d.r. > 20:1. Major diastereomer: $[\alpha]^{20}_D$ +28.8 (*c* 0.54, $CHCl_3$) for 96% *ee*. ^{1}H-NMR (400 MHz, $CDCl_3$) δ 7.42 – 7.23 (m, 7H), 7.20 – 7.12 (m, 2H), 7.08 (ddd, *J* = 7.9, 6.4, 1.6 Hz, 1H), 7.03 (d, *J* = 8.3 Hz, 2H), 5.77 (d, *J* = 16.9 Hz, 1H), 5.67 (d, *J* = 17.0 Hz, 1H), 4.99 (dd, *J* = 11.0, 3.4 Hz, 1H), 3.09 (d, *J* = 3.4 Hz, 1H), 2.86 – 2.72 (m, 3H), 1.40 (s, 9H), 1.29 (s, 9H), 1.24 (t, *J* = 7.3 Hz, 3H), 0.67 (d, *J* = 7.1 Hz, 3H). ^{13}C-NMR (100 MHz, $CDCl_3$) δ 174.6, 150.0, 138.1, 137.8, 135.3, 135.3, 133.8, 132.7, 128.0, 127.5, 126.9, 125.8, 125.6, 125.3, 122.6, 120.1, 119.6, 115.4, 110.6, 80.7, 70.4, 48.2, 45.8, 34.6, 31.5, 28.1, 26.3, 14.7, 13.9. HRMS (ESI) calcd. for $C_{35}H_{43}O_3NNaS$ (M + Na^+) 580.2856. Found 580.2849. HPLC (IA column, 95:5 n-Hex/iPrOH, 30 °C, 1.0 mL/min): t_R 5.89 min (minor) and 7.84 min (major).

tert-Butyl (2_S_,3_R_,_S_a)-3-(3-(2-(ethylthio)phenyl)-1-(4-methoxybenzyl)-1_H_-indol-2-yl)-3-hydroxy-2-methylpropanoate (2r)

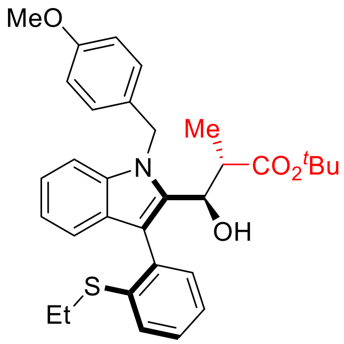

Following the general procedure from **1r** (0.10 mmol, 40 mg), purification by column chromatography afforded **2r** as a pale-yellow foam (42 mg, 79%). d.r. > 20:1. Major diastereomer: $[\alpha]^{20}_{D}$ +29.4 (*c* 0.43, $CHCl_3$) for 95% *ee*. ^{1}H-NMR (400 MHz, $CDCl_3$) δ 7.42 – 7.14 (m, 7H), 7.08 (ddd, *J* = 7.9, 5.7, 2.3 Hz, 1H), 7.03 (d, *J* = 8.5 Hz, 2H), 6.83 (d, *J* = 8.6 Hz, 2H), 5.78 (d, *J* = 16.8 Hz, 1H), 5.64 (d, *J* = 16.8 Hz, 1H), 4.98 (dd, *J* = 11.0, 3.3 Hz, 1H), 3.78 (s, 3H), 3.12 (d, *J* = 3.4 Hz, 1H), 2.92 – 2.64 (m, 3H), 1.41 (s, 9H), 1.24 (t, *J* = 7.4 Hz, 3H), 0.67 (d, *J* = 7.1 Hz, 3H). ^{13}C-NMR (100 MHz, $CDCl_3$) δ 174.5, 158.6, 137.9, 137.7, 135.1, 133.6, 132.5, 130.3, 129.4, 127.9, 127.4, 127.1, 126.8, 125.2, 122.5, 119.5, 115.3, 114.0, 110.4, 80.6, 70.3, 55.3, 47.7, 45.6, 28.0, 26.2, 14.6, 13.7. HRMS (ESI) calcd. for $C_{32}H_{37}O_4NNaS$ (M + Na^+) 554.2336. Found 554.2332. HPLC (IA column, 95:5 n-Hex/iPrOH, 30 °C, 1.0 mL/min): t_R 9.02 min (minor) and 11.74 min (major).

tert-Butyl (2_S_,3_R_,_S_a)-3-(1-allyl-3-(2-(ethylthio)phenyl)-1_H_-indol-2-yl)-3-hydroxy-2-methylpropanoate (2s)

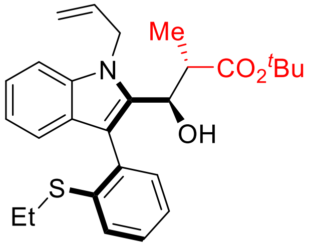

Following the general procedure from **1s** (0.10 mmol, 32 mg), purification by column chromatography afforded **2s** as a pale-yellow foam (43 mg, 95%). d.r. > 20:1. Major diastereomer: $[\alpha]^{20}_{D}$ +61.4 (*c* 0.53, $CHCl_3$) for 93% *ee*. ^{1}H-NMR (400 MHz, $CDCl_3$) δ 7.40 – 7.28 (m, 5H), 7.26 – 7.21 (m, 2H), 7.09 (t, *J* = 7.6 Hz, 1H), 6.08 (ddt, *J* = 15.0, 10.1, 4.5 Hz, 1H), 5.39 – 5.30 (m, 1H), 5.21 (d, *J* = 10.4 Hz, 1H), 5.01 (d, *J* = 17.4 Hz, 1H), 4.99 – 4.92 (m, 2H), 2.94 (dq, *J* = 11.0, 7.0 Hz, 1H), 2.85 – 2.68 (m, 2H), 1.45 (s, 9H), 1.21 (t, *J* = 7.3 Hz, 3H), 0.74 (d, *J* = 7.0 Hz, 3H). ^{13}C-NMR (100 MHz, $CDCl_3$) δ 174.7, 137.8, 137.7, 134.8, 134.0, 133.7, 132.6, 127.9, 127.4, 126.8, 125.2, 122.5, 120.0, 119.6, 116.2, 115.2, 110.2, 80.7, 70.3, 47.1, 45.6, 28.2, 26.3, 14.7, 13.8. HRMS (ESI) calcd. for $C_{27}H_{33}O_3NNaS$ (M + Na^+) 474.2073. Found 474.2069. HPLC (IA column, 95:5 n-Hex/iPrOH, 30 °C, 1.0 mL/min): t_R 5.63 min (minor) and 7.86 min (major).

tert-Butyl (2_S_,3_R_,_S_a)-3-(1-benzyl-3-(2-(ethylthio)phenyl)-6-methoxy-1_H_-indol-2-yl)-3-hydroxy-2-methylpropanoate (2t)

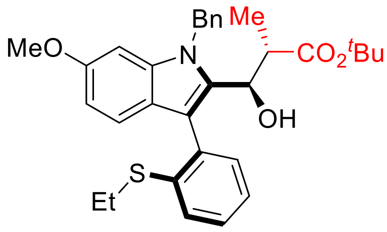

Following the general procedure from **1t** (0.10 mmol, 40 mg), purification by column chromatography afforded **2t** as a pale-yellow foam (53 mg, 99%). d.r. > 20:1. Major diastereomer: $[\alpha]^{20}_{D}$ +20.7 (*c* 0.46, $CHCl_3$) for 96% *ee*. ^{1}H-NMR (400 MHz, $CDCl_3$) δ 7.42 – 7.39 (m, 1H), 7.38 – 7.30 (m, 4H), 7.28 – 7.22 (m, 3H), 7.14 (dd, *J* = 8.1, 1.2 Hz, 2H), 6.77 (dd, *J* = 8.6, 2.2 Hz, 1H), 6.63

(d, J = 2.1 Hz, 1H), 5.82 (d, J = 17.2 Hz, 1H), 5.64 (d, J = 17.2 Hz, 1H), 4.94 (d, J = 11.0 Hz, 1H), 3.75 (s, 3H), 3.10 (s, 1H), 2.90 – 2.70 (m, 3H), 1.42 (s, 9H), 1.26 (t, J = 7.4 Hz, 3H), 0.69 (d, J = 7.0 Hz, 3H). ^{13}C-NMR (100 MHz, $CDCl_3$) δ 174.7, 157.0, 138.9, 138.3, 137.7, 134.0, 133.8, 132.5, 128.8, 128.0, 127.2, 126.8, 126.1, 125.2, 121.9, 120.3, 115.6, 109.9, 94.1, 80.6, 70.4, 55.8, 48.4, 45.7, 28.1, 26.3, 14.7, 13.8. HRMS (ESI) calcd. for $C_{32}H_{37}O_4NNaS$ (M + Na^+) 554.2336. Found 554.2327. HPLC (IA column, 95:5 n-Hex/iPrOH, 30 °C, 1.0 mL/min): t_R 7.90 min (minor) and 12.35 min (major).

tert-Butyl (2*S*,3*R*,*S*$_a$)-3-(1-benzyl-5-(benzyloxy)-3-(2-(ethylthio)phenyl)-1*H*-indol-2-yl)-3-hydroxy-2-methylpropanoate (2u)

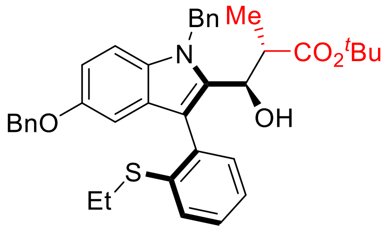

Following the general procedure from **1u** (0.10 mmol, 48 mg), purification by column chromatography afforded **2u** as a pale-yellow foam (45 mg, 74%). d.r. > 20:1. Major diastereomer: $[\alpha]^{20}_D$ -3.6 (*c* 0.40, $CHCl_3$) for 96% *ee*. ^{1}H-NMR (400 MHz, $CDCl_3$) δ 7.45 – 7.22 (m, 12H), 7.09 (d, J = 7.2 Hz, 2H), 7.05 (d, J = 9.5 Hz, 1H), 6.92 – 6.86 (m, 2H), 5.81 (d, J = 17.1 Hz, 1H), 5.67 (d, J = 17.2 Hz, 1H), 4.98 (s, 2H), 4.96 (d, J = 11.3 Hz, 1H), 2.90 – 2.70 (m, 3H), 1.41 (s, 9H), 1.26 (t, J = 7.4 Hz, 3H), 0.69 (d, J = 7.0 Hz, 3H). ^{13}C-NMR (100 MHz, $CDCl_3$) δ 174.5, 153.8, 138.4, 137.8, 137.6, 135.8, 133.6, 133.3, 132.5, 128.6, 128.5, 127.9, 127.8, 127.7, 127.1, 126.7, 125.9, 125.2, 115.1, 113.3, 111.2, 102.5, 80.6, 70.7, 70.3, 48.4, 45.6, 28.0, 26.2, 14.6, 13.7. HRMS (ESI) calcd. for $C_{37}H_{41}O_4NNaS$ (M + Na^+) 630.2649 Found 630.2650. HPLC (IA column, 95:5 n-Hex/iPrOH, 30 °C, 1.0 mL/min): t_R 10.32 min (minor) and 13.64 min (major).

tert-Butyl (2*S*,3*R*,*S*$_a$)-3-(1-benzyl-3-(2-(ethylthio)phenyl)-5-fluoro-1*H*-indol-2-yl)-3-hydroxy-2-methylpropanoate (2v)

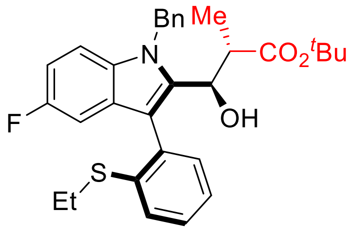

Following the general procedure from **1v** (0.10 mmol, 39 mg), purification by column chromatography afforded **2v** as a pale-yellow foam (43 mg, 83%). d.r. > 20:1. Major diastereomer: $[\alpha]^{20}_D$ +33.5 (*c* 0.39, $CHCl_3$) for 91% *ee*. ^{1}H-NMR (400 MHz, $CDCl_3$) δ 7.37 (t, J = 8.1 Hz, 2H), 7.34 – 7.22 (m, 5H), 7.09 – 7.01 (m, 3H), 6.97 (dd, J = 9.3, 2.3 Hz, 1H), 6.87 (td, J = 9.0, 2.3 Hz, 1H), 5.81 (d, J = 17.1 Hz, 1H), 5.67 (d, J = 17.1 Hz, 1H), 4.95 (d, J = 11.0 Hz, 1H), 2.90 – 2.68 (m, 3H), 1.39 (s, 9H), 1.25 (t, J = 7.3 Hz, 3H), 0.68 (d, J = 7.0 Hz, 3H). ^{13}C-NMR (100 MHz, $CDCl_3$) δ 174.5, 158.4 (d, J = 236 Hz), 138.1, 137.8, 137.0, 134.5, 133.2, 132.5, 128.8, 128.2, 127.9 (d, J = 10 Hz), 127.3, 126.9, 126.0, 125.4, 115.4 (d, J = 5 Hz), 111.2 (d, J = 5 Hz), 110.9, 104.5 (d, J = 24 Hz), 80.8, 70.3, 48.6, 45.7, 28.1, 26.3, 14.7, 13.8. ^{19}F-NMR (376 MHz, $CDCl_3$) δ -124.1. HRMS (ESI) calcd. for $C_{31}H_{34}O_3NFNaS$ (M + Na^+) 542.2136. Found 542.2133. HPLC (IA column, 95:5 n-Hex/iPrOH, 30 °C, 1.0 mL/min): t_R 6.56 min (minor) and 8.90 min (major).

***tert*-Butyl (2*S*,3*R*,*S*$_a$)-3-(1-benzyl-5-chloro-3-(2-(ethylthio)phenyl)-1*H*-indol-2-yl)-3-hydroxy-2-methylpropanoate (2x)**

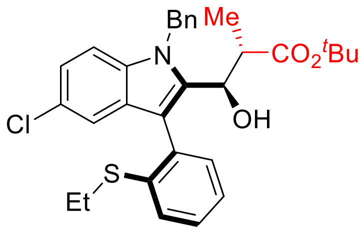

Following the general procedure from **1x** (0.10 mmol, 41 mg), purification by column chromatography afforded **2x** as a pale-yellow foam (27 mg, 50%). d.r. > 20:1. Major diastereomer: $[\alpha]^{20}_{D}$ +15.5 (*c* 0.36, $CHCl_3$) for 94% *ee*. ^{1}H-NMR (400 MHz, $CDCl_3$) δ 7.40 – 7.33 (m, 2H), 7.32 – 7.22 (m, 6H), 7.10 – 7.02 (m, 4H), 5.81 (d, *J* = 17.1 Hz, 1H), 5.67 (d, *J* = 17.1 Hz, 1H), 4.95 (d, *J* = 11.0 Hz, 1H), 2.93 – 2.63 (m, 3H), 1.39 (s, 9H), 1.25 (t, *J* = 7.4 Hz, 3H), 0.67 (d, *J* = 7.0 Hz, 3H). ^{13}C-NMR (100 MHz, $CDCl_3$) δ 174.4, 138.0, 137.8, 136.7, 136.4, 132.8, 132.5, 128.8, 128.5, 128.3, 127.3, 126.8, 126.0, 126.0, 125.4, 123.0, 119.1, 115.1, 111.6, 80.9, 70.3, 48.6, 45.7, 28.1, 26.3, 14.6, 13.8. HRMS (ESI) calcd. for $C_{31}H_{34}O_3NClNaS$ (M + Na$^+$) 558.1840. Found 558.1834. HPLC (IA column, 95:5 n-Hex/iPrOH, 30 °C, 1.0 mL/min): t_R 6.81 min (minor) and 10.73 min (major).

***tert*-Butyl (2*S*,3*R*,*S*$_a$)-3-(1-benzyl-5-bromo-3-(2-(ethylthio)phenyl)-1*H*-indol-2-yl)-3-hydroxy-2-methylpropanoate (2y)**

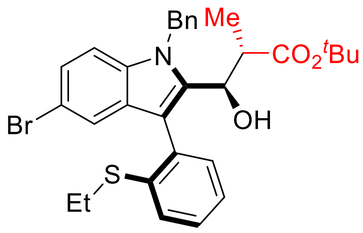

Following the general procedure from **1y** (0.10 mmol, 45 mg), purification by column chromatography afforded **2y** as a pale-yellow foam (44 mg, 76%). d.r. > 20:1. Major diastereomer: $[\alpha]^{20}_{D}$ +6.4 (*c* 0.54, $CHCl_3$) for 94% *ee*. ^{1}H-NMR (400 MHz, $CDCl_3$) δ 7.45 (d, *J* = 1.7 Hz, 1H), 7.40 – 7.23 (m, 7H), 7.21 (dd, *J* = 8.7, 1.8 Hz, 1H), 7.05 (d, *J* = 7.2 Hz, 2H), 7.00 (d, *J* = 8.7 Hz, 1H), 5.81 (d, *J* = 17.1 Hz, 1H), 5.67 (d, *J* = 17.1 Hz, 1H), 4.96 (d, *J* = 11.0 Hz, 1H), 1.39 (s, 9H), 1.26 (t, *J* = 7.3 Hz, 3H), 0.67 (d, *J* = 7.0 Hz, 3H). ^{13}C-NMR (100 MHz, $CDCl_3$) δ 174.3, 137.8, 137.7, 136.6, 136.5, 132.7, 132.4, 129.1, 128.7, 128.2, 127.2, 126.6, 125.8, 125.4, 125.2, 122.0, 114.8, 113.5, 111.9, 80.8, 70.1, 48.4, 45.5, 28.0, 26.2, 14.5, 13.7. HRMS (ESI) calcd. for $C_{31}H_{34}O_3NBrNaS$ (M + Na$^+$) 602.1335. Found 602.1333. HPLC (IA column, 95:5 n-Hex/iPrOH, 30 °C, 1.0 mL/min): t_R 7.23 min (minor) and 12.19 min (major).

***tert*-Butyl (2*S*,3*R*,*S*$_a$)-3-(1-benzyl-3-(2-(benzylthio)phenyl)-1*H*-indol-2-yl)-3-hydroxy-2-methylpropanoate (2z)**

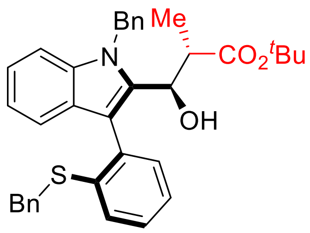

Following the general procedure from **1z** (0.10 mmol, 43 mg), purification by column chromatography afforded **2z** as a yellow foam (54 mg, 95%). d.r. > 20:1. Major diastereomer: $[\alpha]^{20}_{D}$ +29.3 (*c* 0.52, $CHCl_3$) for 95% *ee*. ^{1}H-NMR (400 MHz, $CDCl_3$) δ 7.46 – 7.04 (m, 18H), 5.86 (d, *J* = 17.2 Hz, 1H), 5.68 (d, *J* = 17.2 Hz, 1H), 5.03 (dd, *J* = 10.9, 3.0 Hz, 1H), 4.04 – 3.89 (m, 2H), 3.03 (d, *J* = 3.1 Hz, 1H), 2.75 (dq, *J* = 14.0, 7.0 Hz, 1H), 1.42 (s, 9H), 0.69 (d, *J* = 7.0 Hz, 3H). ^{13}C-NMR (100 MHz, $CDCl_3$) δ 174.6, 138.3, 138.1, 137.7, 136.8, 135.2, 134.1, 132.7, 128.9, 128.7, 128.6, 128.3, 128.1, 127.6, 127.3,

127.1, 126.0, 122.7, 120.2, 119.6, 115.4, 110.4, 80.7, 70.4, 48.4, 45.7, 37.8, 28.1, 14.6. HRMS (ESI) calcd. for $C_{36}H_{37}O_3NNaS$ (M + Na^+) 586.2386. Found 586.2379. HPLC (IA column, 95:5 n-Hex/iPrOH, 30 °C, 1.0 mL/min): t_R 8.51 min (minor) and 12.20 min (major).

Butyl (2*S*,3*R*,*S*$_a$)-3-(1-benzyl-3-(2-(methylthio)phenyl)-1*H*-indol-2-yl)-3-hydroxy-2-methylpropanoate (2aa)

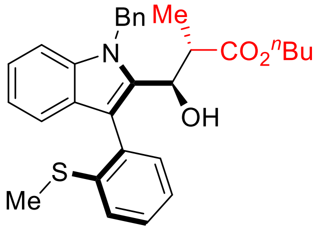

Following the general procedure from **1a** (0.10 mmol, 37 mg) and butyl acrylate (0.15 mmol, 22 μL), purification by column chromatography afforded **2aa** as a pale-yellow oil (40 mg, 78%). d.r. 5:1. Major diastereomer: 69% *ee*. ^{1}H-NMR (400 MHz, $CDCl_3$) δ 7.43 – 7.06 (m, 13H), 5.88 (d, *J* = 17.2 Hz, 1H), 5.69 (d, *J* = 17.2 Hz, 1H), 5.00 (d, *J* = 11.0 Hz, 1H), 4.11 – 4.00 (m, 2H), 3.12 (br, 1H), 2.85 (dq, *J* = 11.0, 7.0 Hz, 1H), 2.36 (s, 3H), 1.57 (d, *J* = 14.5 Hz, 2H), 1.33 (d, *J* = 14.6 Hz, 2H), 0.91 (t, *J* = 7.4 Hz, 3H), 0.73 (d, *J* = 7.1 Hz, 3H). ^{13}C-NMR (100 MHz, $CDCl_3$) δ 175.2, 139.2, 138.2, 138.1, 134.9, 132.4, 132.2, 128.8, 128.7, 128.2, 127.1, 125.9, 124.6, 124.4, 122.8, 120.2, 119.6, 115.4, 110.3, 70.2, 64.5, 48.2, 44.8, 30.6, 19.1, 15.2, 14.5, 13.7. HPLC (IA column, 98:2 n-Hex/iPrOH, 30 °C, 1.0 mL/min): t_R 28.34 min (minor) and 32.77 min (major).

***tert*-Butyl (2*S*,3*R*)-3-(3-(benzo[*b*]thiophen-7-yl)-1-benzyl-1*H*-indol-2-yl)-3-hydroxy-2-methylpropanoate (2ab)**

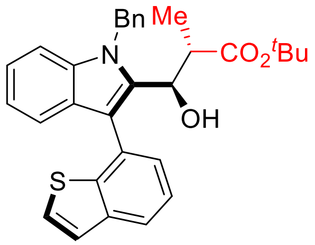

Following the general procedure from **1ab** (0.10 mmol, 37 mg), purification by column chromatography afforded **2ab** as a pale-yellow oil (46 mg, 96%). d.r. 1.6:1. ^{1}H-NMR (400 MHz, $CDCl_3$) δ 7.88 (t, *J* = 8.2 Hz, 1H), 7.51 (t, *J* = 7.5 Hz, 1H), 7.47 – 7.06 (m, 12H), 5.87 (d, *J* = 10.9 Hz, 1H), 5.74 (d, *J* = 17.2 Hz, 1H), 5.17 (d, *J* = 10.7 Hz, 1H, minor), 5.09 (dd, *J* = 10.7, 3.5 Hz, 1H, major), 3.10 (d, *J* = 3.6 Hz, 1H), 2.80 – 2.65 (m, 1H), 1.37 (s, 9H), 0.74 (d, *J* = 7.1 Hz, 3H, major), 0.60 (d, *J* = 7.2 Hz, 3H, minor). ^{13}C-NMR (100 MHz, $CDCl_3$) δ 175.0 (minor), 174.8 (major), 142.0 (major), 141.9 (minor), 140.1 (major), 140.0 (minor), 138.3 (major), 138.2 (minor), 138.0 (major), 137.7 (minor), 134.7 (major), 134.6 (minor), 129.8 (minor), 129.5 (major), 128.8, 127.3 (major), 127.1 (minor), 126.7 (major), 126.6 (minor), 126.1, 124.9, 124.7 (minor), 124.7 (major), 124.2, 123.0 (major), 123.0 (minor), 122.8 (minor), 122.7 (major), 121.0 (major), 120.8 (minor), 120.2 (major), 120.0, 119.9 (minor), 116.6 (minor), 116.3 (major), 110.6 (minor), 110.5 (major), 81.4 (minor), 81.2 (major), 70.0 (major), 69.8 (minor), 48.6 (minor), 48.5 (major), 45.9 (major), 45.6 (minor), 28.1, 14.8 (major), 14.6 (minor). HRMS (ESI) calcd. for $C_{31}H_{31}O_3NNaS$ (M + Na^+) 520.1917. Found 520.1909.

***tert*-Butyl (2*S*,3*R*)-3-(1-benzyl-3-(dibenzo[*b*,*d*]thiophen-4-yl)-1*H*-indol-2-yl)-3-hydroxy-2-methylpropanoate (2ac)**

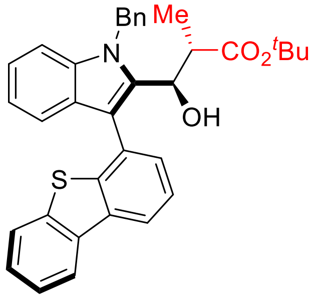

Following the general procedure from **1ac** (0.10 mmol, 42 mg), purification by column chromatography afforded **2ac** as a pale-yellow oil (51 mg, 96%). d.r. 1.25:1. ^{1}H-NMR (400 MHz, $CDCl_3$) δ 8.26 – 8.19 (m, 2H), 7.82 – 7.76 (m, 1H), 7.64 – 7.15 (m, 12H), 7.10 (ddd, *J* = 7.8, 6.1, 1.9 Hz, 1H), 5.88 (d, *J* = 17.1 Hz, 1H), 5.75 (d, *J* = 17.8 Hz, 1H), 5.19 (dd, *J* = 10.7, 3.5 Hz, 1H, minor), 5.10 (dd, *J* = 10.7, 3.6 Hz, 1H, major), 3.43 (d, *J* = 3.6 Hz, 1H, minor), 3.16 (d, *J* = 3.7 Hz, 1H, major), 2.71 (ddq, *J* = 14.3, 10.7, 7.2 Hz, 1H), 1.34 (s, 9H, major), 1.34 (s, 9H, minor), 0.73 (d, *J* = 7.1 Hz, 3H, major), 0.63 (d, *J* = 7.2 Hz, 3H, minor). ^{13}C-NMR (100 MHz, $CDCl_3$) δ 178.0 (minor), 174.8 (major), 141.7 (major), 141.7 (minor), 139.9 (major), 139.6 (minor), 138.3 (major), 138.2 (minor), 138.0 (minor), 137.7 (major), 136.5 (minor), 136.1 (major), 134.9 (major), 134.7 (minor), 130.1 (minor), 129.8 (major), 129.7 (major), 129.2 (minor), 128.9, 127.3, 127.1, 126.8 (major), 126.8 (minor), 126.1, 125.1 (minor), 125.0 (major), 124.5 (major), 124.4 (minor), 123.1 (minor), 123.0 (major), 122.9, 121.9, 121.0, 120.6, 120.3, 120.0 (major), 120.0 (minor), 116.4 (minor), 116.1 (major), 110.6 (minor), 110.6 (major), 81.5 (minor), 81.2 (major), 70.0 (major), 69.8 (minor), 48.6 (minor), 48.5 (major), 45.9 (major), 45.7 (minor), 28.1 (major), 27.1 (minor), 14.8 (major), 14.6 (minor). HRMS (ESI) calcd. for $C_{35}H_{33}O_3NNaS$ (M + Na^+) 570.2073. Found 570.2066.

5. Representative transformations

(1*R*,2*R*,*S*$_a$)-1-(1-Benzyl-3-(2-(ethylthio)phenyl)-1*H*-indol-2-yl)-2-methylpropane-1,3-diol (3)

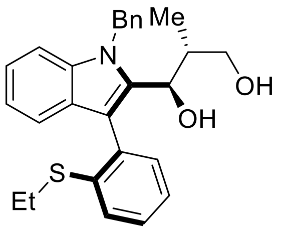

Over a solution of **2m** (0.05 mmol, 25 mg) in THF (0.5 mL) at 0 ºC under inert atmosphere, $LiAlH_4$ (0.13 mmol, 5.0 mg) was added in small portions. After stirring the reaction mixture at 0º C for 1 h, it was allowed to warm up to room temperature and the stirring continued for 1 h. The reaction was quenched by addition of NH_4Cl (aq. sat.) and extracted with ethyl acetate (× 3). The combined organic layers were dried over $MgSO_4$ and concentrated. The residue was purified by silica gel column chromatography using cyclohexane/EtOAc (5:1 → 3:1) as eluent to give **3** as a colourless oil (21 mg, 99%). ^{1}H-NMR (400 MHz, $CDCl_3$) δ 7.44 – 7.33 (m, 4H), 7.32 – 7.21 (m, 4H), 7.20 – 7.13 (m, 2H), 7.12 – 7.03 (m, 3H), 5.89 (d, *J* = 17.3 Hz, 1H), 5.70 (d, *J* = 17.3 Hz, 1H), 4.69 (d, *J* = 12.7 Hz, 1H), 3.61 – 3.49 (m, 2H), 3.44 (d, *J* = 2.2 Hz, 1H), 3.39 (d, *J* = 7.7 Hz, 1H), 2.86 – 2.69 (m, 2H), 2.27 (ddq, *J* = 10.7, 7.3, 3.7 Hz, 1H), 1.23 (t, *J* = 7.4 Hz, 3H), 0.41 (d, *J* = 6.8 Hz, 3H). ^{13}C-NMR (100 MHz, $CDCl_3$) δ 138.2, 138.0, 137.2, 136.1, 133.9, 132.5, 128.7, 127.9, 127.2, 127.0, 125.7, 125.5, 122.7, 120.2, 119.4, 114.7, 110.4, 75.0, 69.0, 48.3, 39.1, 26.3, 13.7, 13.7. HRMS (ESI) calcd. for $C_{27}H_{29}O_2NNaS$ (M + Na^+) 454.1811. Found 454.1806.

(4*R*,5*R*,*S*$_a$)-1-Benzyl-3-(2-(ethylthio)phenyl)-2-(2,2,5-trimethyl-1,3-dioxan-4-yl)-1*H*-indole (4)

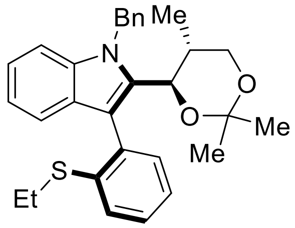

Over a solution of **3m** (0.05 mmol, 21 mg) in 2,2-dimethoxypropane (1.25 mL) *p*-toluenesulfonic acid monohydrate (0.05 mmol, 9.5 mg) was added and the reaction mixture was allowed to stir at room temperature for 1.5 h. $NaHCO_3$ (aq. sat.) was added, and the mixture was extracted with CH_2Cl_2 (x3). The combined organic phases were dried over $MgSO_4$ and concentrated. Purification of the residue by silica gel column chromatography, using cyclohexane/EtOAc (3:1) as eluent, afforded **4** as a white foam (20 mg, 84%). $[\alpha]^{20}_D$ +43.4 (*c* 1.00, $CHCl_3$) for 95% *ee*. ^{1}H-NMR (400 MHz, $CDCl_3$) δ 7.44 – 7.33 (m, 3H), 7.32 – 7.19 (m, 5H), 7.10 (dp, *J* = 22.3, 8.0 Hz, 5H), 5.91 (d, *J* = 17.1 Hz, 1H), 5.72 (d, *J* = 17.1 Hz, 1H), 4.71 (d, *J* = 11.0 Hz, 1H), 3.62 (dd, *J* = 11.7, 4.9 Hz, 1H), 3.42 (t, *J* = 11.3 Hz, 1H), 2.80 (q, *J* = 7.3 Hz, 2H), 2.04 (dh, *J* = 11.2, 5.8, 5.2 Hz, 1H), 1.43 (s, 3H), 1.37 (s, 3H), 1.23 (t, *J* = 7.3 Hz, 3H), 0.42 (d, *J* = 6.6 Hz, 3H). ^{13}C-NMR (100 MHz, $CDCl_3$) δ 138.7, 138.4, 137.6, 134.2, 133.7, 133.3, 128.6, 127.9, 127.0, 126.7, 126.1, 124.7, 122.4, 119.9, 119.8, 115.6, 110.3, 99.1, 72.3, 66.5, 48.4, 33.6, 29.9, 26.6, 23.1, 18.8, 14.1, 13.0. HRMS (ESI) calcd. for $C_{30}H_{33}O_2NNaS$ (M + Na$^+$) 494.2124. Found 494.2117. HPLC (IB column, 98:2 n-Hex/iPrOH, 30 °C, 1.0 mL/min): t_R 4.94 min (minor) and 5.59 min (major).

(2*S*,3*R*,*S*$_a$)-*tert*-Butyl-3-(1-benzyl-3-(2-(ethylsulfonyl)phenyl)-1*H*-indol-2-yl)-3-hydroxy-2-methylpropanoate (5)

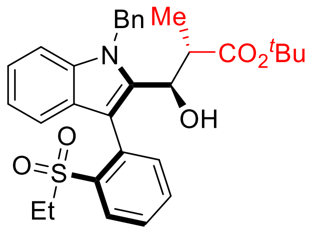

Over a solution of **2m** (0.05 mmol, 25 mg) in dry CH_2Cl_2 (0.5 mL) at room temperature, *m*CPBA (0.13 mmol, 28 mg) was added and the mixture was stirred for 2 h. The reaction was quenched by addition of $NaHCO_3$ (aq. sat.) and extracted with CH_2Cl_2 (x3). The combined organic phases were dried over $MgSO_4$, concentrated and the residue was purified by silica gel column chromatography, using cyclohexane/EtOAc (3:2) as eluent, affording **5** as a white solid (26 mg, 97%). $[\alpha]^{20}_D$ +31.1 (*c* 0.44, $CHCl_3$) for . 95% *ee* ^{1}H-NMR (400 MHz, $CDCl_3$) δ 8.31 (dd, *J* = 8.0, 1.5 Hz, 1H), 7.72 (td, *J* = 7.5, 1.5 Hz, 1H), 7.64 (td, *J* = 7.7, 1.5 Hz, 1H), 7.48 (dd, *J* = 7.4, 1.4 Hz, 1H), 7.32 – 7.20 (m, 3H), 7.19 – 7.03 (m, 6H), 5.87 – 5.75 (m, 2H), 4.80 (dd, *J* = 11.1, 3.3 Hz, 1H), 3.77 (d, *J* = 3.4 Hz, 1H), 2.77 (dq, *J* = 11.3, 6.9 Hz, 1H), 2.61 (dq, *J* = 14.8, 7.4 Hz, 1H), 2.51 (dq, *J* = 14.5, 7.3 Hz, 1H), 1.37 (s, 9H), 0.95 (t, *J* = 7.4 Hz, 3H), 0.66 (d, *J* = 7.0 Hz, 3H). ^{13}C-NMR (100 MHz, $CDCl_3$) δ 174.1, 138.2, 138.1, 137.8, 137.3, 135.2, 134.6, 133.4, 130.8, 128.7, 128.4, 127.9, 127.3, 126.2, 122.9, 120.7, 118.3, 111.9, 111.1, 80.6, 70.2, 48.7, 48.7, 45.8, 28.1, 14.6, 7.4. HRMS (ESI) calcd. for $C_{31}H_{35}O_5NNaS$ (M + Na$^+$) 556.2128. Found 556.2125. HPLC (IA column, 95:5 n-Hex/iPrOH, 30 °C, 1.0 mL/min): t_R 19.61 min (minor) and 21.24 min (major).

(2*S*,3*R*,*S*$_a$)-*tert*-Butyl-3-(1-benzyl-3-(2-((*R*)-ethylsulfinyl)phenyl)-1*H*-indol-2-yl)-3-hydroxy-2-methylpropanoate (6)

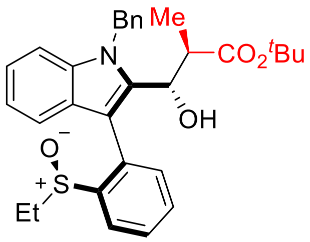

Over a solution of **2m** (0.1 mmol, 50.1 mg) in dry CH_2Cl_2 (1.0 mL) at -78 ºC, *m*CPBA (1.0 mmol, 22.4 mg) was added. After stirring overnight at this temperature, the reaction mixture was quenched by addition of $NaHCO_3$ (aq. sat.) and the mixture was extracted with CH_2Cl_2 (x3). The combined organic phases were dried over $MgSO_4$, concentrated and the residue was purified by silica gel column chromatography, using cyclohexane/EtOAc (3:2 → 1:3) as eluent, affording **6** as a white solid (46.6 mg, 90%). The pure product was crystallized by slow evaporation of a solution of the product in CH_2Cl_2 to give **6** as white crystals suitable for X-Ray analysis. $[\alpha]^{20}_D$ -76.1 (*c* 0.51, $CHCl_3$) for 95 % *ee*. ^{1}H-NMR (400 MHz, $CDCl_3$) δ 8.04 (dd, *J* = 7.8, 1.4 Hz, 1H), 7.64 (td, *J* = 7.6, 1.4 Hz, 1H), 7.58 (td, *J* = 7.4, 1.5 Hz, 1H), 7.39 (dd, *J* = 7.4, 1.4 Hz, 1H), 7.32 – 7.21 (m, 4H), 7.19 – 7.15 (m, 2H), 7.12 – 7.05 (m, 3H), 5.77 (s, 2H), 4.98 (dd, *J* = 10.7, 3.9 Hz, 1H), 3.42 (d, *J* = 4.0 Hz, 1H), 2.72 (dq, *J* = 10.7, 7.1 Hz, 1H), 2.53 (dq, *J* = 14.7, 7.3 Hz, 1H), 2.27 (dq, *J* = 14.6, 7.4 Hz, 1H), 1.38 (s, 9H), 0.94 (t, *J* = 7.4 Hz, 3H), 0.63 (d, *J* = 7.1 Hz, 3H). ^{13}C-NMR (100 MHz, $CDCl_3$) δ 174.2, 142.4, 138.1, 138.0, 135.1, 132.9, 132.4, 130.8, 128.8, 128.6, 127.7, 127.4, 126.1, 125.3, 123.2, 120.8, 118.9, 112.0, 110.9, 81.2, 70.1, 48.8, 46.6, 46.4, 28.1, 14.5, 5.5. HRMS (ESI) calcd. for $C_{31}H_{35}O_4NNaS$ (M + Na^+) 540.2179. Found 540.2179. HPLC (IA column, 90:10 n-Hex/iPrOH, 30 °C, 1.0 mL/min): t_R 6.17 min (minor) and 8.95 min (major).

6. Computational Studies

Computational methods

All of the calculations were performed using the Gaussian16 program.[4] Computations were done using wb97xd functional[5] in conjunction with standard basis sets def2SVP and def2TZVP.[6,7] Geometry full optimizations were made at ωB97XD/def2SVP level. Single point calculations using def2TZVP basis set were carried out over optimized geometries to obtain the energy values. Solvent effects (toluene) were considered using the SMD model.[8] The nature of stationary points was defined on the basis of calculations of normal vibrational frequencies (force constant Hessian matrix). The optimizations were carried out using the Berny analytical gradient optimization method.[9] Minimum energy pathways for the reactions studied were found by gradient descent of transition states in the forward and backward direction of the transition vector (IRC analysis).[10] Analytical second derivatives of the energy were calculated to classify the nature of every stationary point, to determine the harmonic vibrational frequencies, and to provide zero-point vibrational energy corrections. The thermal and entropic contributions to the free energies were also obtained from the vibrational frequency calculations, using the unscaled frequencies. Correction to free energy was made by substracing S_{trans} contribution and considering a 1M concentration.[11] Structural representations were generated using CYLView.[12]

NCI (non-covalent interactions) were computed using the methodology previously described.[13,14] Quantitative data were obtained with the NCIPLOT4 program.[15] A density cutoff of ρ=0.5 a.u. was applied and isosurfaces of s(r) =0.5 were coloured by $sign(\lambda_2)\rho$ in the [-0.03,0.03] a.u. range using VMD software.[16] s(**r**) against $sign(\lambda_2)\rho(\mathbf{r})$ plots were generated with gnuplot software.[17]

Conformational Studies

A one-dimensional relaxed sca was carried out along the dihedral angle in both the indole derivative and the biphenyl derivative (Figure S1). The two maxima were used as starting points for locating the corresponding transition structures. For that maxima facing the carbonyl group and the methylthio group two different transition structures should be considered depending on the relative orientation of both groups (*s-cis* and *s-trans*). In both cases the most stable transition structure resulted that facing carbonyl and methylthio groups with a relative *s-cis* disposition, presumably due to the presence of favourable interactions as confirmed by NCI analyses (see below and main text of the manuscript).

Figure S1. Relaxed scans for compounds **A** and **B**.

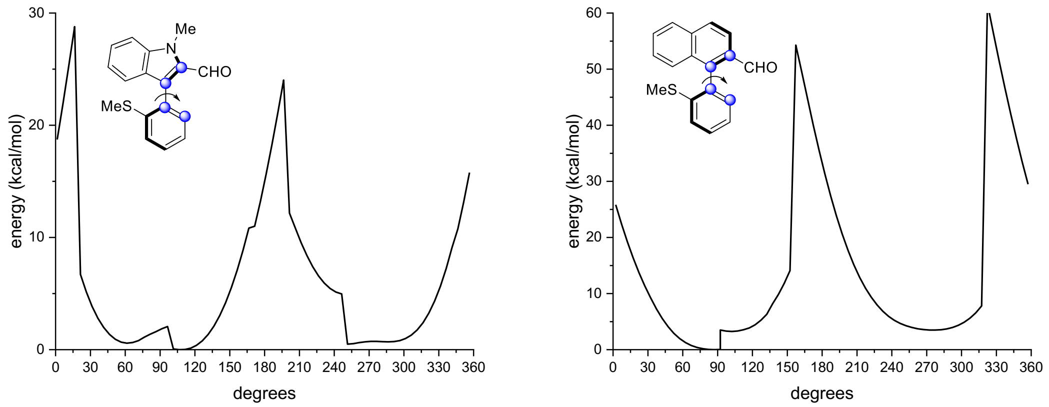

Figure S2. Optimized structures (wb97xd/def2svp/pcm=toluene) of the transition structures corresponding to compound **A**.

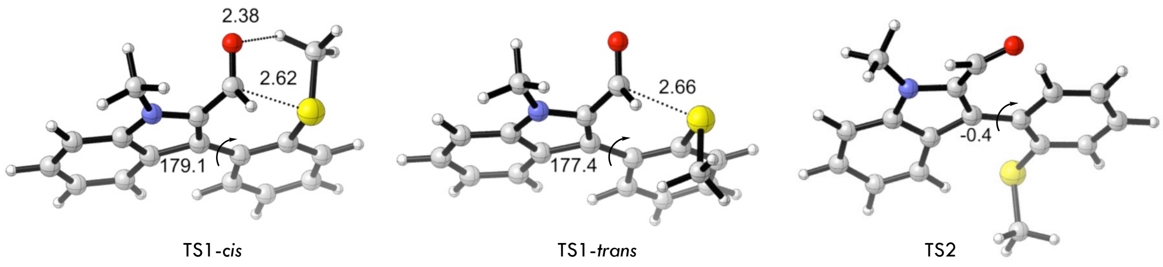

Figure S3. Intrinsic reaction coordinates (wb97xd/def2svp/pcm=toluene) for the transition structures corresponding to compound **A**.

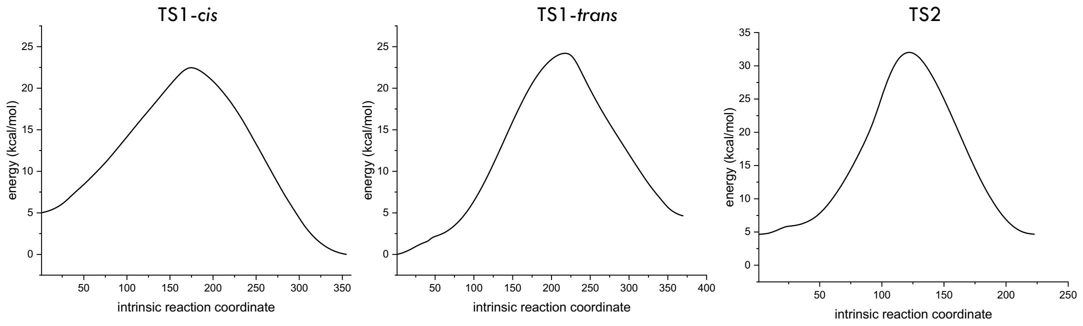

Figure S4. NCI analyses for the transition structures corresponding to compound **A**. Thin, delocalized green surface indicates van der Waals interactions. Small, lenticular, bluish surfaces indicate strong interactions such as hydrogen bonding. Steric clashes are shown as red isosurfaces.

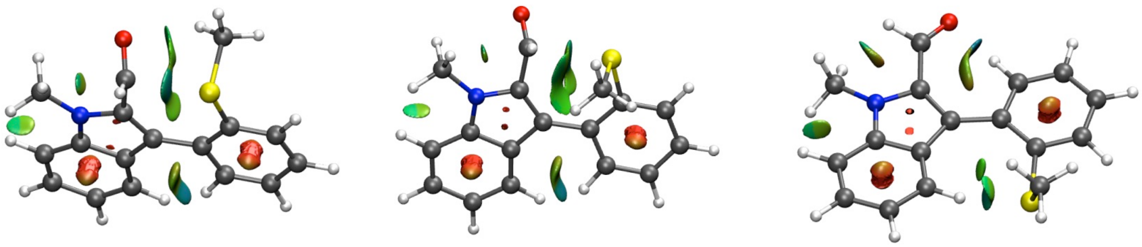

TS1a-*cis* TS1a-*trans* TS2a

Figure S5. Optimized structures (wb97xd/def2svp/pcm=toluene) of the transition structures corresponding to compound **B**.

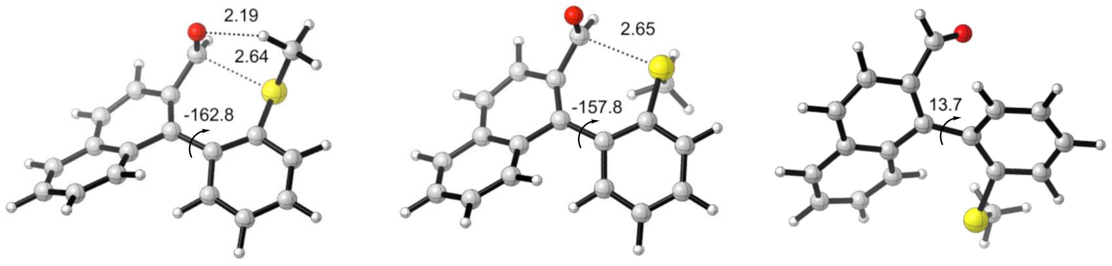

TS1b-*cis* TS1b-*trans* TS2b

Figure S6. NCI analyses for the transition structures corresponding to compound **B**. Thin, delocalized green surface indicates van der Waals interactions. Small, lenticular, bluish surfaces indicate strong interactions such as hydrogen bonding. Steric clashes are shown as red isosurfaces.

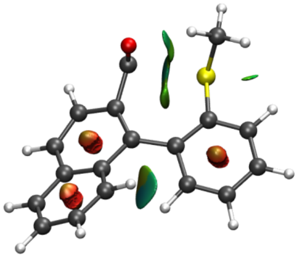

TS1b-*cis*

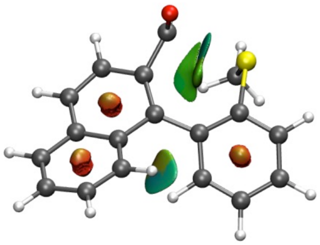

TS1b-*trans*

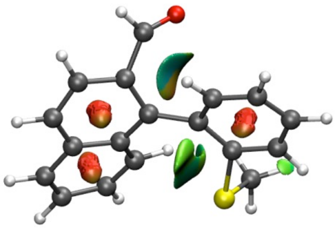

TS2b

Figure S7. Optimized structures (wb97xd/def2svp/pcm=toluene) of the transition structures corresponding to compound **C**. Right: NCI **D**. Thin, delocalized green surface indicates van der Waals interactions. Small, lenticular, bluish surfaces indicate strong interactions such as hydrogen bonding. Steric clashes are shown as red isosurfaces.

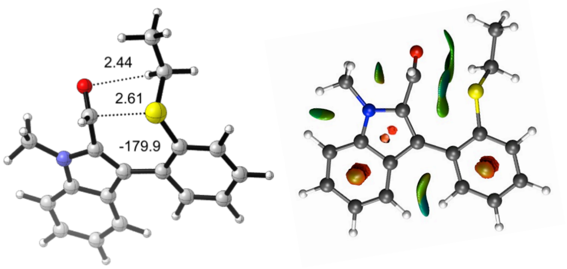

TS1c-*cis*

Figure S8. Optimized structures (wb97xd/def2svp/pcm=toluene) of the transition structures corresponding to compounds **A-Me** and **B-Me** (compounds in which the formyl group has been replaced by a methyl group).

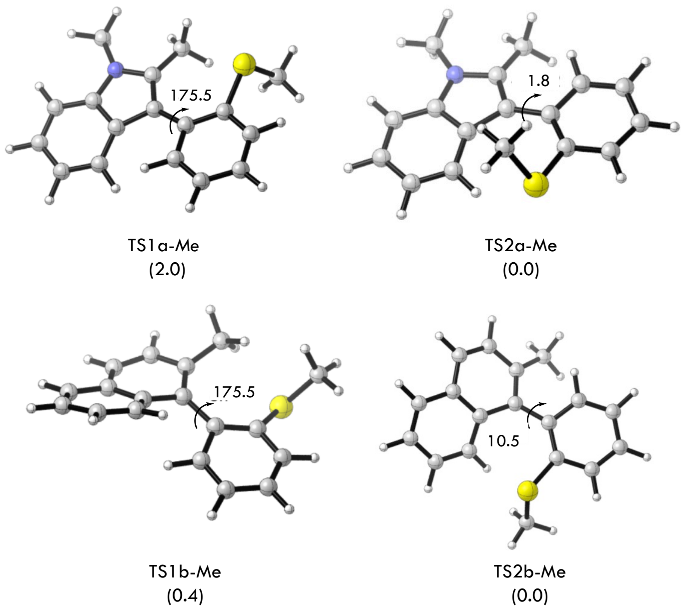

Energies

Table S4. *Absolute (hartree) and relative (kcal/mol) energies (wb97xd/def2tzvp/smd=toluene// wb97xd/def2svp/smd=toluene) corresponding to the conformational analysis of compound **A**.*

	E_0	ΔE_0	G	ΔG	im. freq
**A**	-1184.763832	0.0	-1184.810520	0.0	
**TS1a-cis**	-1184.727730	22.7	-1184.772185	24.1	-88.9
**TS1a-trans**	-1184.725877	23.8	-1184.770041	25.4	-67.0
**TS2a**	-1184.711029	33.1	-1184.755536	34.5	-71.5

Table S5. *Absolute (hartree) and relative (kcal/mol) energies (wb97xd/def2tzvp/smd=toluene// wb97xd/def2svp/smd=toluene) corresponding to the conformational analysis of compound **B**.*

	E_0	ΔE_0	G	ΔG	im. freq
**B**	-1167.522088	0.0	-1167.567507	0.0	
**TS1b-cis**	-1167.465048	35.8	-1167.507061	37.9	-41.1
**TSb-trans**	-1167.463101	37.0	-1167.505576	38.9	-40.3
**TS2b**	-1167.453630	43.0	-1167.496611	44.5	-38.9

Table S6. *Absolute (hartree) and relative (kcal/mol) energies (wb97xd/def2tzvp/smd=toluene// wb97xd/def2svp/smd=toluene) corresponding to the conformational analysis of compound **C** (only the TS-cis is showed for comparison with **A** and **B**).*

	E_0	ΔE_0	G	ΔG	im. freq
**C**	-1773.464329	0.0	-1773.514917	0.0	
**TS1c-cis**	-1773.424186	25.2	-1773.472123	26.9	-42.1

Table S7. *Absolute (hartree) and relative (kcal/mol) energies (wb97xd/def2tzvp/smd=toluene// wb97xd/def2svp/smd=toluene) corresponding to the conformational analysis of compounds **A-Me** and **B-Me**.*

	E_0	ΔE_0	G	ΔG	im. freq
**A-Me**	-1110.733721	0.0	-1110.779350	0.0	
**TS1ba-Me**	-1110.691154	26.7	-1110.734254	28.3	-63.3212
**TS2a-Me**	-1110.694795	24.4	-1110.737476	26.3	-69.0173
**B-Me**	-1093.490910	0.0	-1093.535201	0.0	
**TS1b-Me**	-1093.422128	43.2	-1093.463487	45.0	-50.0597
**TS2b-Me**	-1093.422652	42.8	-1093.464093	44.6	-44.9908

Cartesian coordinates

A

0 1

C	-1.2716526737	-0.9369881860	-0.4066779007
C	-1.2626518780	-2.3249535117	-0.6494453671
C	-2.4494108900	-3.0237212654	-0.5469946553
C	-3.6518841095	-2.3609380571	-0.2031742373
C	-3.6895681036	-1.0009565727	0.0446526353
C	-2.4818579720	-0.2849760774	-0.0567688184
C	-0.2512231014	0.0623269131	-0.4231769933
C	-0.8716466006	1.2581562243	-0.0932535501
N	-2.2190261696	1.0456261813	0.1354415079
H	-0.3289023300	-2.8274263327	-0.9122134318
H	-2.4670356650	-4.0997199922	-0.7309408224
H	-4.5756039438	-2.9394234877	-0.1311141421
H	-4.6283112286	-0.5110744170	0.3072115173
C	-0.2151792434	2.5624533756	-0.0186833619
O	-0.7531887583	3.6102938430	0.2651152102
H	0.8714913824	2.5186860192	-0.2631628872
C	-3.1926128604	2.0586229646	0.4913664758
H	-2.8550848828	2.6269926112	1.3658484795
H	-3.3513568924	2.7628471834	-0.3369209764
H	-4.1429328017	1.5678086525	0.7292853551
C	1.1783212518	-0.1607283736	-0.7425672432
C	1.5416874216	-0.4910725905	-2.0519029217
C	2.1794863223	-0.0911195373	0.2523890859

C	2.8690846278	-0.7375537260	-2.3965128248
H	0.7571631686	-0.5485610784	-2.8102558660
C	3.5122795640	-0.3336005237	-0.1008776906
C	3.8517828532	-0.6531382516	-1.4148261096
H	3.1313910730	-0.9906837960	-3.4251415881
H	4.3030638449	-0.2825395462	0.6475693009
H	4.8982849377	-0.8401609748	-1.6655452284
S	1.6575338656	0.2896148755	1.9053872078
C	3.1958588056	0.2497076879	2.8457480193
H	2.9081704043	0.4724674104	3.8818360895
H	3.6664985816	-0.7430801376	2.8174901218
H	3.9056760006	1.0167404938	2.5055726103

A-Me

0 1

C	0	1.4164760000	0.7402160000	0.1278410000
C	0	1.5883890000	1.9882380000	0.7465830000
C	0	2.8488860000	2.3375510000	1.2118410000
C	0	3.9433030000	1.4605470000	1.0736850000
C	0	3.7990120000	0.2186780000	0.4682600000
C	0	2.5259340000	-0.1279960000	-0.0002810000
C	0	0.2894550000	0.0592870000	-0.4550520000
C	0	0.7510550000	-1.1574290000	-0.9117410000
N	0	2.0994070000	-1.2746050000	-0.6301900000
H	0	0.7409840000	2.6693990000	0.8561830000
H	0	2.9983490000	3.3059360000	1.6945630000

H	0	4.9231820000	1.7624010000	1.4506410000
H	0	4.6489220000	-0.4587040000	0.3637410000
C	0	2.9620020000	-2.3857280000	-0.9485200000
H	0	2.3727860000	-3.2098140000	-1.3656910000
H	0	3.7272830000	-2.0963860000	-1.6858360000
H	0	3.4686340000	-2.7527780000	-0.0430980000
C	0	-1.0860940000	0.5947310000	-0.5579240000
C	0	-1.3352630000	1.7085920000	-1.3673230000
C	0	-2.1594240000	0.0318500000	0.1702030000
C	0	-2.6108630000	2.2566740000	-1.4849990000
H	0	-0.4976360000	2.1442210000	-1.9174750000
C	0	-3.4432720000	0.5748700000	0.0380980000
C	0	-3.6653160000	1.6789020000	-0.7839190000
H	0	-2.7796450000	3.1249540000	-2.1249740000
H	0	-4.2863860000	0.1487850000	0.5818340000
H	0	-4.6740170000	2.0895200000	-0.8688620000
S	0	-1.7796880000	-1.3323770000	1.2406200000
C	0	-3.3607100000	-1.6867210000	2.0340980000
H	0	-3.1573760000	-2.5063300000	2.7363030000
H	0	-3.7352860000	-0.8233830000	2.6022340000
H	0	-4.1200860000	-2.0219000000	1.3134760000
C	0	-0.0013810000	-2.2285610000	-1.6261300000
H	0	0.4566610000	-2.4637600000	-2.5996580000
H	0	-0.0484600000	-3.1566670000	-1.0346120000
H	0	-1.0318700000	-1.8974350000	-1.8056610000

B

0 1

C	0	-0.4040620000	0.4287500000	-0.4132710000
C	0	-1.5105320000	-0.4568630000	-0.1999060000
C	0	-0.5954010000	1.7978490000	-0.3053070000
C	0	-1.3709840000	-1.8712230000	-0.2748800000
C	0	-2.7923900000	0.0898680000	0.1020510000
C	0	-1.8770880000	2.3310380000	-0.0007750000
C	0	-2.4490670000	-2.6967610000	-0.0667630000
H	0	-0.3913050000	-2.2976270000	-0.4969420000
C	0	-3.8884710000	-0.7907190000	0.3070210000
C	0	-2.9460380000	1.5023280000	0.1939140000
H	0	-1.9748870000	3.4156580000	0.0717130000
C	0	-3.7225980000	-2.1517600000	0.2241010000
H	0	-2.3243770000	-3.7798760000	-0.1264440000
H	0	-4.8684210000	-0.3637100000	0.5331230000
H	0	-3.9319120000	1.9125540000	0.4250940000
H	0	-4.5725190000	-2.8185130000	0.3846380000
C	0	0.9314820000	-0.1461370000	-0.7659420000
C	0	1.8893430000	-0.4306290000	0.2295850000
C	0	1.2242390000	-0.4302420000	-2.1015200000
C	0	3.1159310000	-0.9928280000	-0.1443450000
C	0	2.4467540000	-0.9891050000	-2.4686330000
H	0	0.4713640000	-0.2071300000	-2.8612410000
C	0	3.3888850000	-1.2693240000	-1.4831480000
H	0	3.8741940000	-1.2212050000	0.6044480000
H	0	2.6589440000	-1.2026460000	-3.5177220000

H	0	4.3518570000	-1.7090610000	-1.7514690000
S	0	1.4498410000	-0.0298150000	1.8986260000
C	0	2.8939190000	-0.5459490000	2.8470420000
H	0	2.6509340000	-0.3222010000	3.8942240000
H	0	3.0794530000	-1.6249050000	2.7512690000
H	0	3.7924550000	0.0208190000	2.5655010000
C	0	0.5308490000	2.7514100000	-0.4892730000
O	0	0.4095300000	3.9506550000	-0.4109570000
H	0	1.5219060000	2.2914370000	-0.7062690000

B-Me

0 1

C	0	-0.4013890000	0.5739270000	-0.4678940000
C	0	-1.4965860000	-0.3105740000	-0.2057770000
C	0	-0.5826520000	1.9480340000	-0.4492230000
C	0	-1.3440350000	-1.7266370000	-0.1958920000
C	0	-2.7855820000	0.2346430000	0.0625160000
C	0	-1.8760210000	2.4691240000	-0.1766570000
C	0	-2.4136740000	-2.5499170000	0.0593640000
H	0	-0.3592520000	-2.1554090000	-0.3908260000
C	0	-3.8743440000	-0.6441830000	0.3187640000
C	0	-2.9441160000	1.6452290000	0.0682160000
H	0	-2.0140870000	3.5532830000	-0.1655980000
C	0	-3.6957180000	-2.0052790000	0.3169930000
H	0	-2.2757530000	-3.6334470000	0.0653420000
H	0	-4.8589010000	-0.2135350000	0.5185960000

H	0	-3.9312690000	2.0675820000	0.2720550000
H	0	-4.5377960000	-2.6715500000	0.5166660000
C	0	0.9444880000	0.0038860000	-0.7799200000
C	0	1.9008010000	-0.2130070000	0.2338050000
C	0	1.2673640000	-0.3213230000	-2.0993570000
C	0	3.1503990000	-0.7479010000	-0.1017980000
C	0	2.5125130000	-0.8524190000	-2.4316410000
H	0	0.5165480000	-0.1529420000	-2.8754820000
C	0	3.4512920000	-1.0637120000	-1.4261360000
H	0	3.9047280000	-0.9247110000	0.6649910000
H	0	2.7447290000	-1.0990180000	-3.4694330000
H	0	4.4316660000	-1.4811990000	-1.6660850000
S	0	1.4229890000	0.2217080000	1.8854440000
C	0	2.8722980000	-0.2164250000	2.8655030000
H	0	2.6112880000	0.0321240000	3.9028360000
H	0	3.0928710000	-1.2916060000	2.8072340000
H	0	3.7571360000	0.3677680000	2.5756820000
C	0	0.5685300000	2.8878130000	-0.6983290000
H	0	1.3385230000	2.7703570000	0.0806040000
H	0	1.0541720000	2.6824530000	-1.6638800000
H	0	0.2361630000	3.9348410000	-0.6950260000

C

0 1

C	0	1.7979765831	-0.7361306233	-0.0937242853
C	0	2.2303757008	-1.9849650655	-0.5818250045

C	0	3.5474772032	-2.1214442934	-0.9782928609
C	0	4.4477393007	-1.0320666510	-0.9011436449
C	0	4.0502051136	0.2071995495	-0.4313767373
C	0	2.7102265327	0.3464859999	-0.0288040918
C	0	0.5443700023	-0.2453782154	0.3865433012
C	0	0.7456464919	1.0863128171	0.7212143098
N	0	2.0571968418	1.4427016751	0.4618011251
H	0	1.5317508628	-2.8225941315	-0.6425329487
H	0	3.9040948010	-3.0804799907	-1.3594475135
H	0	5.4819050869	-1.1739412715	-1.2229394826
H	0	4.7507492090	1.0421877528	-0.3786528651
C	0	-0.2936089408	1.9626601420	1.2623955321
O	0	-0.1915525491	3.1454241053	1.5102010706
H	0	-1.2510839949	1.4241721443	1.4459951826
C	0	2.6863177478	2.7305142675	0.6746842261
H	0	1.9364132286	3.4333533108	1.0472895072
H	0	3.5019050157	2.6418202277	1.4083004781
H	0	3.0997353664	3.1101757712	-0.2715597279
C	0	-0.7087181876	-1.0275572722	0.4990669408
C	0	-0.7488606797	-2.1010038470	1.3996203220
C	0	-1.8503692661	-0.7530028691	-0.2858175410
C	0	-1.8928300726	-2.8778341986	1.5543513399
H	0	0.1404769740	-2.3102807211	1.9987113028
C	0	-2.9950034133	-1.5441152492	-0.1280696243
C	0	-3.0216094422	-2.5899283667	0.7902112217
H	0	-1.9033704000	-3.7013683033	2.2708648082
H	0	-3.8795334600	-1.3519308973	-0.7371370632
H	0	-3.9283107929	-3.1892192981	0.8970655202

S	0	-1.7618721267	0.5422832501	-1.5058654777
C	0	-3.4673994432	1.1725099928	-1.5776266321
H	0	-3.4161802683	1.9160876131	-2.3874763972
H	0	-4.1492948019	0.3834494926	-1.9285316554
C	0	-3.9543037937	1.8102816990	-0.2858208975
H	0	-4.9717072845	2.2091981930	-0.4181057923
H	0	-3.9848769569	1.0779324592	0.5350375779
H	0	-3.2948641879	2.6366428019	0.0170454771

TS1a-cis

0 1

C	0	-1.6283736237	0.8354430773	-0.0079314818
C	0	-2.1884735094	2.1223624873	0.1855984442
C	0	-3.5610814926	2.2924521607	0.2785205713
C	0	-4.4450048277	1.2046159241	0.1916136971
C	0	-3.9480284895	-0.0730092158	0.0163717943
C	0	-2.5585054190	-0.2318214985	-0.0797376039
C	0	-0.2871967593	0.2421584254	-0.1381016520
C	0	-0.5169186960	-1.1292933751	-0.2519820010
N	0	-1.8635434988	-1.4012358462	-0.2375605114
H	0	-1.5898182868	3.0217868051	0.2858905517
H	0	-3.9582256596	3.2981765732	0.4306362087
H	0	-5.5218536080	1.3652302013	0.2733155346
H	0	-4.6196322558	-0.9310360924	-0.0322353388
C	0	0.3081062775	-2.3956200834	-0.2945806863
O	0	0.4012759054	-3.0978687998	0.6902845035

H	0	0.5699403480	-2.7710503859	-1.3065014021
C	0	-2.4744910637	-2.7138136779	-0.2865499553
H	0	-1.8907222796	-3.3878415114	-0.9241064262
H	0	-2.5511135925	-3.1596645723	0.7159326172
H	0	-3.4756207316	-2.6296648077	-0.7251732164
C	0	1.0052573621	0.9866520845	-0.1315680340
C	0	0.9722921128	2.3929664471	-0.0213716221
C	0	2.3109851286	0.4385384859	-0.2258650723
C	0	2.1035738711	3.1956249097	0.0402514849
H	0	0.0195057760	2.8981202837	0.0099755522
C	0	3.4527757094	1.2501394316	-0.1696547200
C	0	3.3702458725	2.6264174239	-0.0191818734
H	0	1.9831588627	4.2773791009	0.1293738166
H	0	4.4293543515	0.7697532639	-0.2624241508
H	0	4.2732660166	3.2376848804	0.0248358259
S	0	2.6658506936	-1.2812543619	-0.5160389020
C	0	3.0770503494	-1.8588815978	1.1498945024
H	0	3.5089039807	-2.8621099688	1.0371268564
H	0	3.8162934002	-1.1896032318	1.6083722443
H	0	2.1692787755	-1.9289009395	1.7654664444

TS1a-Me

0 1

C	0	-1.6263260000	0.6471740000	0.0644390000
C	0	-2.1330700000	1.9352190000	0.3652640000
C	0	-3.4999000000	2.1763020000	0.3832920000

C	0	-4.4274730000	1.1563540000	0.1223440000
C	0	-3.9825880000	-0.1351010000	-0.1023240000
C	0	-2.6013050000	-0.3666850000	-0.0990940000
C	0	-0.3052340000	-0.0075500000	-0.0052080000
C	0	-0.5951640000	-1.3791610000	-0.0403280000
N	0	-1.9523220000	-1.5706550000	-0.1784530000
H	0	-1.4870170000	2.7618220000	0.6486100000
H	0	-3.8554390000	3.1802280000	0.6250990000
H	0	-5.4980630000	1.3708600000	0.1337170000
H	0	-4.6927180000	-0.9509000000	-0.2456170000
C	0	-2.6265910000	-2.8403750000	-0.3256970000
H	0	-2.0078910000	-3.5415320000	-0.8977310000
H	0	-2.8782930000	-3.2927380000	0.6472740000
H	0	-3.5549380000	-2.6903080000	-0.8899240000
C	0	0.9900460000	0.7461330000	-0.0962760000
C	0	0.8688330000	2.1541480000	-0.2044580000
C	0	2.3417220000	0.2886630000	-0.1557940000
C	0	1.9274490000	3.0486460000	-0.1823770000
H	0	-0.1131030000	2.5772180000	-0.3569650000
C	0	3.4100770000	1.2034210000	-0.1204160000
C	0	3.2310660000	2.5762680000	-0.0850560000
H	0	1.7225230000	4.1187590000	-0.2600960000
H	0	4.4230730000	0.7969350000	-0.1644560000
H	0	4.0876120000	3.2520030000	-0.0533730000
S	0	2.9082910000	-1.3751110000	-0.5036050000
C	0	3.5882600000	-1.8718430000	1.1024730000
H	0	4.0344650000	-2.8667230000	0.9646950000
H	0	4.3699090000	-1.1735510000	1.4325830000

H	0	2.8075340000	-1.9329450000	1.8752720000
C	0	0.2014670000	-2.6237780000	0.2078390000
H	0	0.6242500000	-3.0624250000	-0.7058510000
H	0	1.0245970000	-2.4230590000	0.8932640000
H	0	-0.4405160000	-3.3742900000	0.6886040000

TS1a-trans

0 1

C	0	1.5424190000	0.8780900000	0.0392160000
C	0	2.0362850000	2.2046090000	-0.0153590000
C	0	3.3972970000	2.4563810000	0.0533940000
C	0	4.3322930000	1.4144160000	0.1669550000
C	0	3.9022080000	0.1009630000	0.1852630000
C	0	2.5231270000	-0.1419420000	0.1108320000
C	0	0.2327620000	0.2108250000	-0.0237880000
C	0	0.5322300000	-1.1538350000	-0.0493010000
N	0	1.8876880000	-1.3545190000	0.0636640000
H	0	1.3925610000	3.0682480000	-0.1483460000
H	0	3.7463880000	3.4898260000	0.0028890000
H	0	5.3996070000	1.6384760000	0.2190340000
H	0	4.6210400000	-0.7177150000	0.2351010000
C	0	-0.2012220000	-2.4545970000	-0.3058150000
O	0	-0.0769720000	-3.0257390000	-1.3624450000
H	0	-0.6126220000	-2.9704240000	0.5900540000
C	0	2.5653370000	-2.6349200000	0.0402220000
H	0	1.9781980000	-3.3918270000	0.5742050000

H	0	2.7313720000	-2.9834740000	-0.9892080000
H	0	3.5285950000	-2.5422850000	0.5543280000
C	0	-1.0871090000	0.8996470000	-0.0740830000
C	0	-1.1204750000	2.3068870000	0.0245510000
C	0	-2.3645430000	0.2933870000	-0.2032040000
C	0	-2.2761340000	3.0657180000	-0.1091720000
H	0	-0.2077680000	2.8436850000	0.2375370000
C	0	-3.5269960000	1.0590480000	-0.3650620000
C	0	-3.4985060000	2.4464080000	-0.3424320000
H	0	-2.2096070000	4.1525870000	-0.0256160000
H	0	-4.4756500000	0.5321330000	-0.4855780000
H	0	-4.4159700000	3.0236680000	-0.4690810000
S	0	-2.6574930000	-1.4544970000	-0.0787010000
C	0	-2.6111130000	-1.6368440000	1.7267780000
H	0	-3.4002400000	-1.0310730000	2.1911500000
H	0	-2.7910470000	-2.6984450000	1.9467640000
H	0	-1.6301650000	-1.3393270000	2.1259180000

TS1b-cis

0 1

C	0	0.4109140000	-0.2178540000	-0.0698480000
C	0	1.8093990000	0.1592430000	0.0836150000
C	0	0.1908920000	-1.5871420000	-0.2562000000
C	0	2.2424960000	1.2779700000	0.8599260000
C	0	2.8376300000	-0.6886050000	-0.4322270000
C	0	1.2203770000	-2.4245210000	-0.7603910000

C	0	3.5714710000	1.6027300000	0.9894210000
H	0	1.5090410000	1.8556780000	1.4206760000
C	0	4.2015860000	-0.2921370000	-0.3436240000
C	0	2.4944740000	-1.9640440000	-0.9460740000
H	0	0.9721510000	-3.4632870000	-0.9962320000
C	0	4.5672320000	0.8401510000	0.3373070000
H	0	3.8591130000	2.4459110000	1.6210760000
H	0	4.9588100000	-0.9406190000	-0.7910400000
H	0	3.2668760000	-2.5989530000	-1.3851590000
H	0	5.6186660000	1.1219100000	0.4218880000
C	0	-0.6586950000	0.8435180000	-0.1378690000
C	0	-2.0675860000	0.6627210000	-0.2345920000
C	0	-0.2340590000	2.1853520000	-0.2984730000
C	0	-2.9304670000	1.7682110000	-0.2733700000
C	0	-1.0928000000	3.2740750000	-0.3421080000
H	0	0.8226410000	2.3933880000	-0.4308010000
C	0	-2.4665420000	3.0749690000	-0.2753500000
H	0	-4.0037810000	1.5803350000	-0.3481280000
H	0	-0.6767420000	4.2774350000	-0.4529670000
H	0	-3.1667210000	3.9119120000	-0.2986270000
S	0	-2.9060260000	-0.8827360000	-0.5708480000
C	0	-3.8108930000	-1.1585870000	0.9707070000
H	0	-4.4931910000	-1.9993780000	0.7863440000
H	0	-3.1081390000	-1.4238970000	1.7727920000
H	0	-4.3965690000	-0.2687830000	1.2359910000
C	0	-0.9404840000	-2.4070450000	0.3009670000
O	0	-1.1761100000	-2.4068440000	1.4897830000
H	0	-1.3265310000	-3.2071510000	-0.3714120000

TS1b-Me

0 1

C	0	0.3773920000	-0.4056360000	0.0076420000
C	0	1.7585160000	0.0859370000	0.1124330000
C	0	0.2706070000	-1.8046250000	0.0178560000
C	0	2.1124510000	1.2757530000	0.8174440000
C	0	2.8464160000	-0.7066960000	-0.3546370000
C	0	1.3753120000	-2.5836860000	-0.4448230000
C	0	3.4138990000	1.7131660000	0.9107220000
H	0	1.3378000000	1.8370530000	1.3392430000
C	0	4.1746630000	-0.2038620000	-0.3123250000
C	0	2.5925100000	-2.0487390000	-0.7433460000
H	0	1.2104140000	-3.6582800000	-0.5639810000
C	0	4.4599670000	0.9952350000	0.2918740000
H	0	3.6363030000	2.6160780000	1.4835930000
H	0	4.9750090000	-0.8209510000	-0.7286110000
H	0	3.3989210000	-2.6618690000	-1.1517780000
H	0	5.4865980000	1.3639670000	0.3404600000
C	0	-0.7292950000	0.6241590000	-0.1366560000
C	0	-2.1533100000	0.4860670000	-0.1351910000
C	0	-0.2983050000	1.9405070000	-0.4555900000
C	0	-2.9747100000	1.6280140000	-0.1312380000
C	0	-1.1185190000	3.0566200000	-0.4877330000
H	0	0.7437890000	2.1001060000	-0.7123430000
C	0	-2.4801580000	2.9165740000	-0.2433730000
H	0	-4.0561160000	1.4754320000	-0.1083570000
H	0	-0.6860900000	4.0313490000	-0.7229010000

H	0	-3.1539630000	3.7752870000	-0.2379670000
S	0	-3.0925880000	-1.0021490000	-0.4806870000
C	0	-4.0321170000	-1.2413960000	1.0495620000
H	0	-4.7249340000	-2.0732770000	0.8618580000
H	0	-3.3779610000	-1.5052530000	1.8933420000
H	0	-4.6157820000	-0.3462450000	1.3051260000
C	0	-0.8317850000	-2.6401170000	0.6199530000
H	0	-1.4681930000	-2.0626110000	1.2945830000
H	0	-1.4666550000	-3.1414150000	-0.1208730000
H	0	-0.3389290000	-3.4186540000	1.2241500000

TS1b-trans

0 1

C	0	0.3439550000	-0.2190880000	0.0737980000
C	0	1.7383460000	0.2075160000	0.0715580000
C	0	0.1498860000	-1.6027160000	-0.0003080000
C	0	2.1887190000	1.4045620000	0.7074360000
C	0	2.7500470000	-0.6470930000	-0.4611550000
C	0	1.1669700000	-2.4436010000	-0.5298770000
C	0	3.5107850000	1.7796740000	0.6967280000
H	0	1.4786400000	2.0128910000	1.2665240000
C	0	4.1013890000	-0.2055930000	-0.5185620000
C	0	2.4071030000	-1.9666860000	-0.8492070000
H	0	0.9357090000	-3.5026990000	-0.6765420000
C	0	4.4786790000	0.9914150000	0.0335390000
H	0	3.8157110000	2.6869210000	1.2225010000

H	0	4.8430090000	-0.8662520000	-0.9741530000
H	0	3.1610010000	-2.6138120000	-1.3025230000
H	0	5.5220970000	1.3117480000	0.0044200000
C	0	-0.7405860000	0.8267710000	0.0422460000
C	0	-2.1412680000	0.6491130000	0.2127920000
C	0	-0.3659580000	2.1331450000	-0.3526330000
C	0	-3.0100680000	1.7489790000	0.2262360000
C	0	-1.2357560000	3.2156700000	-0.3750380000
H	0	0.6525550000	2.3136000000	-0.6847730000
C	0	-2.5692230000	3.0410890000	-0.0263050000
H	0	-4.0705860000	1.5585570000	0.4015090000
H	0	-0.8602610000	4.1948970000	-0.6789490000
H	0	-3.2689210000	3.8786340000	-0.0109010000
S	0	-2.9976730000	-0.9180160000	0.2209910000
C	0	-3.0074580000	-1.2328110000	-1.5665120000
H	0	-3.5578550000	-2.1714190000	-1.7189600000
H	0	-3.5173130000	-0.4192560000	-2.0994760000
H	0	-1.9821980000	-1.3467020000	-1.9488970000
C	0	-0.8900720000	-2.4582630000	0.6929030000
O	0	-0.8759430000	-2.5620560000	1.8943520000
H	0	-1.4342080000	-3.1848990000	0.0437270000

TS1c-cis

0 1

C	-1.6167499136	0.8281925057	-0.0045393833
C	-2.1811447359	2.1136529737	0.1876724509

C	-3.5524830572	2.2767171723	0.3058847644
C	-4.4315803125	1.1832061252	0.2447749016
C	-3.9310540236	-0.0918681096	0.0627680911
C	-2.5427674734	-0.2435553170	-0.0617950103
C	-0.2752185550	0.2420095703	-0.1603733153
C	-0.5014121259	-1.1293621818	-0.2882472188
N	-1.8461907950	-1.4082880664	-0.2439936605
H	-1.5877294323	3.0192504836	0.2581111046
H	-3.9525243071	3.2817475688	0.4549906927
H	-5.5074511693	1.3380484489	0.3476592757
H	-4.5991563072	-0.9532828421	0.0282938668
C	0.3269295416	-2.3881624858	-0.4045699049
O	0.4126571089	-3.1526072071	0.5347512339
H	0.5934560839	-2.6984329129	-1.4364905887
C	-2.4550675580	-2.7217937564	-0.3037048428
H	-1.8985950594	-3.3763106817	-0.9844703112
H	-2.4870378596	-3.1957147099	0.6880081943
H	-3.4742634977	-2.6276025327	-0.6957863255
C	1.0152983675	0.9908225147	-0.1513210788
C	0.9809688307	2.3933783121	-0.0037856334
C	2.3212648221	0.4476079234	-0.2692000486
C	2.1111013235	3.1984043887	0.0528707214
H	0.0284834329	2.8942918849	0.0683379437
C	3.4616603809	1.2615747410	-0.2180239663
C	3.3778672277	2.6352664473	-0.0453963168
H	1.9890941445	4.2771948384	0.1710992958
H	4.4374943191	0.7838857997	-0.3296220826
H	4.2797214807	3.2486444287	-0.0071460294

S	2.6789831012	-1.2700506462	-0.5549780527
C	3.0473173125	-1.8520146682	1.1372330411
H	3.7487955303	-1.1346316516	1.5868262588
H	2.1081438391	-1.8421021177	1.7105151966
C	3.6281099659	-3.2556338047	1.0840029802
H	4.5546033733	-3.2875268232	0.4911575457
H	3.8601859731	-3.5979113169	2.1032775119
H	2.9024148729	-3.9593092755	0.6517421686

TS2a

0 1

C	0	-1.1296620000	-0.7435330000	-0.0418070000
C	0	-0.9511520000	-2.1452060000	0.0579250000
C	0	-2.0318170000	-3.0044150000	0.0645120000
C	0	-3.3537170000	-2.5254760000	-0.0279330000
C	0	-3.5895600000	-1.1701910000	-0.0960620000
C	0	-2.4782540000	-0.3058350000	-0.0753600000
C	0	-0.2779560000	0.4461980000	-0.0529740000
C	0	-1.1977770000	1.5327100000	0.0220180000
N	0	-2.4997750000	1.0500070000	-0.0479610000
H	0	0.0423590000	-2.5639950000	0.1102880000
H	0	-1.8526040000	-4.0784340000	0.1448870000
H	0	-4.1913370000	-3.2261550000	-0.0258830000
H	0	-4.6071470000	-0.7797360000	-0.1299530000
C	0	-1.2034620000	2.9703020000	0.4175780000
O	0	-0.3844260000	3.8504290000	0.2954090000

H	0	-2.1580870000	3.2257620000	0.9275320000
C	0	-3.7176290000	1.8251680000	-0.1906600000
H	0	-4.1890050000	2.0402550000	0.7810110000
H	0	-3.5229660000	2.7670640000	-0.7161540000
H	0	-4.4274800000	1.2579800000	-0.8047710000
C	0	1.2237820000	0.6013480000	-0.1143700000
C	0	1.6933190000	1.9337040000	-0.1026750000
C	0	2.2646820000	-0.3752070000	-0.2129910000
C	0	3.0276470000	2.3096700000	-0.0585250000
H	0	0.9808250000	2.7463830000	-0.1344650000
C	0	3.6146950000	0.0195960000	-0.1695500000
C	0	4.0193710000	1.3398730000	-0.0596530000
H	0	3.2761630000	3.3729710000	-0.0353250000
H	0	4.3692480000	-0.7649160000	-0.2538540000
H	0	5.0798410000	1.5963250000	-0.0233790000
S	0	2.1496840000	-2.1322350000	-0.5575260000
C	0	2.4149450000	-2.8228620000	1.1010890000
H	0	2.4702950000	-3.9142090000	0.9841900000
H	0	3.3598260000	-2.4592240000	1.5279720000
H	0	1.5842360000	-2.5728640000	1.7774010000

TS2a-Me

0 1

C	0	-1.1696860000	-0.4858330000	-0.1930450000
C	0	-1.1907020000	-1.8539550000	-0.5401430000
C	0	-2.3821130000	-2.5624090000	-0.5729510000

C	0	-3.6109740000	-1.9460690000	-0.2873090000
C	0	-3.6496650000	-0.5924590000	-0.0035110000
C	0	-2.4378490000	0.1114730000	0.0165820000
C	0	-0.1648210000	0.5842660000	-0.0878250000
C	0	-0.9248960000	1.7633000000	0.0359400000
N	0	-2.2585510000	1.4617960000	0.1685980000
H	0	-0.2696910000	-2.3634300000	-0.8025200000
H	0	-2.3596100000	-3.6202280000	-0.8447330000
H	0	-4.5376050000	-2.5232070000	-0.3194940000
H	0	-4.6009240000	-0.0866910000	0.1685900000
C	0	-3.3379020000	2.3997680000	0.3793270000
H	0	-3.0121030000	3.2276650000	1.0201800000
H	0	-3.7247530000	2.8075520000	-0.5684690000
H	0	-4.1584450000	1.8910220000	0.8989480000
C	0	1.3370110000	0.5741270000	-0.0858880000
C	0	1.9916260000	1.8148260000	0.1269830000
C	0	2.2479040000	-0.5182790000	-0.2342920000
C	0	3.3533010000	2.0322170000	-0.0265950000
H	0	1.4155760000	2.6685070000	0.4503580000
C	0	3.6233320000	-0.2879150000	-0.4140500000
C	0	4.1890100000	0.9764410000	-0.3674950000
H	0	3.7534890000	3.0355420000	0.1358170000
H	0	4.2649080000	-1.1608050000	-0.5497030000
H	0	5.2609890000	1.1155900000	-0.5181750000
S	0	1.9109320000	-2.2679550000	-0.0792730000
C	0	1.2723590000	-2.3406500000	1.6152360000
H	0	1.1037020000	-3.4033460000	1.8381770000
H	0	2.0098010000	-1.9332310000	2.3202110000

H	0	0.3191170000	-1.8025350000	1.7087840000
C	0	-0.5931010000	3.2236690000	-0.0962030000
H	0	-0.2529910000	3.6843600000	0.8451600000
H	0	0.1831560000	3.3814180000	-0.8537960000
H	0	-1.4766820000	3.7814130000	-0.4297280000

TS2b

0 1

C	0	-0.3031840000	0.7720910000	-0.0154560000
C	0	-1.4520220000	-0.1121490000	0.0006940000
C	0	-0.6366630000	2.1383010000	-0.1453110000
C	0	-1.4624130000	-1.4025460000	-0.5948100000
C	0	-2.7094510000	0.3783470000	0.4796270000
C	0	-1.9025450000	2.6201200000	0.2805250000
C	0	-2.5720650000	-2.2120860000	-0.5554420000
H	0	-0.6049320000	-1.7105920000	-1.1799730000
C	0	-3.8151290000	-0.5090690000	0.5968540000
C	0	-2.8804970000	1.7671410000	0.7109120000
H	0	-2.0785950000	3.6992390000	0.2611970000
C	0	-3.7450530000	-1.7894040000	0.1088720000
H	0	-2.5496420000	-3.1812390000	-1.0581000000
H	0	-4.7444040000	-0.1266050000	1.0256720000
H	0	-3.8272040000	2.1397880000	1.1071240000
H	0	-4.6090780000	-2.4540460000	0.1735120000
C	0	1.1481010000	0.4108830000	0.2154000000
C	0	1.8269510000	-0.8471100000	0.2541520000

C	0	1.9639410000	1.5133040000	0.5788730000
C	0	3.2263740000	-0.8831270000	0.3810370000
C	0	3.3402080000	1.4657750000	0.7153680000
H	0	1.4870290000	2.4642040000	0.7986620000
C	0	4.0003180000	0.2532950000	0.5543940000
H	0	3.7086340000	-1.8628000000	0.3953190000
H	0	3.8866720000	2.3752000000	0.9717450000
H	0	5.0850960000	0.1768960000	0.6482560000
S	0	1.1096530000	-2.4839090000	0.4394830000
C	0	1.5124300000	-3.2473170000	-1.1545050000
H	0	1.2081250000	-4.3005070000	-1.0814680000
H	0	0.9675270000	-2.7740980000	-1.9853750000
H	0	2.5913880000	-3.2008130000	-1.3559750000
C	0	0.1445730000	3.1554220000	-0.9120860000
O	0	0.9783560000	2.9330430000	-1.7527610000
H	0	-0.2051430000	4.2023530000	-0.7248110000

TS2b-Me

0 1

C	0	0.3276200000	0.8917560000	0.1447470000
C	0	1.4106120000	-0.0806060000	0.0365020000
C	0	0.7538140000	2.2070350000	0.4009240000
C	0	1.3270220000	-1.4056440000	0.5349060000
C	0	2.6996970000	0.3392080000	-0.4091750000
C	0	2.0713220000	2.6058510000	0.0269110000
C	0	2.3674240000	-2.3000730000	0.4165530000

H	0	0.4533280000	-1.6906260000	1.1076100000
C	0	3.7341630000	-0.6141240000	-0.6034680000
C	0	2.9776190000	1.7300770000	-0.4947660000
H	0	2.3336940000	3.6614490000	0.1386120000
C	0	3.5666330000	-1.9237950000	-0.2226830000
H	0	2.2658290000	-3.3015630000	0.8406810000
H	0	4.6910120000	-0.2684400000	-1.0029480000
H	0	3.9524350000	2.0680950000	-0.8537260000
H	0	4.3754190000	-2.6467340000	-0.3485380000
C	0	-1.1499070000	0.6237590000	-0.0794100000
C	0	-1.9164500000	-0.5819670000	-0.1649920000
C	0	-1.9044440000	1.7900430000	-0.3758290000
C	0	-3.3202210000	-0.5183030000	-0.2392200000
C	0	-3.2854020000	1.8401140000	-0.4727170000
H	0	-1.3709340000	2.7124750000	-0.5760230000
C	0	-4.0241140000	0.6710670000	-0.3320210000
H	0	-3.8663750000	-1.4631460000	-0.2844390000
H	0	-3.7743590000	2.7935110000	-0.6835160000
H	0	-5.1142330000	0.6706790000	-0.3869990000
S	0	-1.3273710000	-2.2469380000	-0.5060980000
C	0	-1.7260580000	-3.1118160000	1.0354660000
H	0	-1.5144310000	-4.1756950000	0.8603550000
H	0	-1.1088150000	-2.7595800000	1.8760770000
H	0	-2.7881020000	-2.9979630000	1.2932270000
C	0	-0.0247660000	3.2780400000	1.1414150000
H	0	0.6404920000	3.6900360000	1.9163840000
H	0	-0.3234560000	4.1240560000	0.5022230000
H	0	-0.9209440000	2.8907140000	1.6397370000

7. References

(1) Gore, V.; Patel, P.; Chang, C.-T.; Sivendran, S.; Kang, N.; Ouedraogo, Y.P.; Gravel, S.; Powell, W.S.; Rokach, J. 5-Oxo-ETE Receptor Antagonists. *J. Med. Chem.* **2013**, *56*, 3725-3732.

(2) (a) Mora-Radó, H.; Sotorríos, L.; Ball-Jones, M.P.; Bialy, L.; Czechtizky, W.; Méndez, M.; Gómez-Bengoa, E.; Harrity, J.P.A. Synthetic and Mechanistic Investigation of an Oxime Ether Electrocyclization Approach to Heteroaromatic Boronic Acid Derivatives. *Chem. Eur. J.* **2018**, *24*, 9530-9534. (b) Nishiyama, T.; Satsuki, N.; Hibino, S.; Fujii, M.; Abe, T.; Choshi, T. Total Synthesis of Carbazole-1,4-quinone Alkaloid Koeniginequinones A and B Based on a One-Pot Cyclocarbonylation Procedure from 2-Alkenyl-3-iodoiodoindole. *Heterocycles*. **2016**, *93*, 84-100. (c) Biswas, S.; Batra, S. Copper-Catalyzed Synthesis of Indole-Fused Benzodiazepines. *Adv. Synth. Catal.* **2011**, *353*, 2861-2867. (d) Zhang, H.; Larock, R.C. Synthesis of β- and γ-Carbolines by the Palladium-Catalyzed Iminoannulation of Alkynes. *J. Org. Chem.* **2002**, *67*, 9318-9330. (e) Nishiyama, T.; Hatae, N.; Yoshimura, T.; Takaki, S.; Abe, T.; Ishikura, M.; Hibino, S.; Choshi, T. Concise synthesis of carbazole-1,4-quinones and evaluation of their antiproliferative activity against HCT-116 and HL-60 cells. *Eur. J. Med. Chem.* **2016**, *121*, 561-577.

(3) Sato, K.; Isoda, M.; Tokura, Y.; Omura, K.; Tarui, A.; Omote, M.; Kumadaki, I.; Ando, A. Reductive aldol-type reaction of α,β-unsaturated esters with aldehydes or ketones in the presence of Rh catalyst and Et_2Zn. *Tetrahedron Letters*. **2013**, *54*, 5913-5915.

(4) Frisch, M.J.; Trucks, G.W.; Schlegel, H.B.; Scuseria, G.E.; Robb, M.A.; Cheeseman, J.R.; Scalmani, G.; Barone, V.; Petersson, G.A.; Nakatsuji, H.; Li, X.; Caricato, M.; Marenich, A.V.; Bloino, J.; Janesko, B.G.; Gomperts, R.; Mennucci, B.; Hratchian, H.P.; Ortiz, J.V.; Izmaylov, A.F.; Sonnenberg, J.L.; Williams; Ding, F.; Lipparini, F.; Egidi, F.; Goings, J.; Peng, B.; Petrone, A.; Henderson, T.; Ranasinghe, D.; Zakrzewski, V.G.; Gao, J.; Rega, N.; Zheng, G.; Liang, W.; Hada, M.; Ehara, M.; Toyota, K.; Fukuda, R.; Hasegawa, J.; Ishida, M.; Nakajima, T.; Honda, Y.; Kitao, O.; Nakai, H.; Vreven, T.; Throssell, K.; Montgomery Jr., J.A.; Peralta, J.E.; Ogliaro, F.; Bearpark, M.J.; Heyd, J.J.; Brothers, E.N.; Kudin, K.N.; Staroverov, V.N.; Keith, T.A.; Kobayashi, R.; Normand, J.; Raghavachari, K.; Rendell, A.P.; Burant, J.C.; Iyengar, S.S.; Tomasi, J.; Cossi, M.; Millam, J.M.; Klene, M.; Adamo, C.; Cammi, R.; Ochterski, J.W.; Martin, R.L.; Morokuma, K.; Farkas, O.; Foresman, J.B.; Fox, D.J. Gaussian, Inc., Wallingford, CT, 2016.

(5) Chai, J.-D.; Head-Gordon, M. Long-range corrected hybrid density functionals with damped atom–atom dispersion corrections. *Phys. Chem. Chem. Phys.* **2008**, *10*, 6615-6620.

(6) Weigend, F.; Ahlrichs, R. Balanced basis sets of split valence, triple zeta valence and quadruple zeta valence quality for H to Rn: Design and assessment of accuracy. *Phys. Chem. Chem. Phys.* **2005**, *7*, 3297-3305.

(7) Weigend, F. Accurate Coulomb-fitting basis sets for H to Rn. *Phys. Chem. Chem. Phys.* **2006**, *8*, 1057–1065.

(8) Marenich, A.V.; Cramer, C.J.; Truhlar, D.G. Universal Solvation Model Based on Solute Electron Density and on a Continuum Model of the Solvent Defined by the Bulk Dielectric Constant and Atomic Surface Tensions. *J. Phys. Chem. B.* **2009**, *113*, 6378-6396.

(9) Schlegel, H.B. Optimization of equilibrium geometries and transition structures. *J. Comput. Chem.* **1982**, *3*, 214-218.

(10) (a) González, C.; Schlegel, H.B. Reaction path following in mass-weighted internal coordinates. *J. Phys. Chem.* **1990**, *94*, 5523-5527. (b) González, C.; Schlegel, H.B. *J. Chem. Phys.* **1991**, *95*, 5853-5860. (c) Hratchian, H.P.; Schlegel, H.B. *J. Phys. Chem. A* **2002**, *106*, 165-169.

(11) Tanaka, R.; Yamashita, M.; Chung, L.W.; Morokuma, K.; Nozaki, K. Mechanistic Studies on the Reversible Hydrogenation of Carbon Dioxide Catalyzed by an Ir-PNP Complex. *Organometallics* **2011**, *30*, 6742-6750.

(12) Legault, C.Y. CYLview, 1.0b. *Université de Sherbrooke* **2009**, http://www.cylview.org (visited Dec 1st, 2021).

(13) Johnson, E.R.; Keinan, S.; Mori-Sanchez, P.; Contreras-Garcia, J.; Cohen, A.J.; Yang, W. Revealing Noncovalent Interactions. *J. Am. Chem. Soc.* **2010**, *132*, 6498-6506.

(14) Lane, J.R.; Contreras-Garcia, J.; Piquemal, J.-P.; Miller, B.J.; Kjaergaard, H.G. Are Bond Critical Points Really Critical for Hydrogen Bonding? *J. Chem. Theory Comput.* **2013**, *9*, 3263-3266.

(15) Boto, R.A.; Peccati, F.; Laplaza, R.; Quan, C.; Carbone, A.; Piquemal, J.-P.; Maday, Y.; Contreras-García, J. NCIPLOT4: Fast, Robust, and Quantitative Analysis of Noncovalent Interactions. *J. Chem. Theory Comput.* **2020**, *16*, 4150-4158.

(16) Humphrey, W.; Dalke, A.; Schulten, K. VMD: Visual molecular dynamics. *J. Mol. Graph.* **1996**, *14*, 33-38.

(17) Williams, T.; Kelley, C. Gnuplot 4.5: an interactive plotting program. **2011**, http://gnuplot.info (visited Dec 1st, 2021).

8. NMR Spectra and HPLC traces

Figure S9. ^{1}H-NMR (400 MHz, $CDCl_3$) of **8a**:

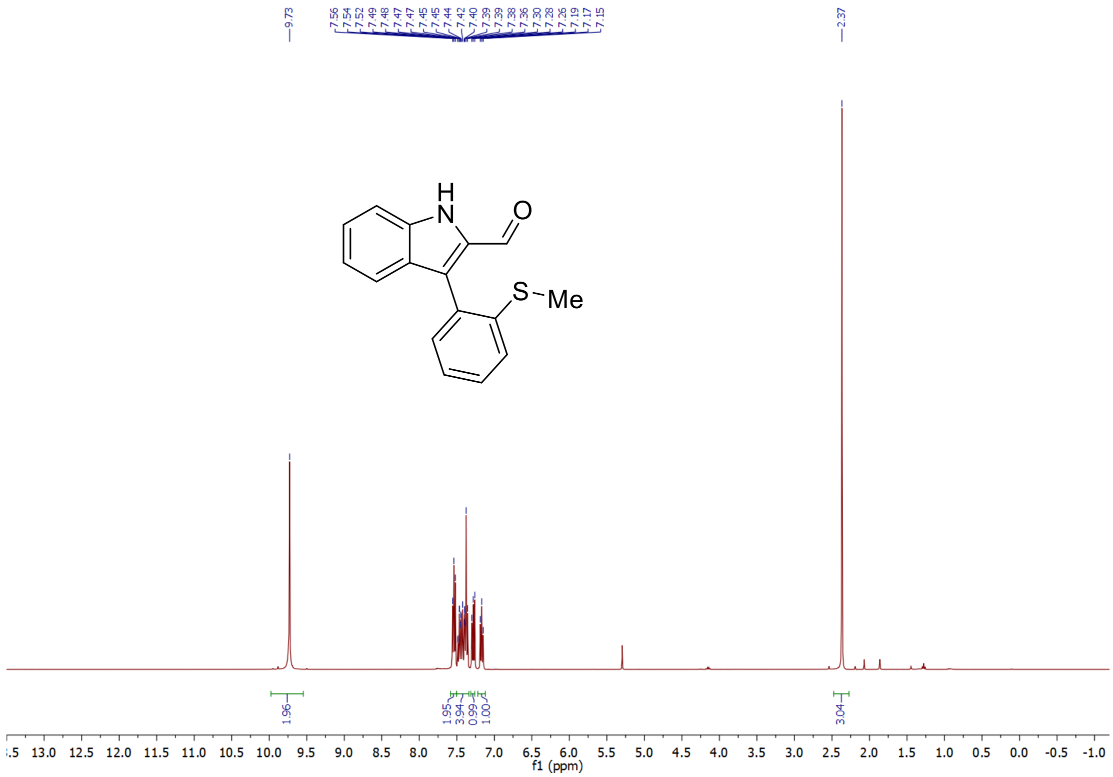

Figure S10. ^{13}C-NMR (100 MHz, $CDCl_3$) of **8a**:

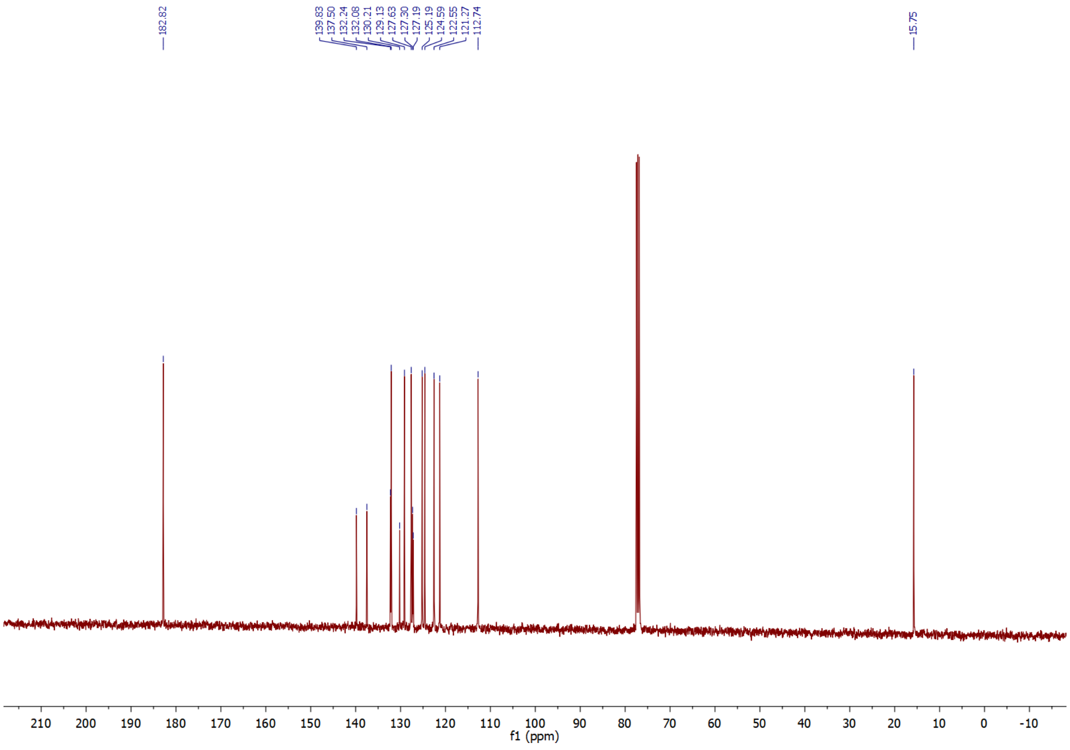

Figure S11. ^{1}H-NMR (400 MHz, $CDCl_3$) of **8g**:

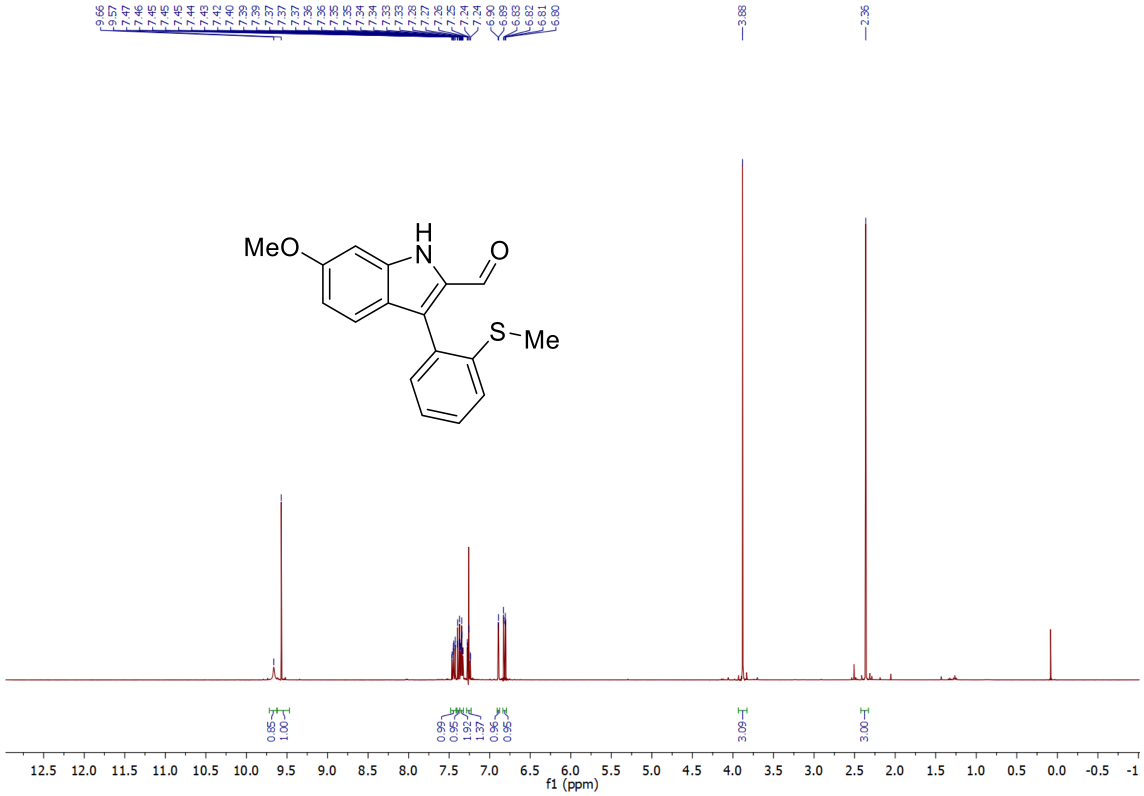

Figure S12. ^{13}C-NMR (100 MHz, $CDCl_3$) of **8g**:

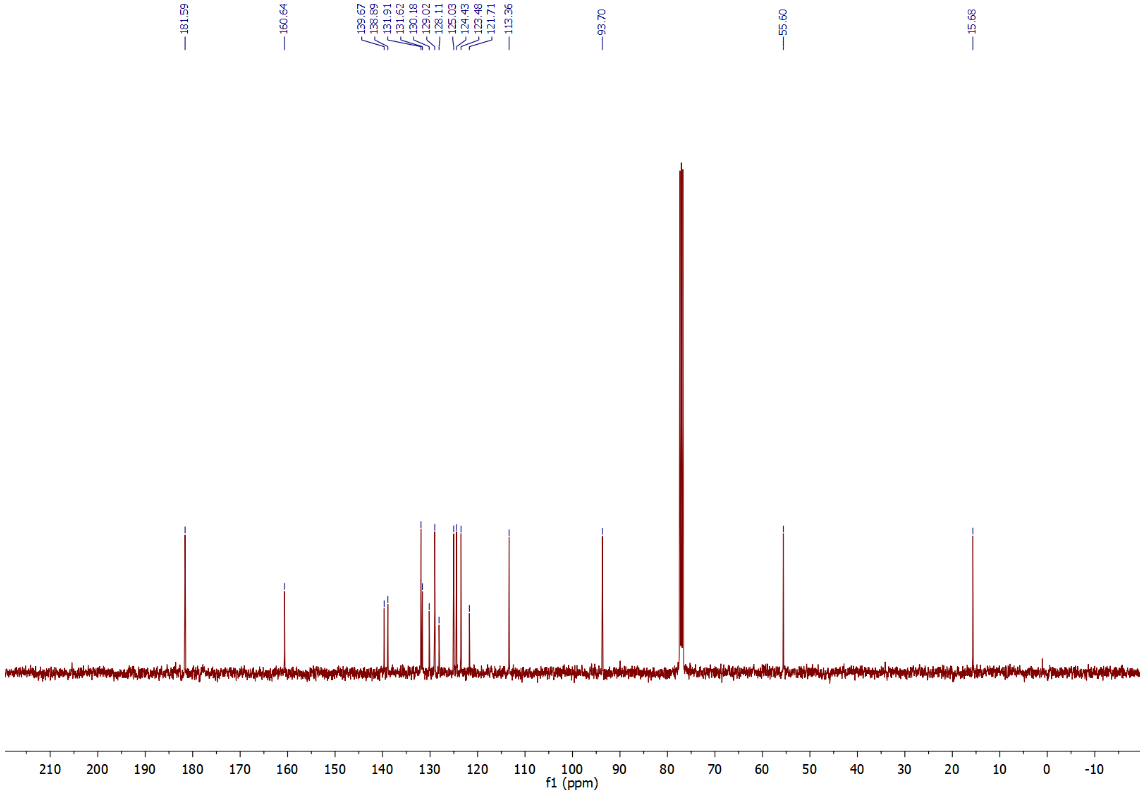

Figure S13. ^{1}H-NMR (400 MHz, DMSO-d_6) of **8h**:

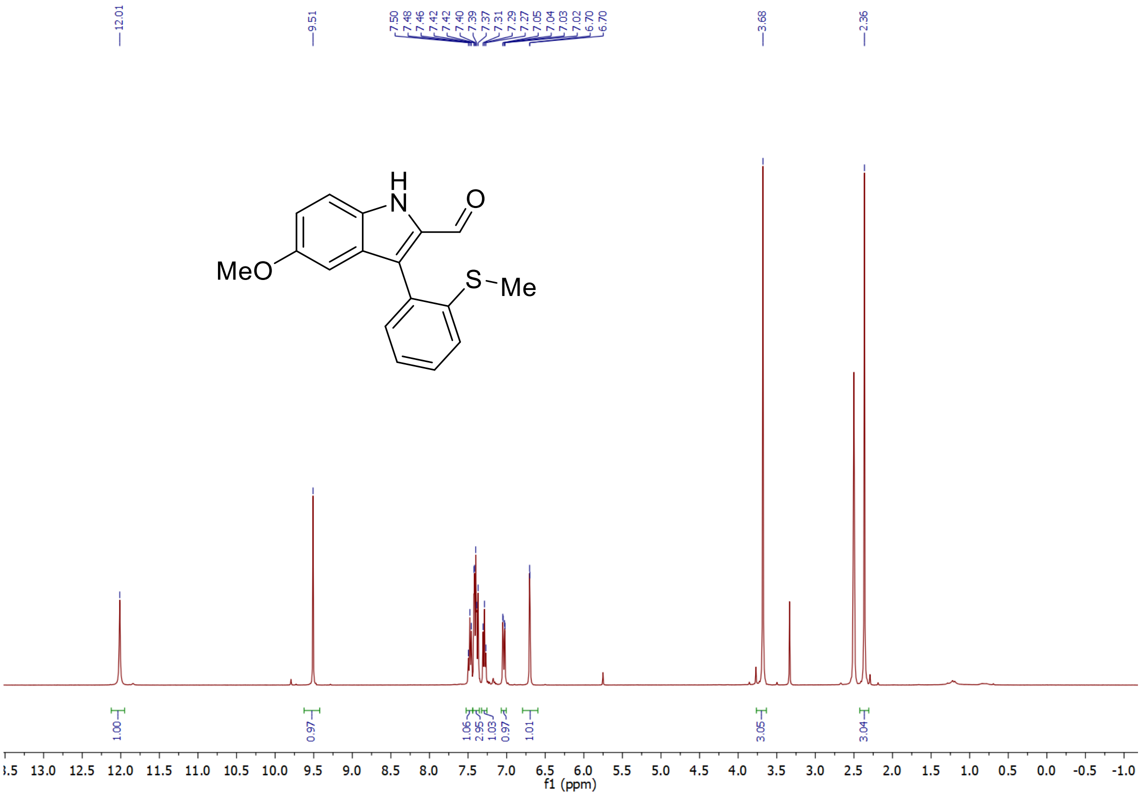

Figure S14. ^{13}C-NMR (100 MHz, DMSO-d_6) of **8h**:

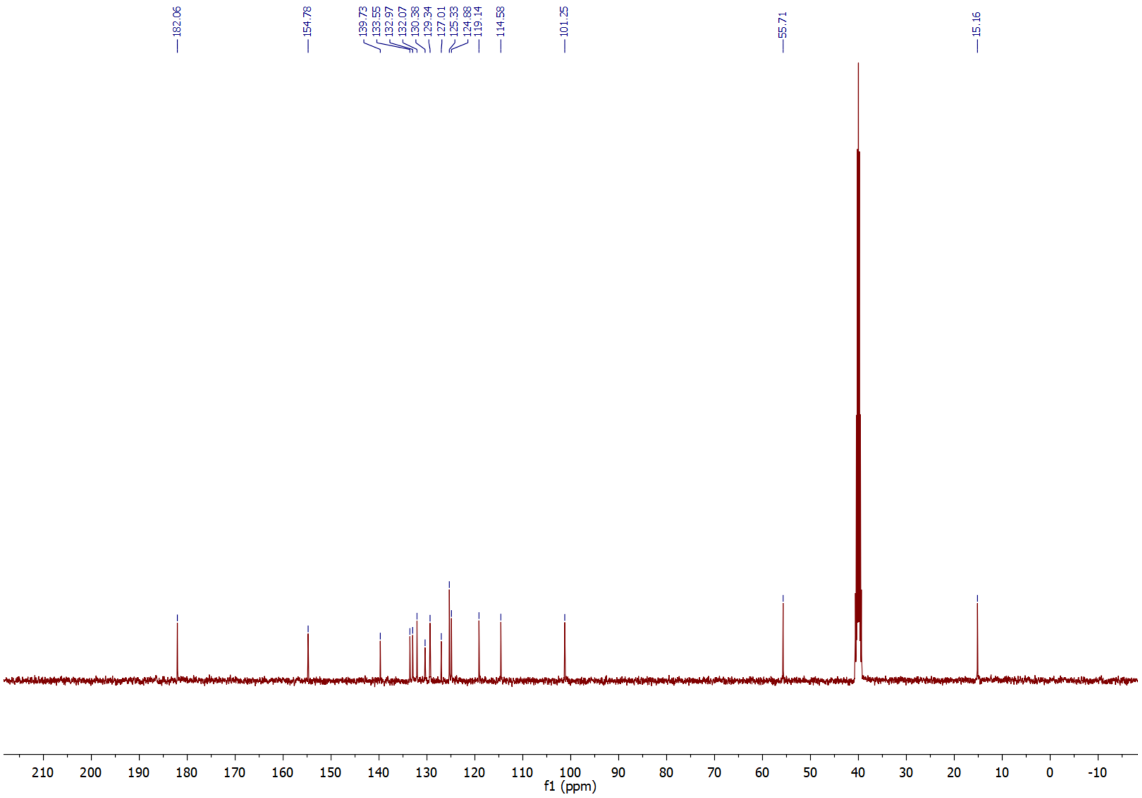

Figure S15. ^{1}H-NMR (400 MHz, $CDCl_3$) of **8i**:

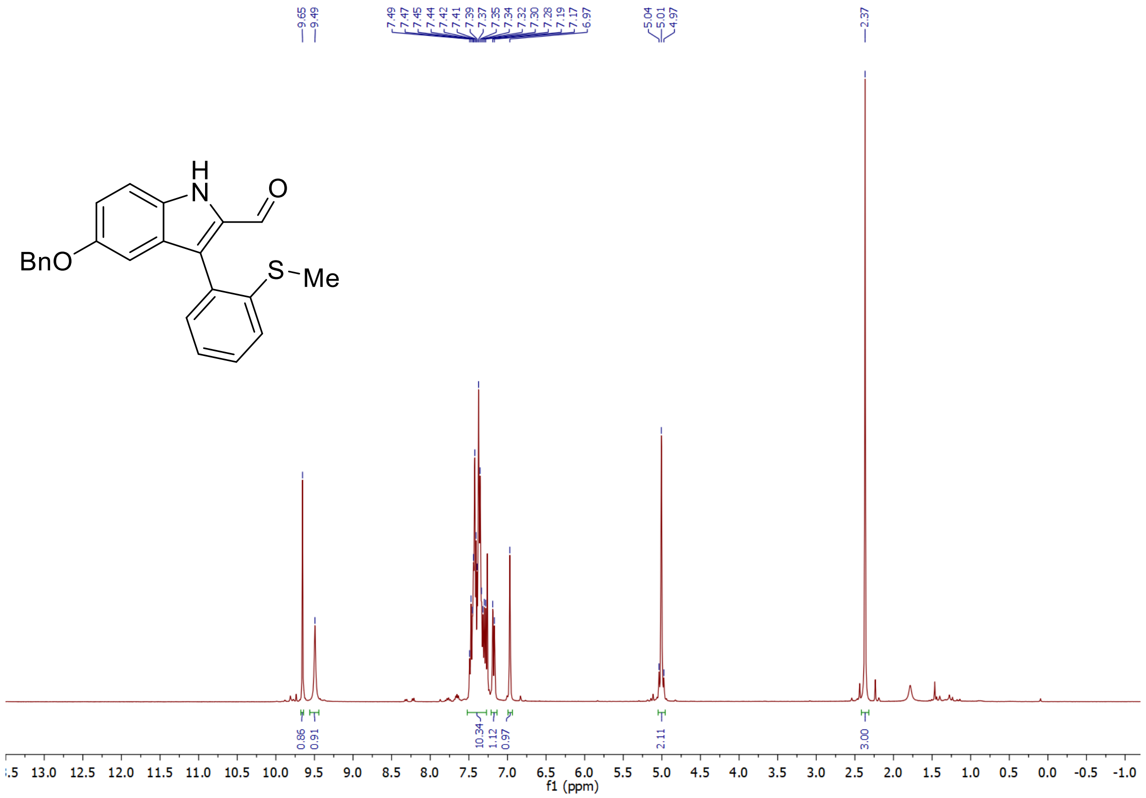

Figure S16. ^{13}C-NMR (100 MHz, $CDCl_3$) of **8i**:

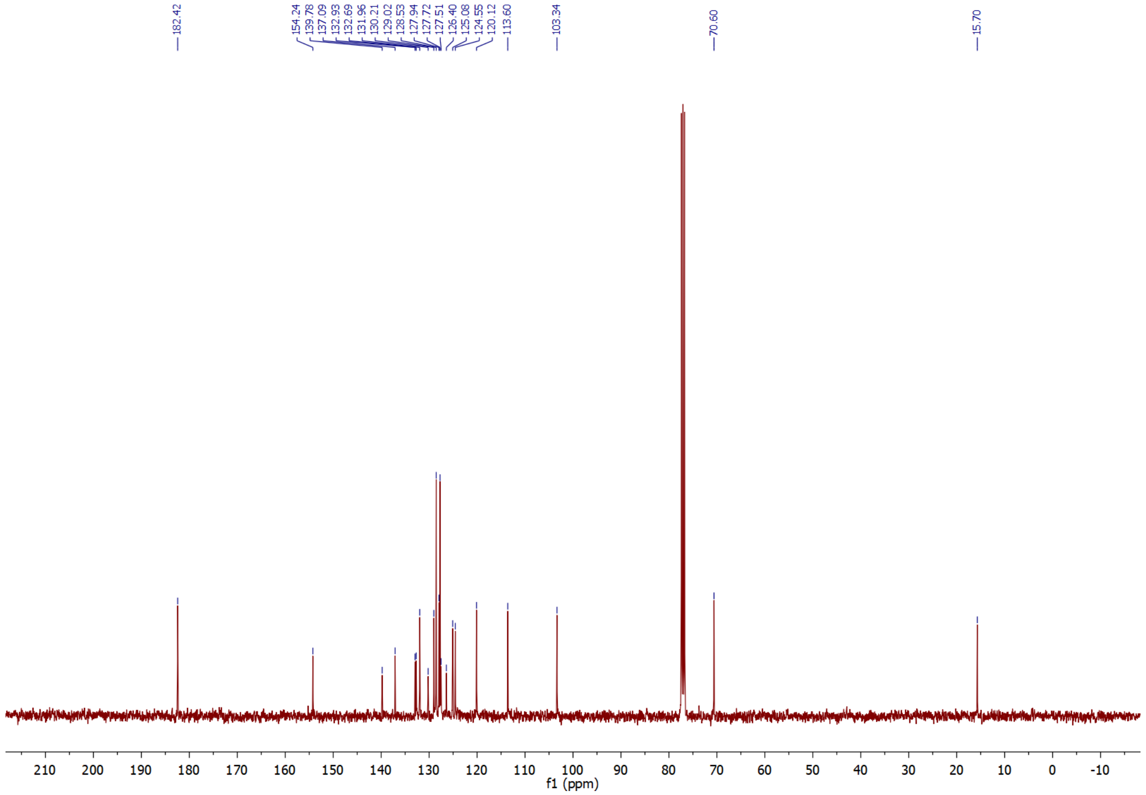

Figure S17. ^{1}H-NMR (400 MHz, DMSO-d_6) of **8j**:

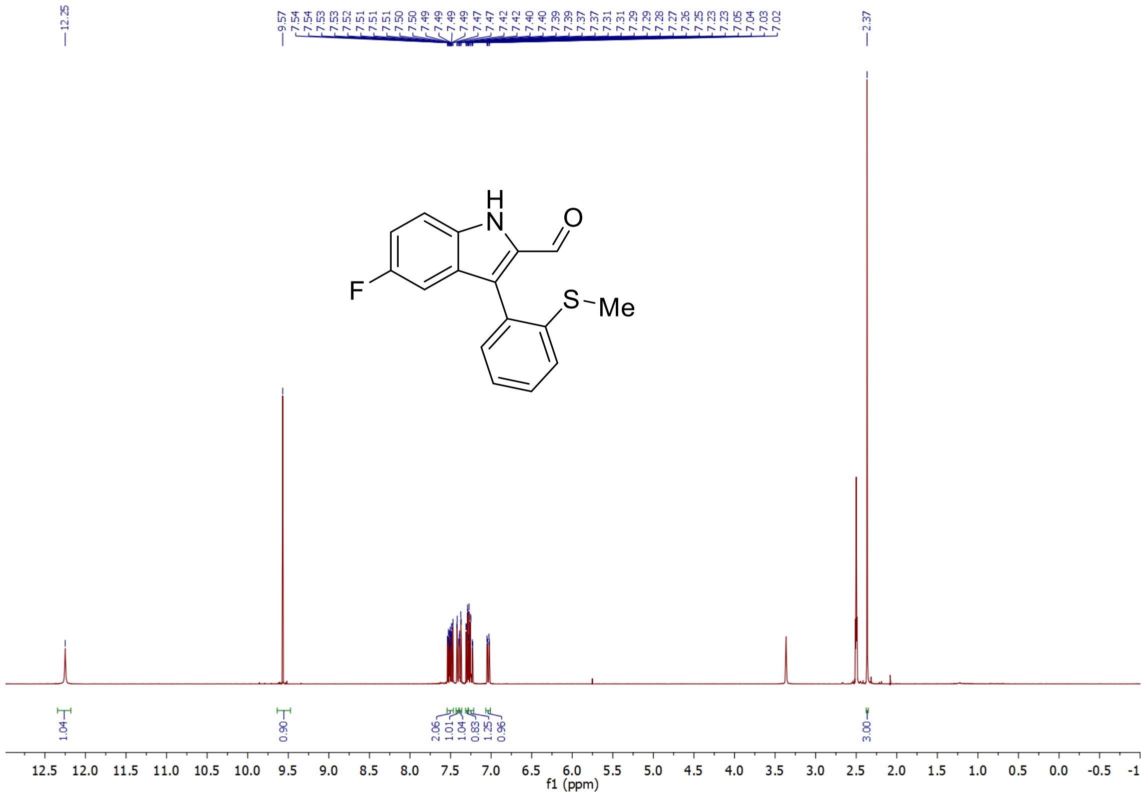

Figure S18. ^{13}C-NMR (100 MHz, DMSO-d_6) of **8j**:

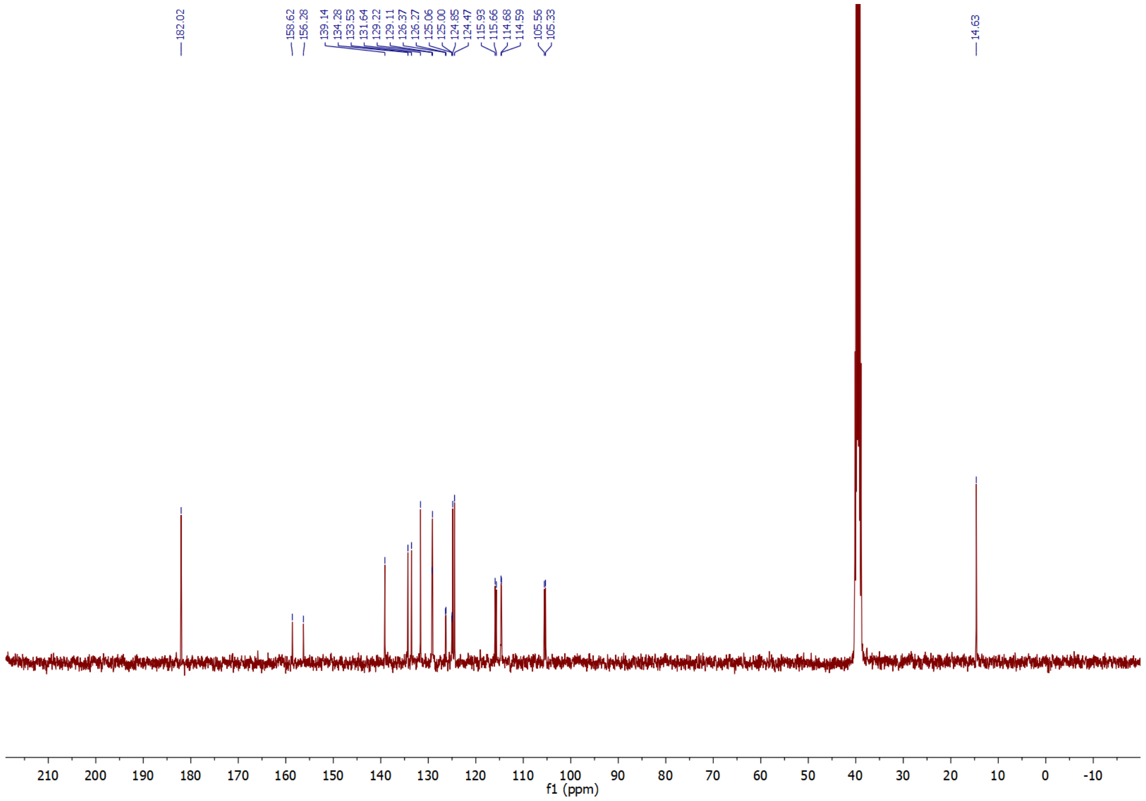

Figure S19. ^{19}F-NMR (376 MHz, DMSO-d_6) of **8j**:

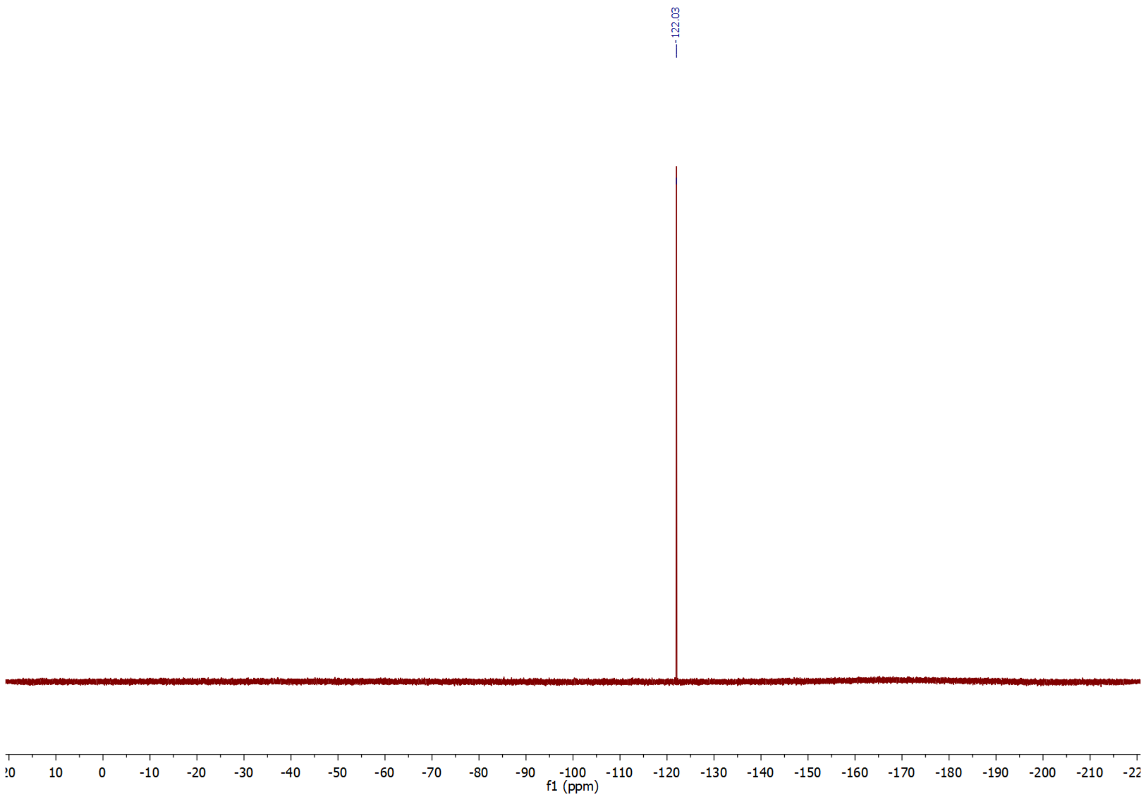

Figure S20. ^{1}H-NMR (400 MHz, $CDCl_3$) of **8k**:

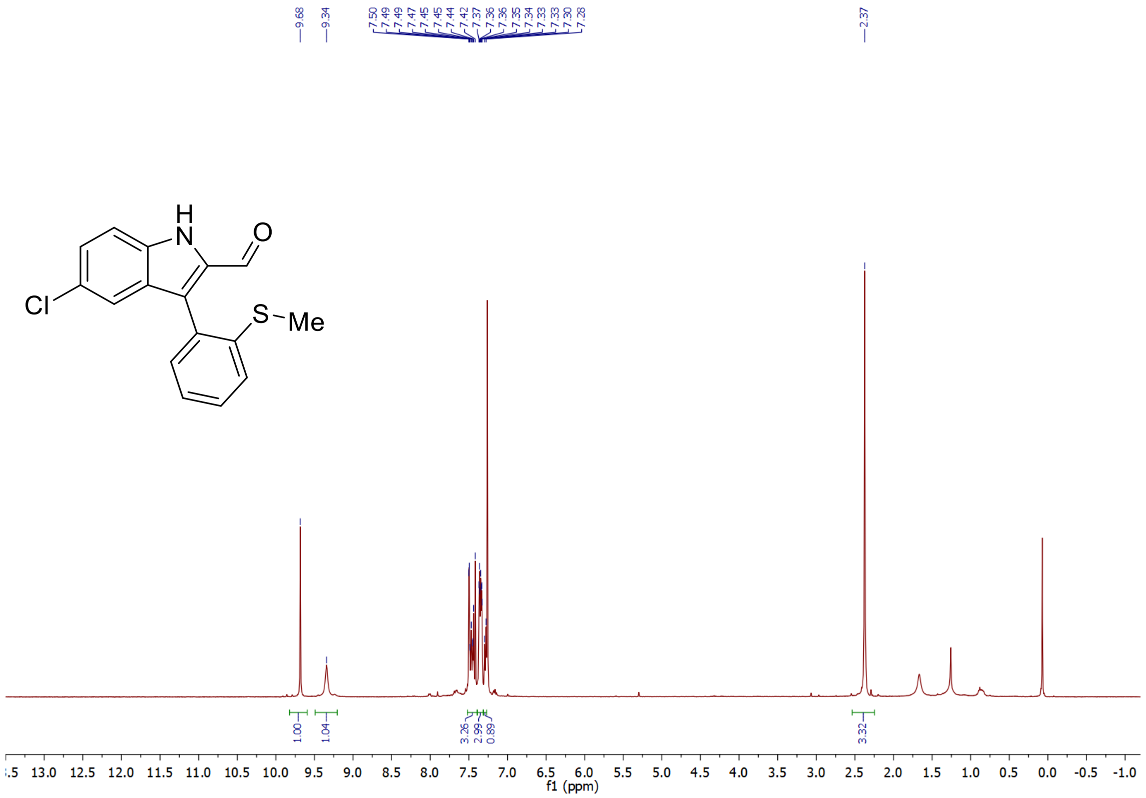

Figure S21. ^{13}C-NMR (100 MHz, $CDCl_3$) of **8k**:

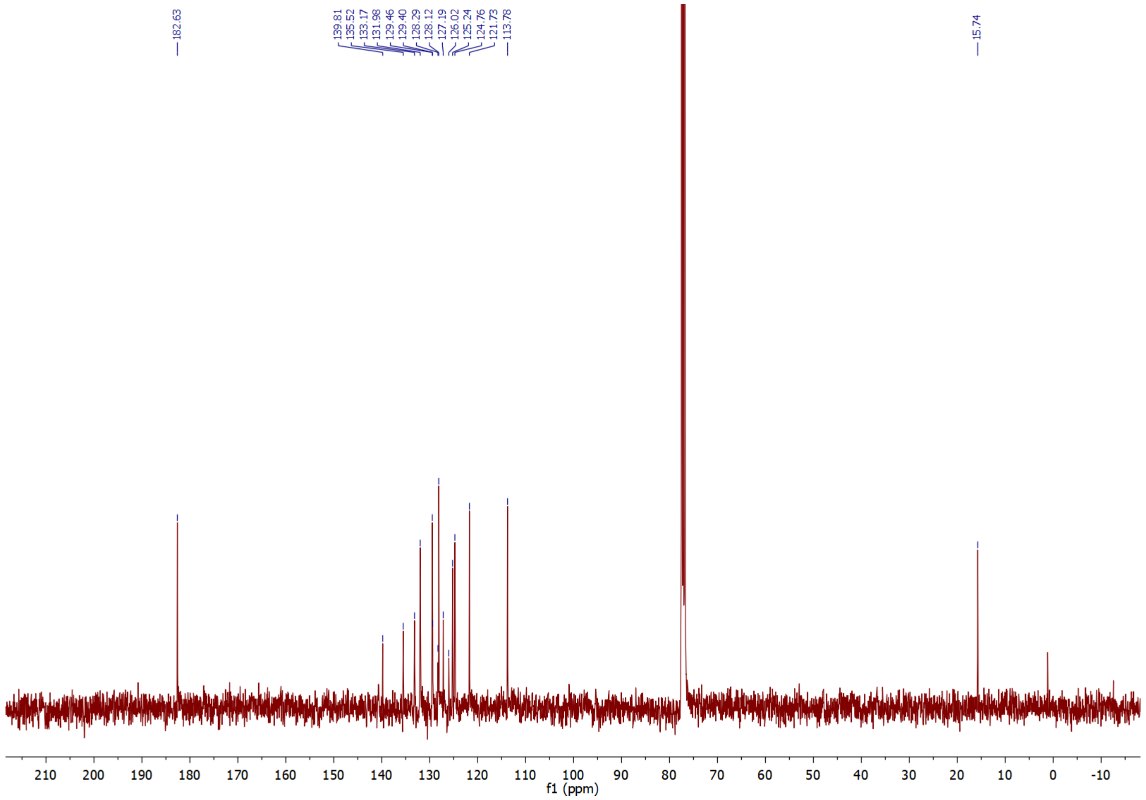

Figure S22. ^{1}H-NMR (400 MHz, DMSO-d_6) of **8l**:

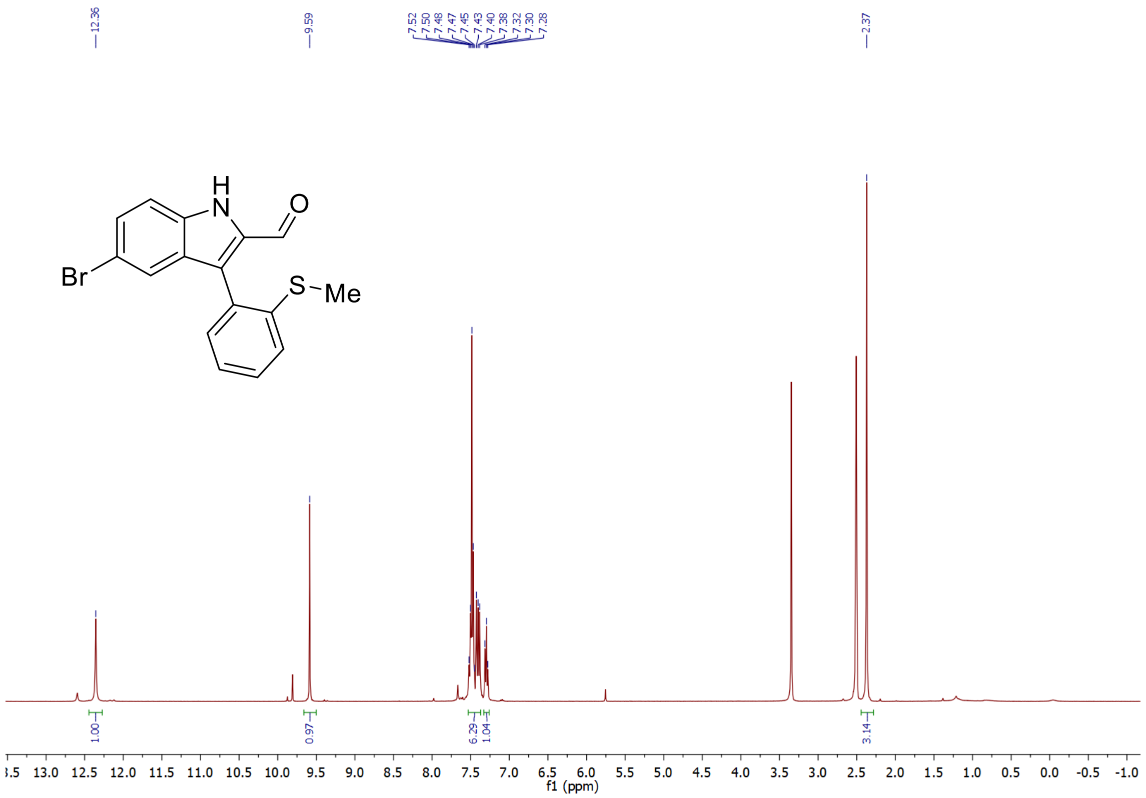

Figure S23. ^{13}C-NMR (100 MHz, DMSO-d_6) of **8l**:

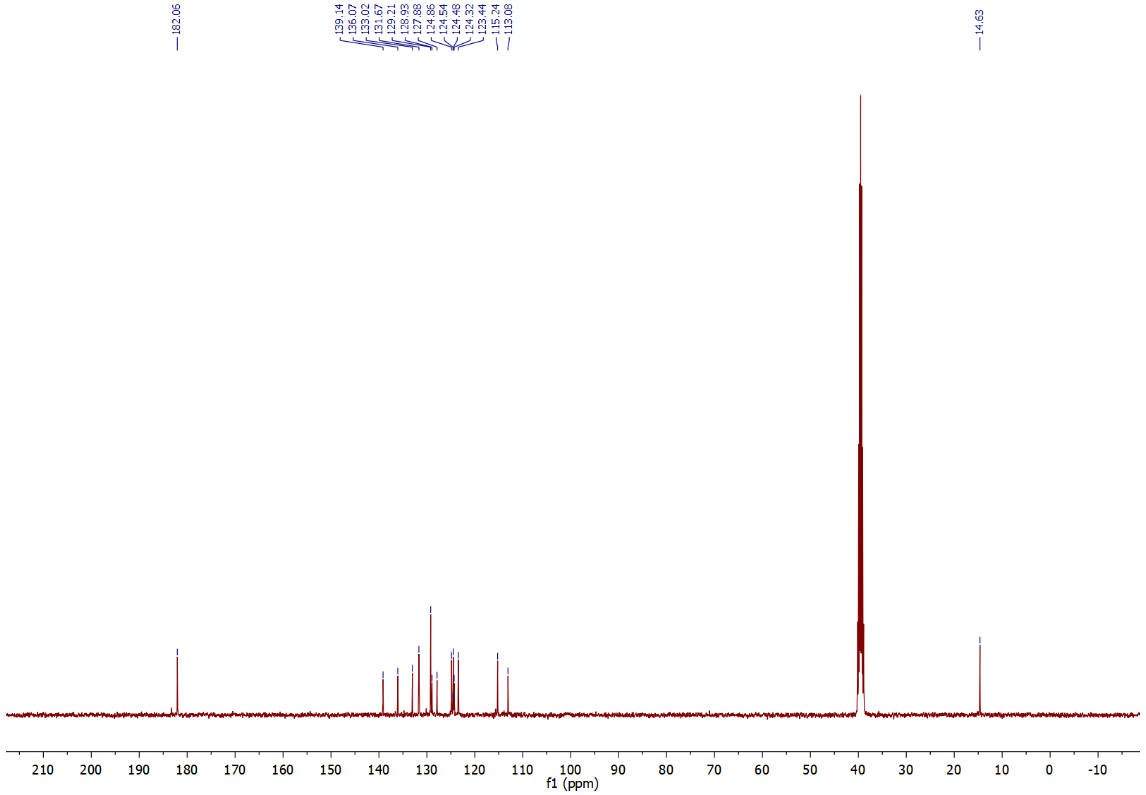

Figure S24. ^{1}H-NMR (400 MHz, $CDCl_3$) of **8m**:

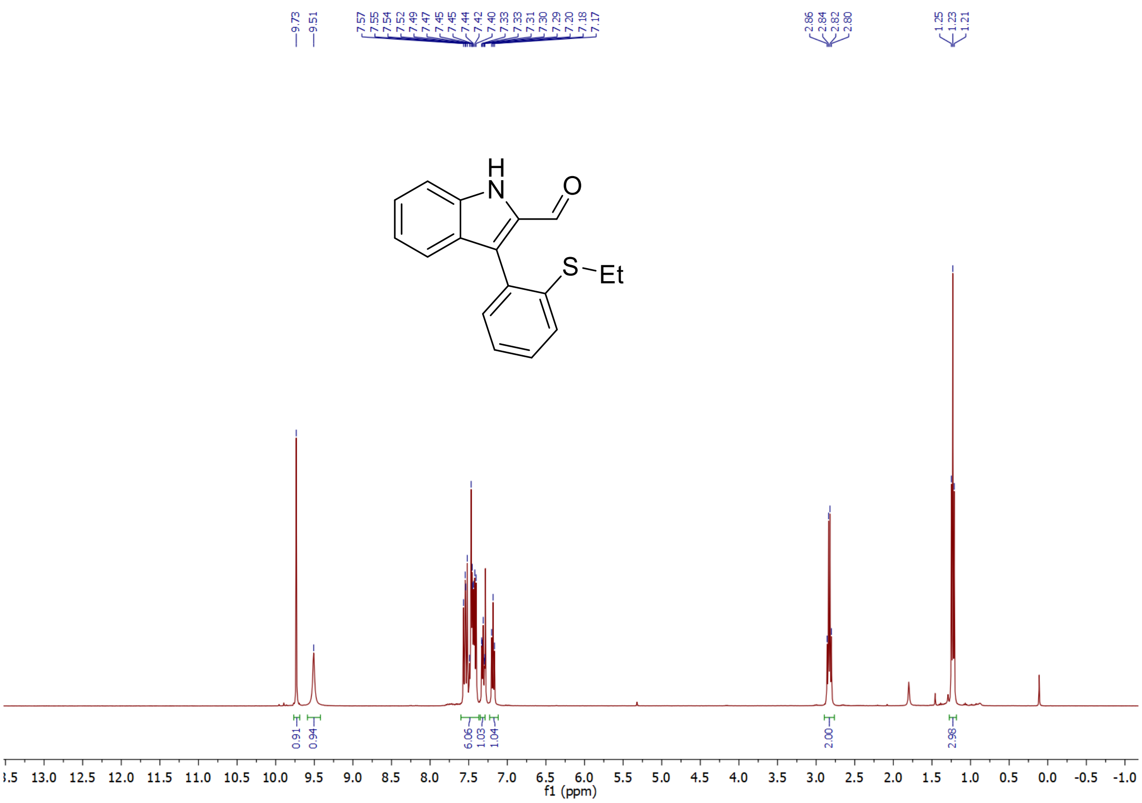

Figure S25. ^{13}C-NMR (100 MHz, $CDCl_3$) of **8m**:

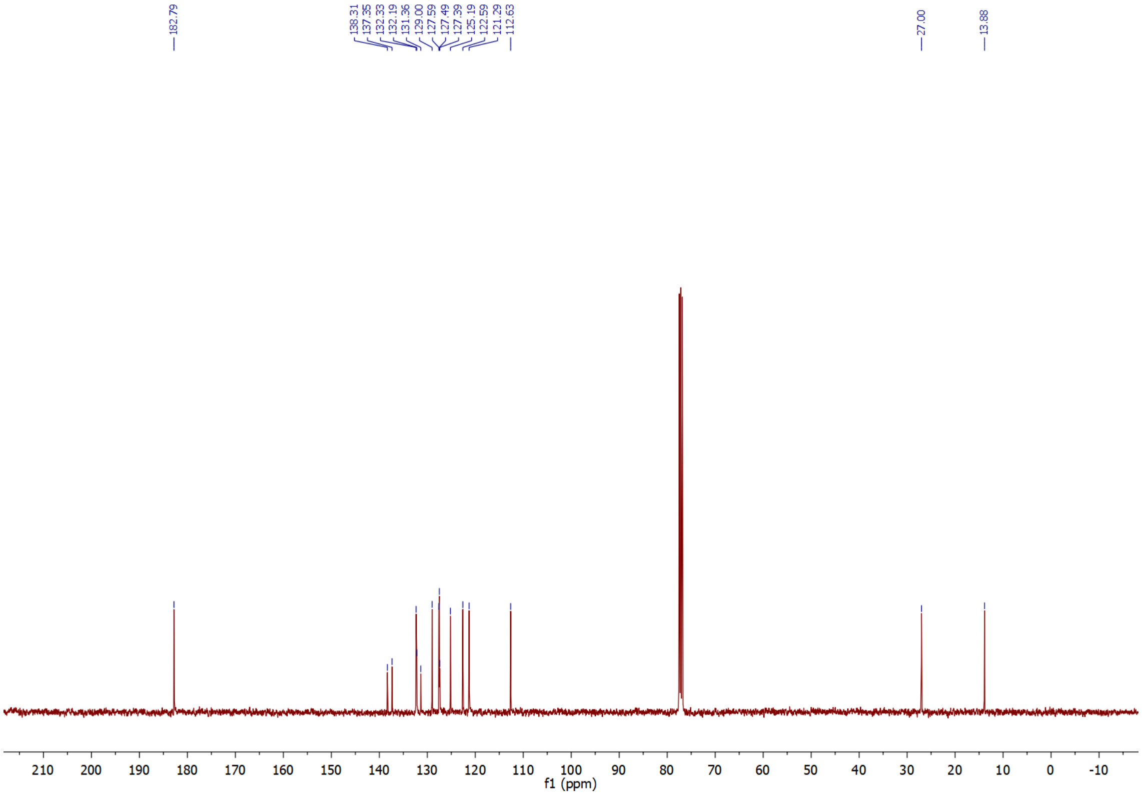

Figure S26. ^{1}H-NMR (400 MHz, $CDCl_3$) of **8t**:

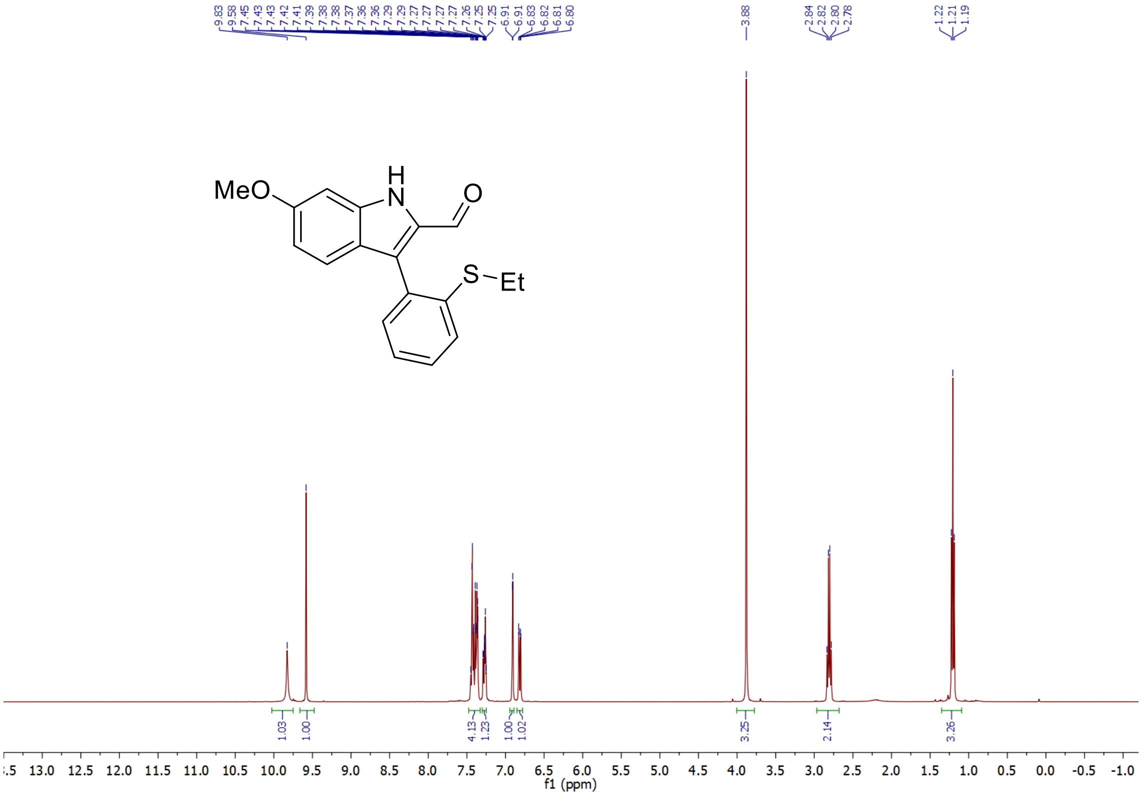

Figure S27. ^{13}C-NMR (100 MHz, $CDCl_3$) of **8t**:

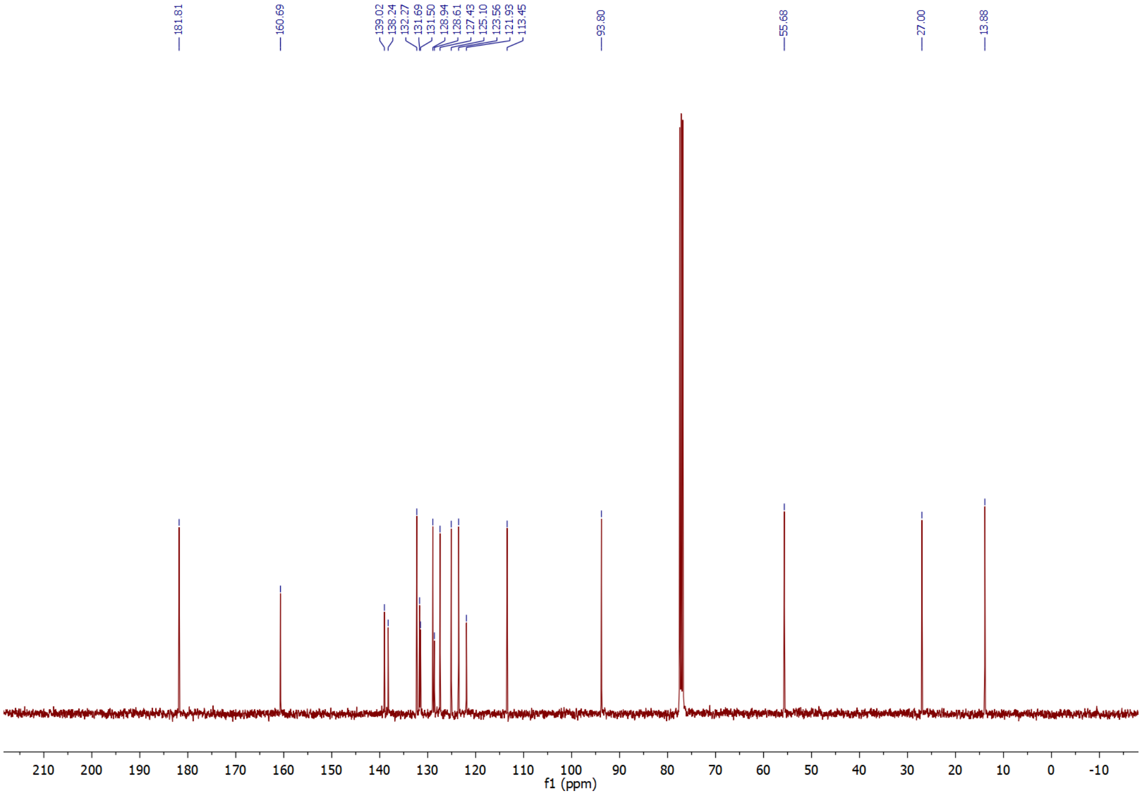

Figure S28. ^{1}H-NMR (400 MHz, $CDCl_3$) of **8u**:

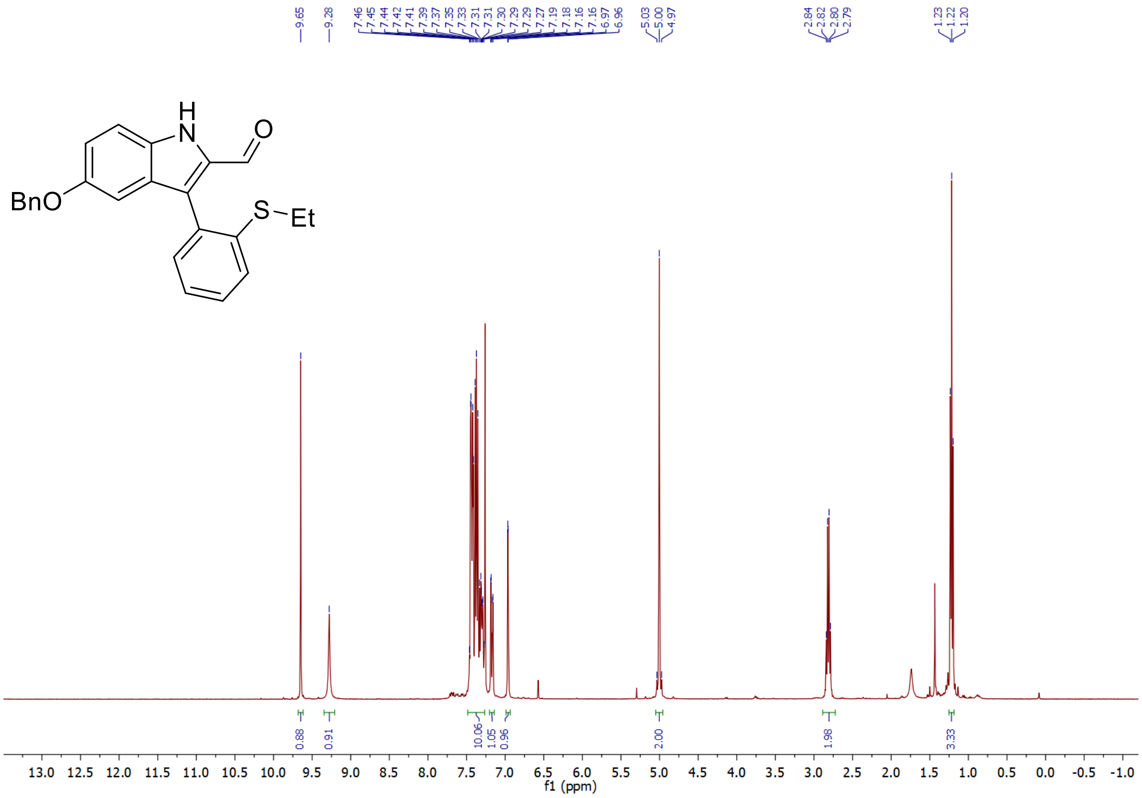

Figure S29. ^{13}C-NMR (100 MHz, $CDCl_3$) of **8u**:

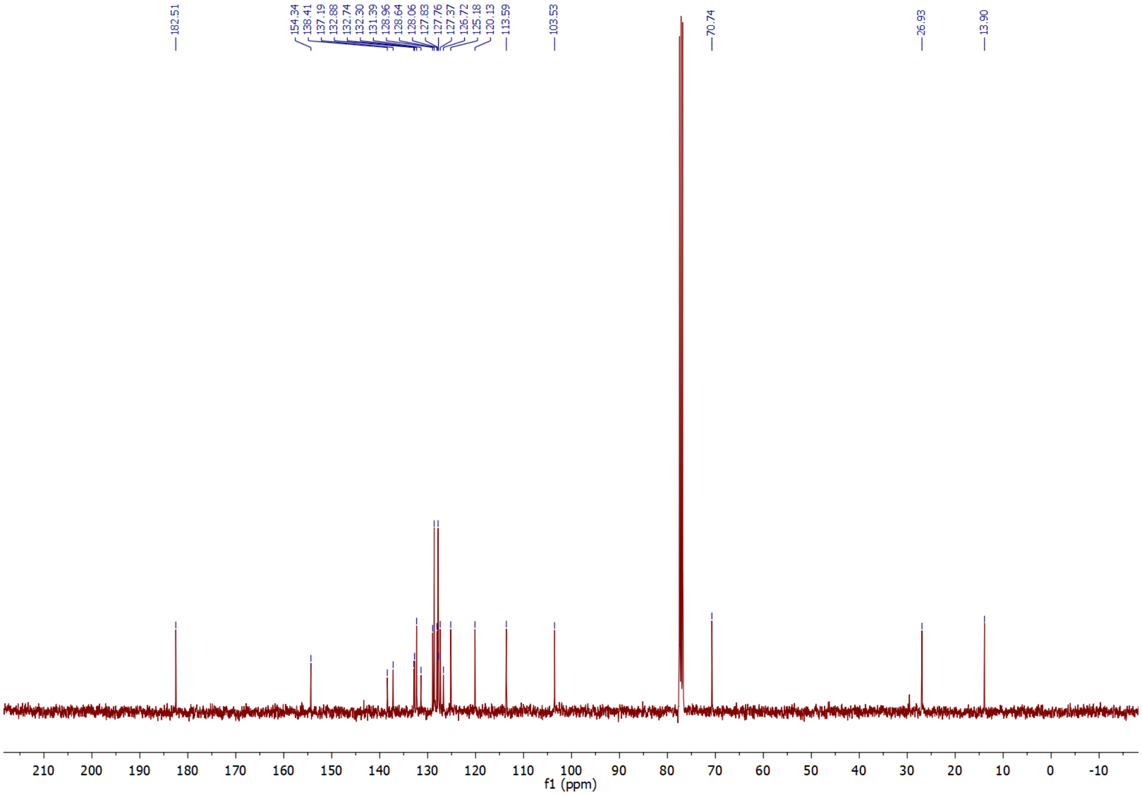

Figure S30. ^{1}H-NMR (400 MHz, $CDCl_3$) of **8v**:

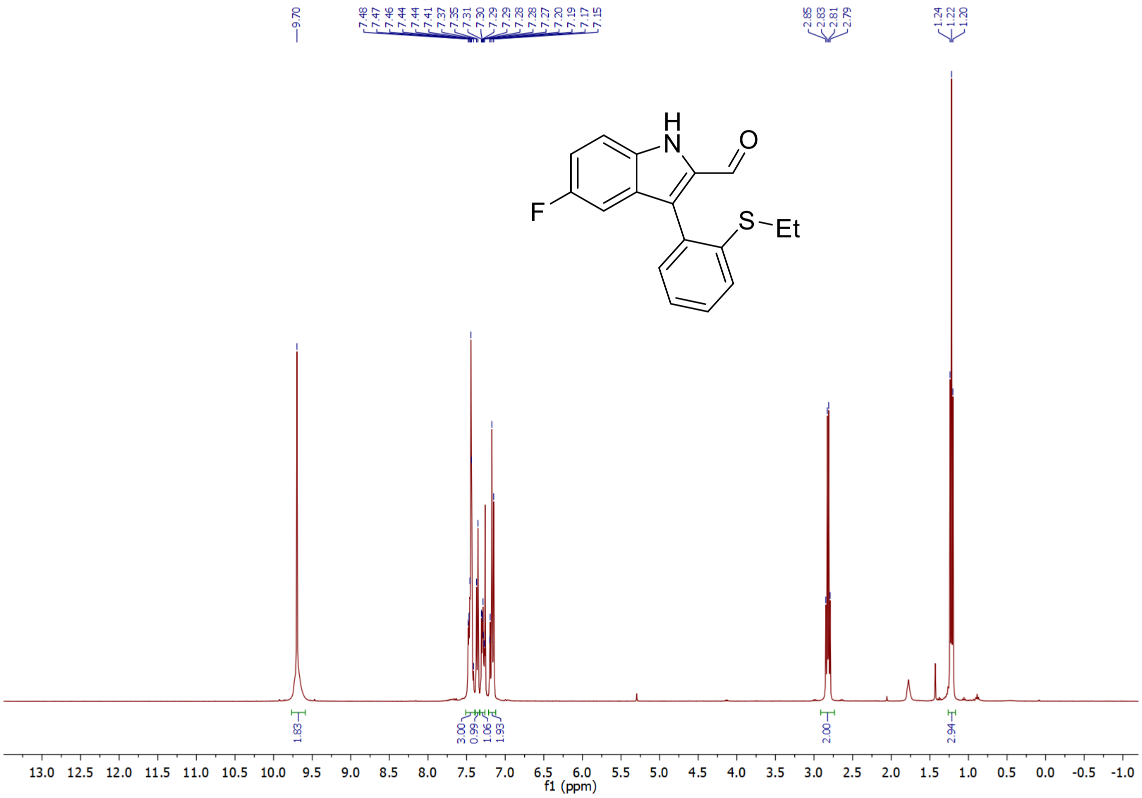

Figure S31. ^{13}C-NMR (100 MHz, $CDCl_3$) of **8v**:

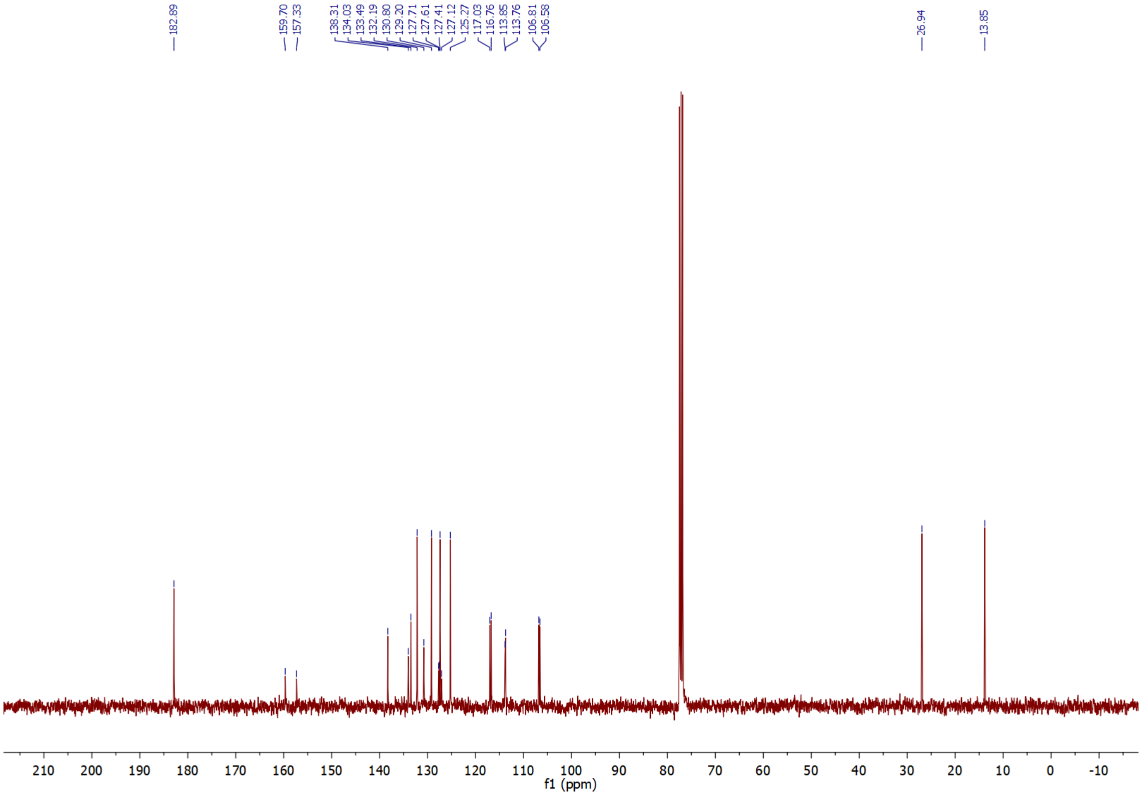

Figure S32. ^{19}F-NMR (376 MHz, $CDCl_3$) of **8v**:

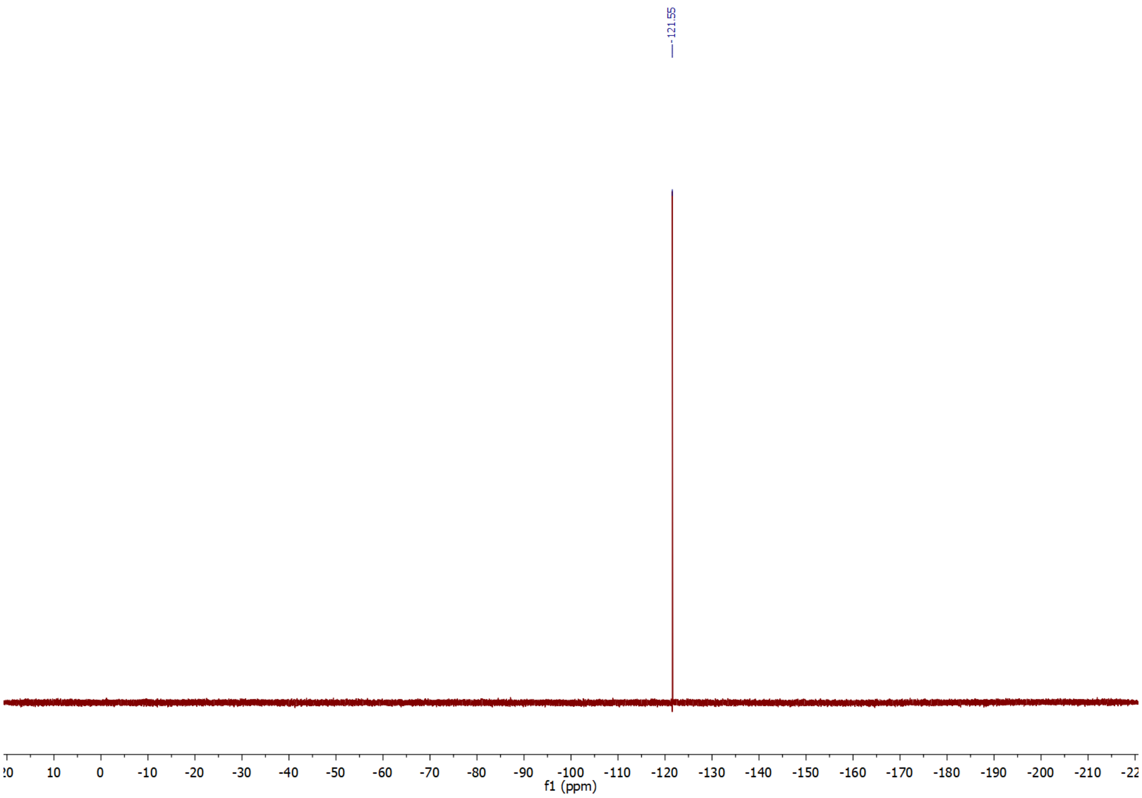

Figure S33. ^{1}H-NMR (400 MHz, $CDCl_3$) of **8x**:

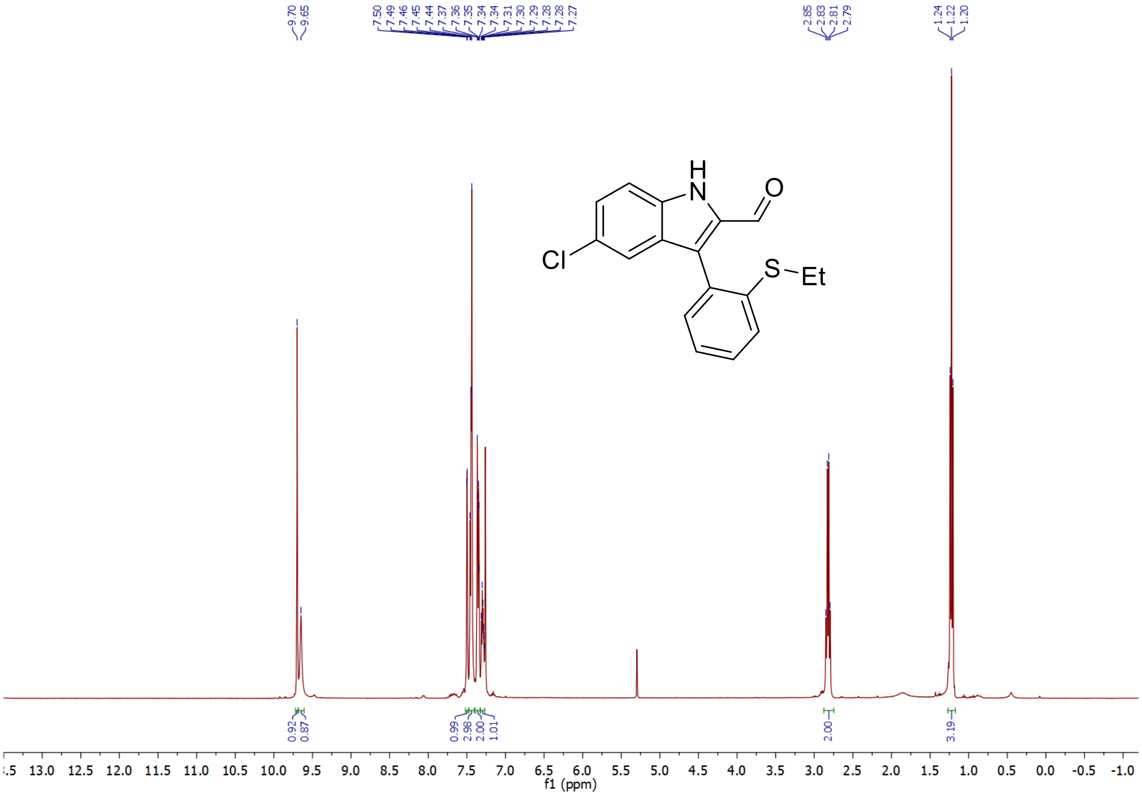

Figure S34. ^{13}C-NMR (100 MHz, $CDCl_3$) of **8x**:

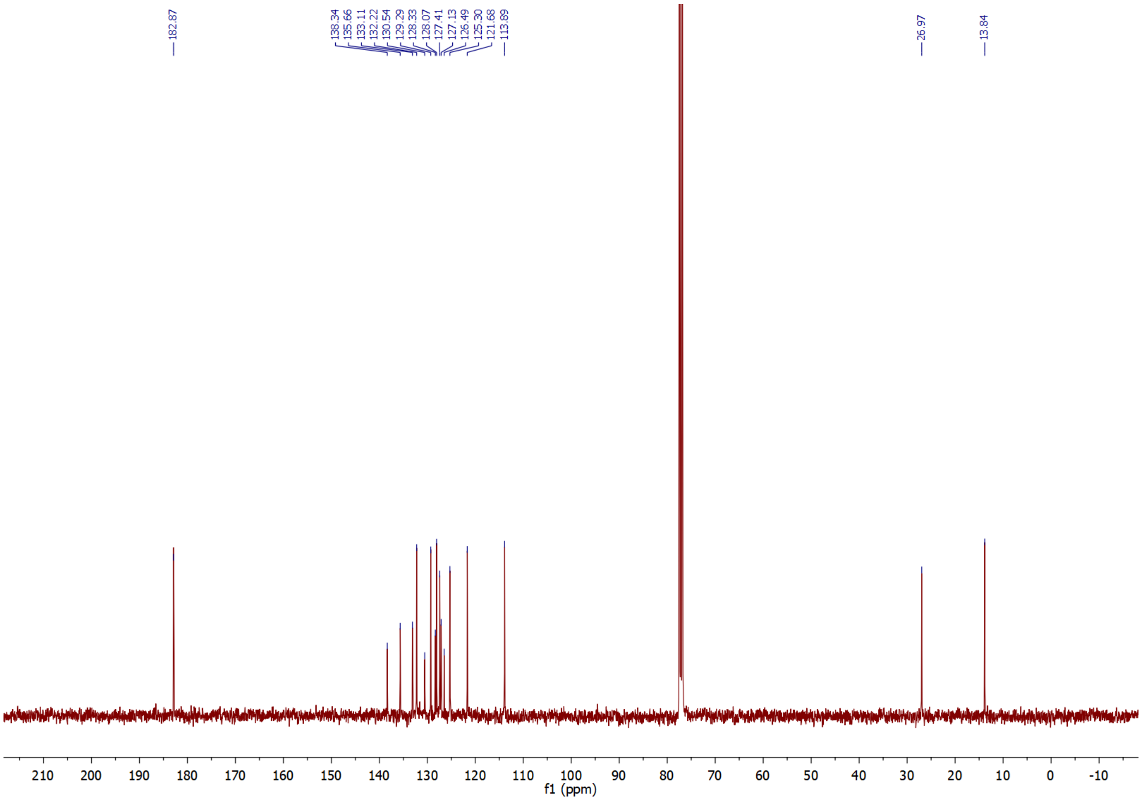

Figure S35. ^{1}H-NMR (400 MHz, $CDCl_3$) of **8y**:

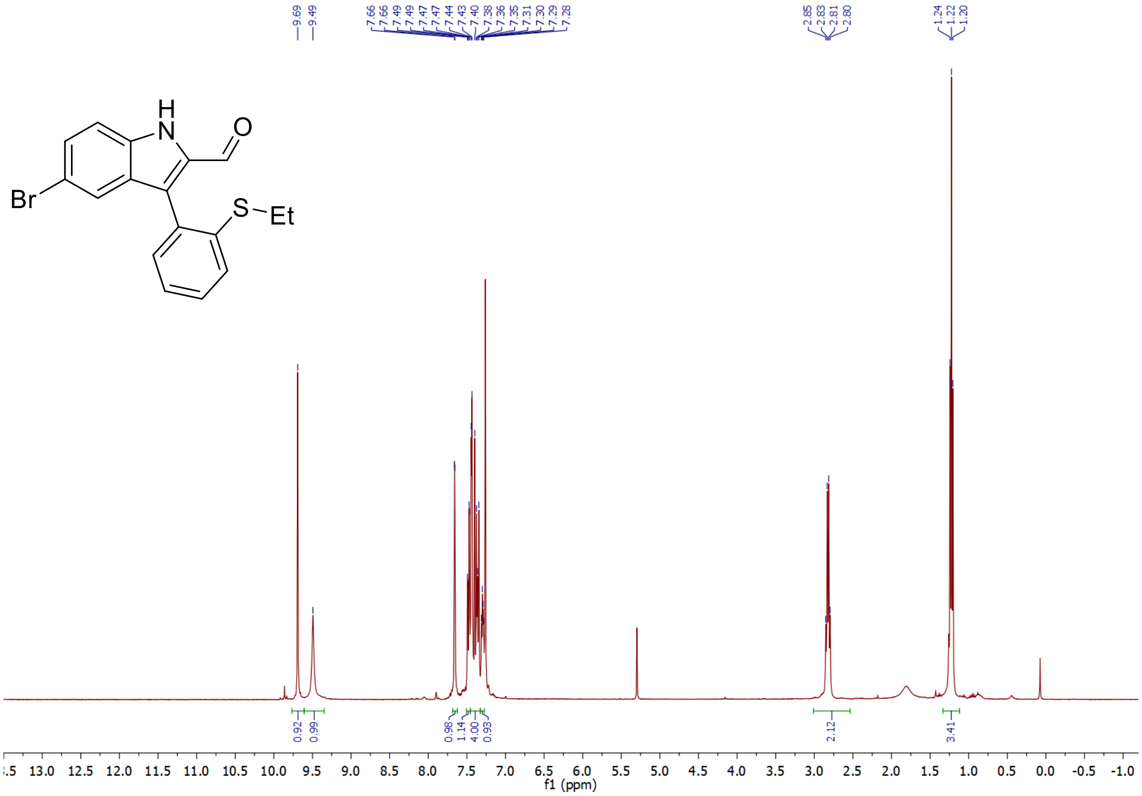

Figure S36. ^{13}C-NMR (100 MHz, $CDCl_3$) of **8y**:

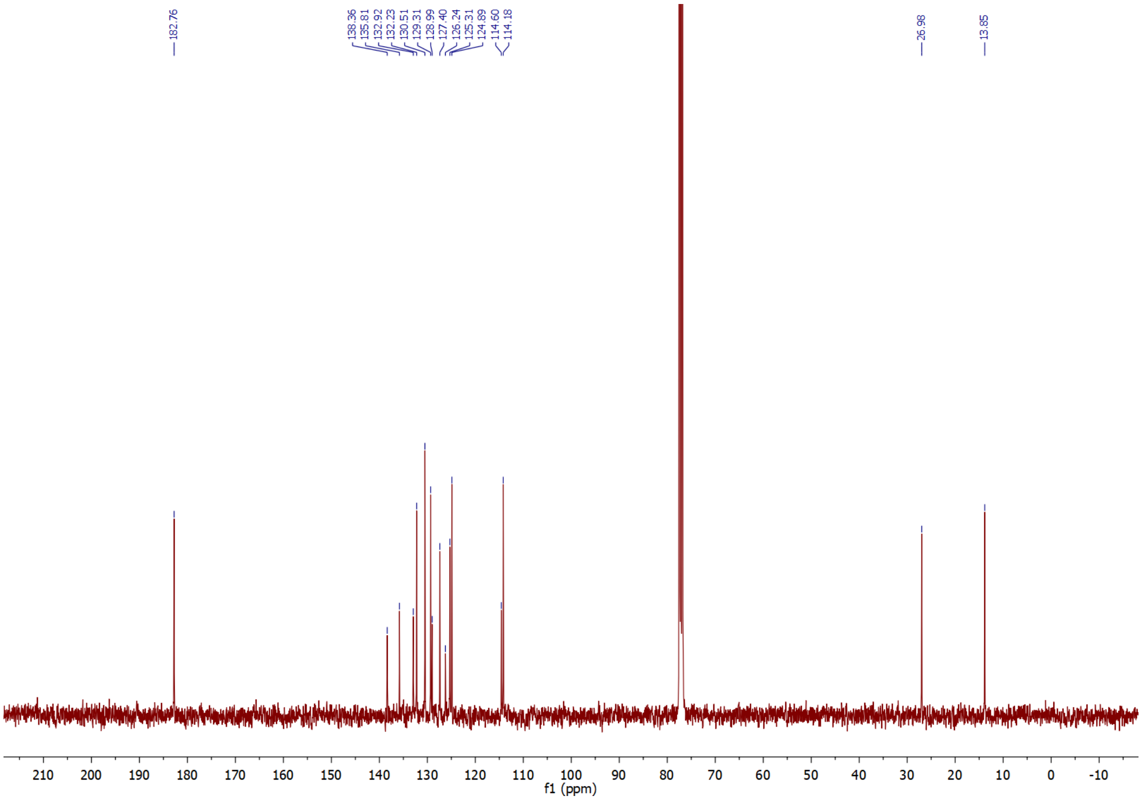

Figure S37. ^{1}H-NMR (400 MHz, DMSO-d_6) of **8z**:

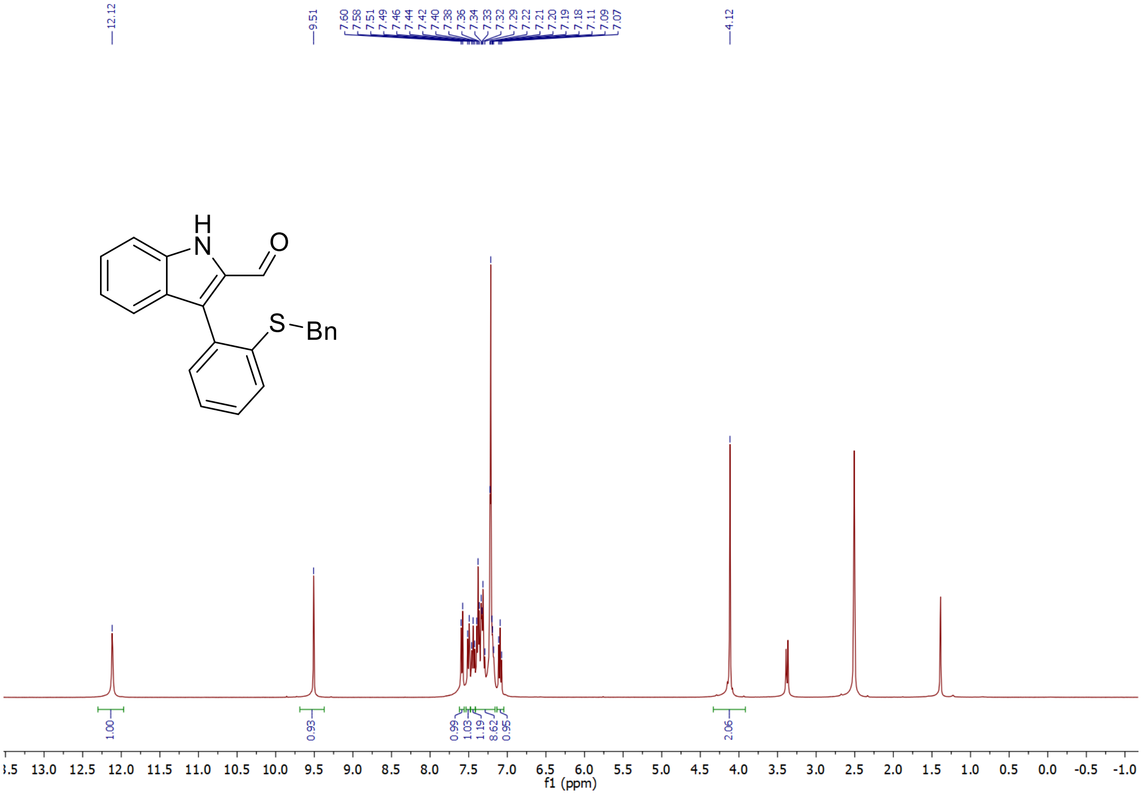

Figure S38. ^{13}C-NMR (100 MHz, DMSO-d_6) of **8z**:

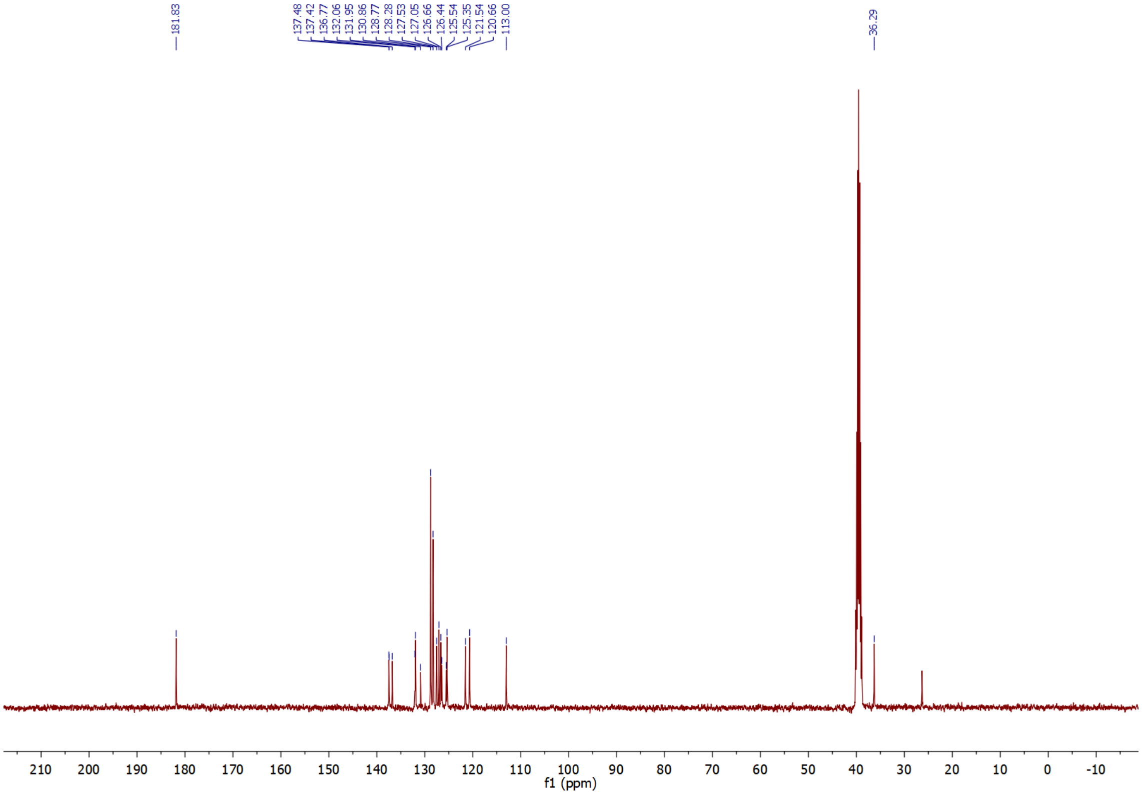

Figure S39. ^{1}H-NMR (400 MHz, $CDCl_3$) of **8ac**:

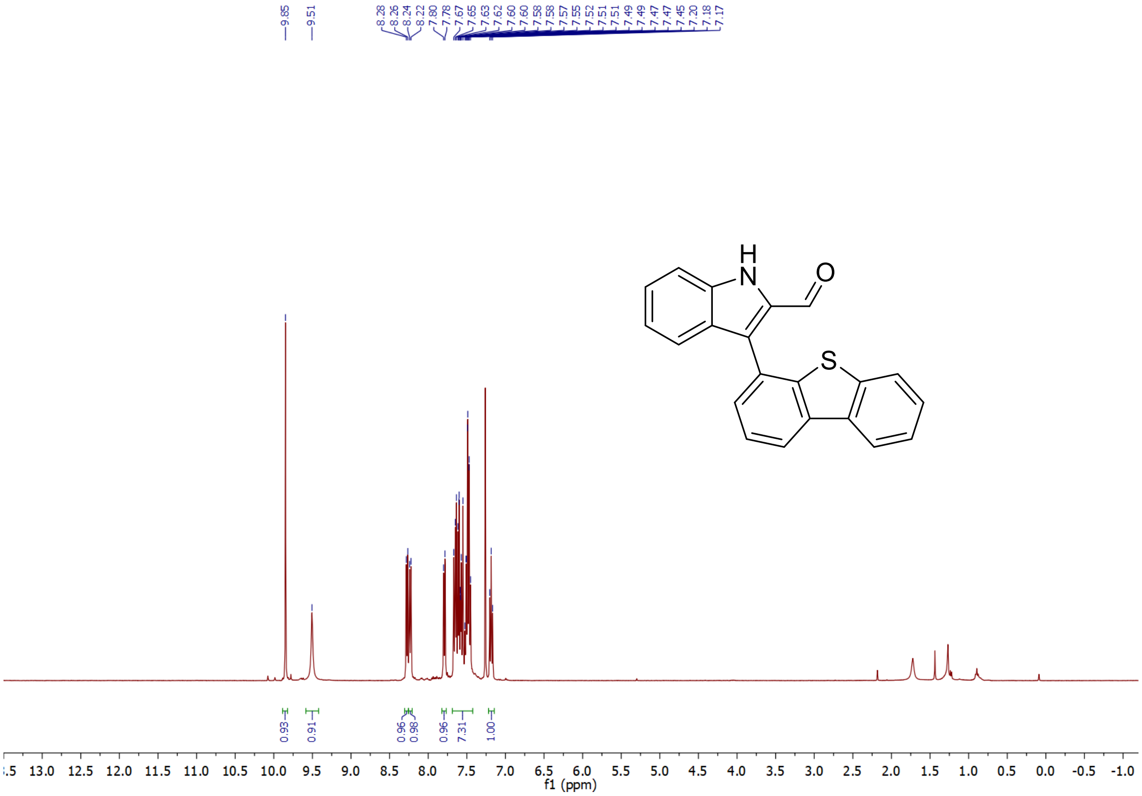

Figure S40. ^{13}C-NMR (100 MHz, $CDCl_3$) of **8ac**:

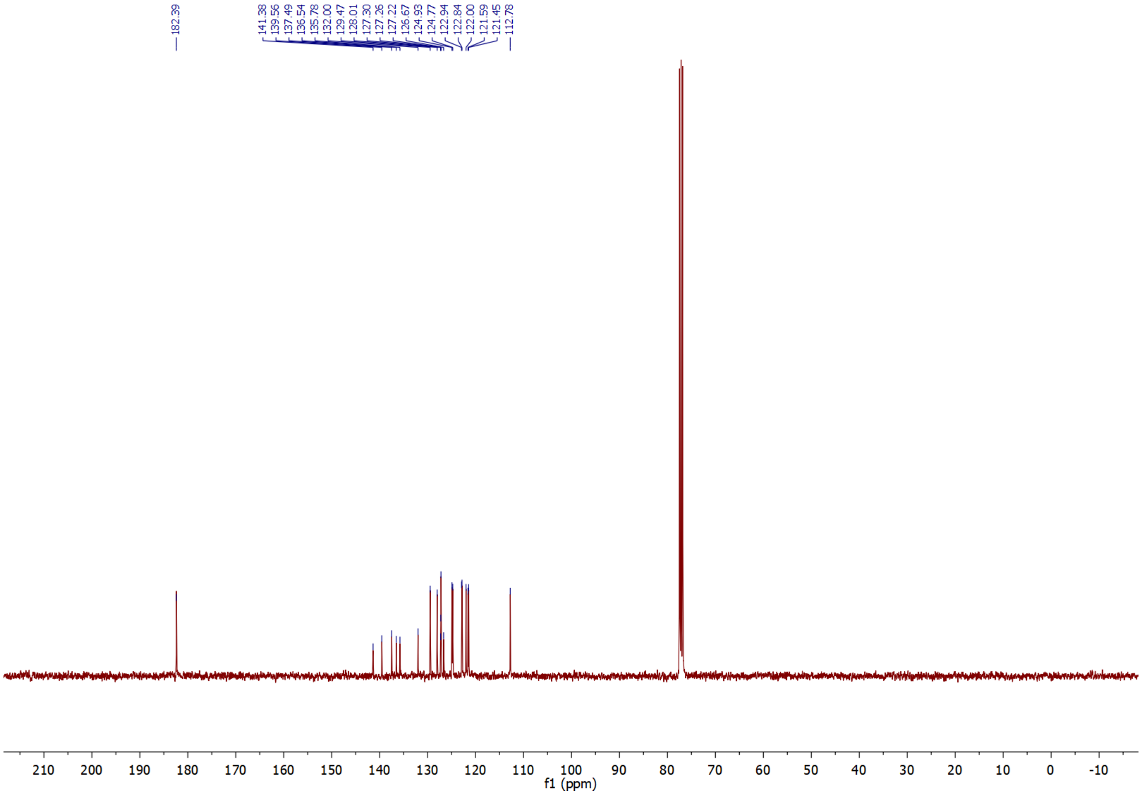

Figure S41. ^{1}H-NMR (400 MHz, $CDCl_3$) of **1a**:

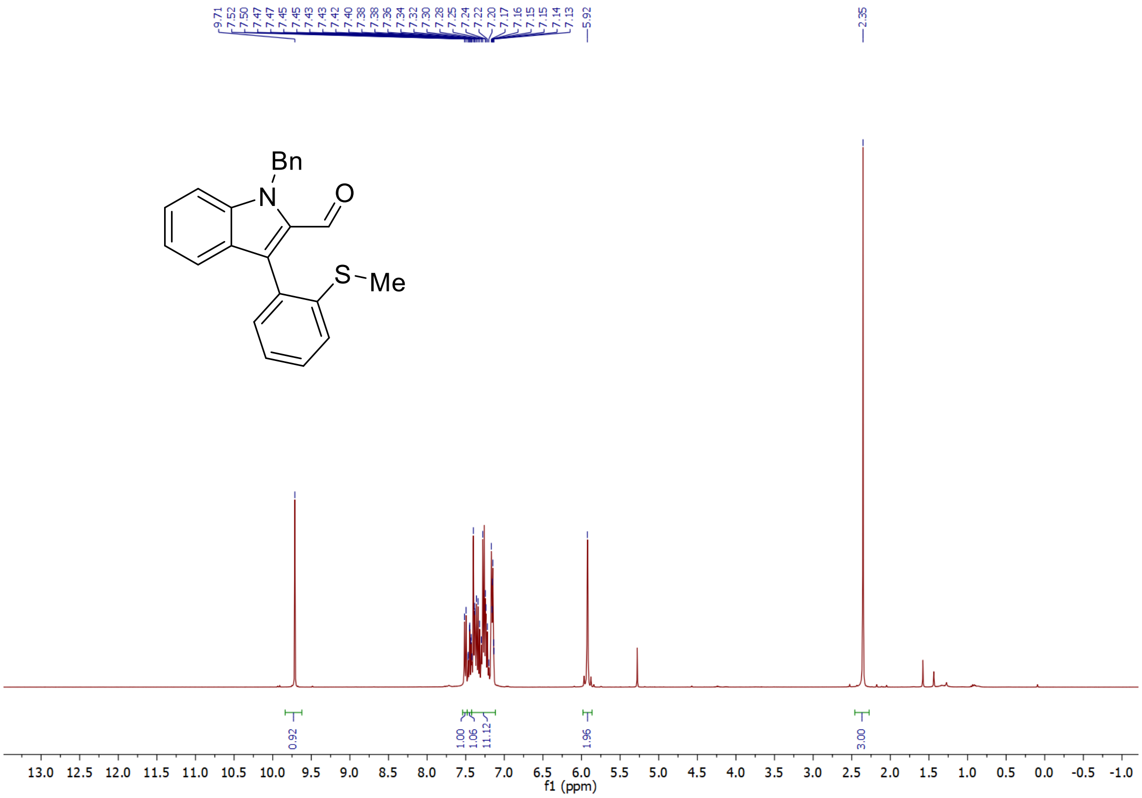

Figure S42. ^{13}C-NMR (100 MHz, $CDCl_3$) of **1a**:

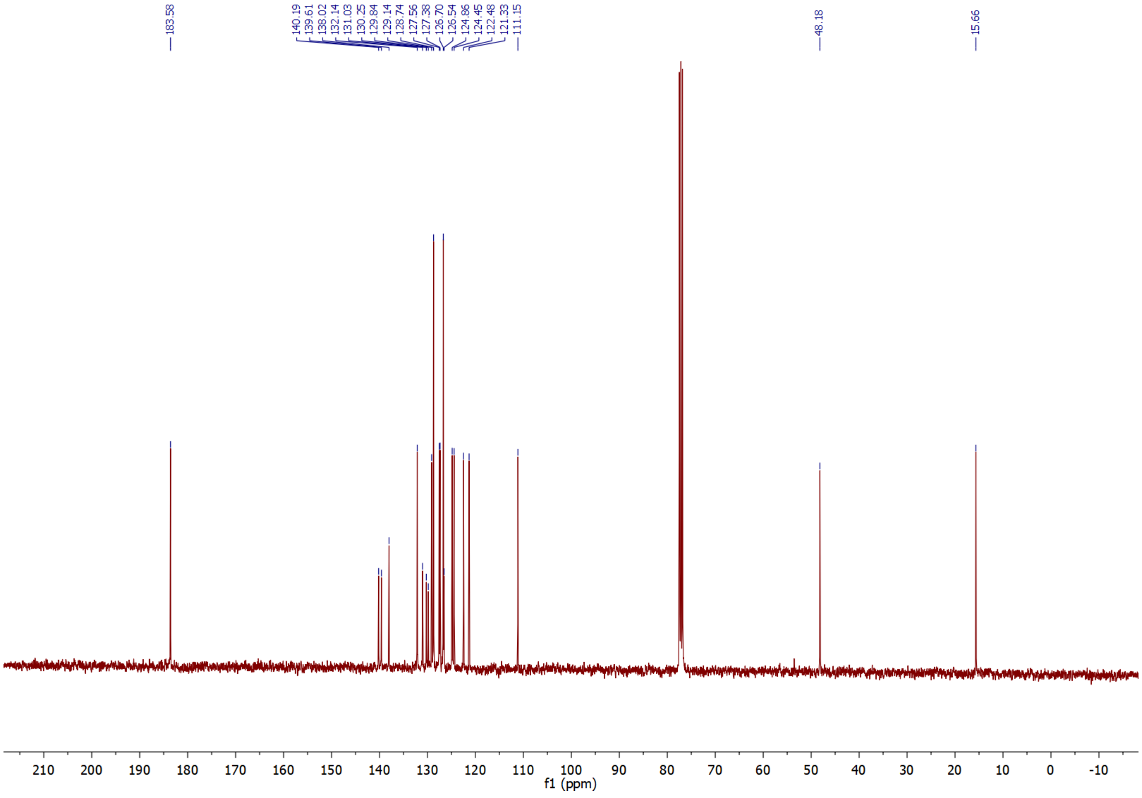

Figure S43. ^{1}H-NMR (400 MHz, $CDCl_3$) of **1b**:

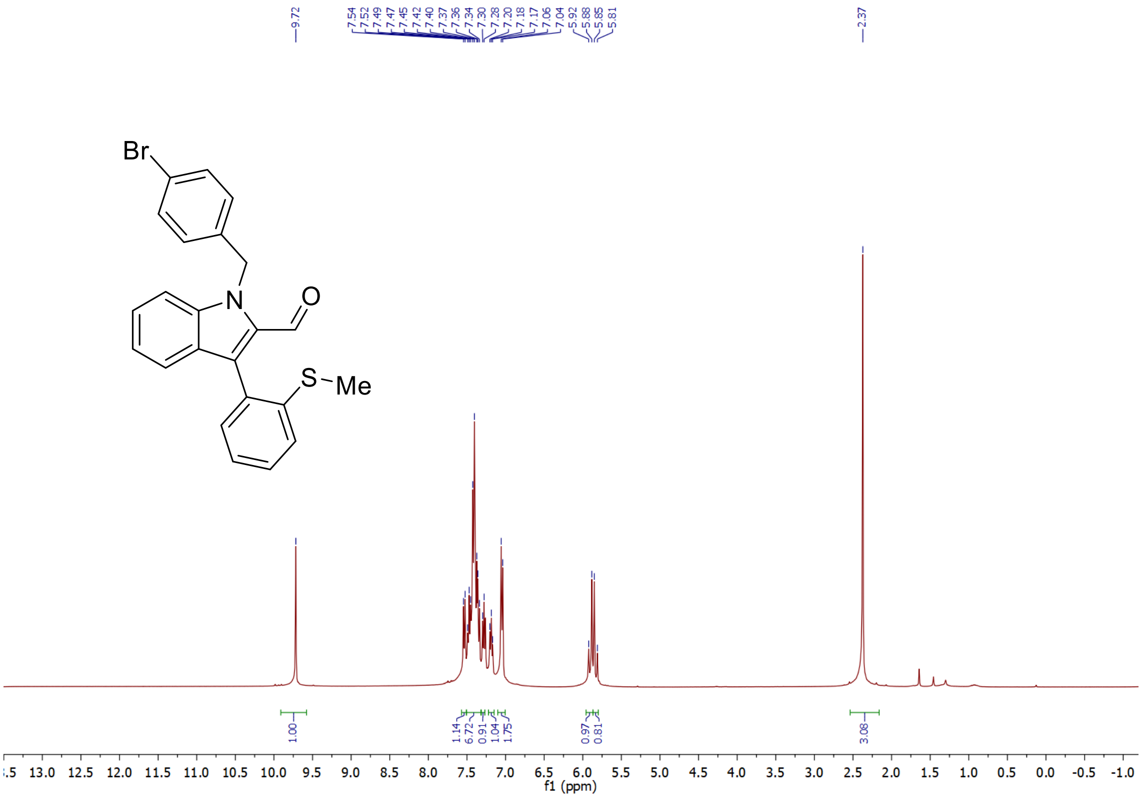

Figure S44. ^{13}C-NMR (100 MHz, $CDCl_3$) of **1b**:

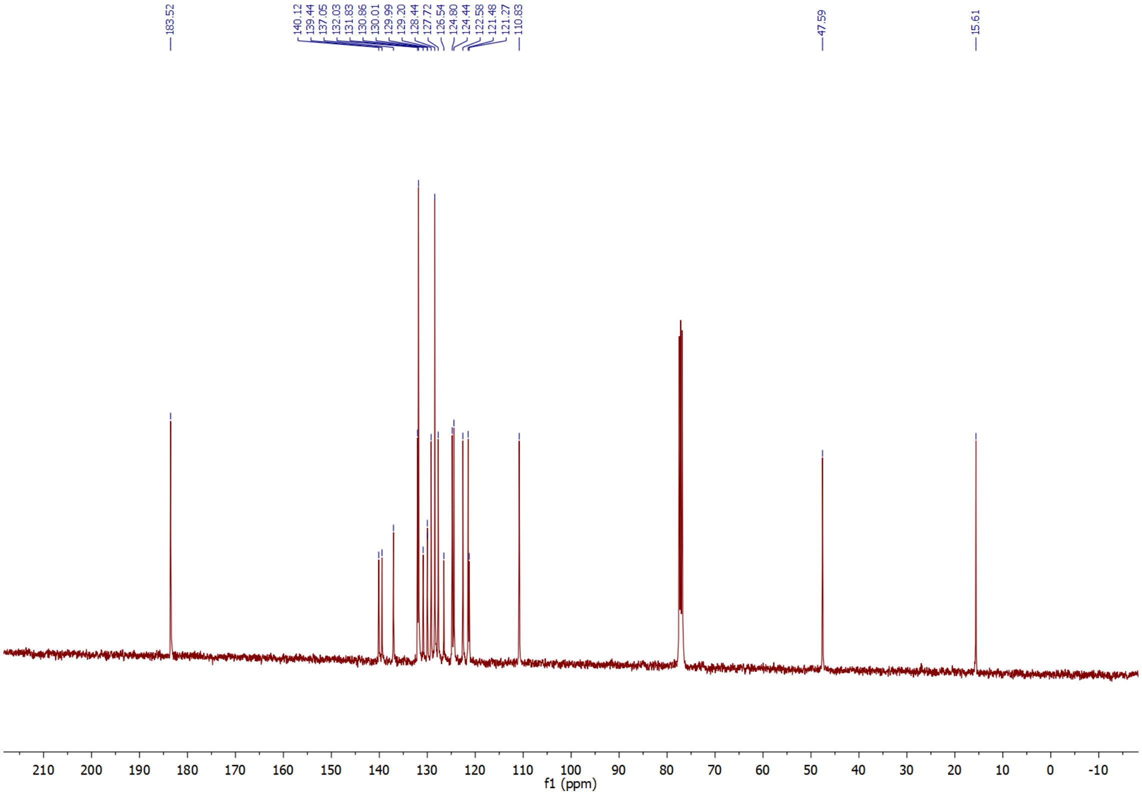

Figure S45. ^{1}H-NMR (400 MHz, $CDCl_3$) of **1c**:

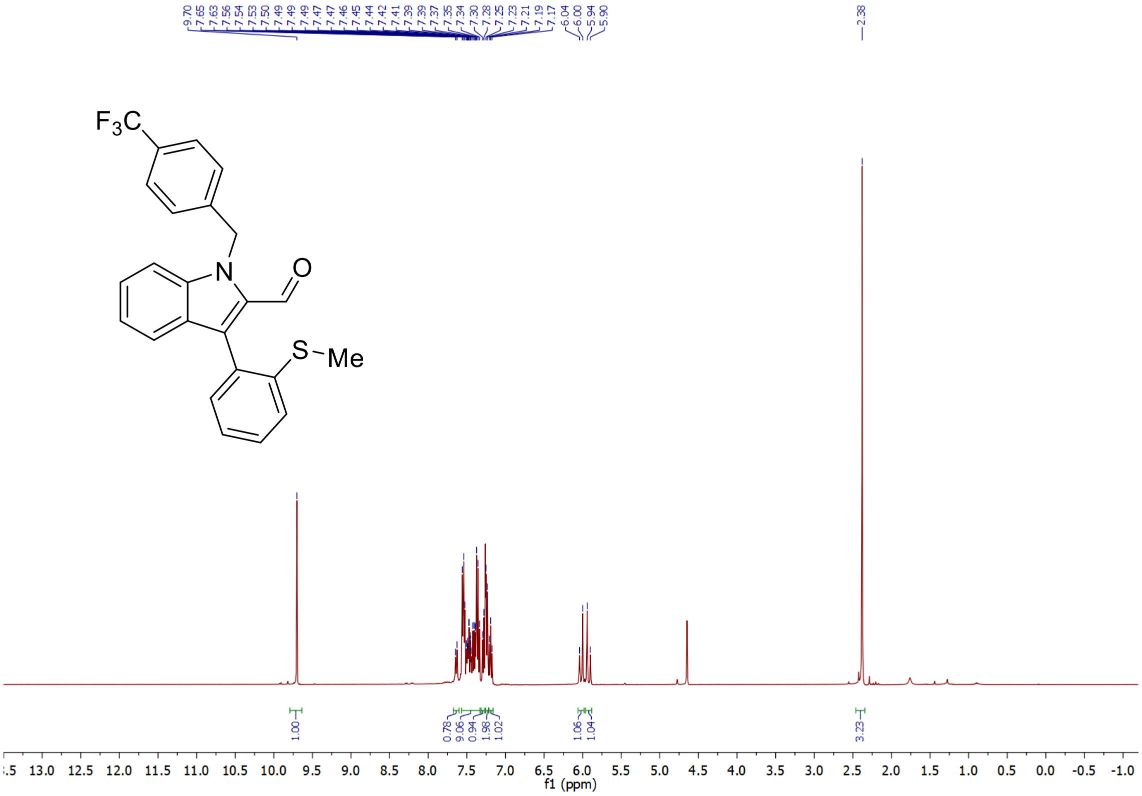

Figure S46. ^{13}C-NMR (100 MHz, $CDCl_3$) of **1c**:

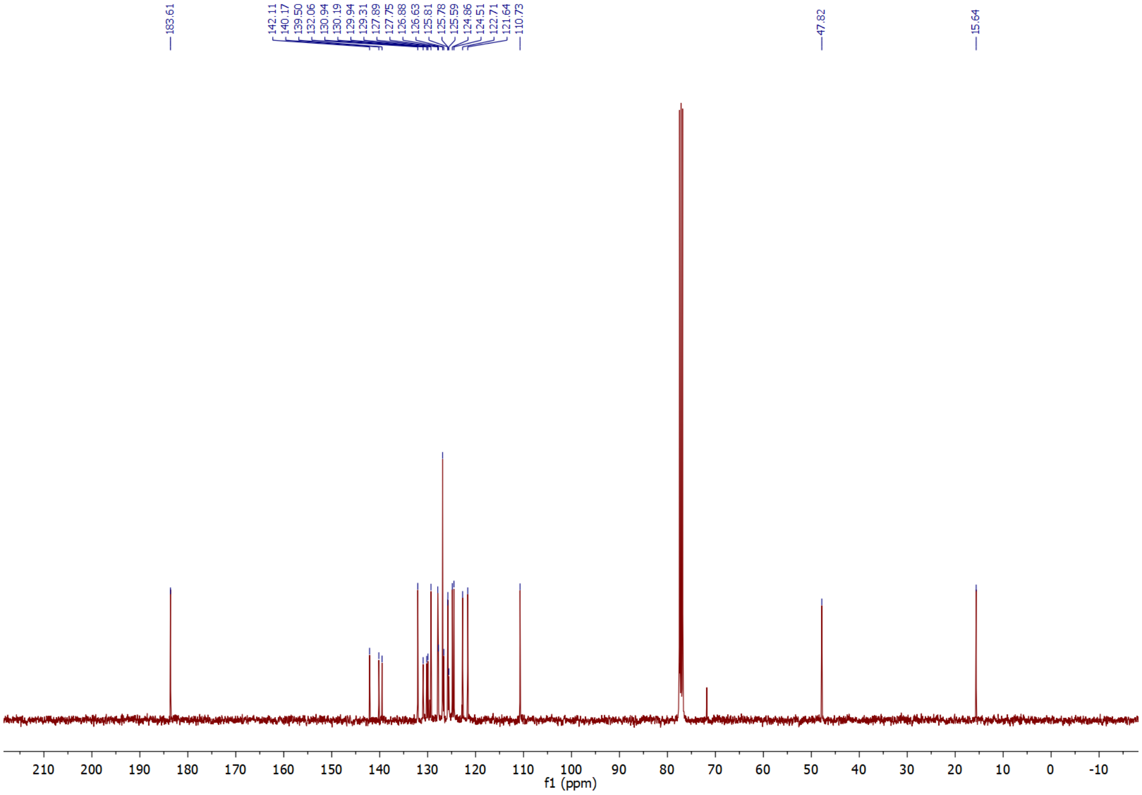

Figure S47. ^{19}F-NMR (376 MHz, $CDCl_3$) of **1c**:

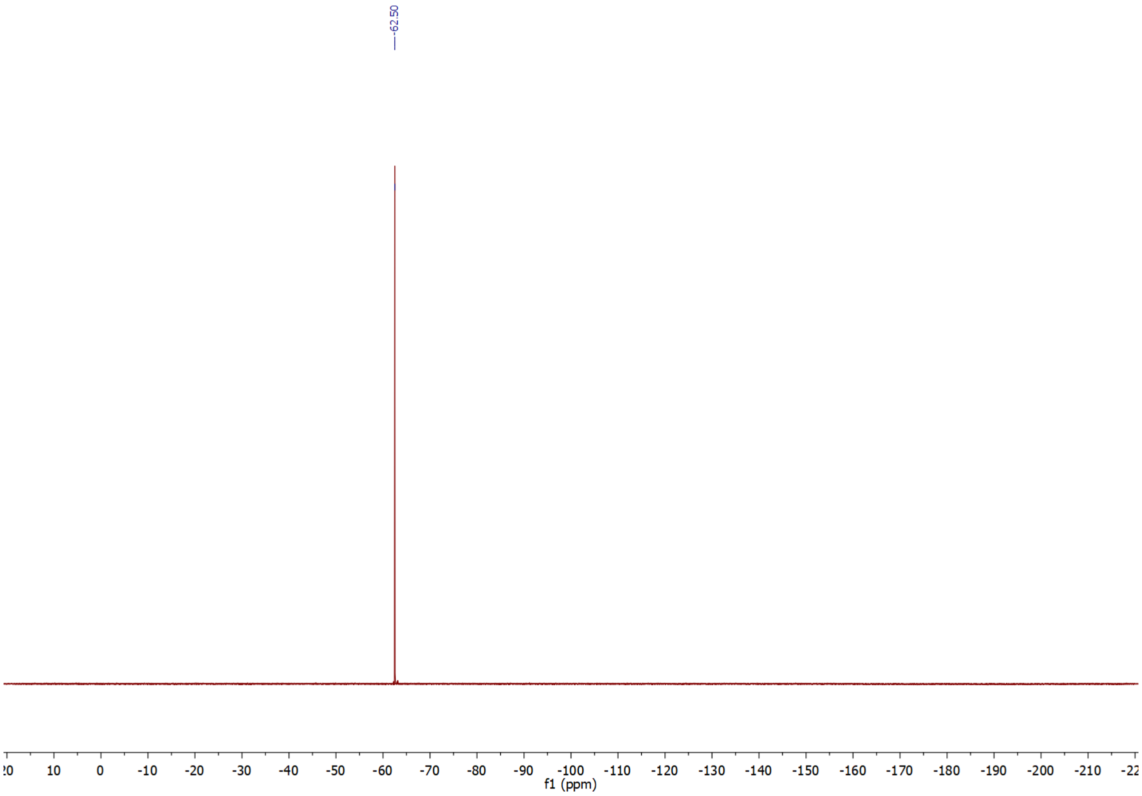

Figure S48. ^{1}H-NMR (400 MHz, $CDCl_3$) of **1d**:

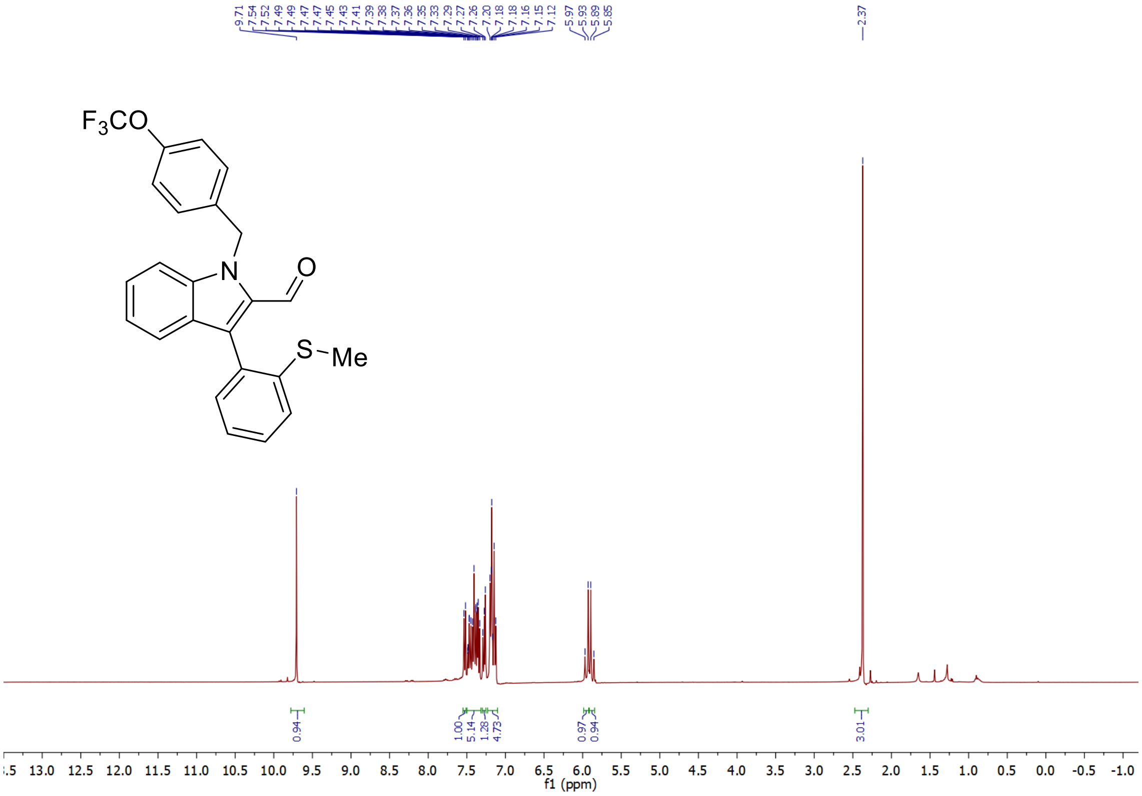

Figure S49. ^{13}C-NMR (100 MHz, $CDCl_3$) of **1d**:

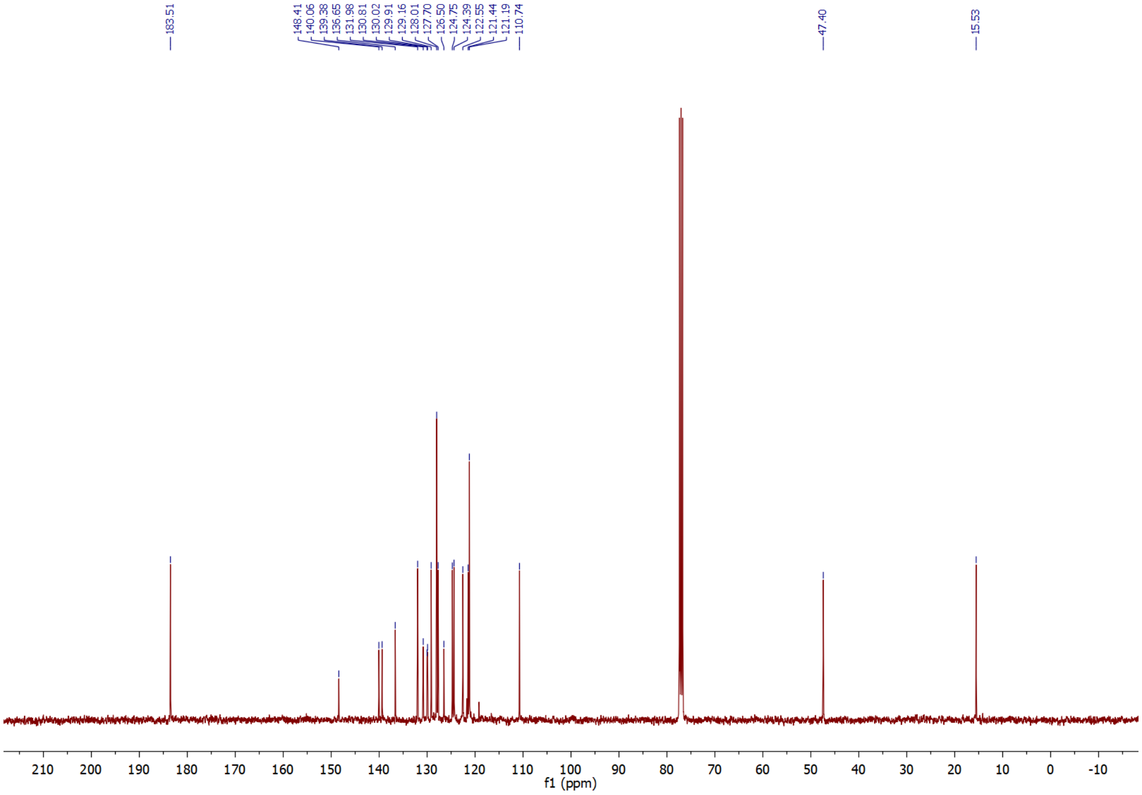

Figure S50. ^{19}F-NMR (376 MHz, $CDCl_3$) of **1d**:

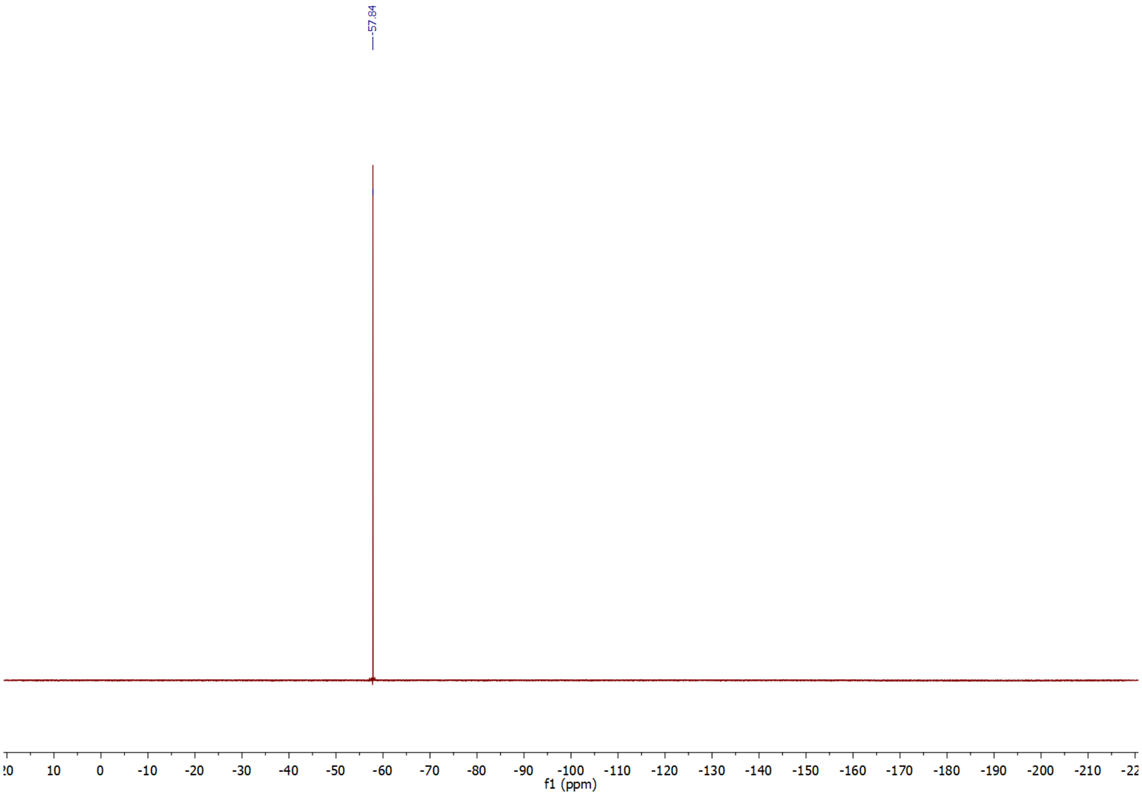

Figure S51. ^{1}H-NMR (400 MHz, $CDCl_3$) of **1e**:

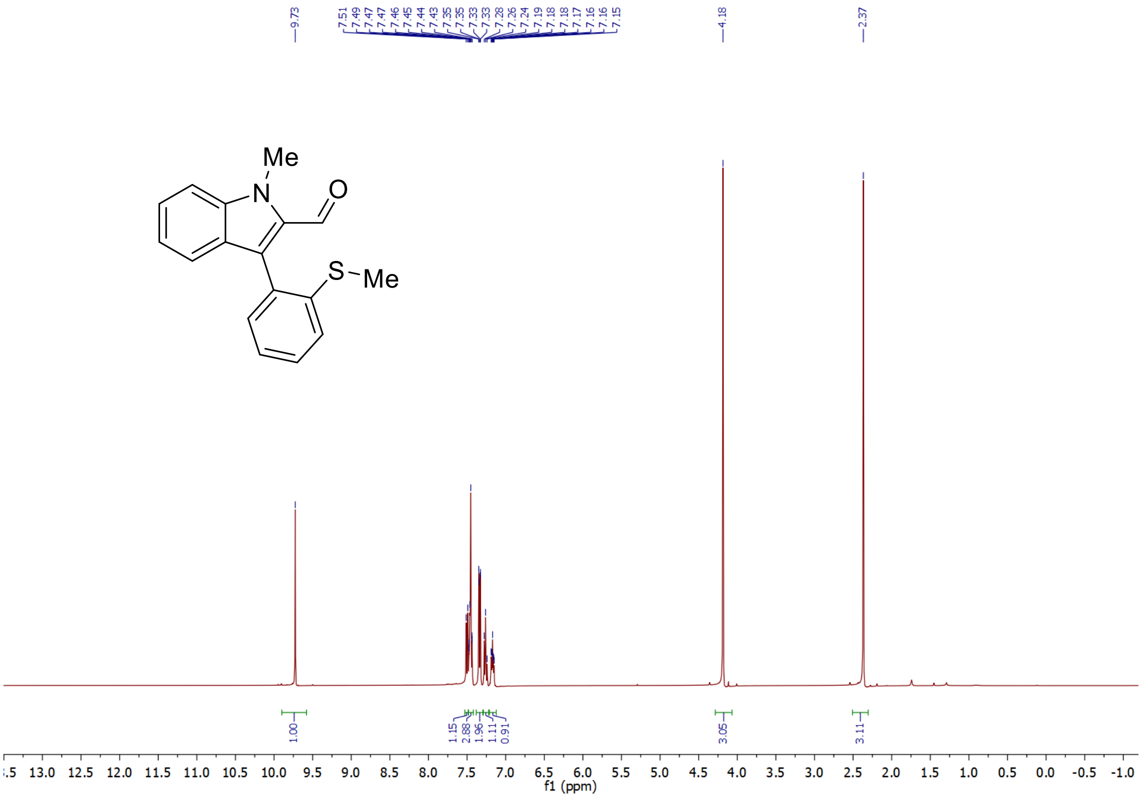

Figure S52. ^{13}C-NMR (100 MHz, $CDCl_3$) of **1e**:

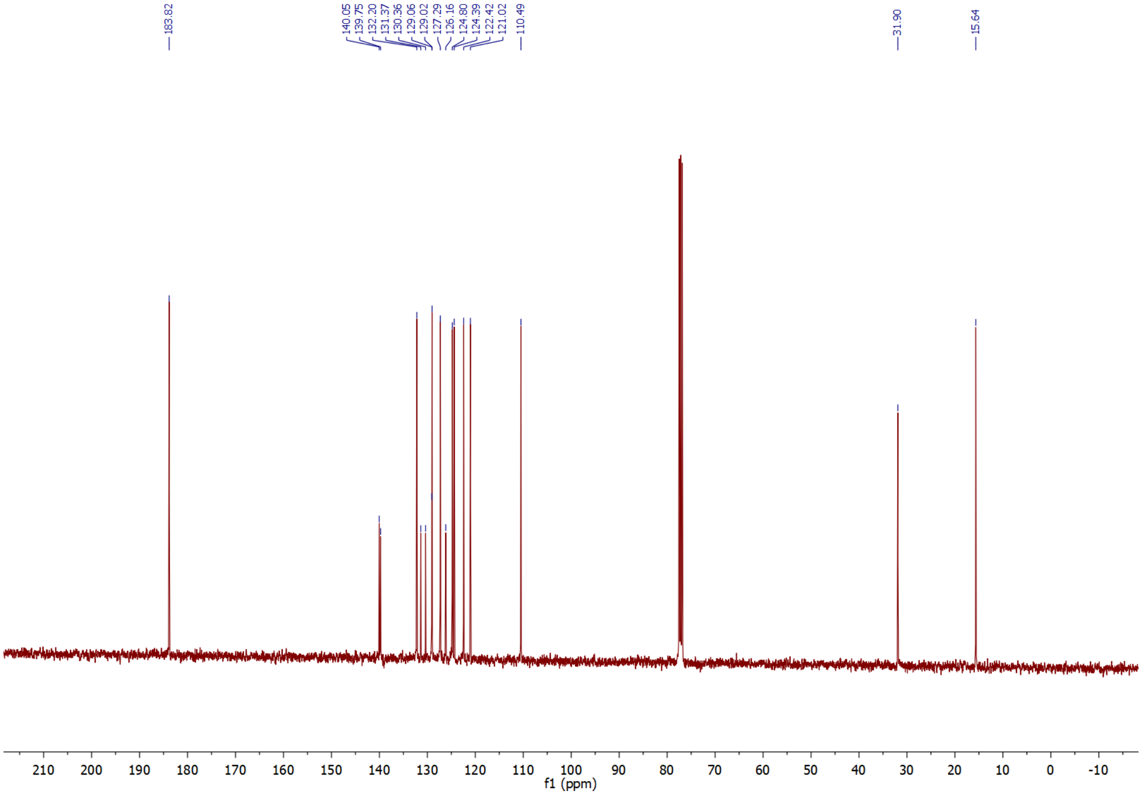

Figure S53. ^{1}H-NMR (400 MHz, $CDCl_3$) of **1f**:

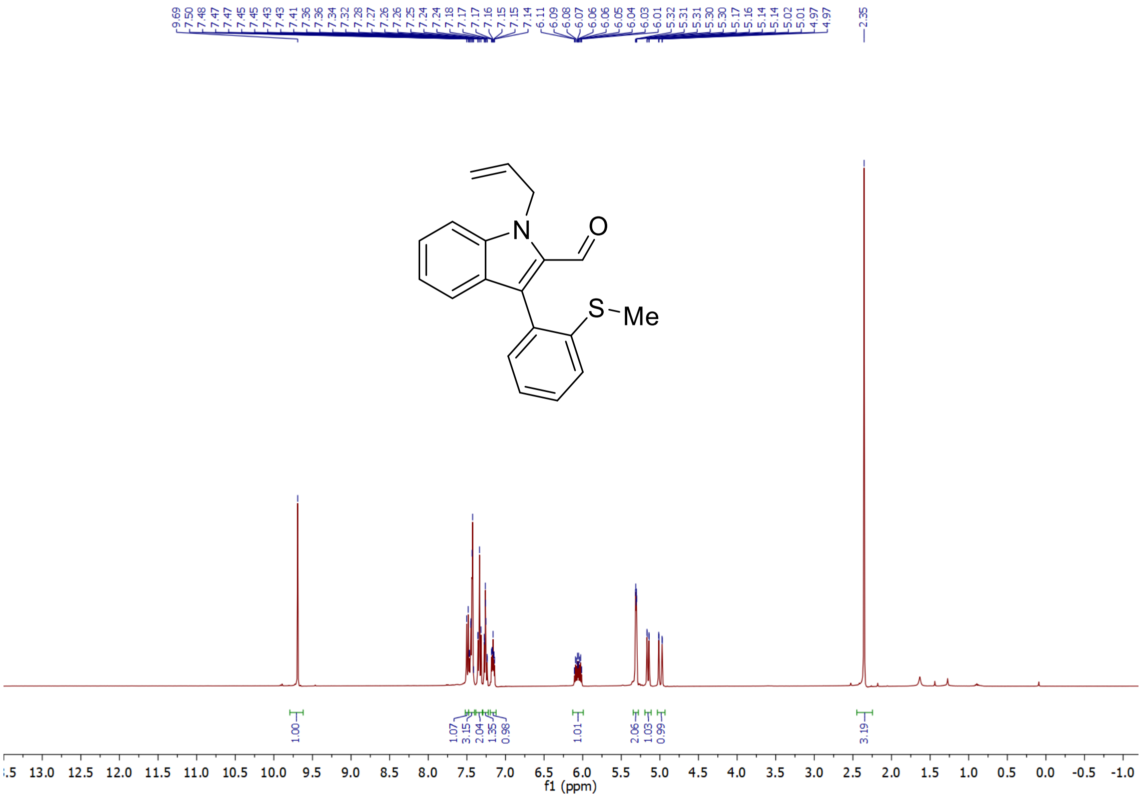

Figure S54. ^{13}C-NMR (100 MHz, $CDCl_3$) of **1f**:

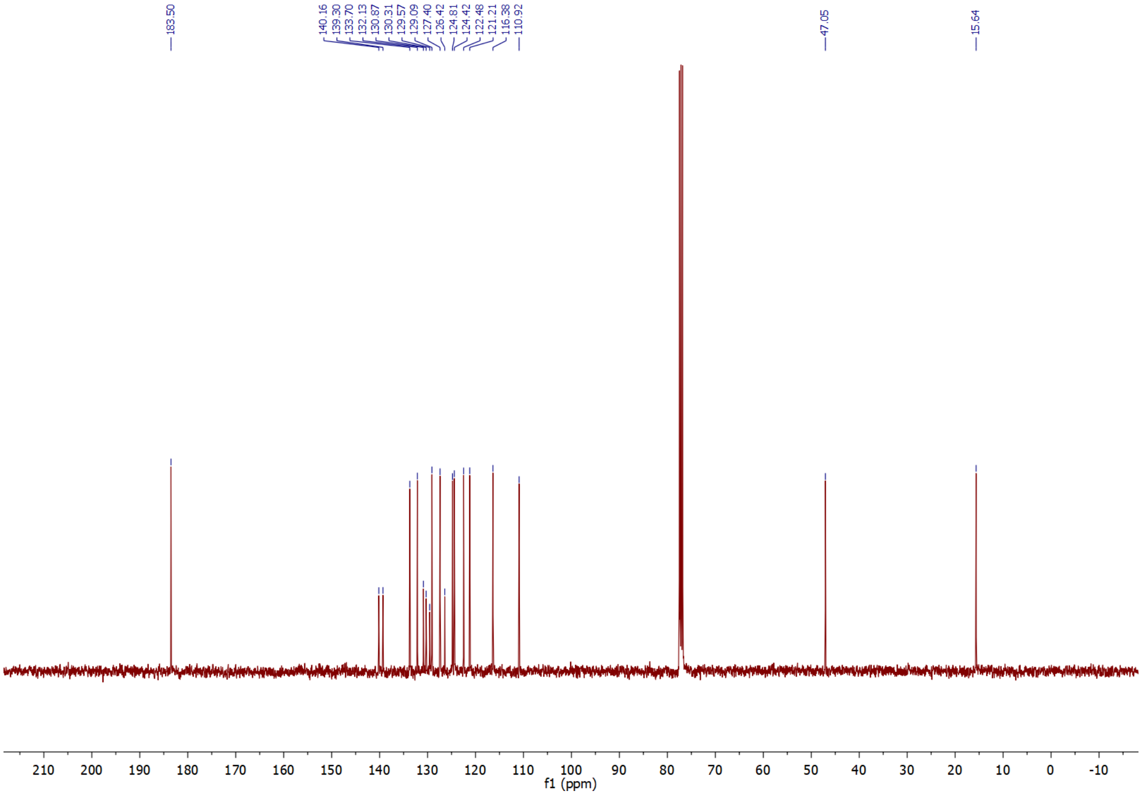

Figure S55. ^{1}H-NMR (400 MHz, $CDCl_3$) of **1g**:

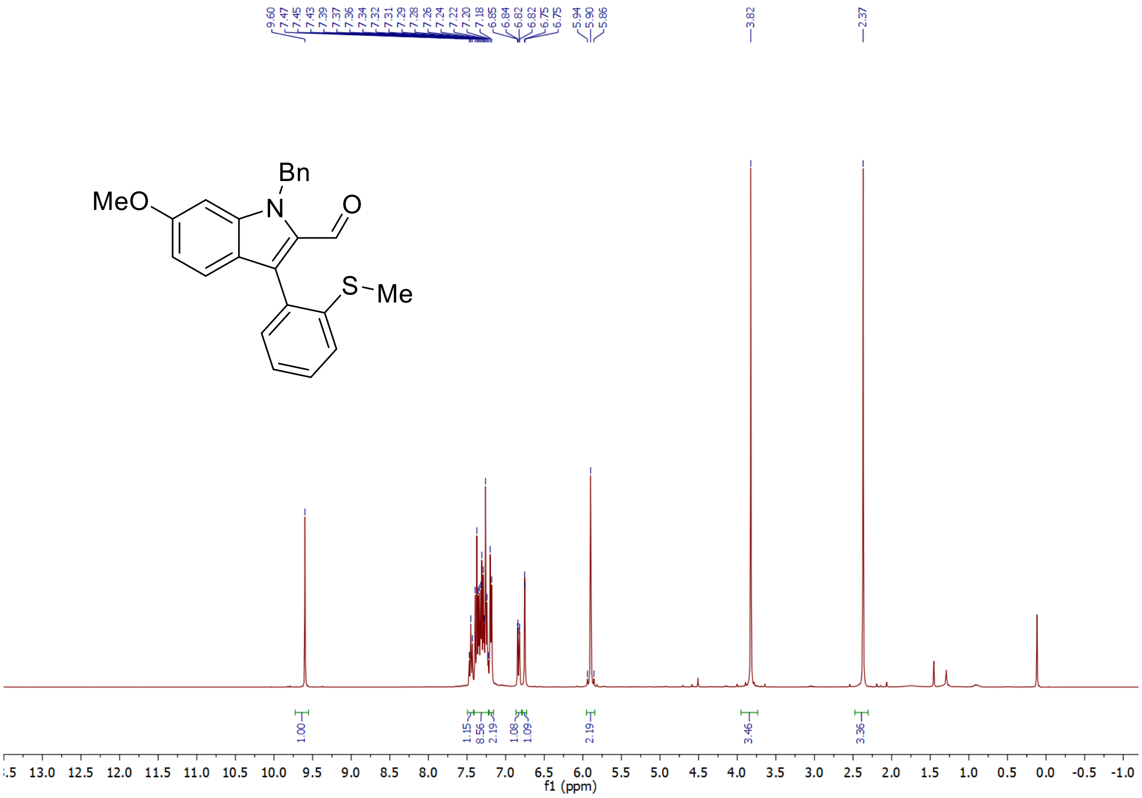

Figure S56. ^{13}C-NMR (100 MHz, $CDCl_3$) of **1g**:

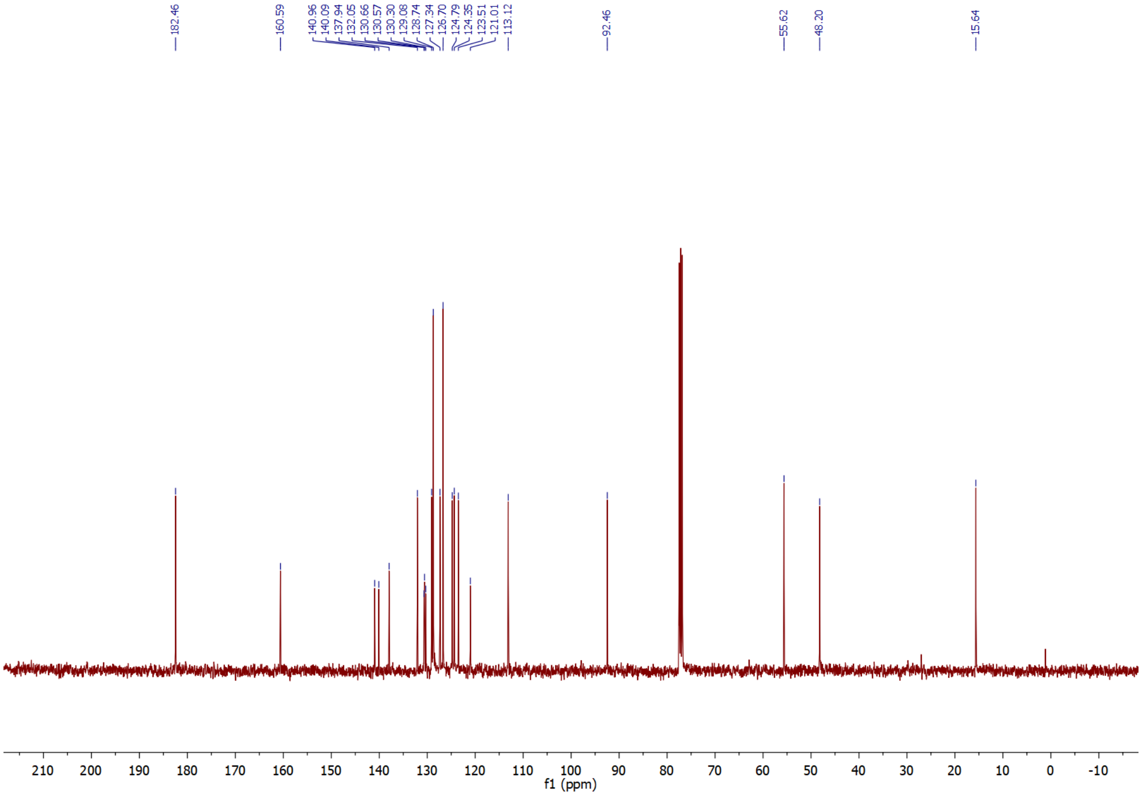

Figure S57. ^{1}H-NMR (400 MHz, $CDCl_3$) of **1h**:

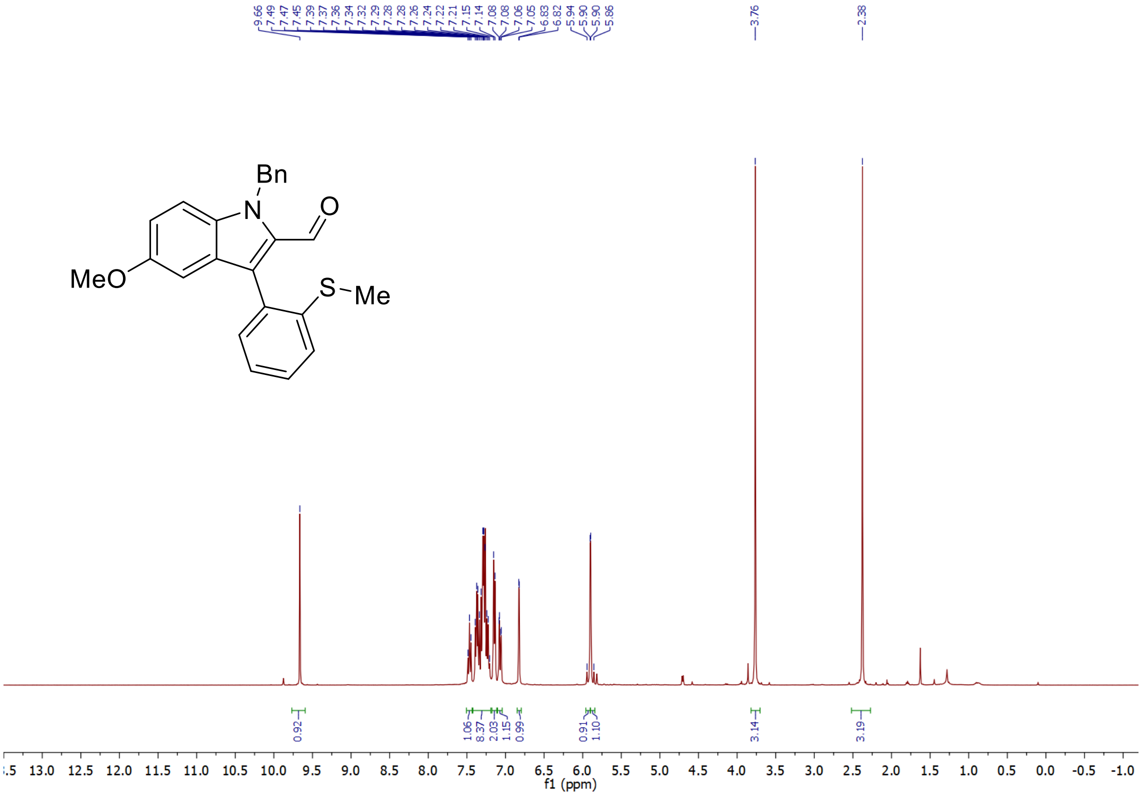

Figure S58. ^{13}C-NMR (100 MHz, $CDCl_3$) of **1h**:

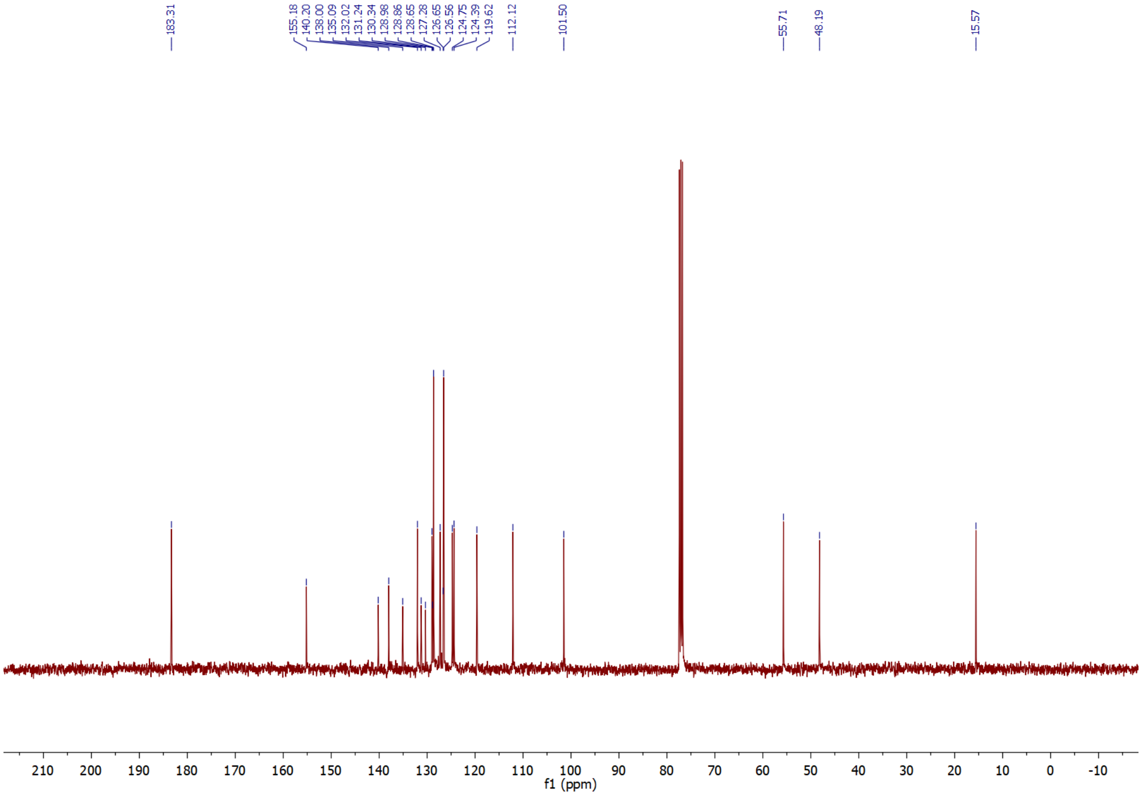

Figure S59. ^{1}H-NMR (400 MHz, $CDCl_3$) of **1i**:

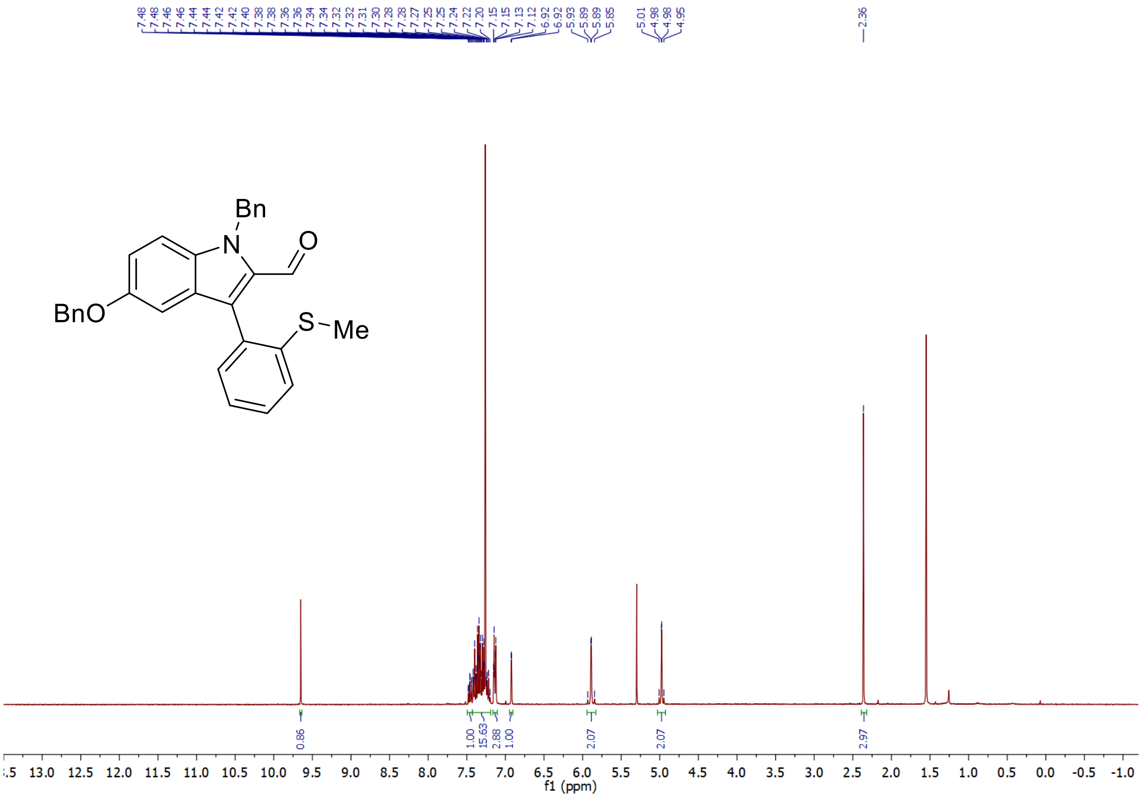

Figure S60. ^{13}C-NMR (100 MHz, $CDCl_3$) of **1i**:

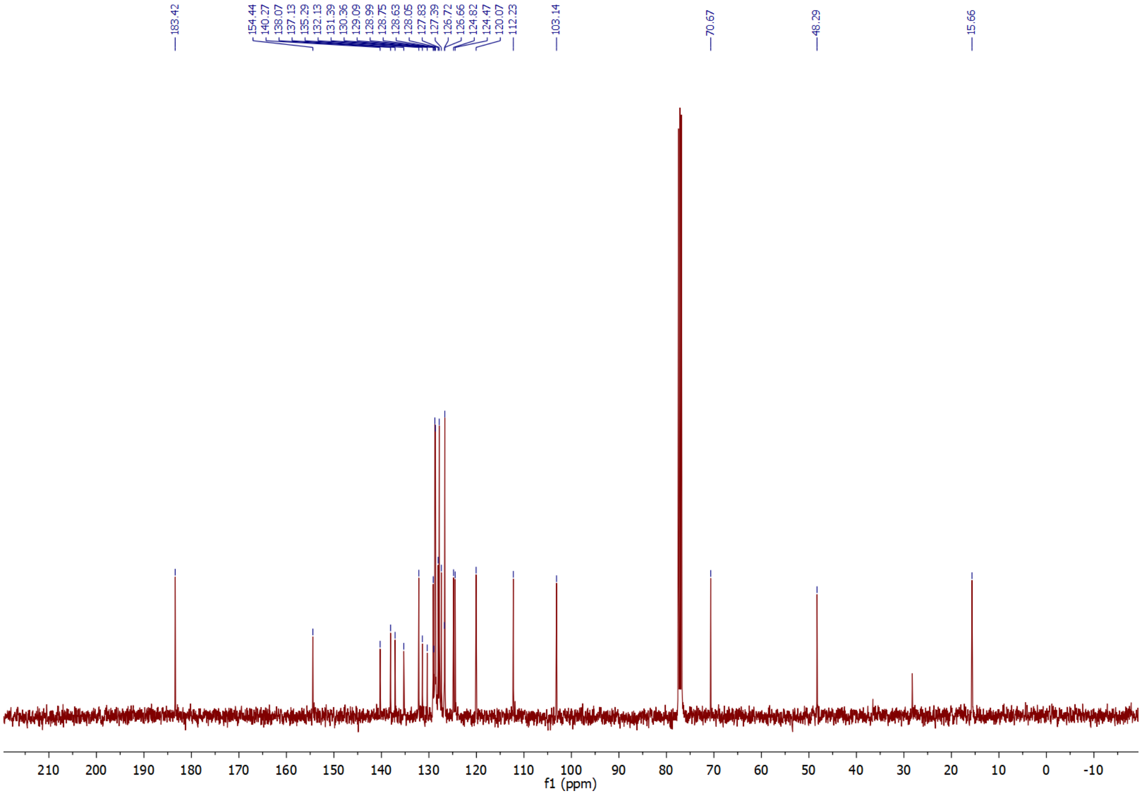

Figure S61. ^{1}H-NMR (400 MHz, $CDCl_3$) of **1j**:

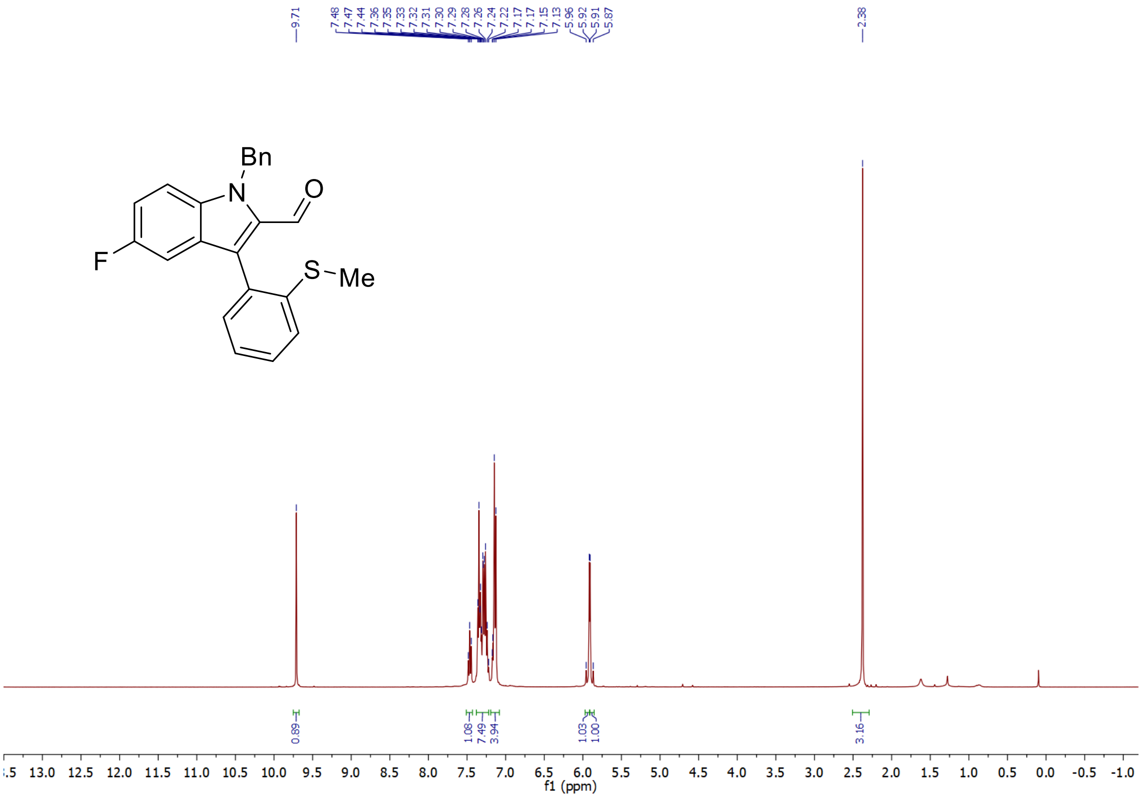

Figure S62. ^{13}C-NMR (100 MHz, $CDCl_3$) of **1j**:

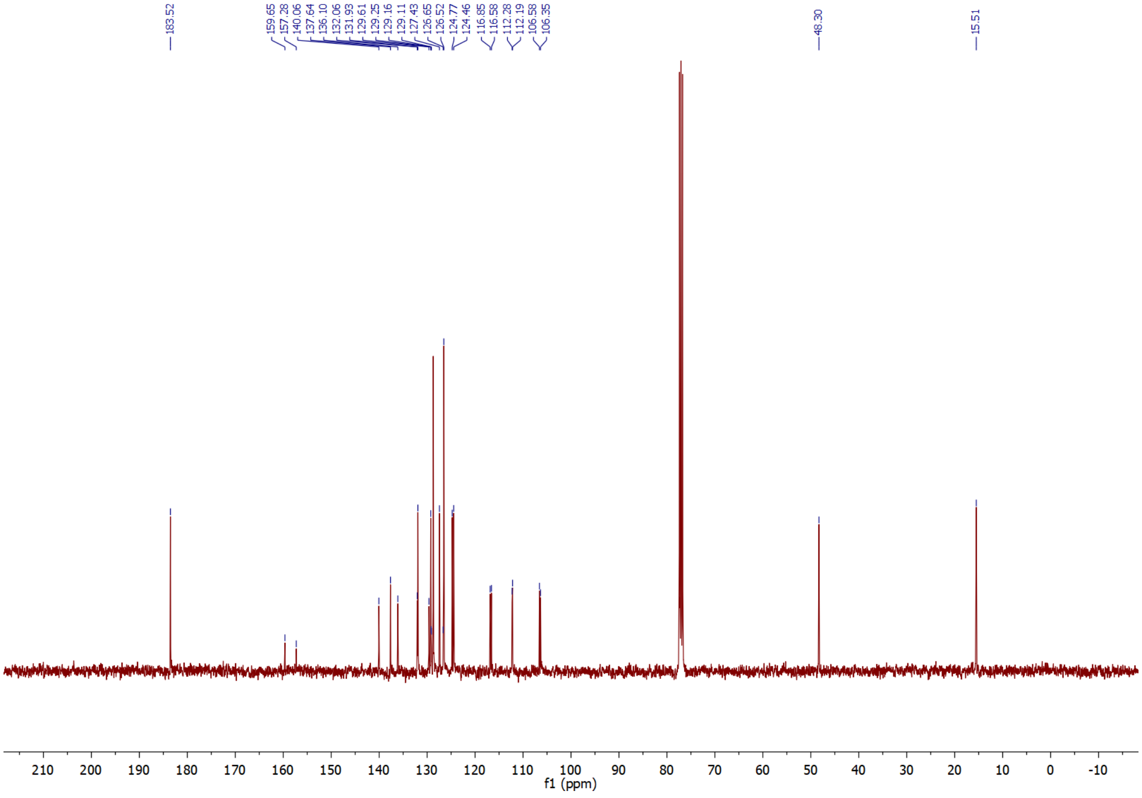

Figure S63. ^{19}F-NMR (376 MHz, $CDCl_3$) of **1j**:

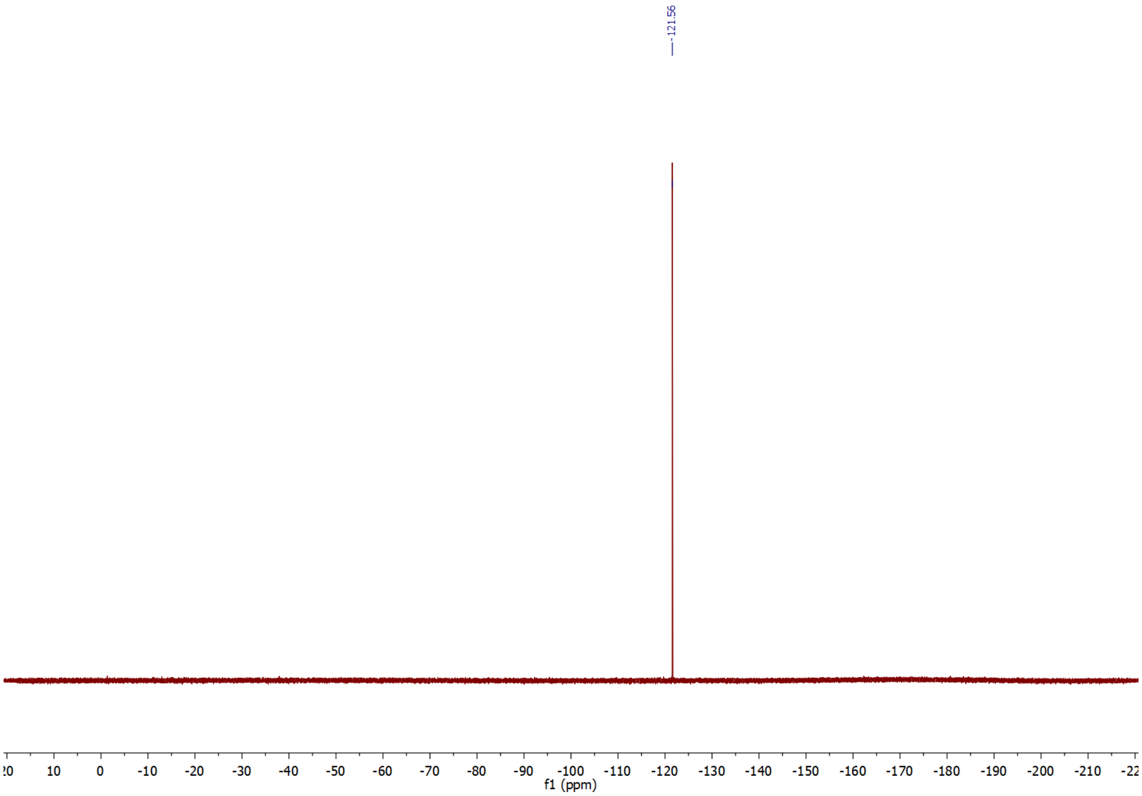

Figure S64. ^{1}H-NMR (400 MHz, $CDCl_3$) of **1k**:

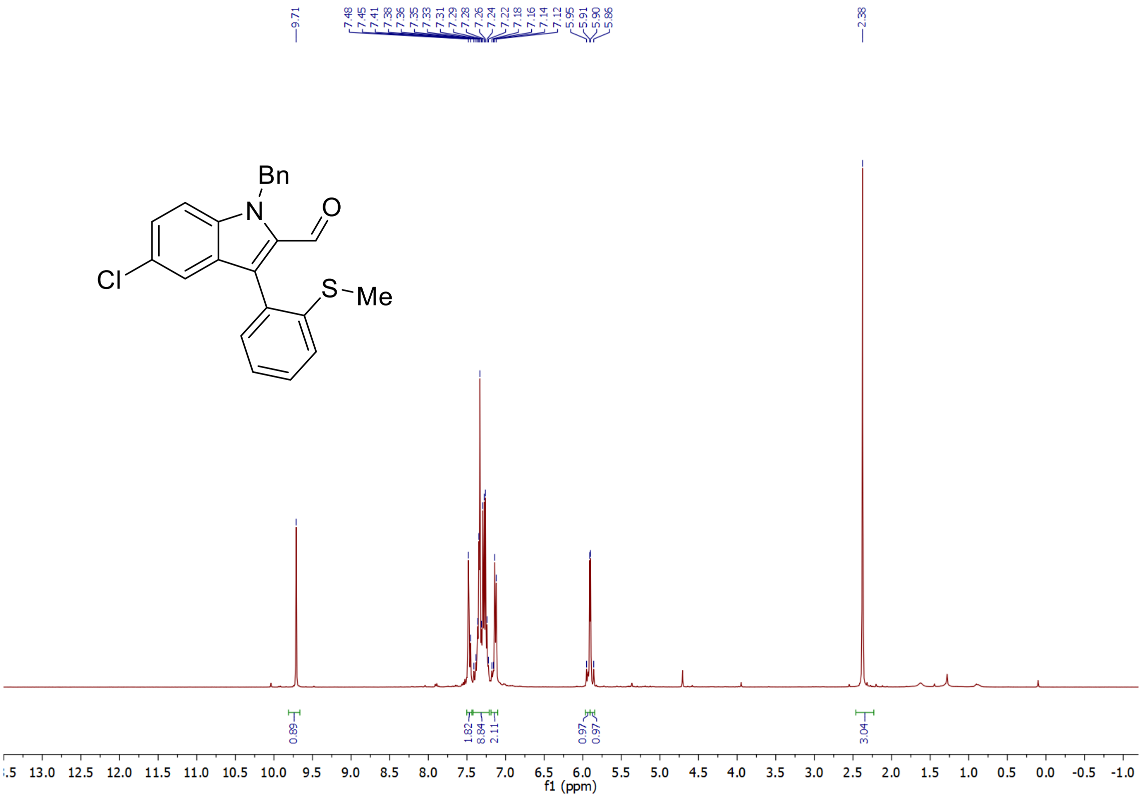

Figure S65. ^{13}C-NMR (100 MHz, $CDCl_3$) of **1k**:

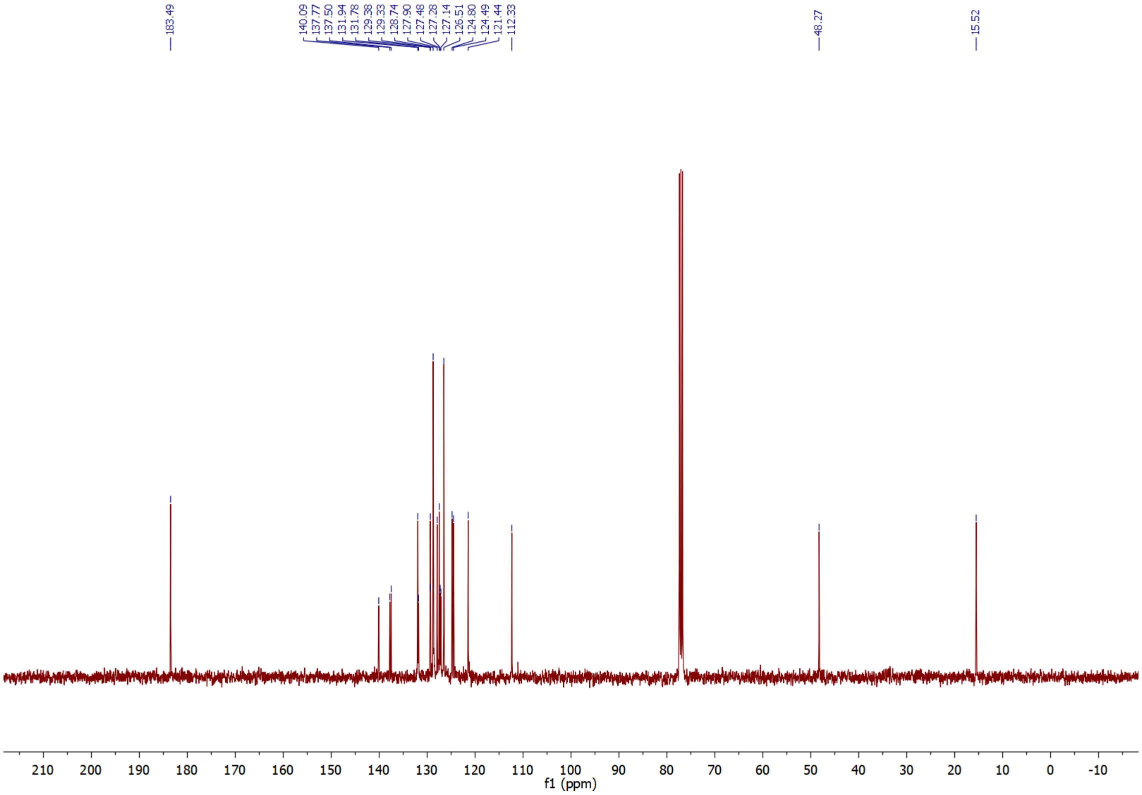

Figure S66. ^{1}H-NMR (400 MHz, $CDCl_3$) of **1l**:

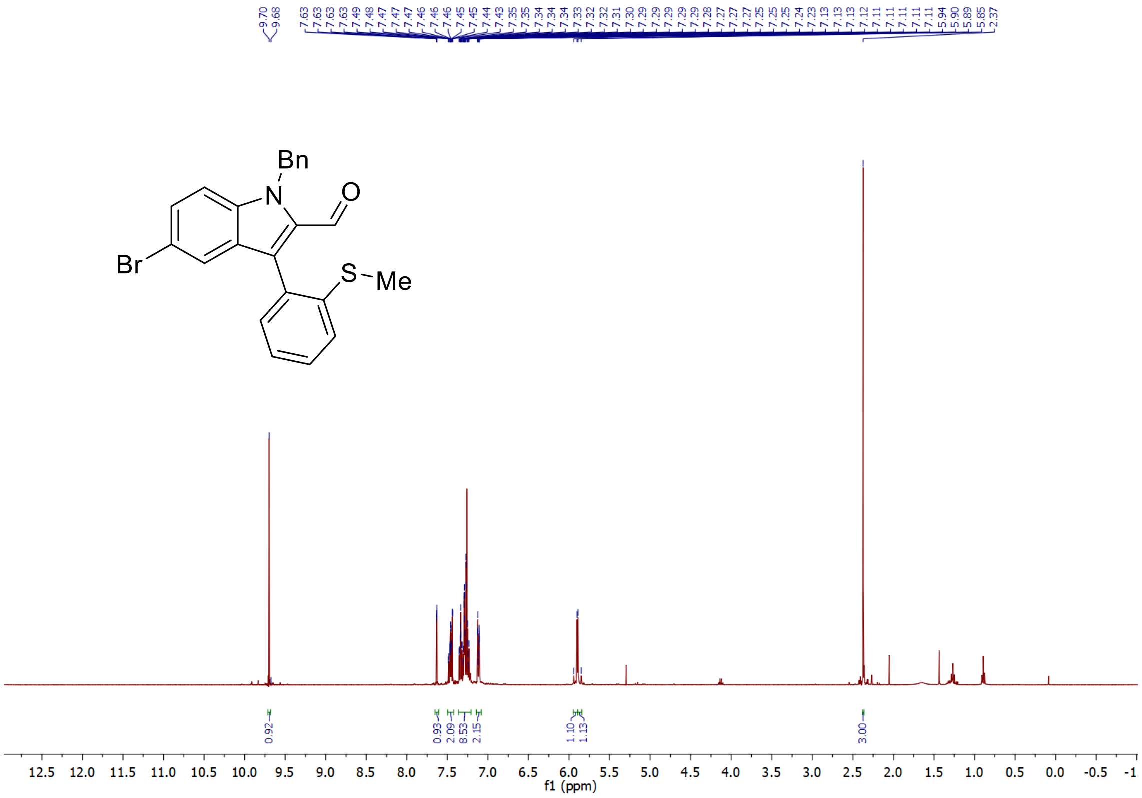

Figure S67. ^{13}C-NMR (100 MHz, $CDCl_3$) of **1l**:

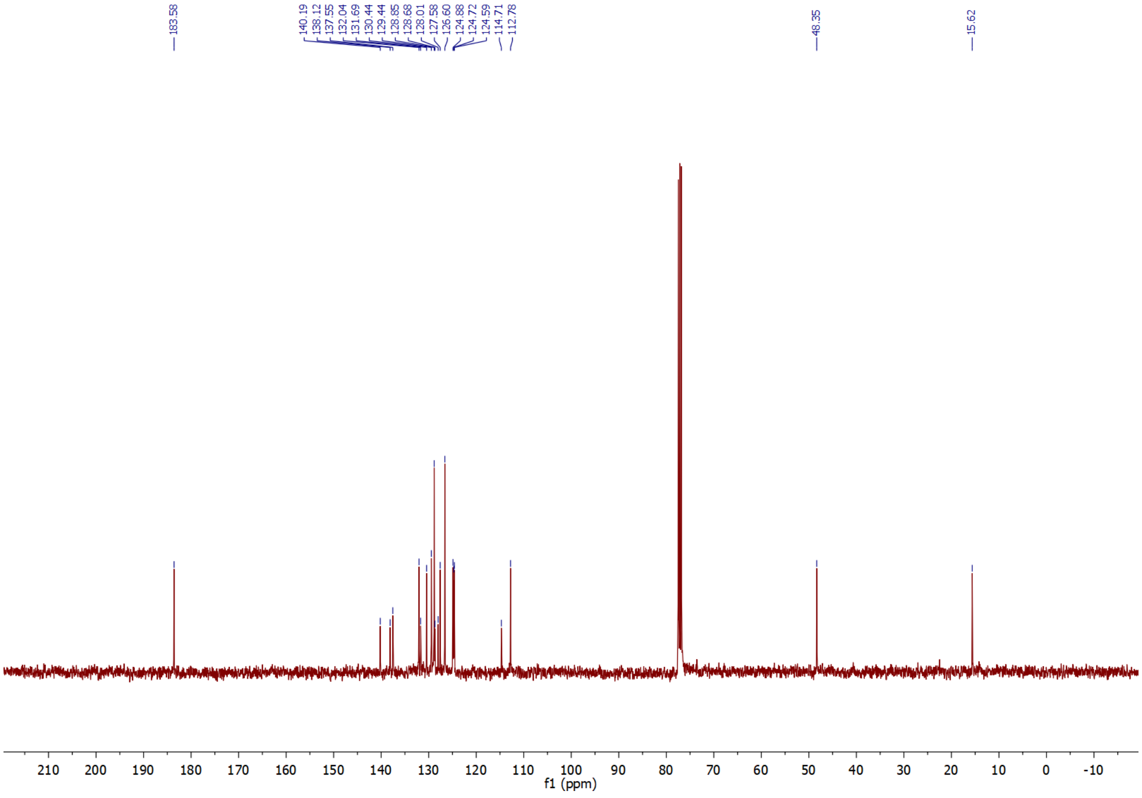

Figure S68. ^{1}H-NMR (400 MHz, $CDCl_3$) of **1m**:

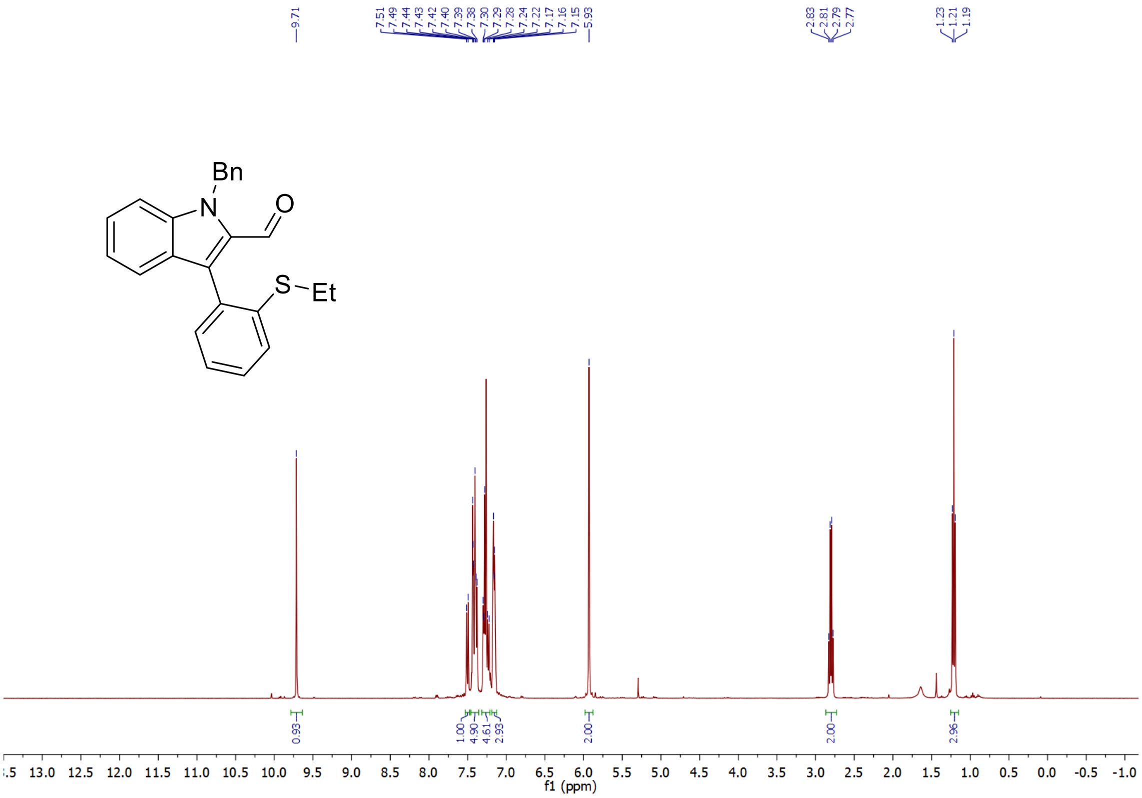

Figure S69. ^{13}C-NMR (100 MHz, $CDCl_3$) of **1m**:

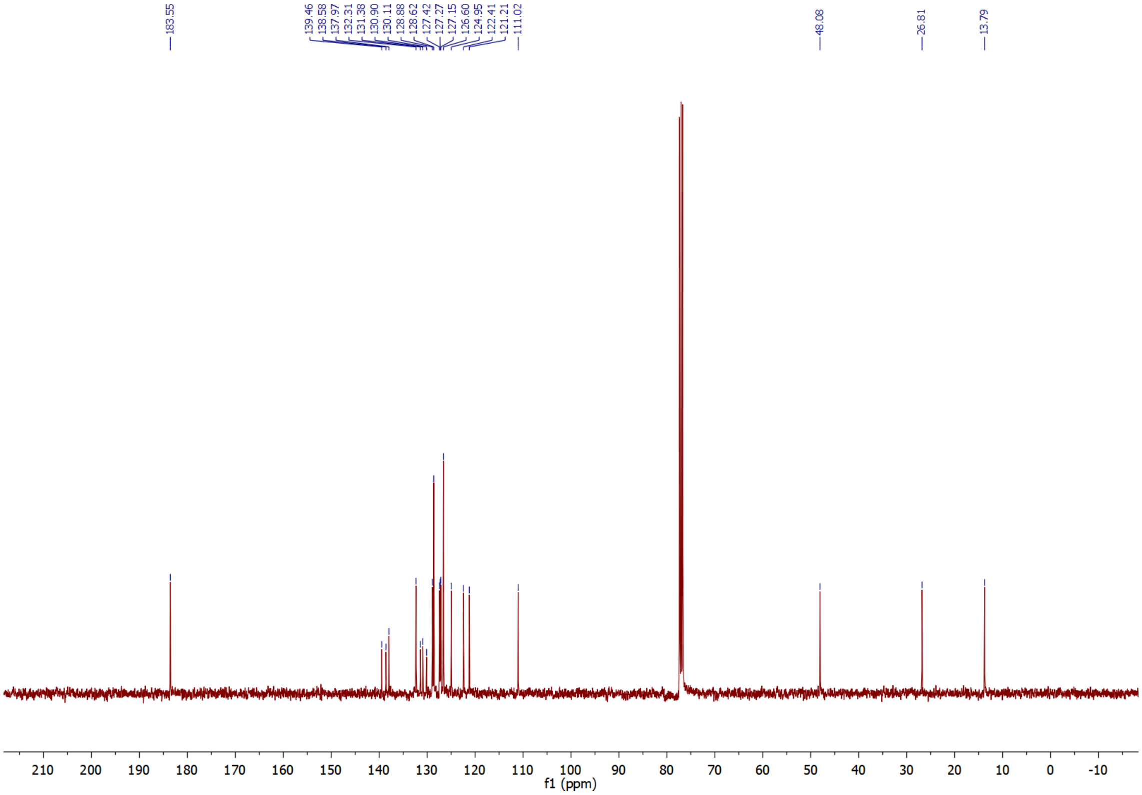

Figure S70. ^{1}H-NMR (400 MHz, $CDCl_3$) of **1o**:

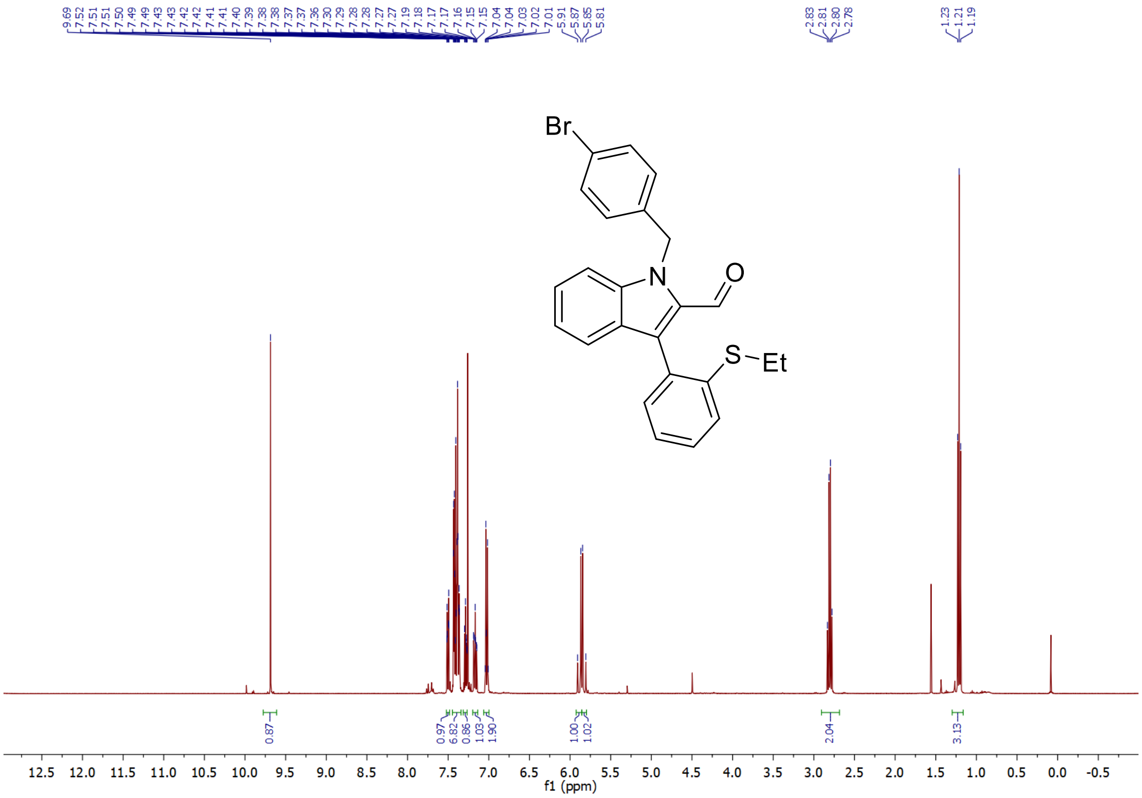

Figure S71. ^{13}C-NMR (100 MHz, $CDCl_3$) of **1o**:

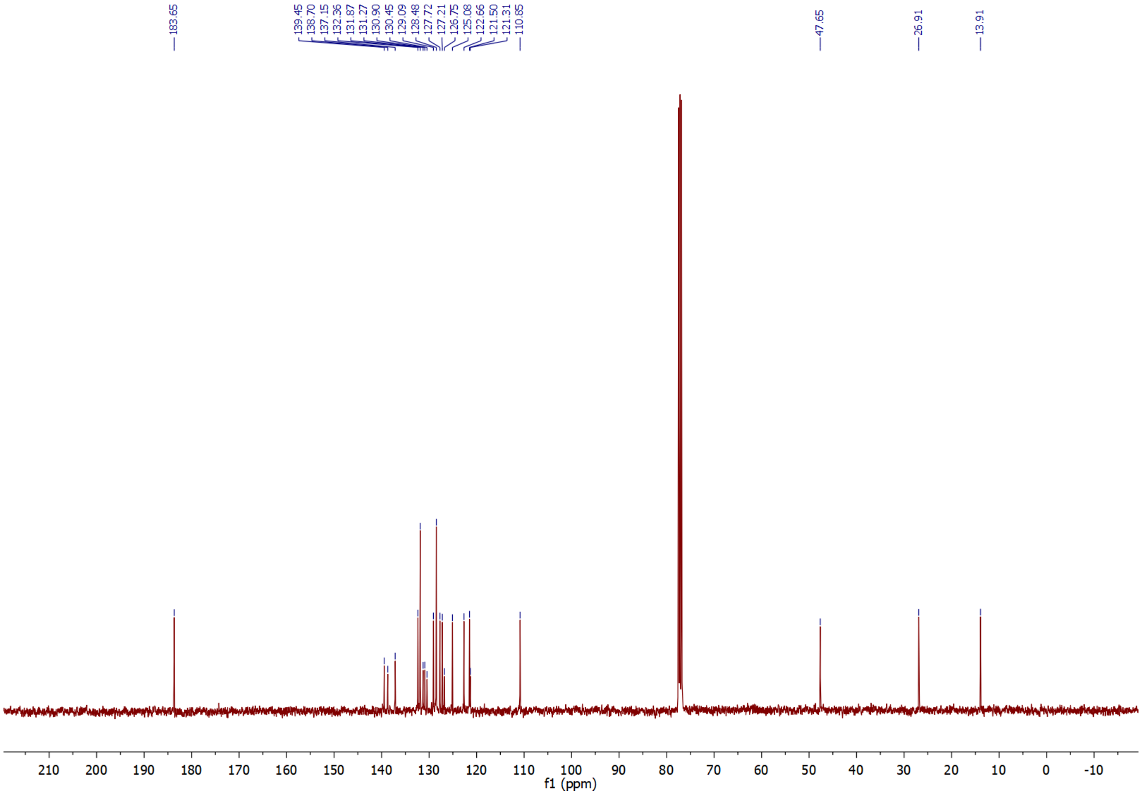

Figure S72. ^{1}H-NMR (400 MHz, $CDCl_3$) of **1p**:

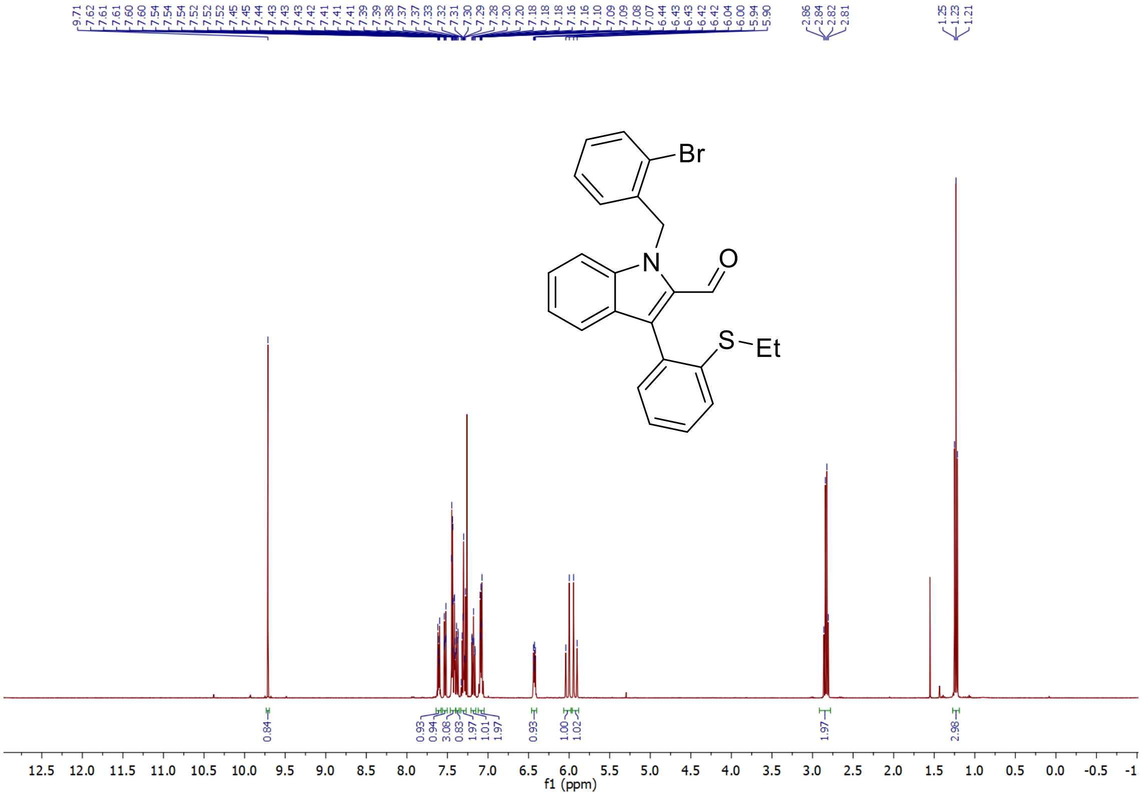

Figure S73. ^{13}C-NMR (100 MHz, $CDCl_3$) of **1p**:

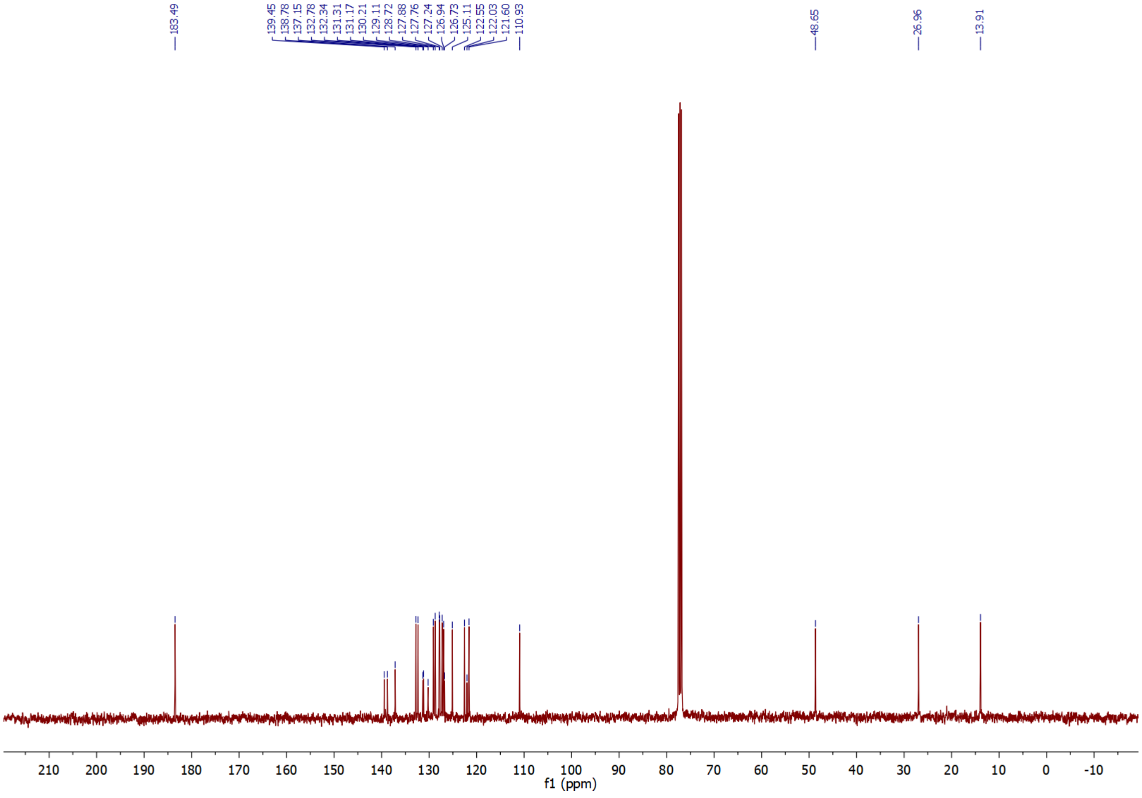

Figure S74. ^{1}H-NMR (400 MHz, $CDCl_3$) of **1q**:

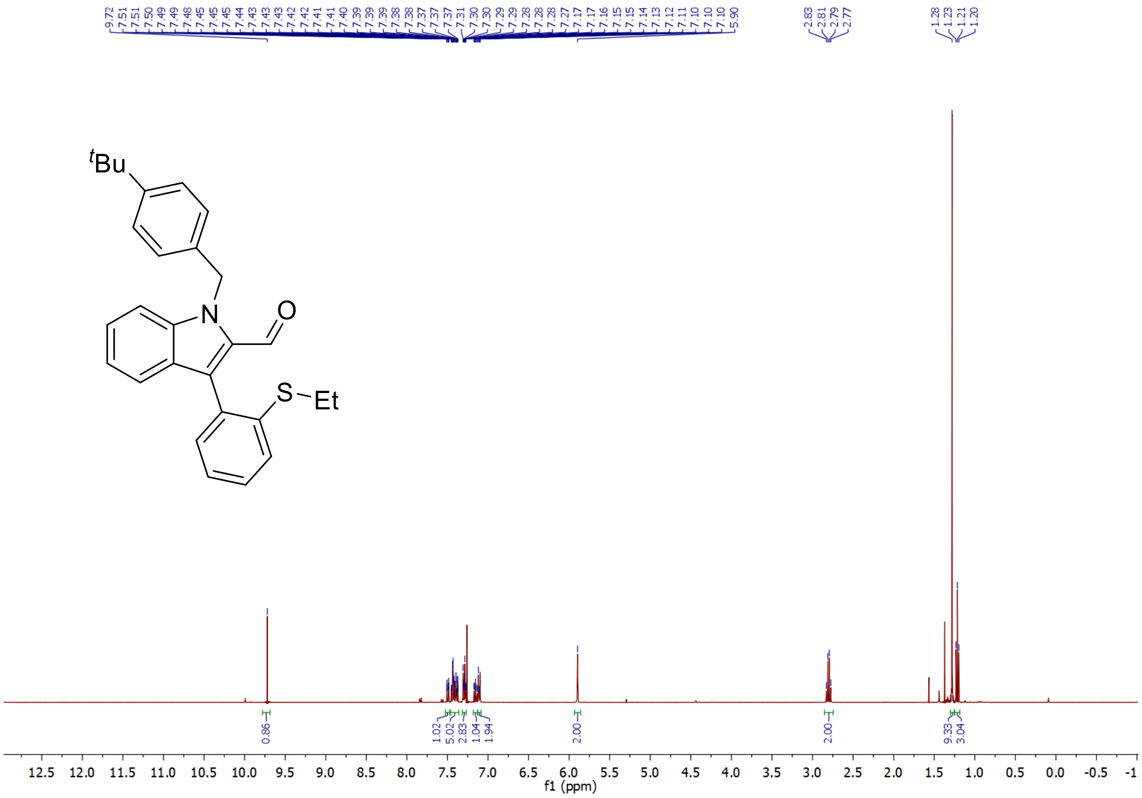

Figure S75. ^{13}C-NMR (100 MHz, $CDCl_3$) of **1q**:

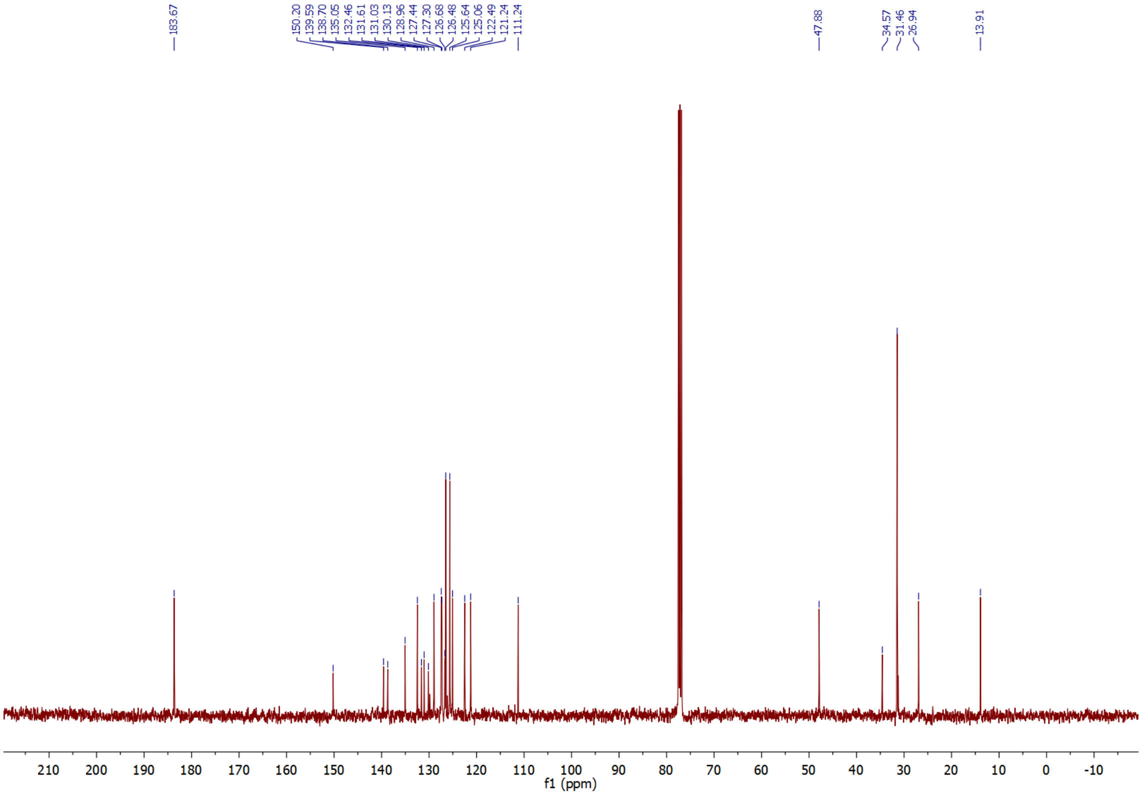

Figure S76. ^{1}H-NMR (400 MHz, $CDCl_3$) of **1r**:

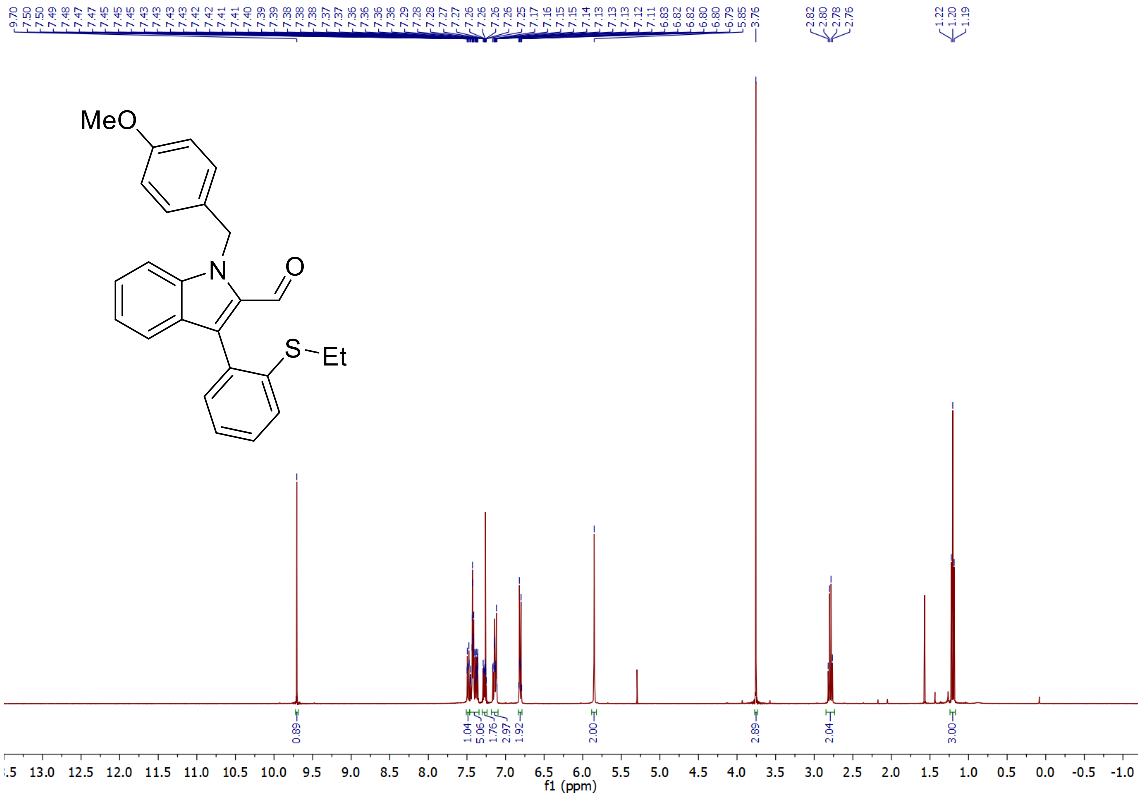

Figure S77. ^{13}C-NMR (100 MHz, $CDCl_3$) of **1r**:

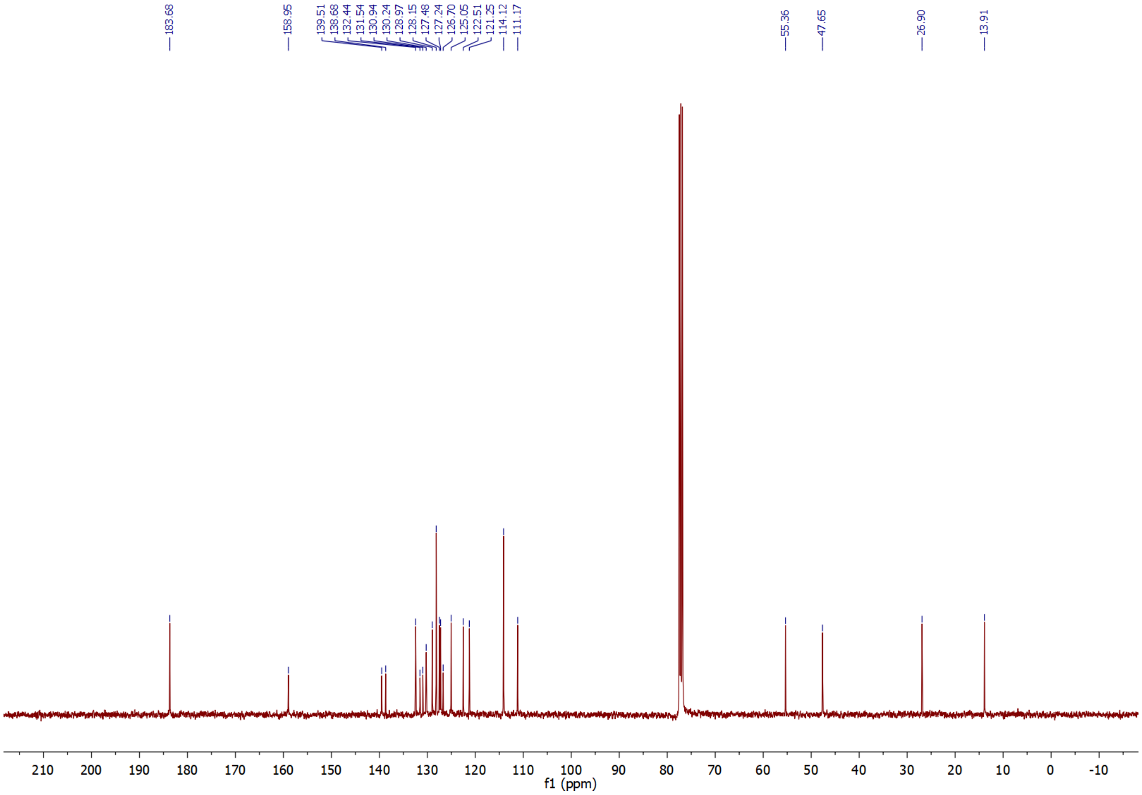

Figure S78. ^{1}H-NMR (400 MHz, $CDCl_3$) of **1s**:

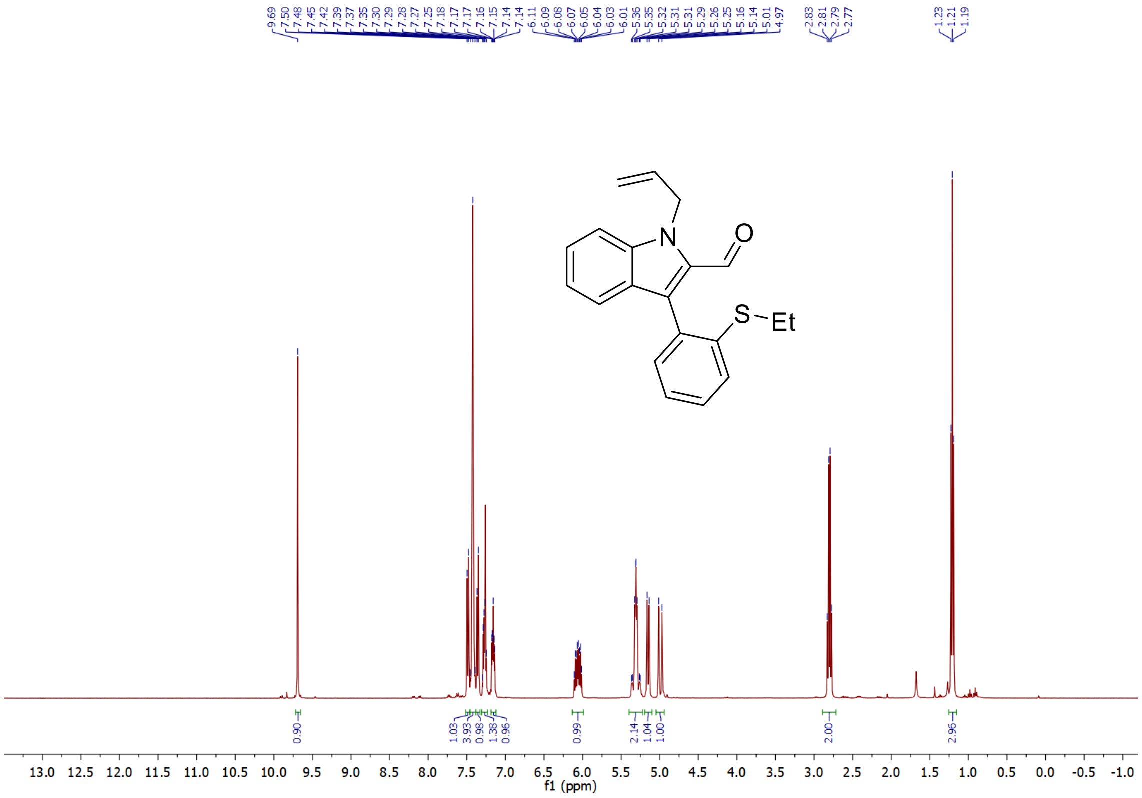

Figure S79. ^{13}C-NMR (100 MHz, $CDCl_3$) of **1s**:

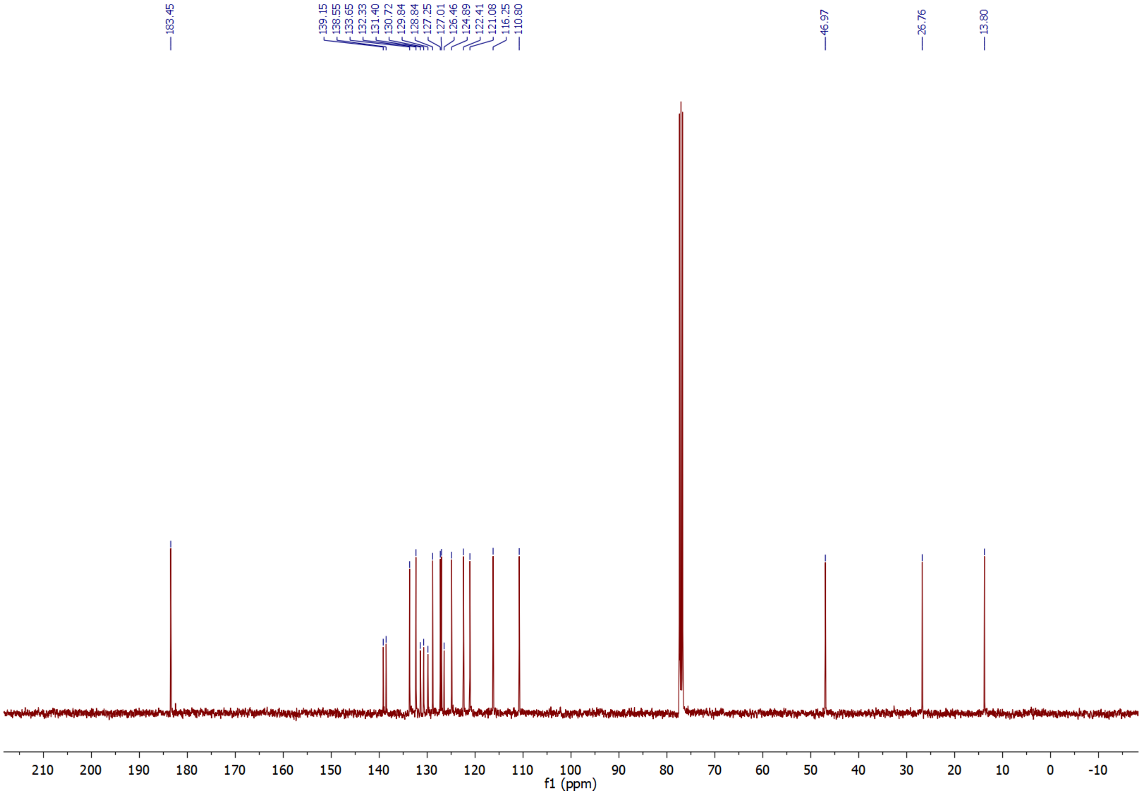

Figure S80. ^{1}H-NMR (400 MHz, $CDCl_3$) of **1t**:

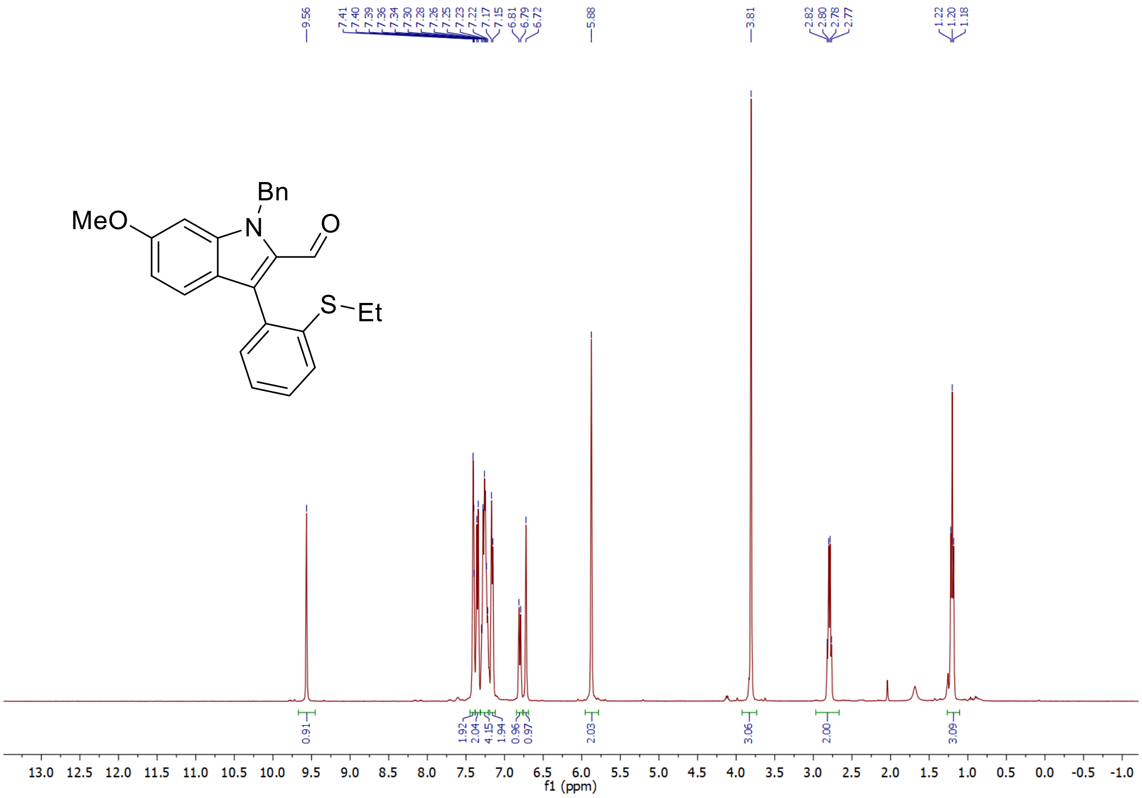

Figure S81. ^{13}C-NMR (100 MHz, $CDCl_3$) of **1t**:

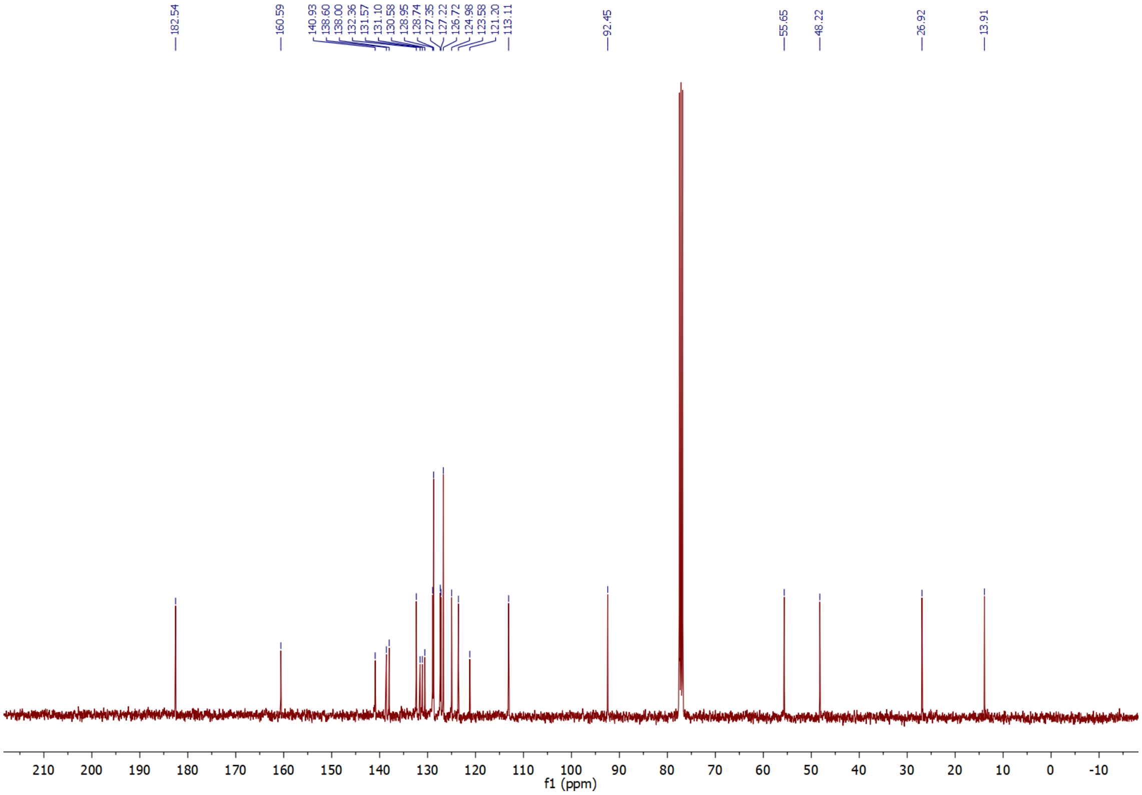

Figure S82. ^{1}H-NMR (400 MHz, $CDCl_3$) of **1u**:

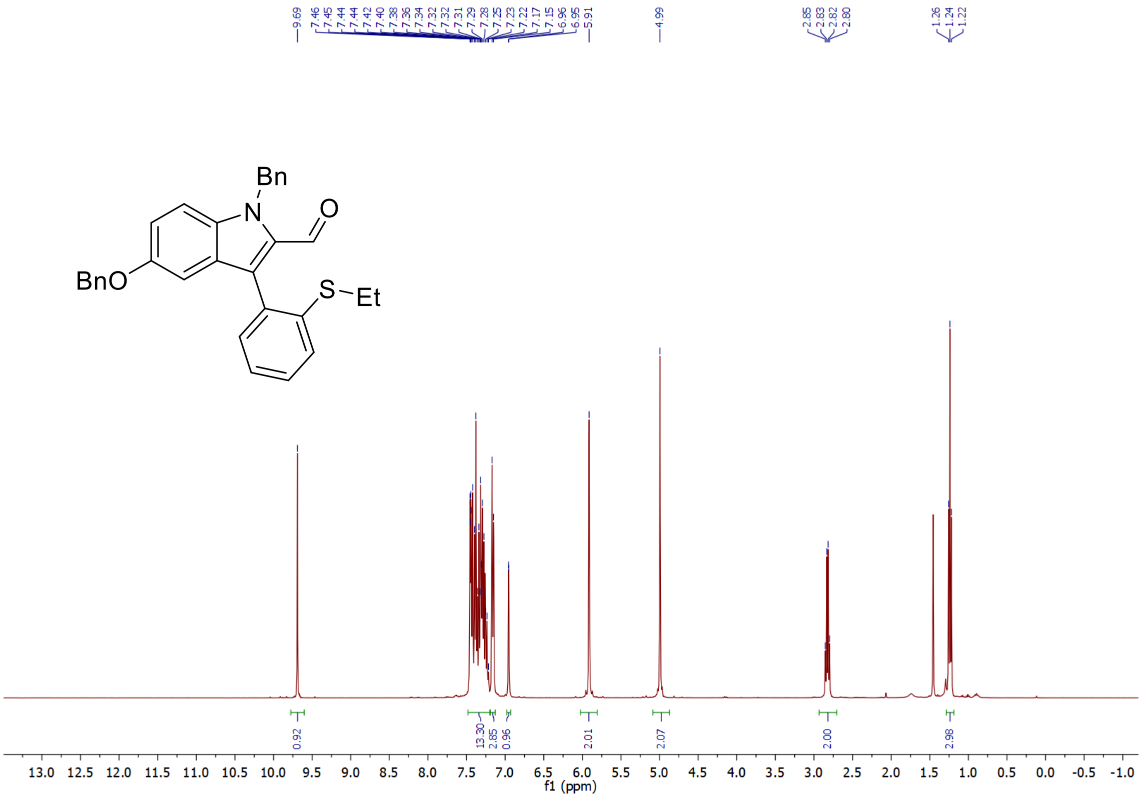

Figure S83. ^{13}C-NMR (100 MHz, $CDCl_3$) of **1u**:

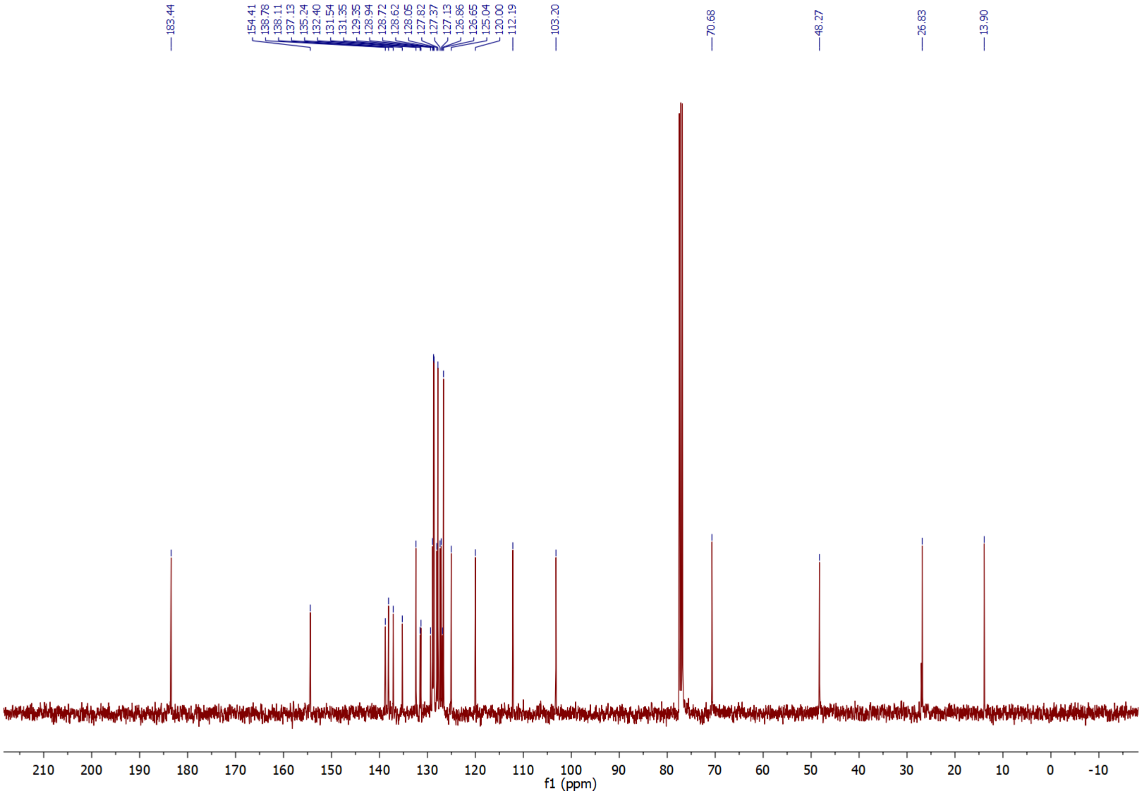

Figure S84. ^{1}H-NMR (400 MHz, $CDCl_3$) of **1v**:

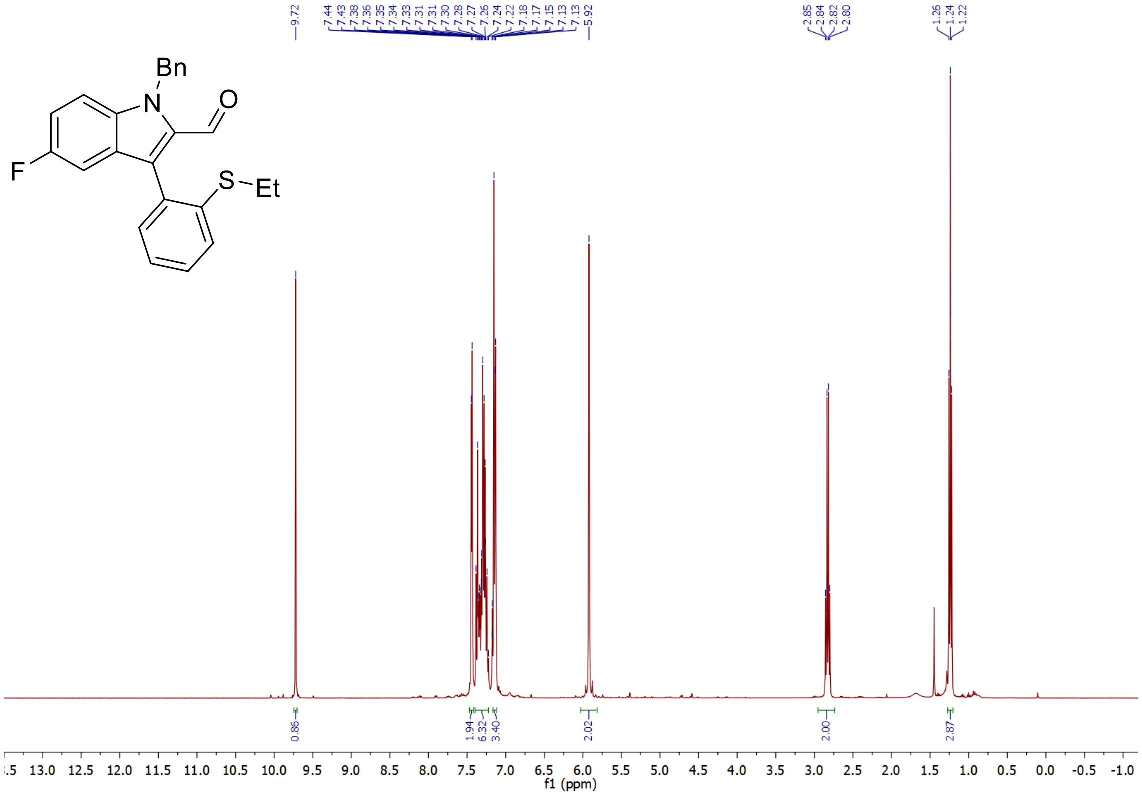

Figure S85. ^{13}C-NMR (100 MHz, $CDCl_3$) of **1v**:

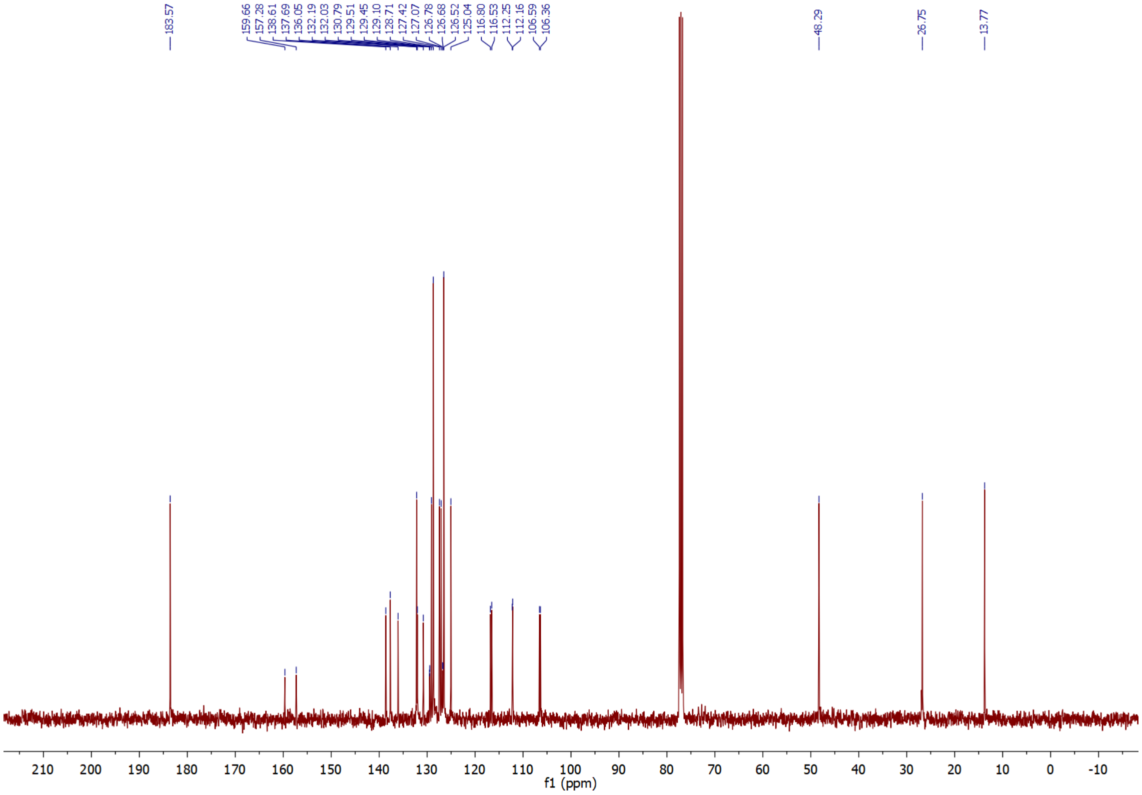

Figure S86. ^{19}F-NMR (376 MHz, $CDCl_3$) of **1v**:

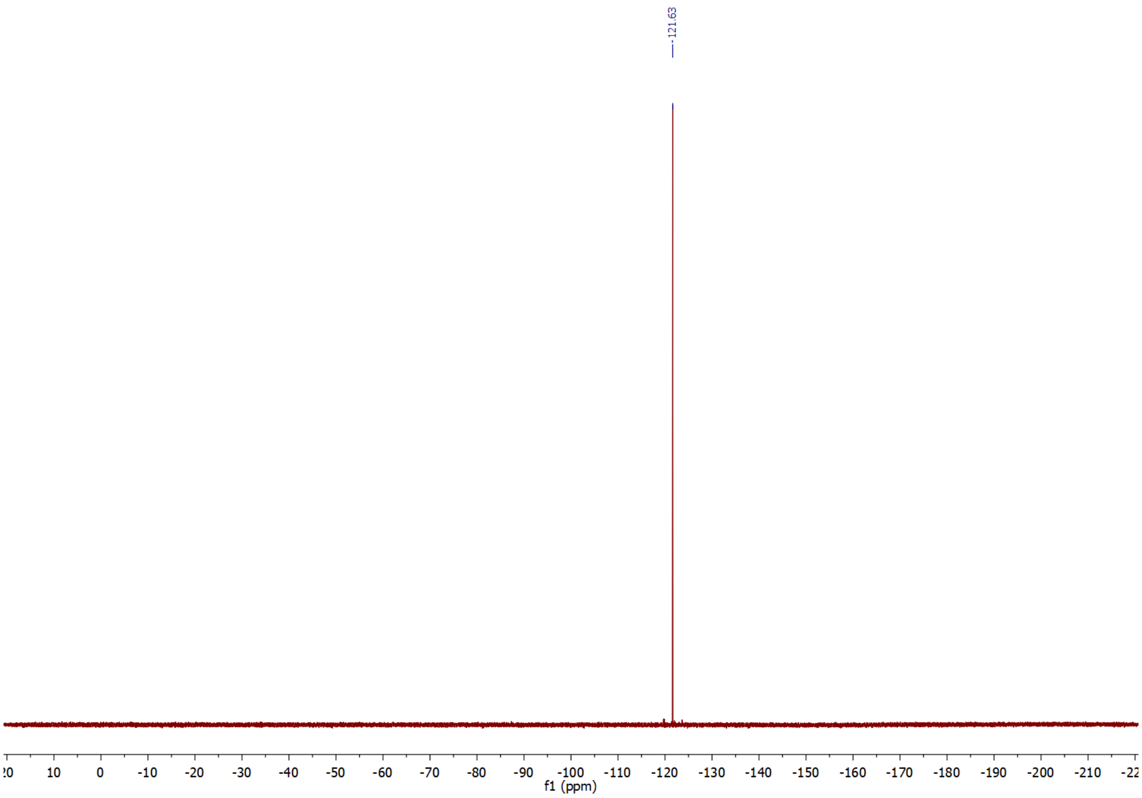

Figure S87. ^{1}H-NMR (400 MHz, $CDCl_3$) of **1x**:

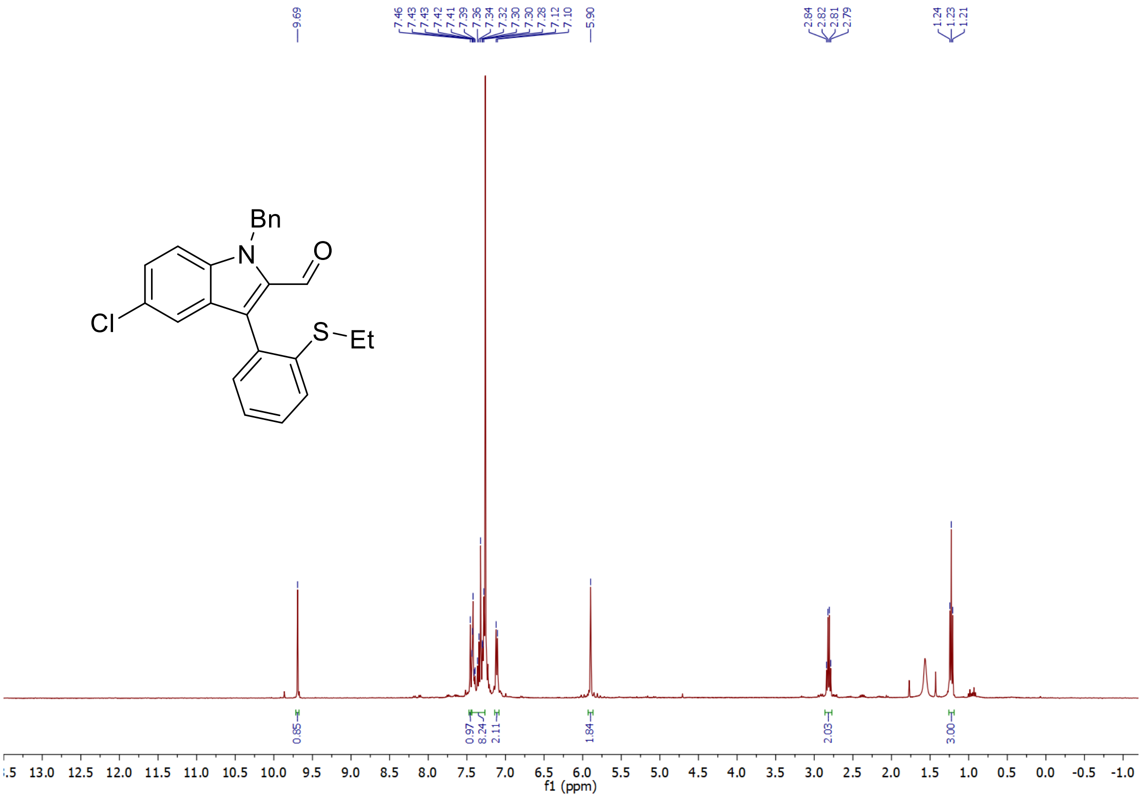

Figure S88. ^{13}C-NMR (100 MHz, $CDCl_3$) of **1x**:

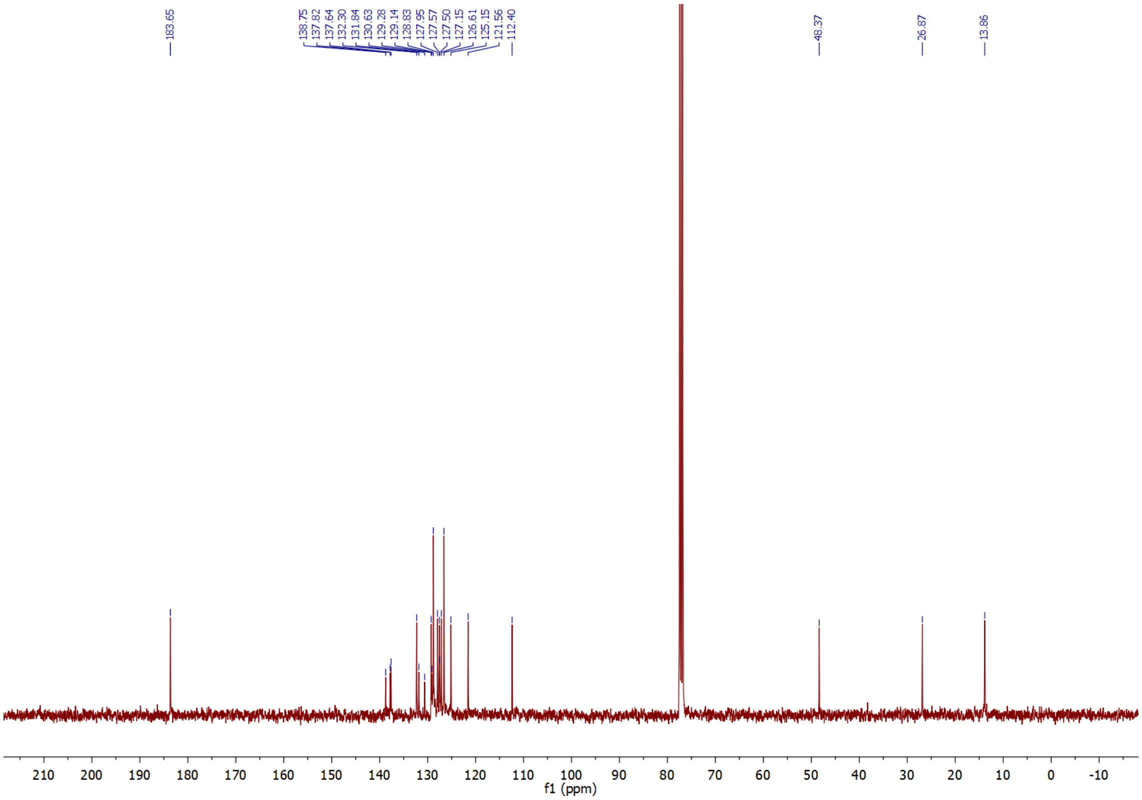

Figure S89. ^{1}H-NMR (400 MHz, $CDCl_3$) of **1y**:

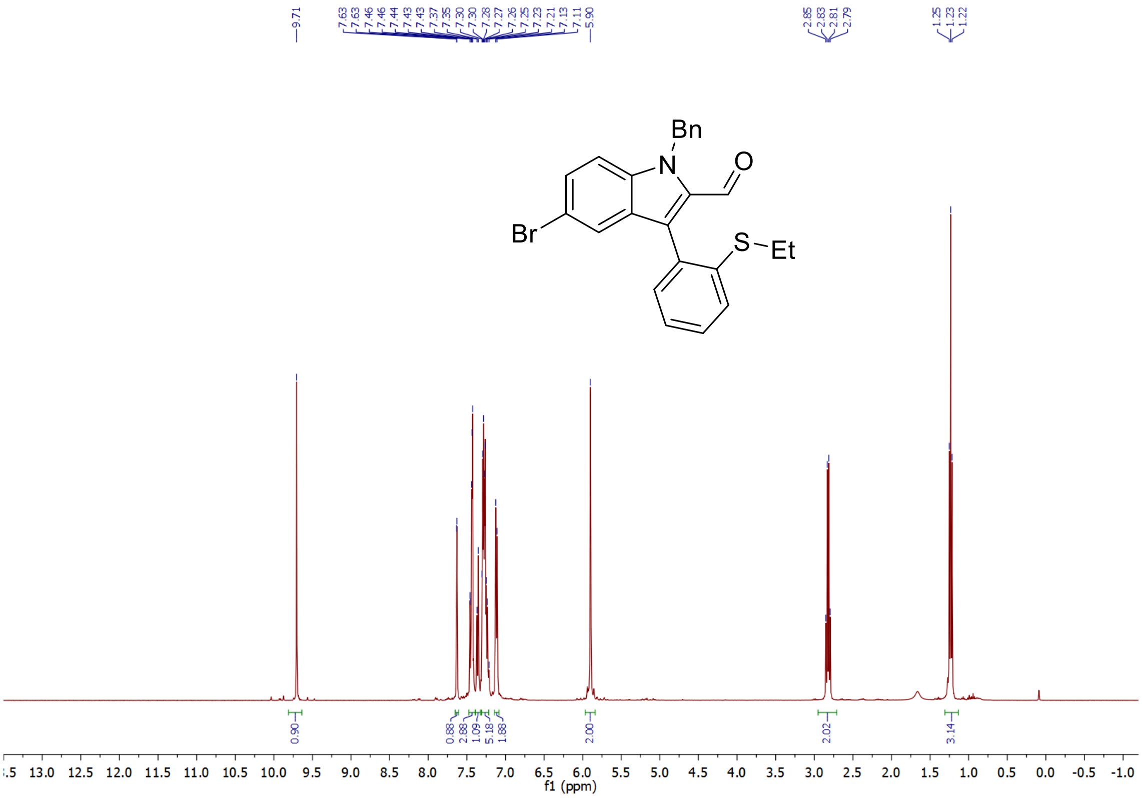

Figure S90. ^{13}C-NMR (100 MHz, $CDCl_3$) of **1y**:

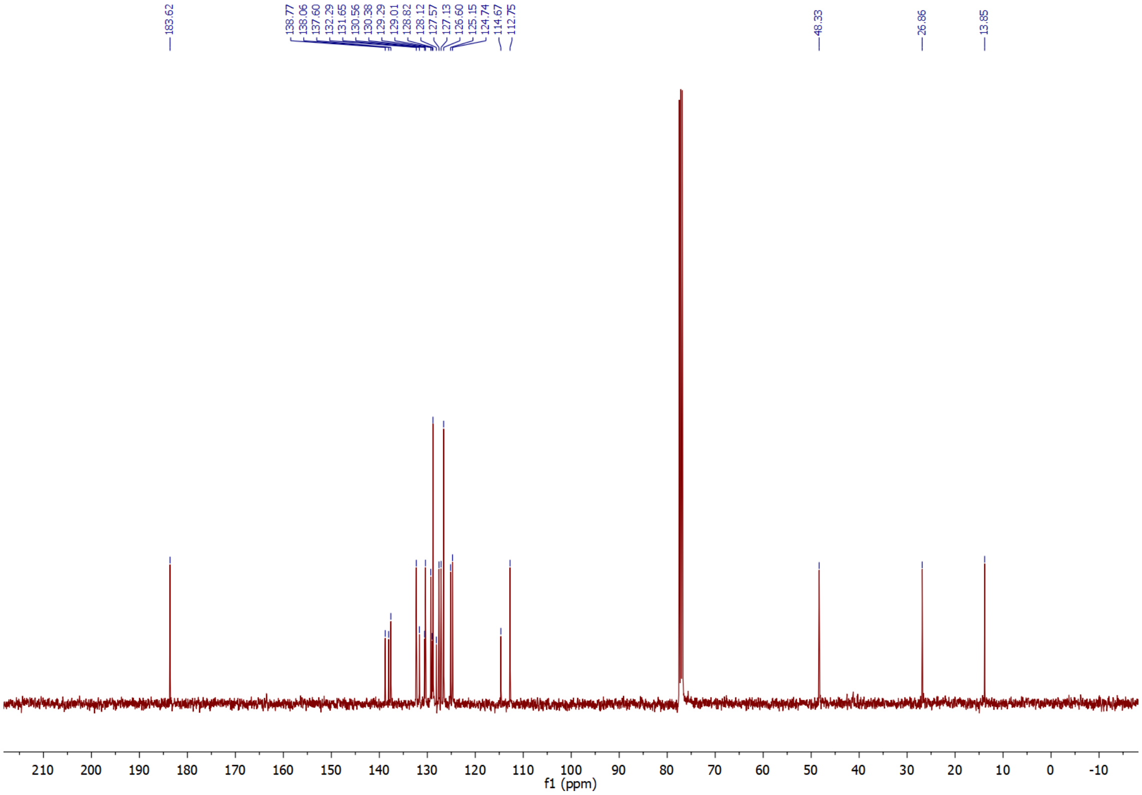

Figure S91. ^{1}H-NMR (400 MHz, $CDCl_3$) of **1z**:

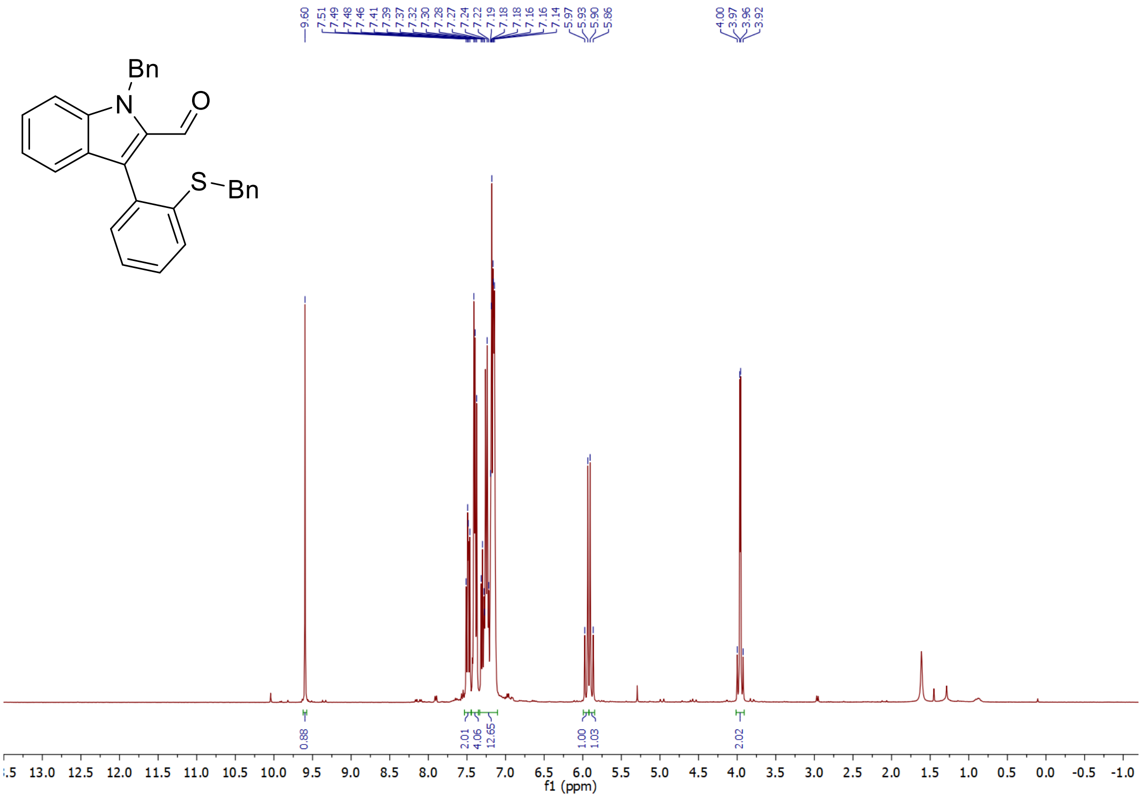

Figure S92. ^{13}C-NMR (100 MHz, $CDCl_3$) of **1z**:

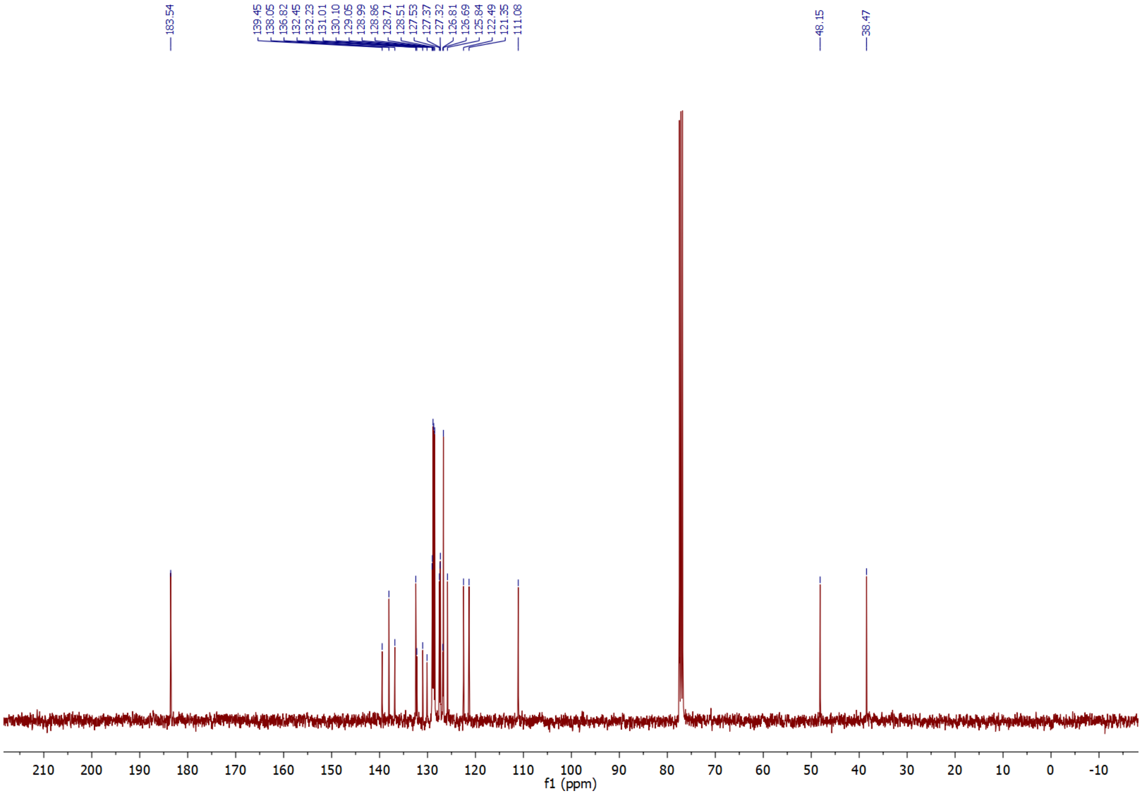

Figure S93. ^{1}H-NMR (400 MHz, $CDCl_3$) of **1ab**:

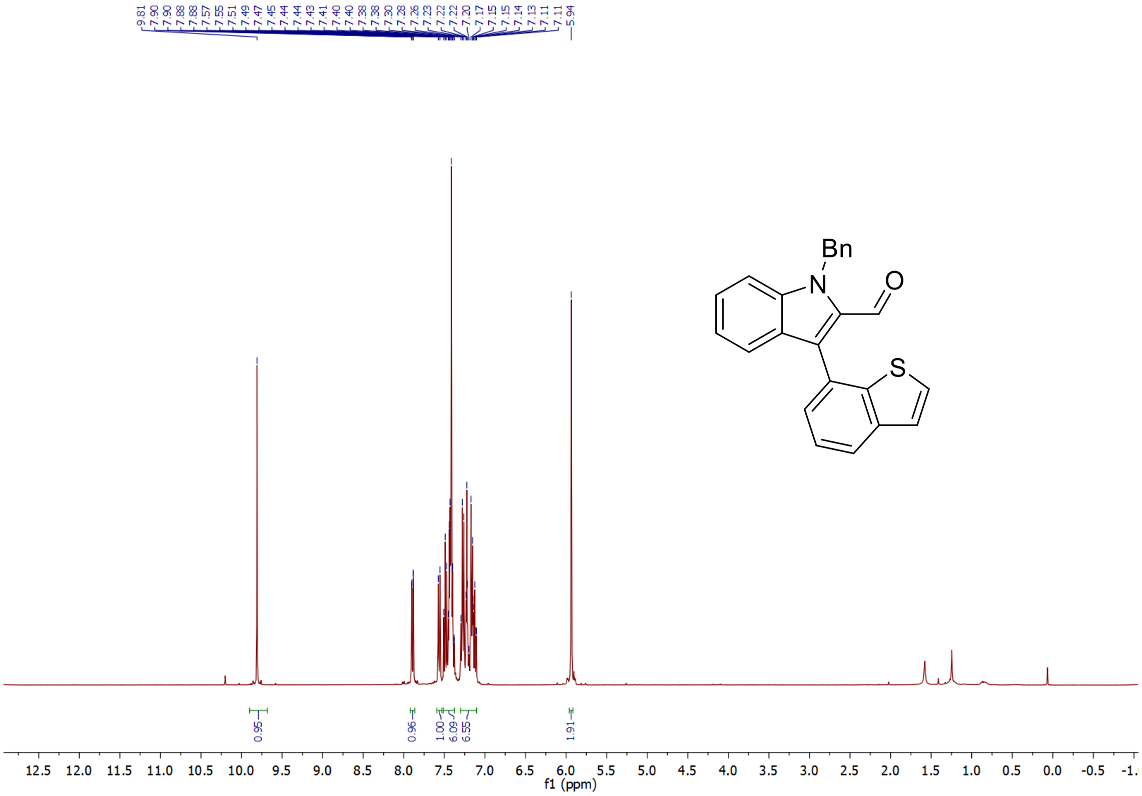

Figure S94. ^{13}C-NMR (100 MHz, $CDCl_3$) of **1ab**:

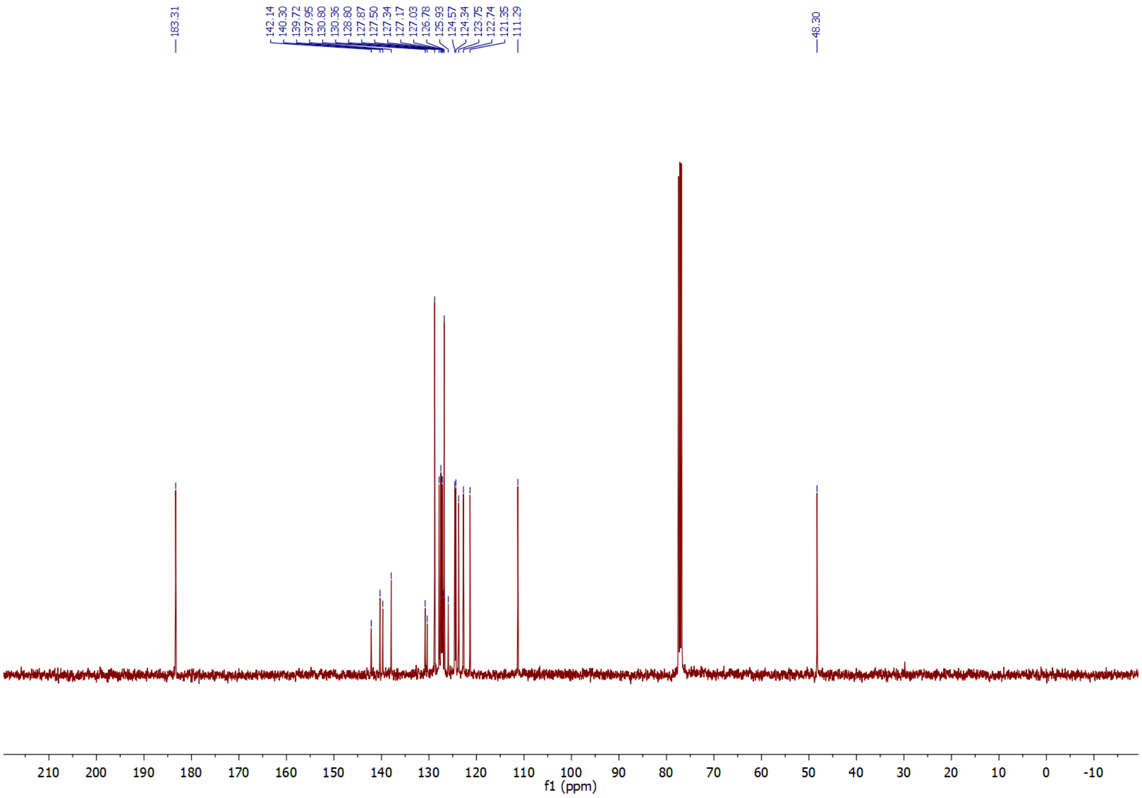

Figure S95. ^{1}H-NMR (400 MHz, $CDCl_3$) of **1ac**:

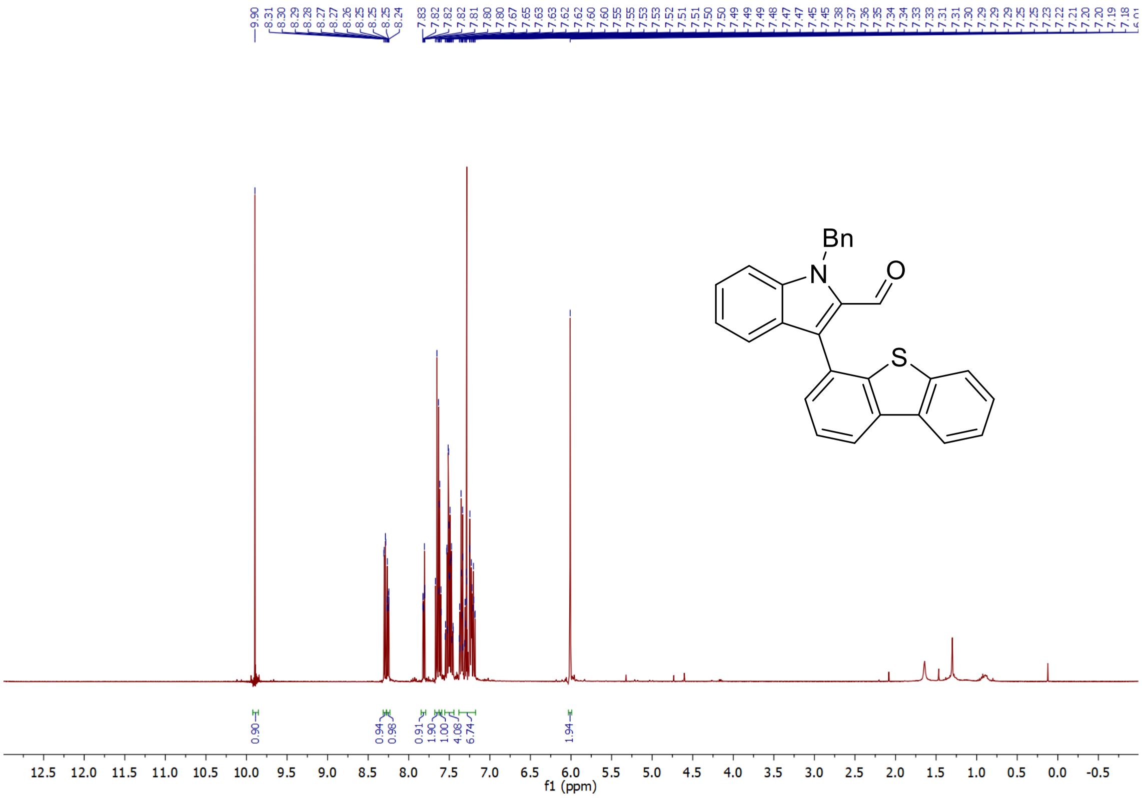

Figure S96. ^{13}C-NMR (100 MHz, $CDCl_3$) of **1ac**:

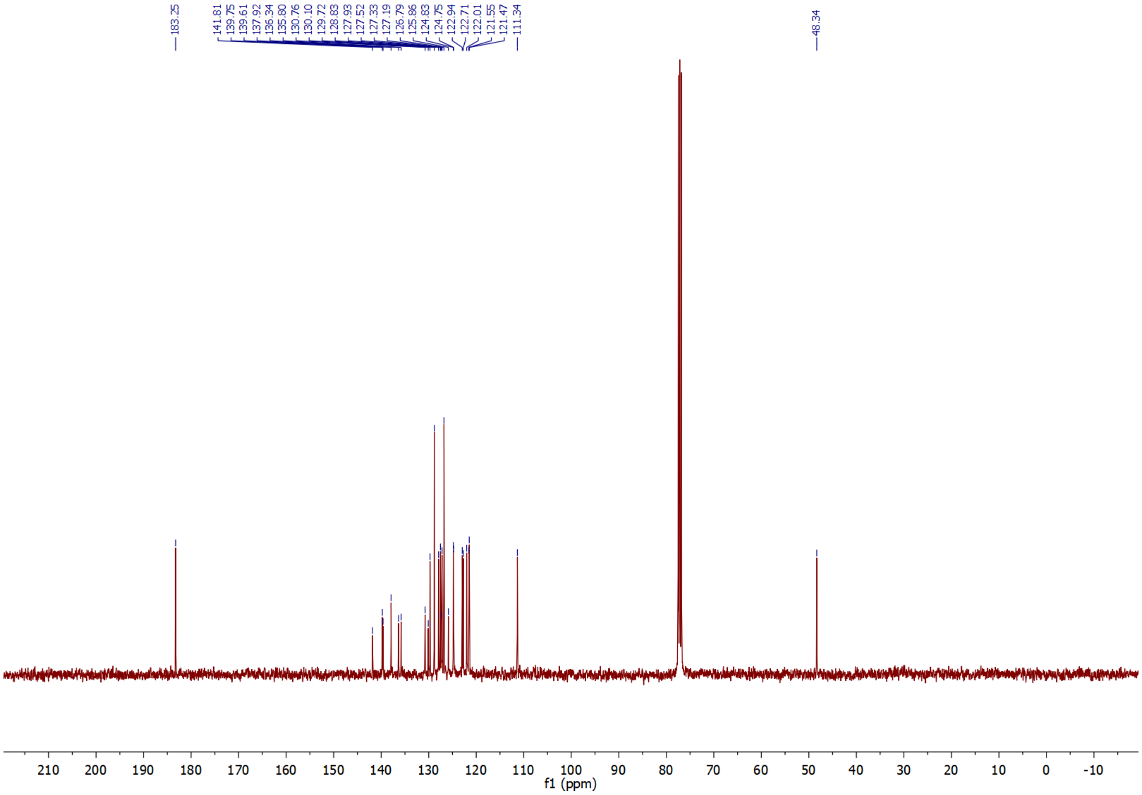

Figure S97. ^{1}H-NMR (400 MHz, $CDCl_3$) of **(2*S*,3*R*,*S*$_a$)-2a**:

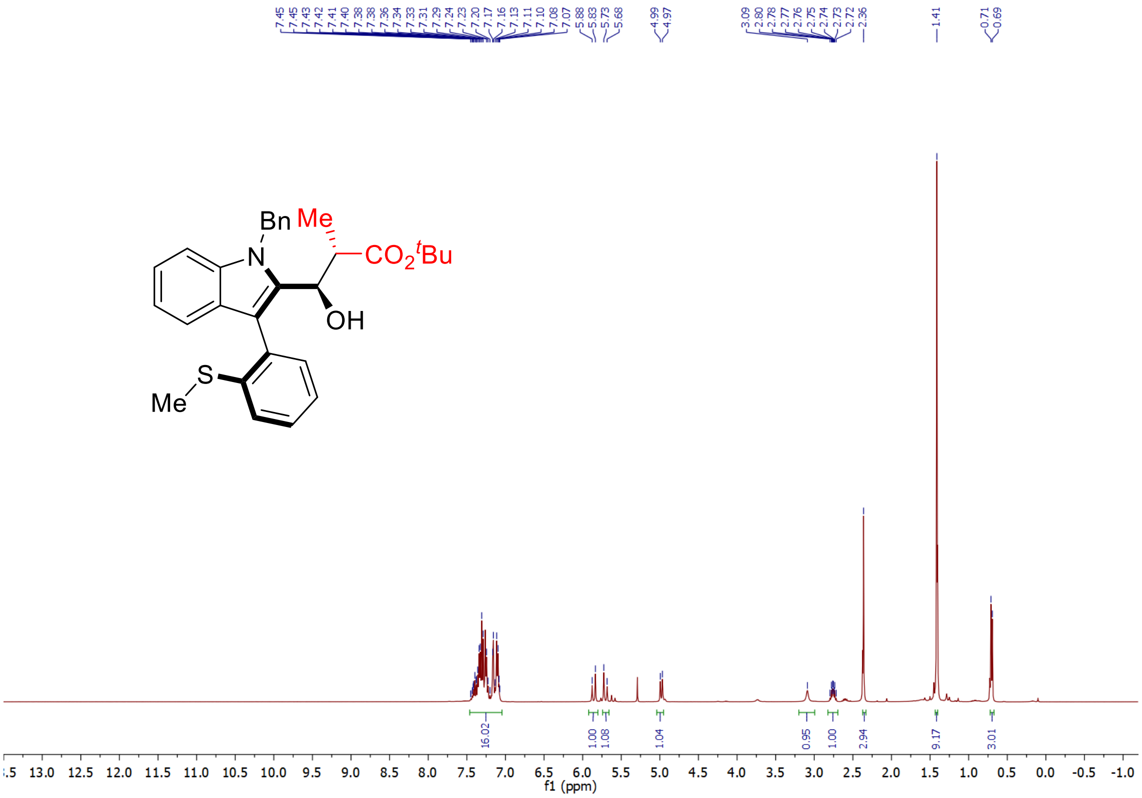

Figure S98. ^{13}C-NMR (100 MHz, $CDCl_3$) of **(2*S*,3*R*,*S*$_a$)-2a**:

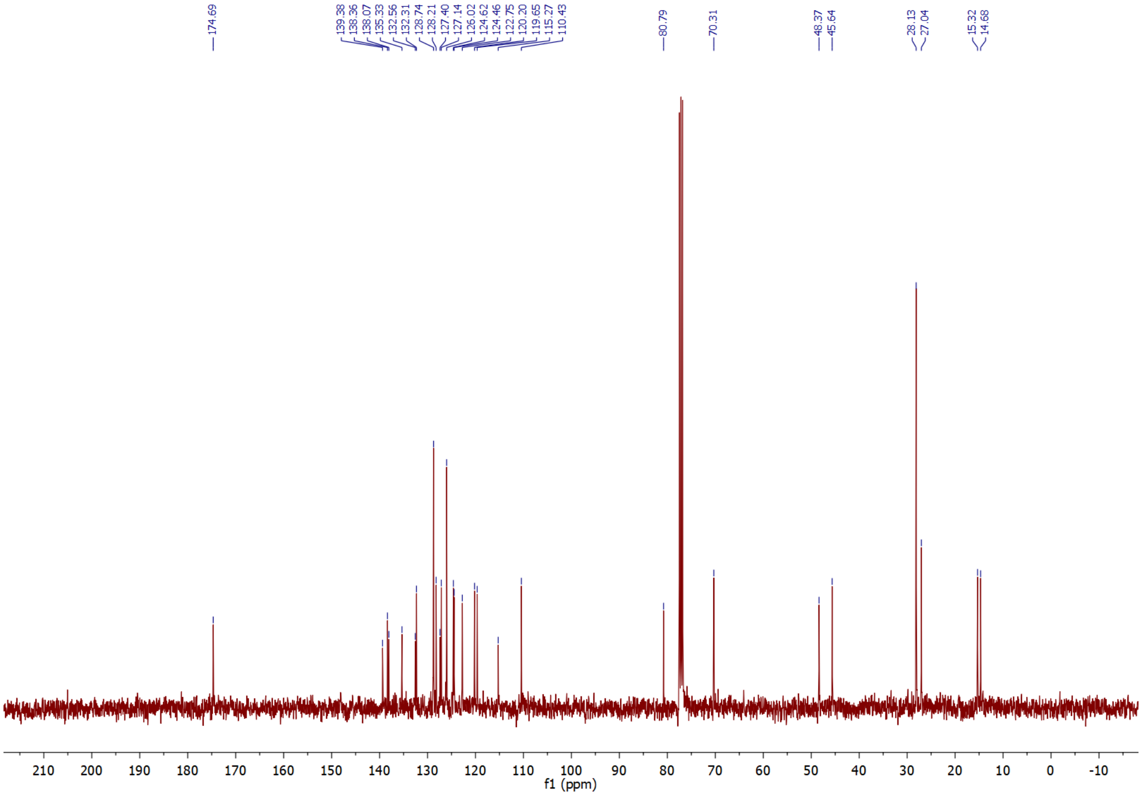

Figure S99. Racemic sample of 2a: IA column, n-Hex/i-PrOH 95:5, T= 30°C, F= 1.0 mL/min.

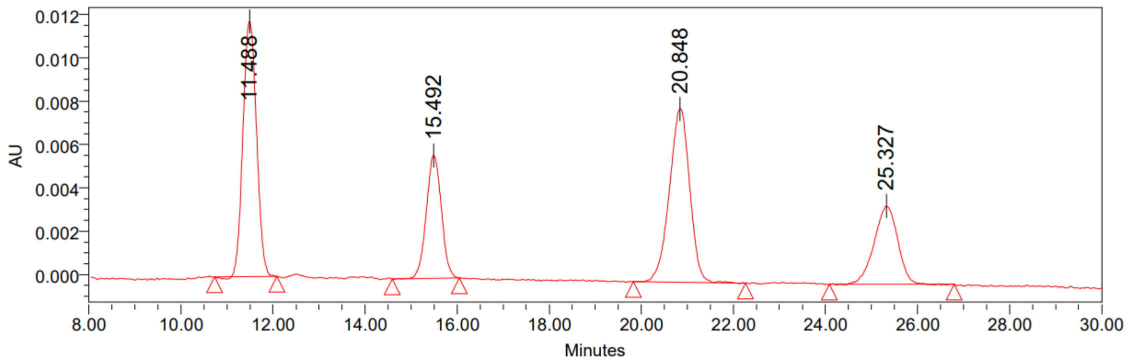

Processed Channel: PDA 288.9 nm

	Processed Channel	Retention Time (min)	Area	% Area	Height
1	PDA 288.9 nm	11.488	241884	32.45	11791
2	PDA 288.9 nm	15.492	129085	17.32	5675
3	PDA 288.9 nm	20.848	245254	32.90	7998
4	PDA 288.9 nm	25.327	129126	17.32	3613

Figure S100. Enantioenriched sample of (2*S*,3*R*,*S*$_a$)-2a: IA column, n-Hex/i-PrOH 95:5, T= 30°C, F= 1.0 mL/min.

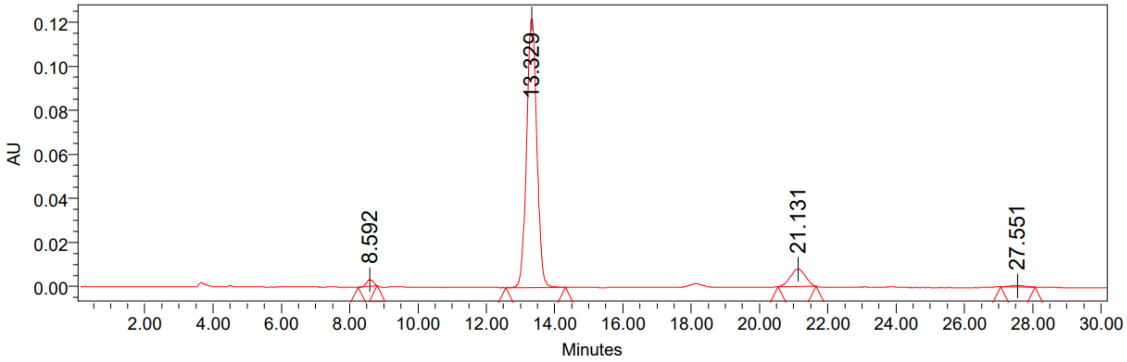

Processed Channel: PDA 289.4 nm

	Processed Channel	Retention Time (min)	Area	% Area	Height
1	PDA 289.4 nm	8.592	44448	1.56	3113
2	PDA 289.4 nm	13.329	2537634	89.25	122258
3	PDA 289.4 nm	21.131	243109	8.55	7929
4	PDA 289.4 nm	27.551	18197	0.64	525

Figure S101. ^{1}H-NMR (400 MHz, $CDCl_3$) of **(2*S*,3*R*,*S*$_a$)-2b**:

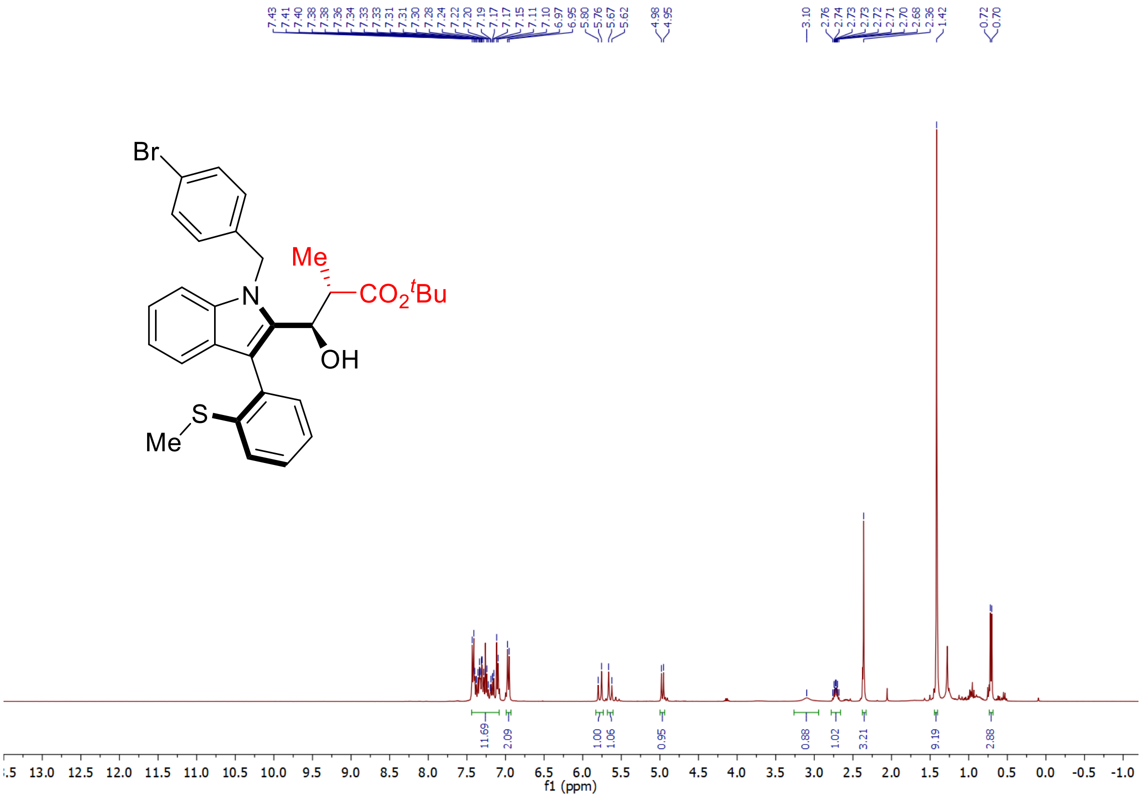

Figure S102. ^{13}C-NMR (100 MHz, $CDCl_3$) of **(2*S*,3*R*,*S*$_a$)-2b**:

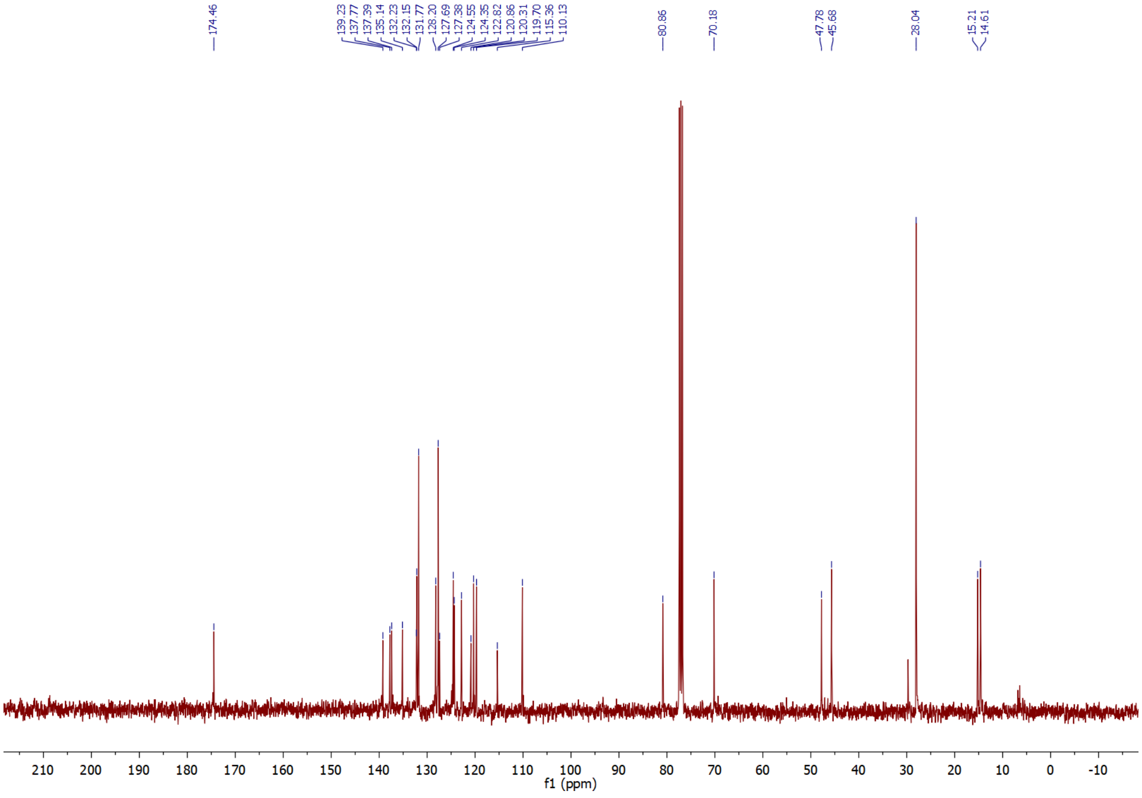

Figure S103. Racemic sample of 2b: IA column, n-Hex/i-PrOH 95:5, T= 30°C, F= 1.0 mL/min.

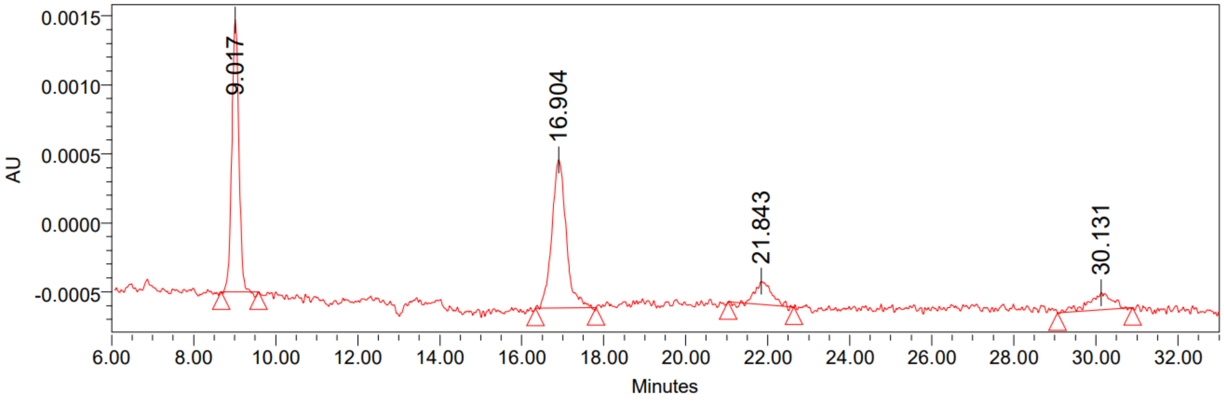

Processed Channel: PDA 320.6 nm

	Processed Channel	Retention Time (min)	Area	% Area	Height
1	PDA 320.6 nm	9.017	23996	39.72	1971
2	PDA 320.6 nm	16.904	26168	43.31	1075
3	PDA 320.6 nm	21.843	5234	8.66	171
4	PDA 320.6 nm	30.131	5017	8.30	124

Figure S104. Enantioenriched sample of (2*S*,3*R*,*S*$_a$)-2b: IA column, n-Hex/i-PrOH 95:5, T= 30°C, F= 1.0 mL/min.

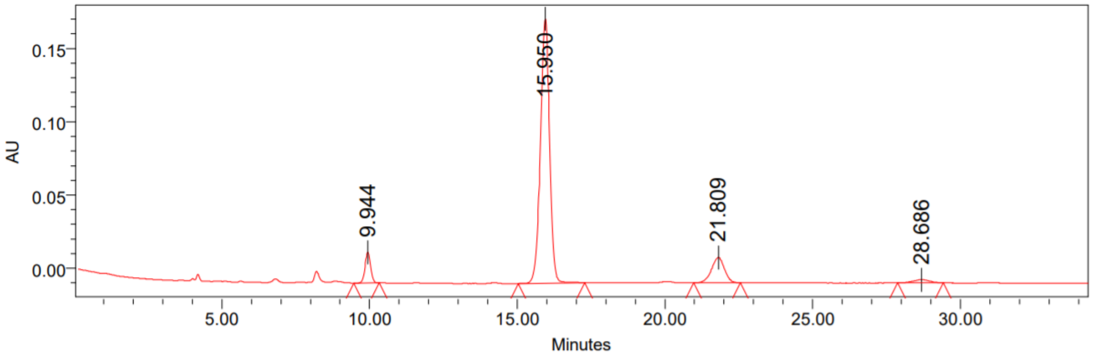

Processed Channel: PDA 265.2 nm

	Processed Channel	Retention Time (min)	Area	% Area	Height
1	PDA 265.2 nm	9.944	277767	5.81	21134
2	PDA 265.2 nm	15.950	3891601	81.45	180733
3	PDA 265.2 nm	21.809	525331	11.00	17302
4	PDA 265.2 nm	28.686	83012	1.74	2126

Figure S105. ^{1}H-NMR (400 MHz, $CDCl_3$) of **(2*S*,3*R*,*S*$_a$)-2c**:

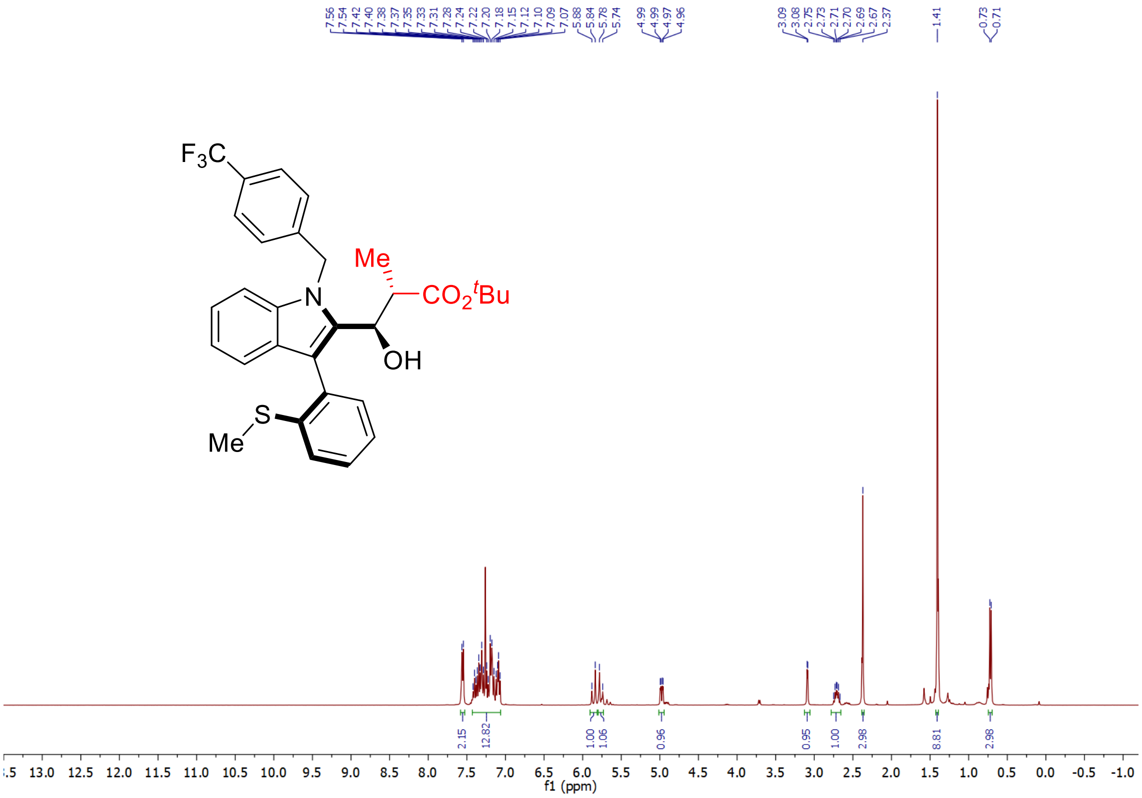

Figure S106. ^{13}C-NMR (100 MHz, $CDCl_3$) of **(2*S*,3*R*,*S*$_a$)-2c**:

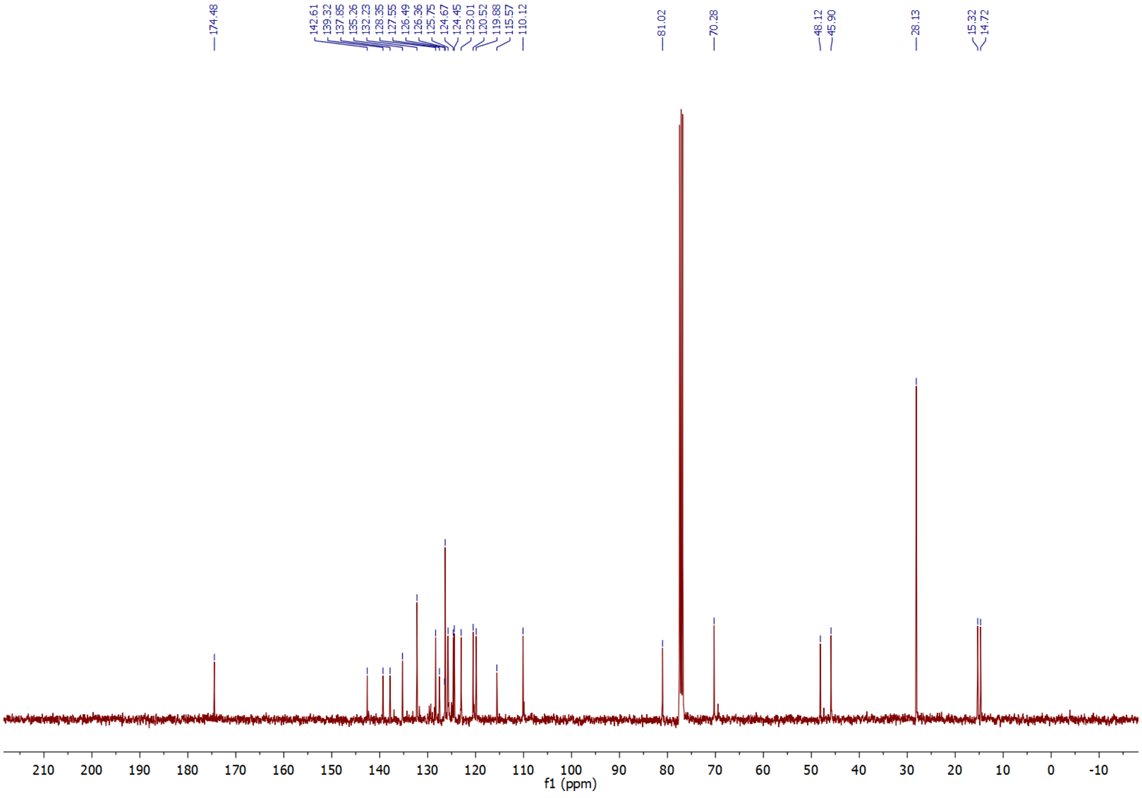

Figure S107. ^{19}F-NMR (376 MHz, $CDCl_3$) of **(2*S*,3*R*,*S*$_a$)-2c**:

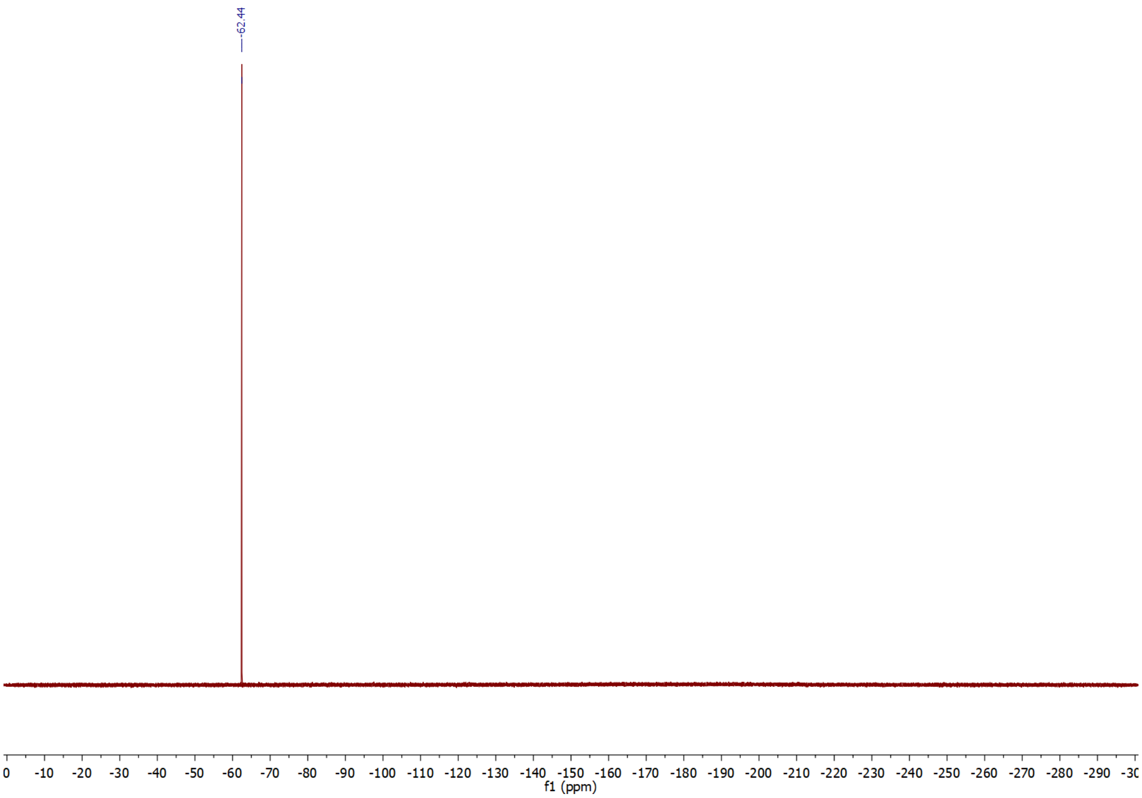

Figure S108. Racemic sample of 2c: IA column, n-Hex/i-PrOH 95:5, T= 30°C, F= 1.0 mL/min.

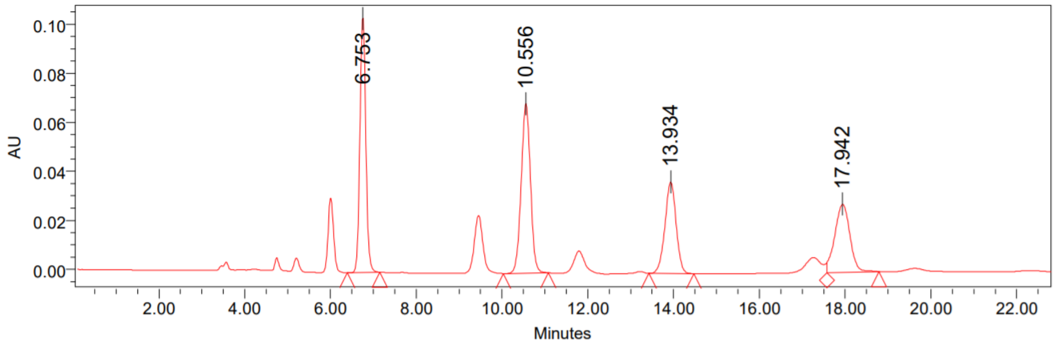

Processed Channel: PDA 258.2 nm

	Processed Channel	Retention Time (min)	Area	% Area	Height
1	PDA 258.2 nm	6.753	991439	29.36	104083
2	PDA 258.2 nm	10.556	1030340	30.52	69234
3	PDA 258.2 nm	13.934	679411	20.12	37443
4	PDA 258.2 nm	17.942	675217	20.00	27778

Figure S109. Enantioenriched sample of (2*S*,3*R*,*S*$_a$)-2c: IA column, n-Hex/i-PrOH 95:5, T= 30°C, F= 1.0 mL/min.

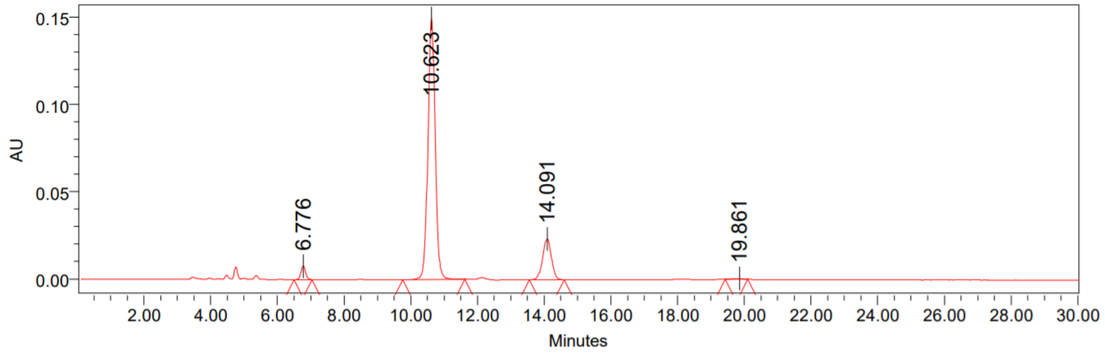

Processed Channel: PDA 282.7 nm

	Processed Channel	Retention Time (min)	Area	% Area	Height
1	PDA 282.7 nm	6.776	75784	2.70	7909
2	PDA 282.7 nm	10.623	2286446	81.49	149764
3	PDA 282.7 nm	14.091	433258	15.44	23489
4	PDA 282.7 nm	19.861	10183	0.36	440

Figure S110. ^{1}H-NMR (400 MHz, $CDCl_3$) of **(2*S*,3*R*,*S*$_a$)-2d**:

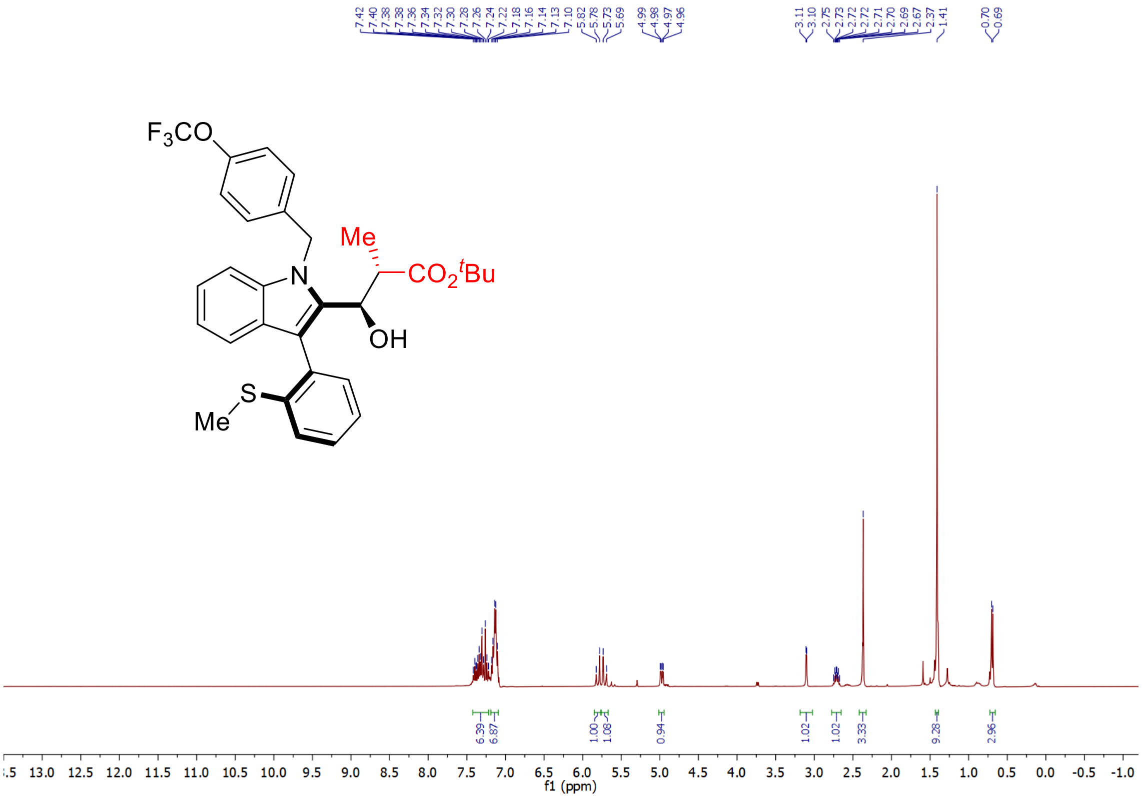

Figure S111. ^{13}C-NMR (100 MHz, $CDCl_3$) of **(2*S*,3*R*,*S*$_a$)-2d**:

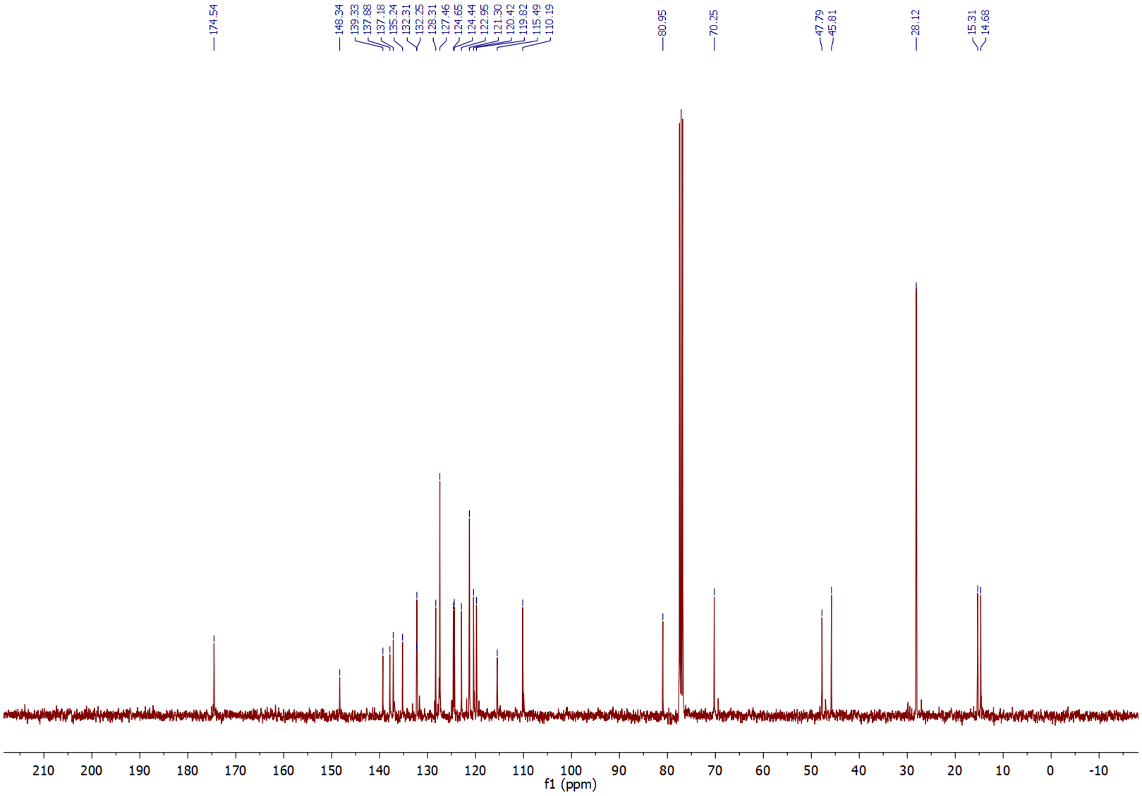

Figure S112. ^{19}F-NMR (376 MHz, $CDCl_3$) of **(2*S*,3*R*,*S**a*)-2d**:

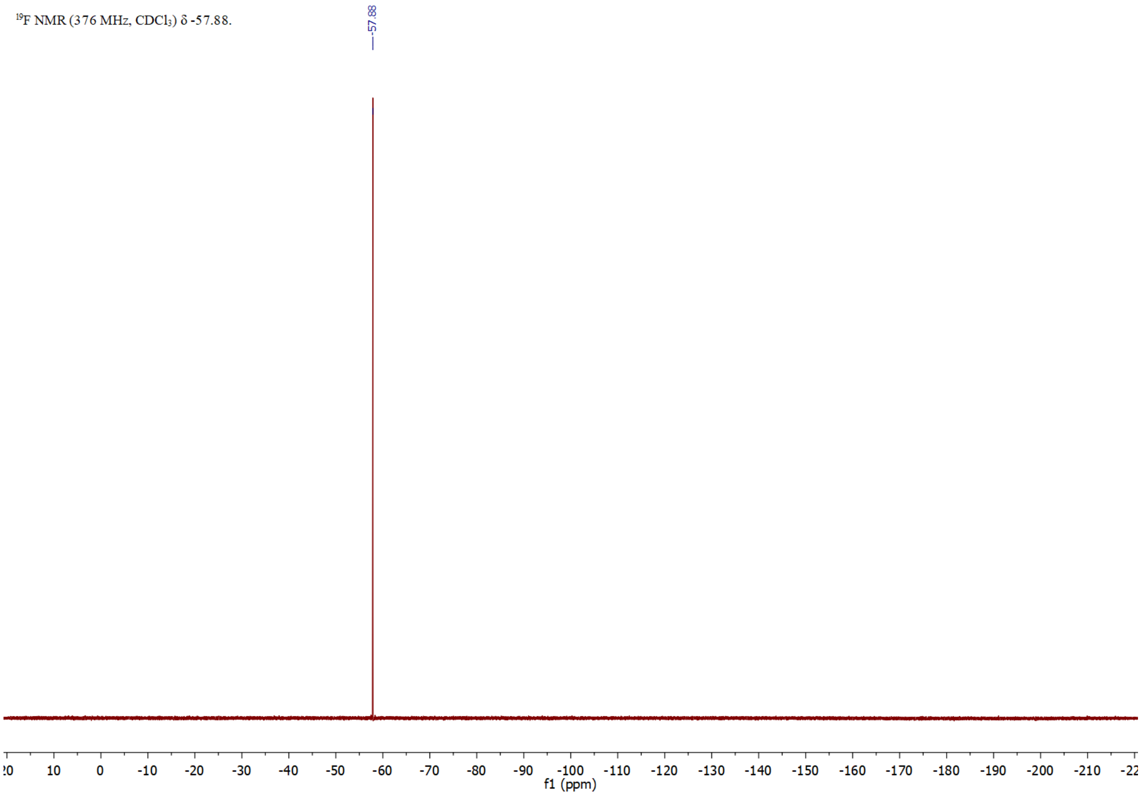

Figure S113. Racemic sample of 2d: IA column, n-Hex/i-PrOH 95:5, T= 30°C, F= 1.0 mL/min.

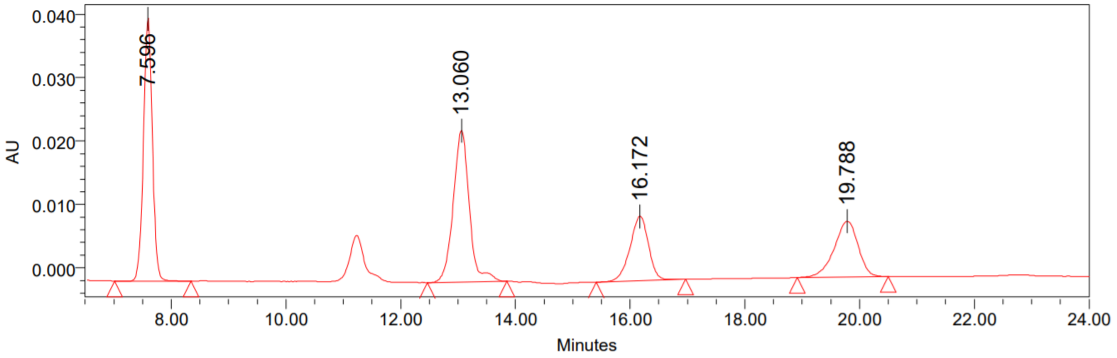

Processed Channel: PDA 255.2 nm

	Processed Channel	Retention Time (min)	Area	% Area	Height
1	PDA 255.2 nm	7.596	449299	32.12	41467
2	PDA 255.2 nm	13.060	466716	33.36	23926
3	PDA 255.2 nm	16.172	227459	16.26	10185
4	PDA 255.2 nm	19.788	255446	18.26	8800

Figure S114. Enantioenriched sample of (2*S*,3*R*,*S*$_a$)-2d: IA column, n-Hex/i-PrOH 95:5, T= 30°C, F= 1.0 mL/min.

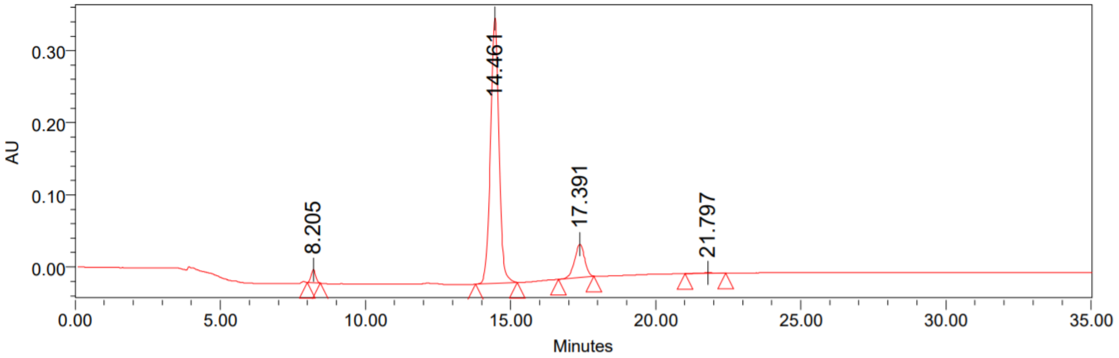

Processed Channel: PDA 229.7 nm

	Processed Channel	Retention Time (min)	Area	% Area	Height
1	PDA 229.7 nm	8.205	200486	2.27	18128
2	PDA 229.7 nm	14.461	7485829	84.88	368261
3	PDA 229.7 nm	17.391	1111275	12.60	45920
4	PDA 229.7 nm	21.797	21618	0.25	635

Figure S115. ^{1}H-NMR (400 MHz, $CDCl_3$) of **(2*S*,3*R*,*S*$_a$)-2e**:

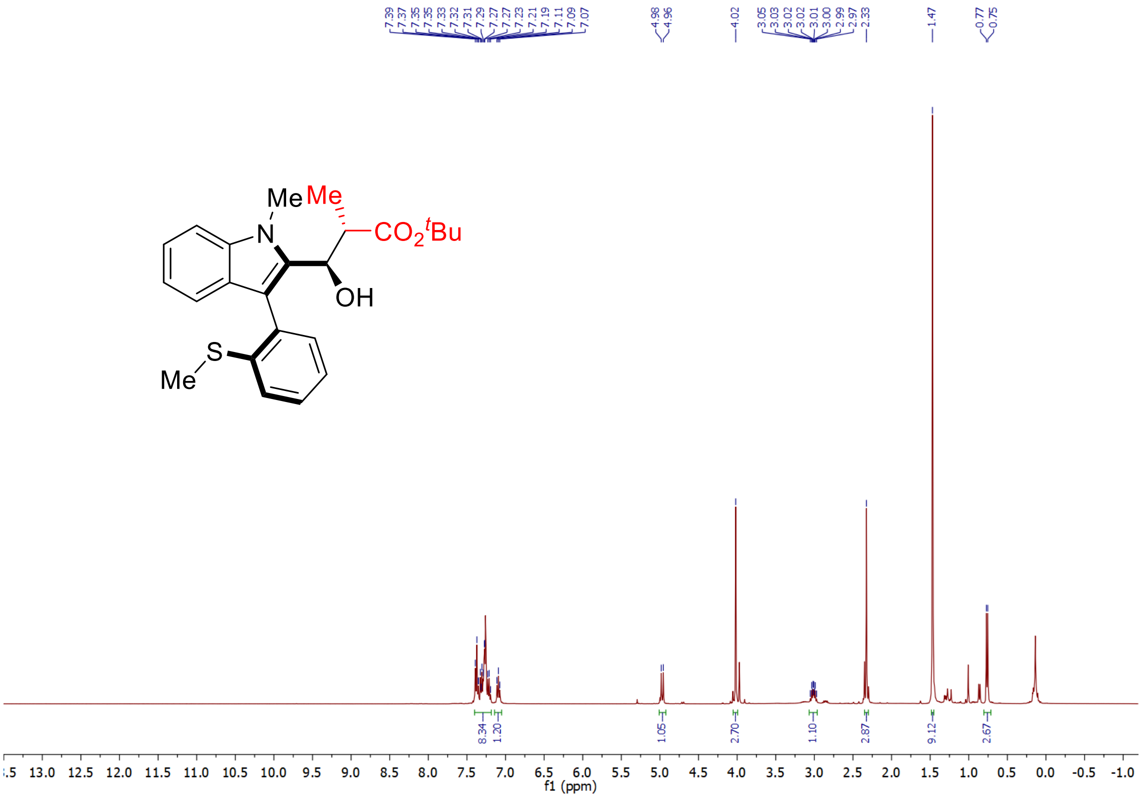

Figure S116. ^{13}C-NMR (100 MHz, $CDCl_3$) of **(2*S*,3*R*,*S*$_a$)-2e**:

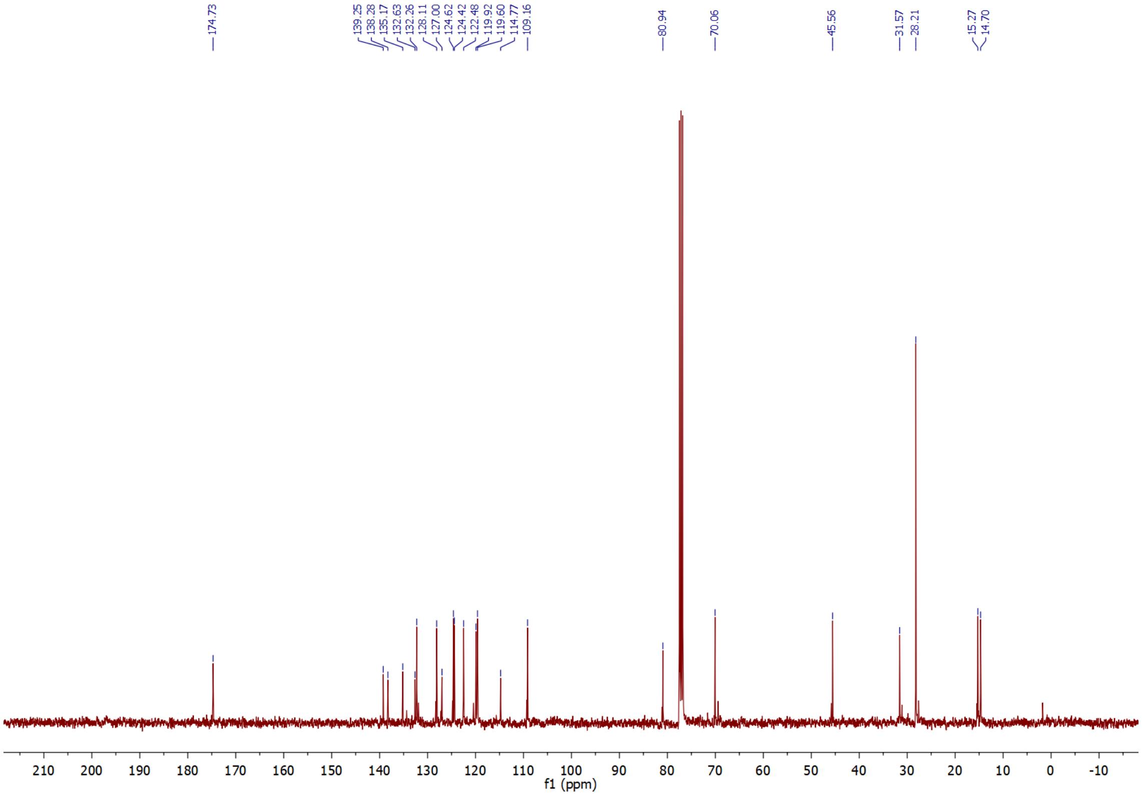

Figure S117. Racemic sample of 2e: IC column, n-Hex/i-PrOH 99:1, T= 30°C, F= 1.0 mL/min.

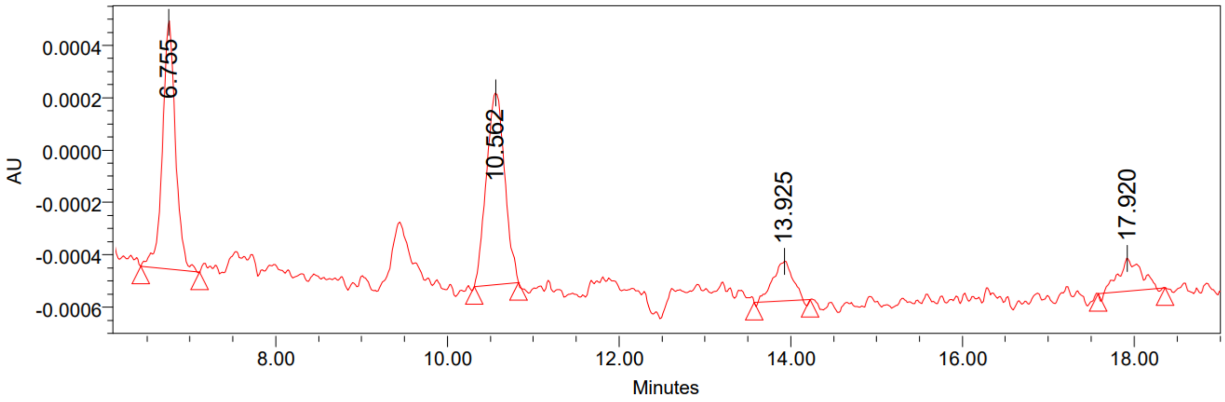

Processed Channel: PDA 325.4 nm

	Processed Channel	Retention Time (min)	Area	% Area	Height
1	PDA 325.4 nm	6.755	10008	38.63	947
2	PDA 325.4 nm	10.562	10910	42.12	734
3	PDA 325.4 nm	13.925	2535	9.78	151
4	PDA 325.4 nm	17.920	2452	9.47	125

Figure S118. Enantioenriched sample of (2*S*,3*R*,*S*$_a$)-2e: IC column, n-Hex/i-PrOH 99:1, T= 30°C, F= 1.0 mL/min.

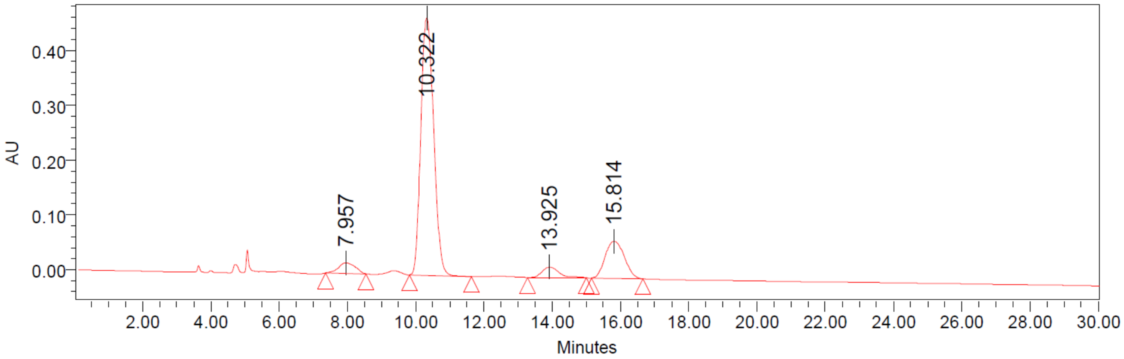

Processed Channel: PDA 246.6 nm

	Processed Channel	Retention Time (min)	Area	% Area	Height
1	PDA 246.6 nm	7.957	662404	4.09	19277
2	PDA 246.6 nm	10.322	12243728	75.53	469719
3	PDA 246.6 nm	13.925	667312	4.12	19177
4	PDA 246.6 nm	15.814	2637928	16.27	67424

Figure S119. ^{1}H-NMR (400 MHz, $CDCl_3$) of **(2*S*,3*R*,*S*$_a$)-2f**:

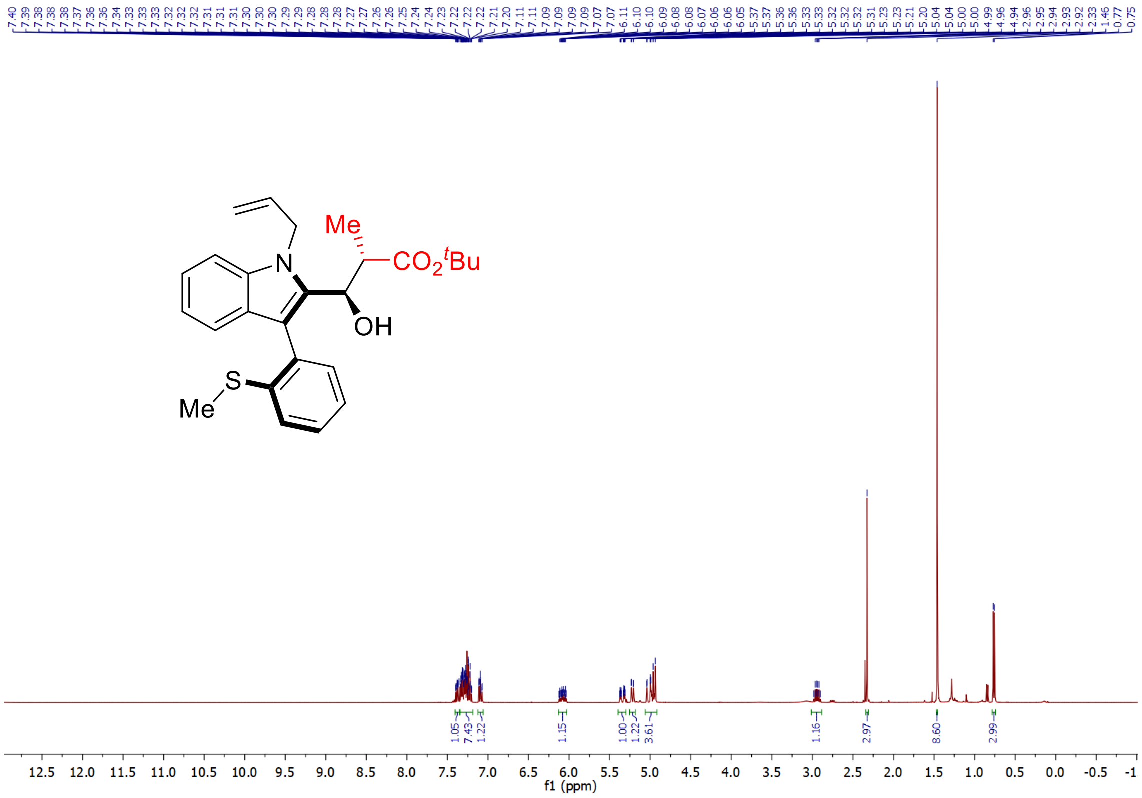

Figure S120. ^{13}C-NMR (100 MHz, $CDCl_3$) of **(2*S*,3*R*,*S*$_a$)-2f**:

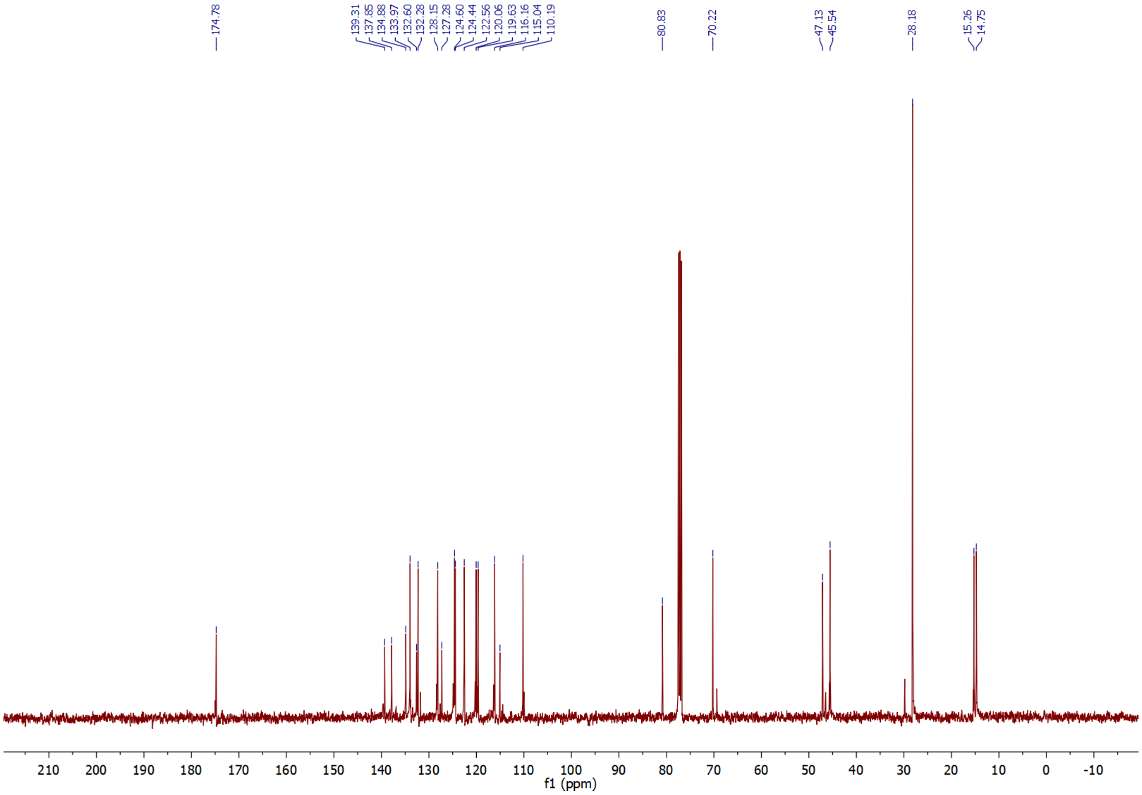

Figure S121. Racemic sample of 2f: IA column, n-Hex/i-PrOH 95:5, T= 30°C, F= 1.0 mL/min.

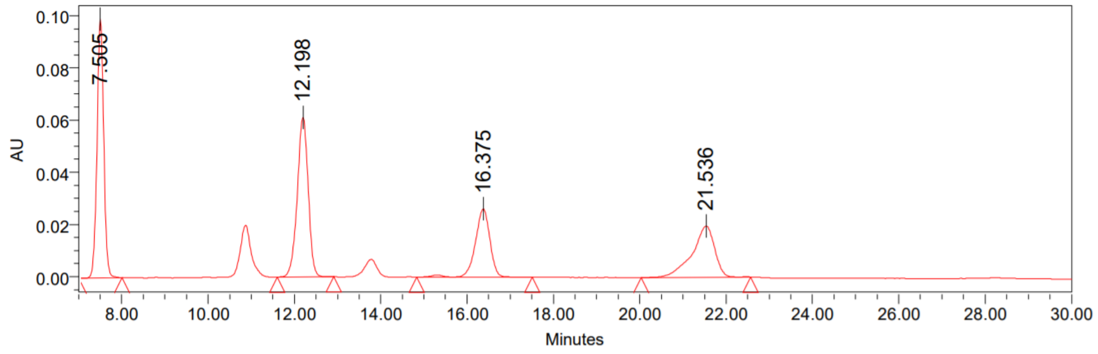

Processed Channel: PDA 259.5 nm

	Processed Channel	Retention Time (min)	Area	% Area	Height
1	PDA 259.5 nm	7.505	1072802	30.83	99213
2	PDA 259.5 nm	12.198	1077349	30.96	61270
3	PDA 259.5 nm	16.375	617351	17.74	26207
4	PDA 259.5 nm	21.536	712177	20.47	19591

Figure S122. Enantioenriched sample of (2*S*,3*R*,*S*$_a$)-2f: IA column, n-Hex/i-PrOH 95:5, T= 30°C, F= 1.0 mL/min.

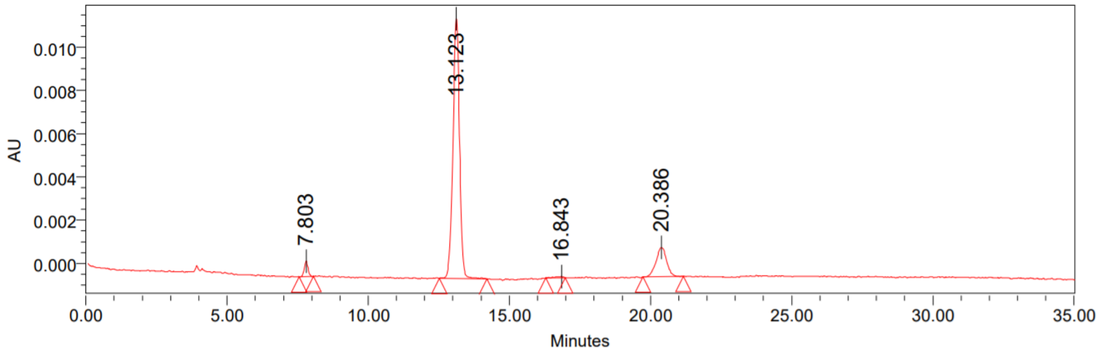

Processed Channel: PDA 288.0 nm

	Processed Channel	Retention Time (min)	Area	% Area	Height
1	PDA 288.0 nm	7.803	6510	2.71	711
2	PDA 288.0 nm	13.123	196834	82.04	12038
3	PDA 288.0 nm	16.843	1189	0.50	55
4	PDA 288.0 nm	20.386	35405	14.76	1357

Figure S123. ^{1}H-NMR (400 MHz, $CDCl_3$) of **(2*S*,3*R*,*S*$_a$)-2g**:

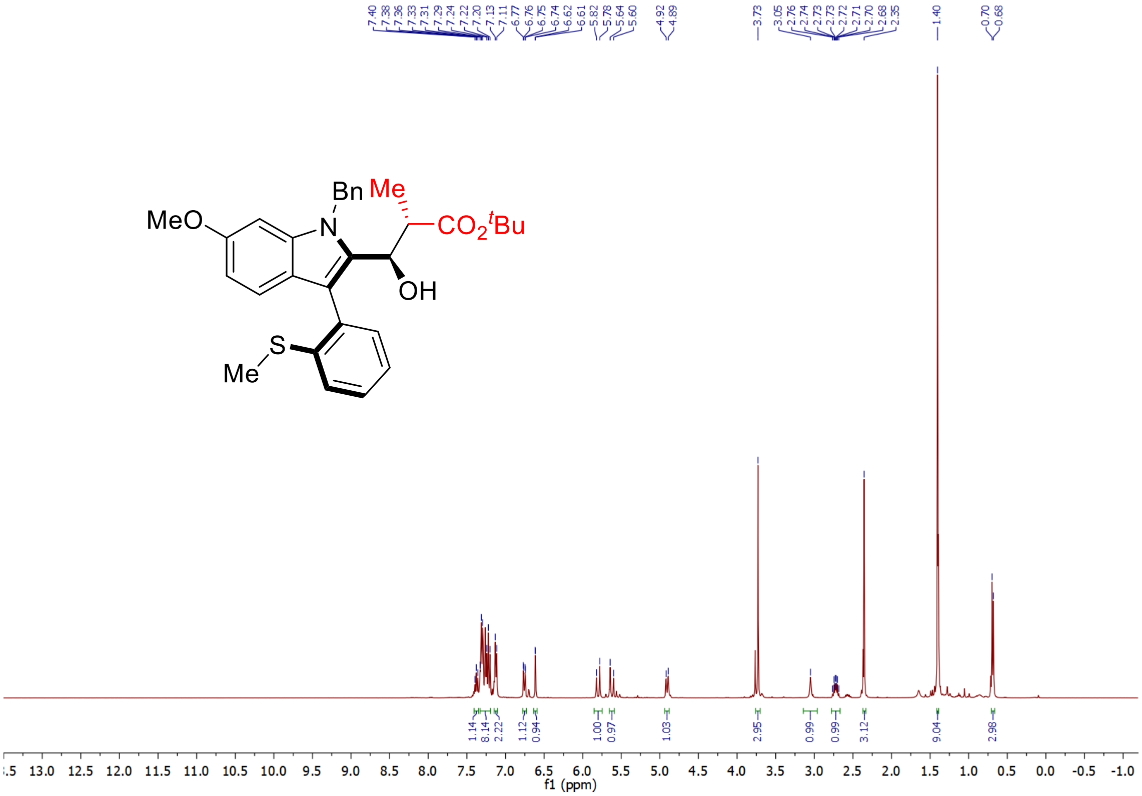

Figure S124. ^{13}C-NMR (100 MHz, $CDCl_3$) of **(2*S*,3*R*,*S*$_a$)-2g**:

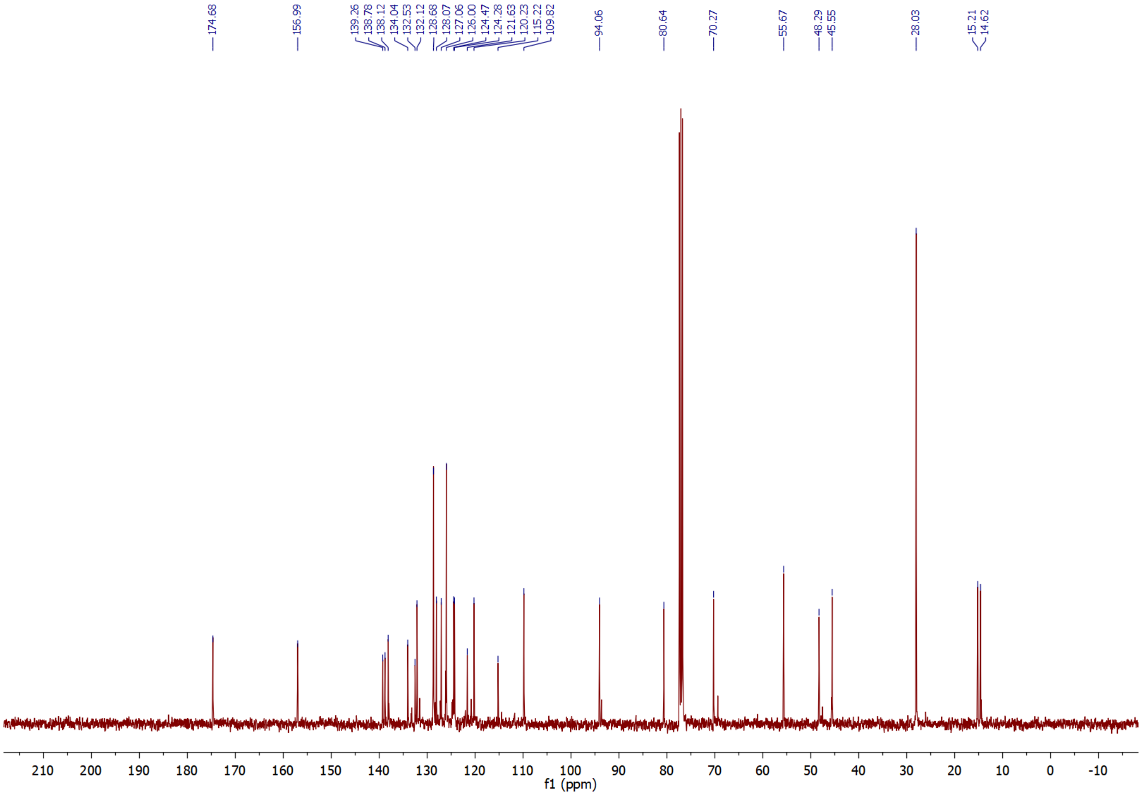

Figure S125. Racemic sample of 2g: IA column, n-Hex/i-PrOH 95:5, T= 30°C, F= 1.0 mL/min.

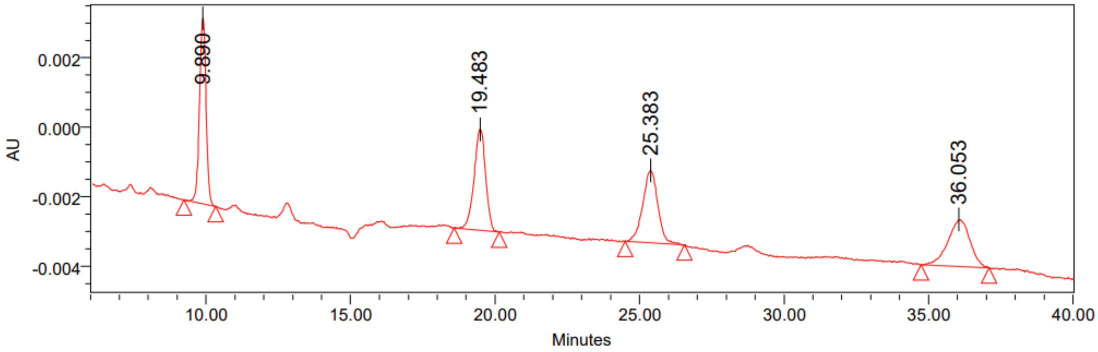

Processed Channel: PDA 267.0 nm

	Processed Channel	Retention Time (min)	Area	% Area	Height
1	PDA 267.0 nm	9.890	83373	26.81	5341
2	PDA 267.0 nm	19.483	80528	25.90	2909
3	PDA 267.0 nm	25.383	75814	24.38	2082
4	PDA 267.0 nm	36.053	71226	22.91	1342

Figure S126. Enantioenriched sample (2*S*,3*R*,*S*$_a$)-2g: IA column, n-Hex/i-PrOH 95:5, T= 30°C, F= 1.0 mL/min.

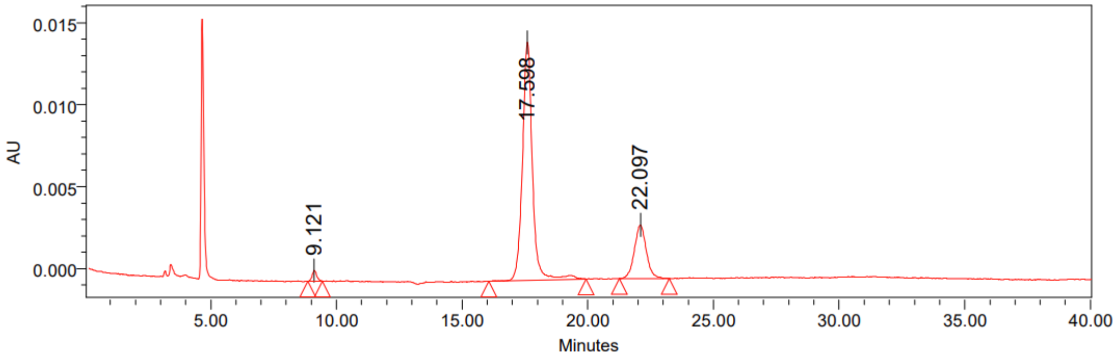

Processed Channel: PDA 282.4 nm

	Processed Channel	Retention Time (min)	Area	% Area	Height
1	PDA 282.4 nm	9.121	8315	1.61	651
2	PDA 282.4 nm	17.598	402347	77.68	14533
3	PDA 282.4 nm	22.097	107323	20.72	3262

Figure S127. ^{1}H-NMR (400 MHz, $CDCl_3$) of **(2*S*,3*R*,*S*$_a$)-2h**:

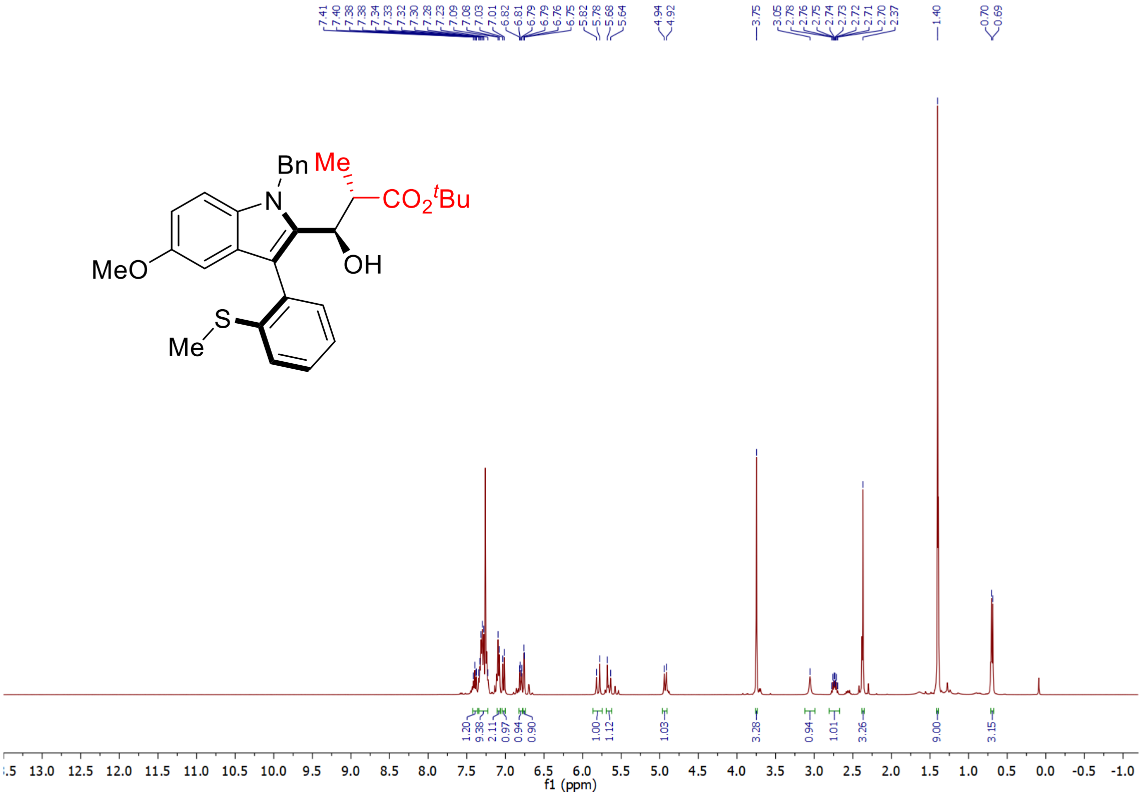

Figure S128. ^{13}C-NMR (100 MHz, $CDCl_3$) of **(2*S*,3*R*,*S*$_a$)-2h**:

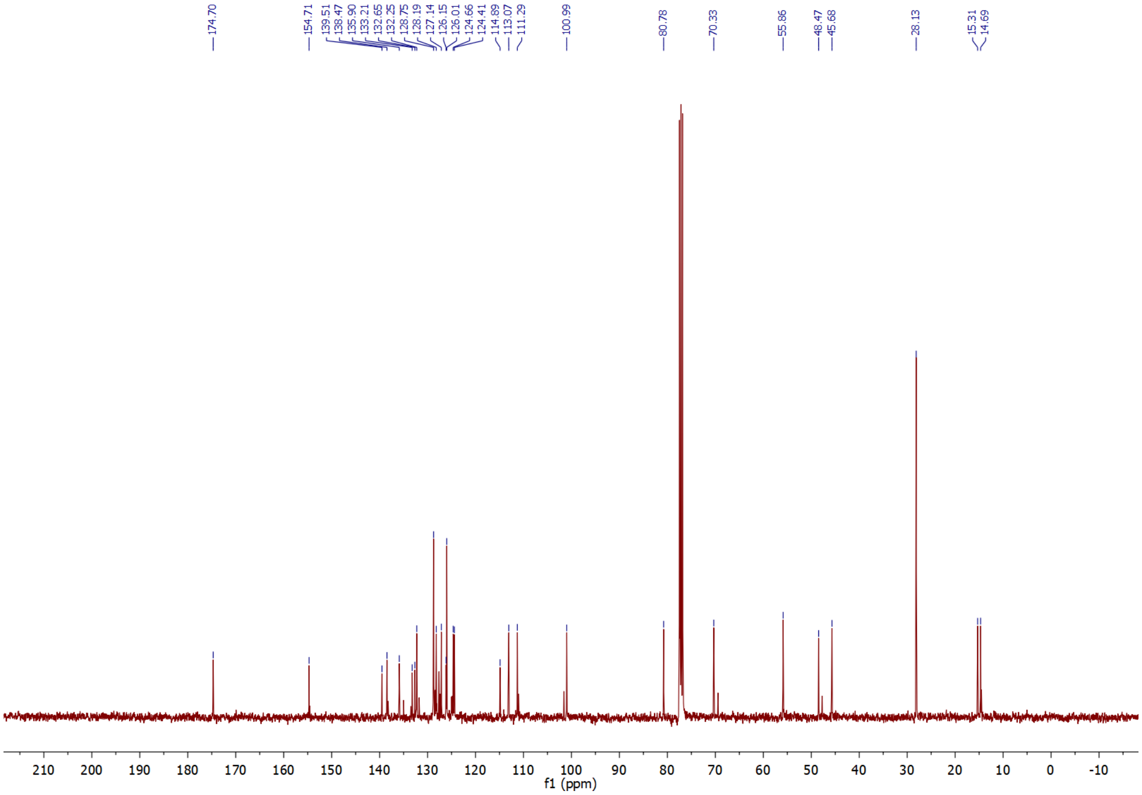

Figure S129. Racemic sample of 2h: IA column, n-Hex/i-PrOH 95:5, T= 30°C, F= 1.0 mL/min.

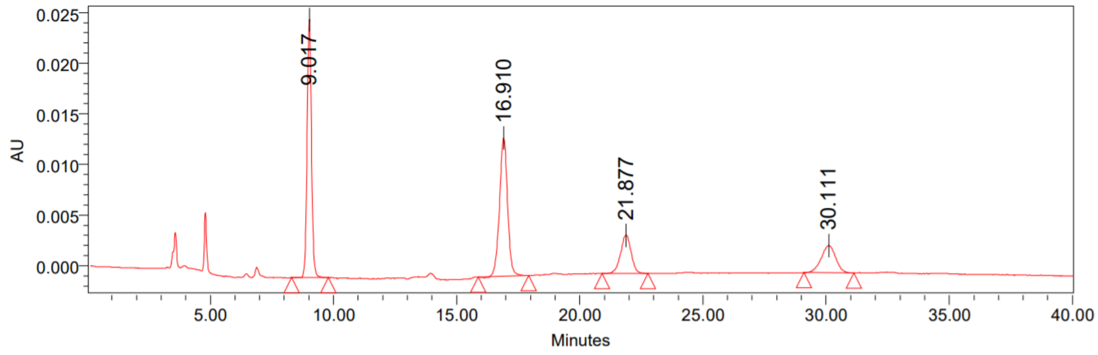

Processed Channel: PDA 262.1 nm

	Processed Channel	Retention Time (min)	Area	% Area	Height
1	PDA 262.1 nm	9.017	311650	36.75	25507
2	PDA 262.1 nm	16.910	313086	36.92	13661
3	PDA 262.1 nm	21.877	113802	13.42	3793
4	PDA 262.1 nm	30.111	109470	12.91	2681

Figure S130. Enantioenriched sample of (2*S*,3*R*,*S*$_a$)-2h: IA column, n-Hex/i-PrOH 95:5, T= 30°C, F= 1.0 mL/min.

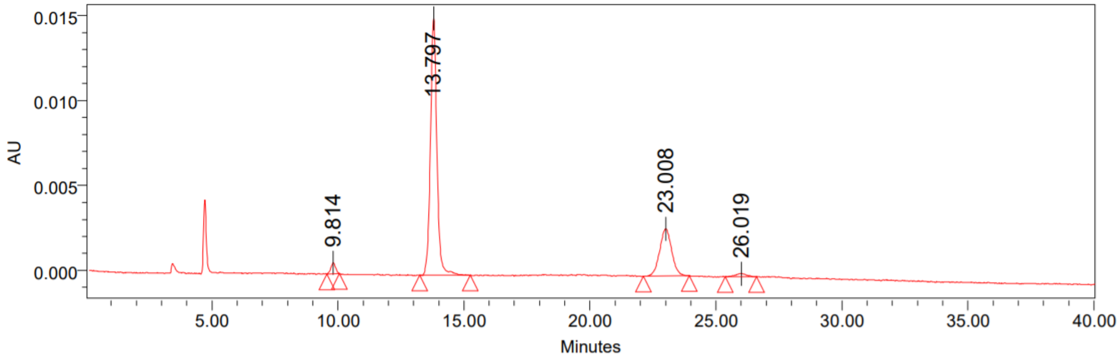

Processed Channel: PDA 309.3 nm

	Processed Channel	Retention Time (min)	Area	% Area	Height
1	PDA 309.3 nm	9.814	8374	2.23	654
2	PDA 309.3 nm	13.797	269905	71.75	15143
3	PDA 309.3 nm	23.008	91951	24.45	2780
4	PDA 309.3 nm	26.019	5918	1.57	191

Figure S131. ^{1}H-NMR (400 MHz, $CDCl_3$) of **(2*S*,3*R*,*S*$_a$)-2i**:

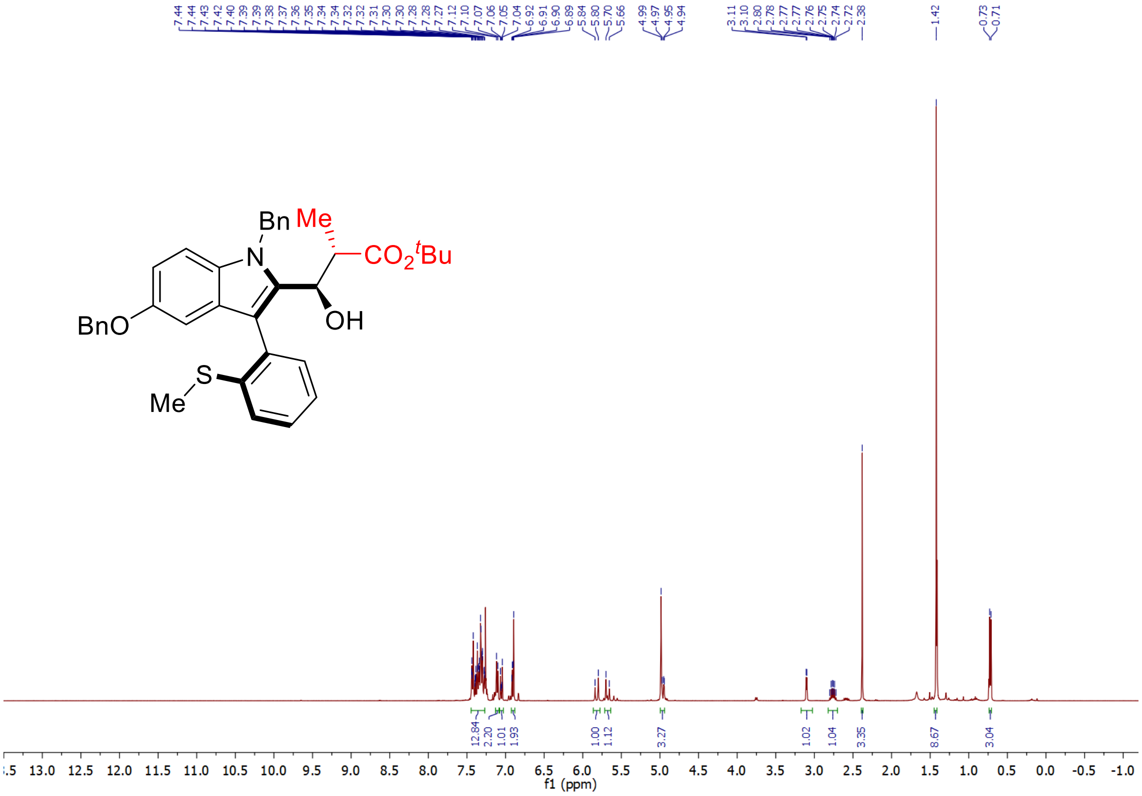

Figure S132. ^{13}C-NMR (100 MHz, $CDCl_3$) of **(2*S*,3*R*,*S*$_a$)-2i**:

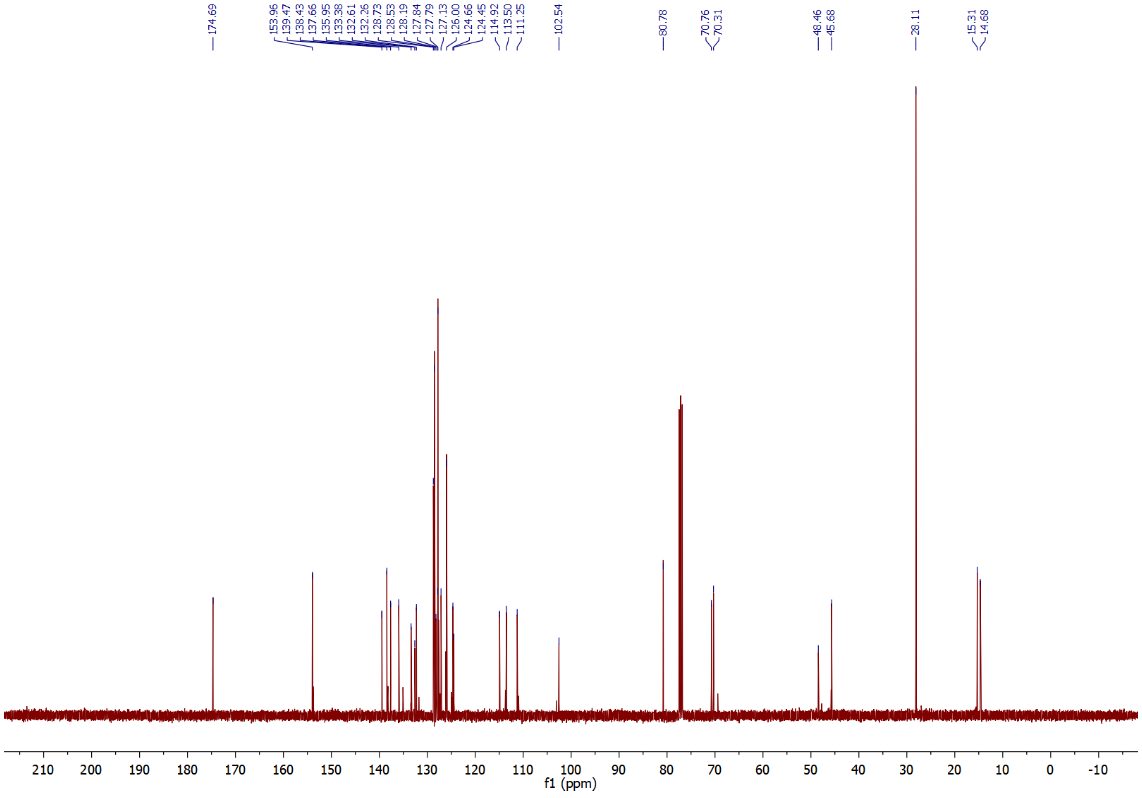

Figure S133. Racemic sample of 2i: IA column, n-Hex/i-PrOH 95:5, T= 30°C, F= 1.0 mL/min.

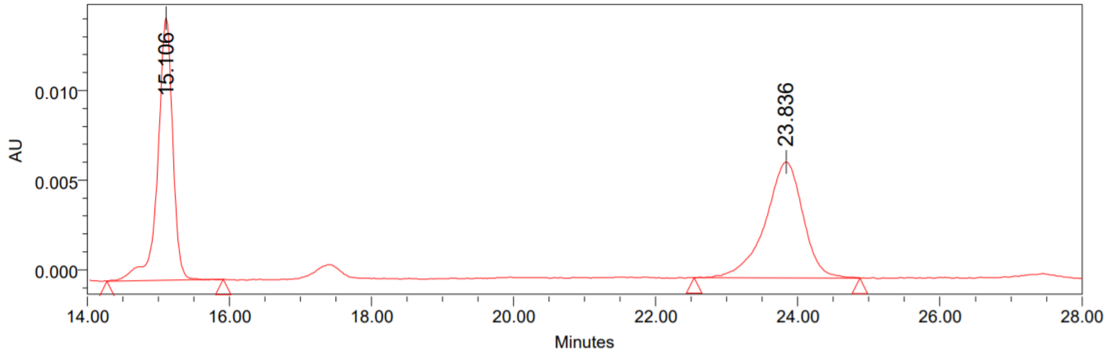

Processed Channel: PDA 290.2 nm

	Processed Channel	Retention Time (min)	Area	% Area	Height
1	PDA 290.2 nm	15.106	214442	47.39	14640
2	PDA 290.2 nm	23.836	238088	52.61	6458

Figure S134. Enantioenriched sample of (2*S*,3*R*,*S*$_a$)-2i: IA column, n-Hex/i-PrOH 95:5, T= 30°C, F= 1.0 mL/min.

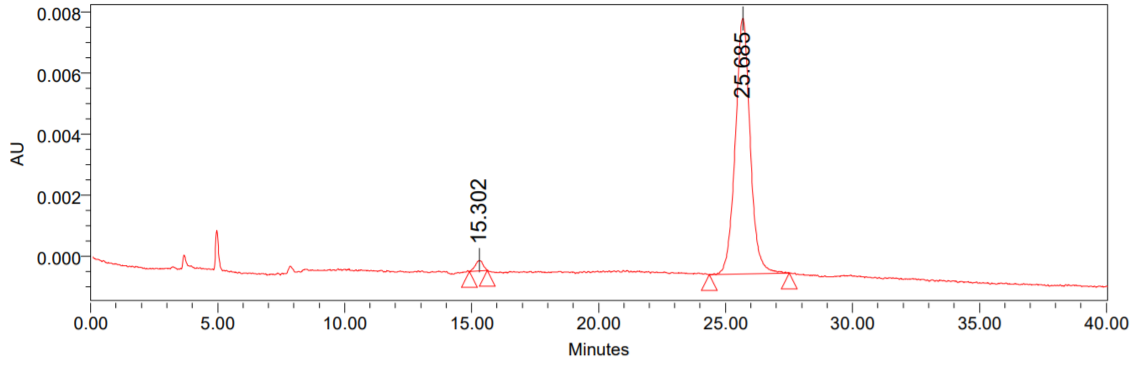

Processed Channel: PDA 319.2 nm

	Processed Channel	Retention Time (min)	Area	% Area	Height
1	PDA 319.2 nm	15.302	7205	2.07	345
2	PDA 319.2 nm	25.685	340888	97.93	8366

Figure S135. ^{1}H-NMR (400 MHz, $CDCl_3$) of **(2*S*,3*R*,*S*$_a$)-2j**:

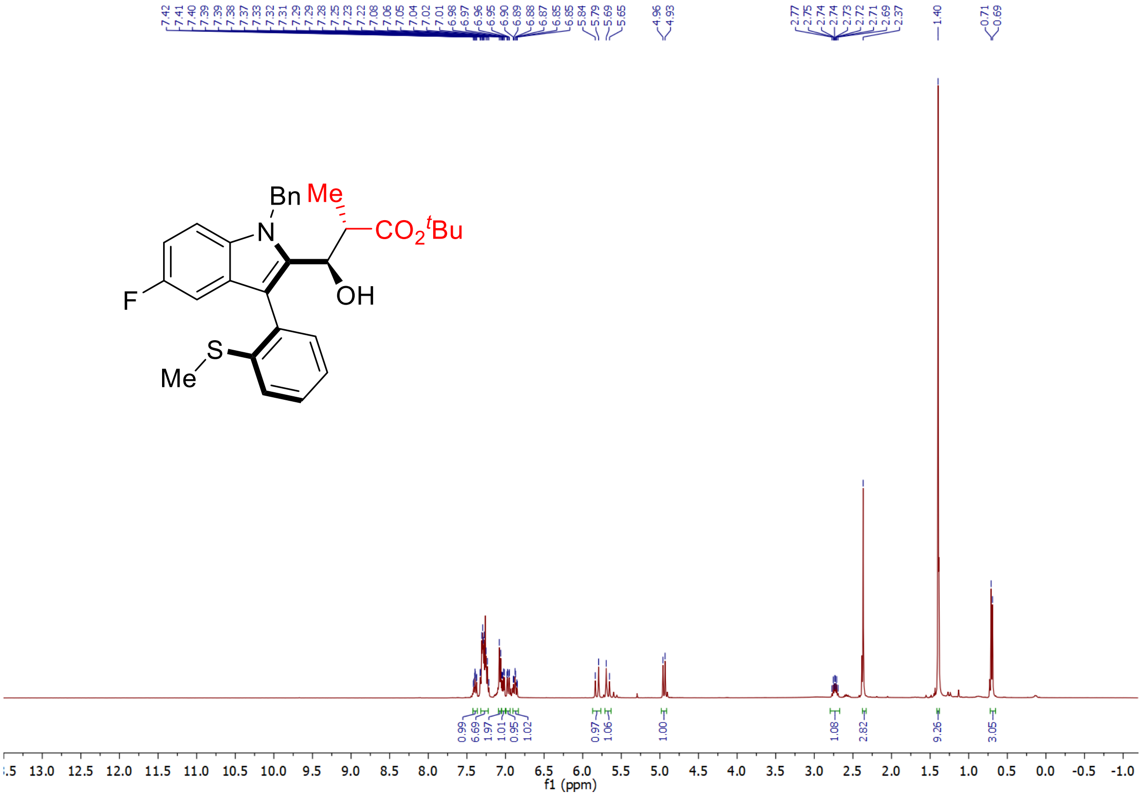

Figure S136. ^{13}C-NMR (100 MHz, $CDCl_3$) of **(2*S*,3*R*,*S*$_a$)-2j**:

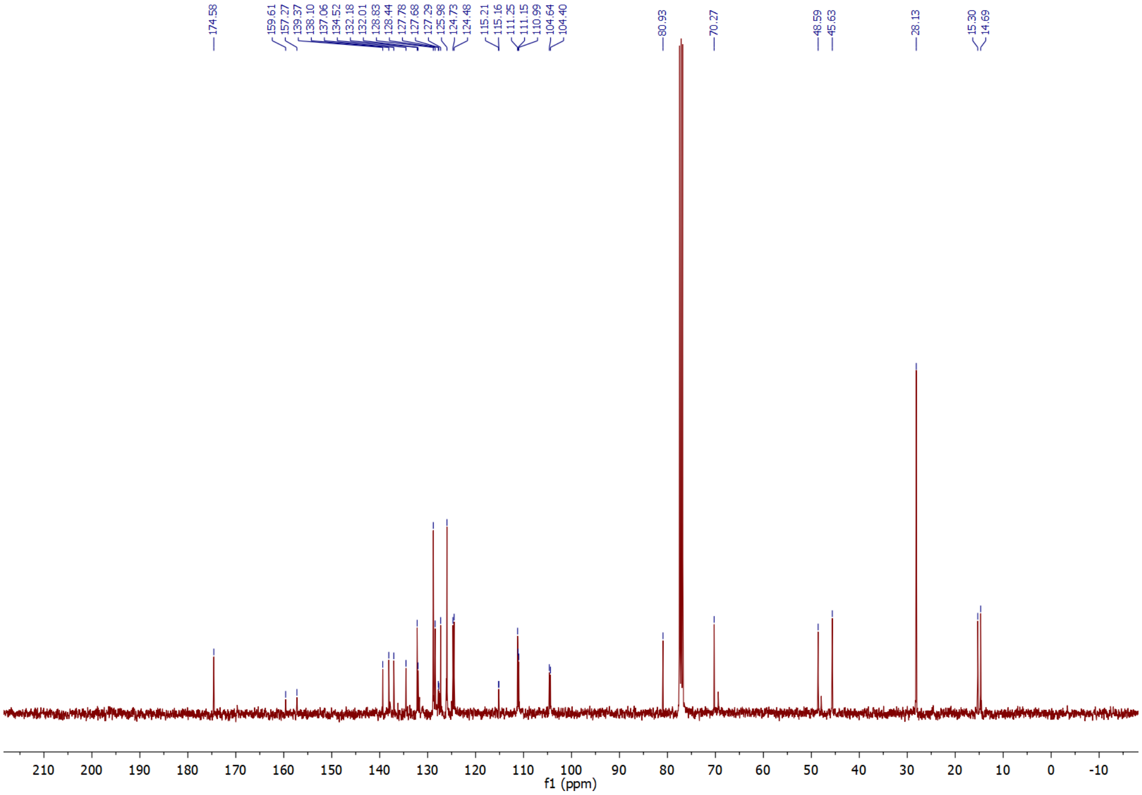

Figure S137. ^{19}F-NMR (376 MHz, $CDCl_3$) of **(2*S*,3*R*,*S*$_a$)-2j**:

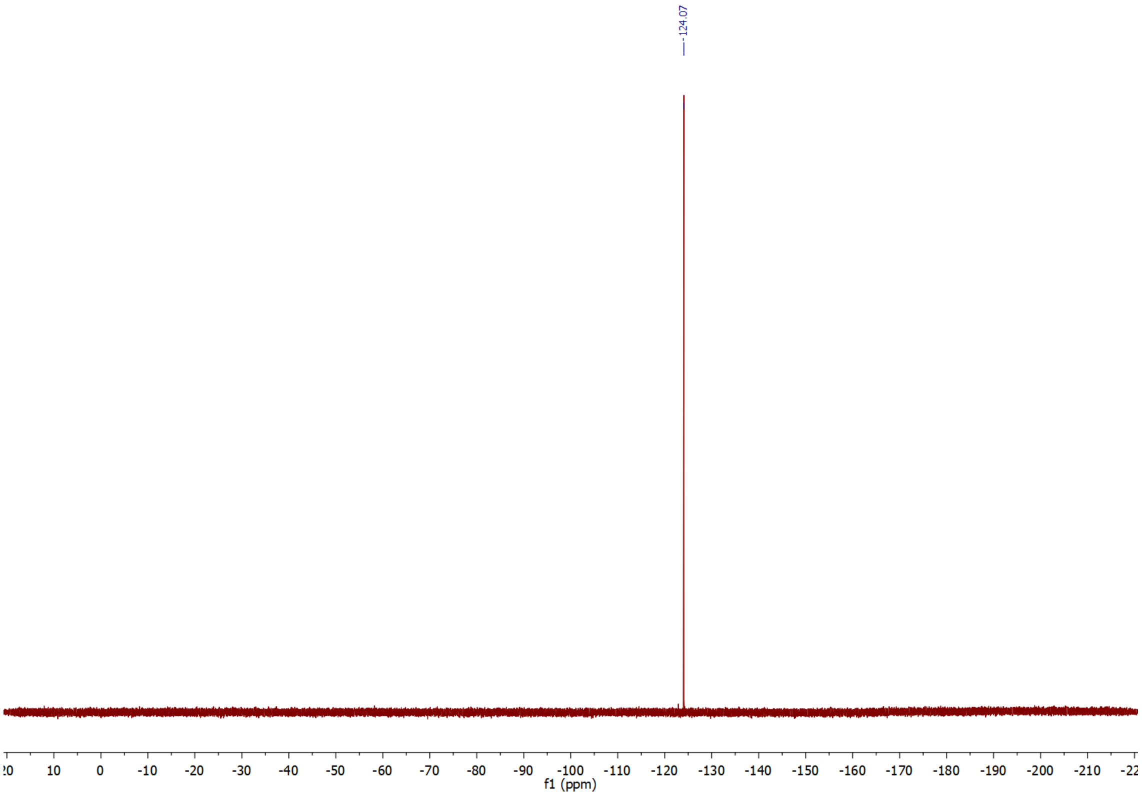

Figure S138. Racemic sample of 2j: IA column, n-Hex/i-PrOH 95:5, T= 30°C, F= 1.0 mL/min.

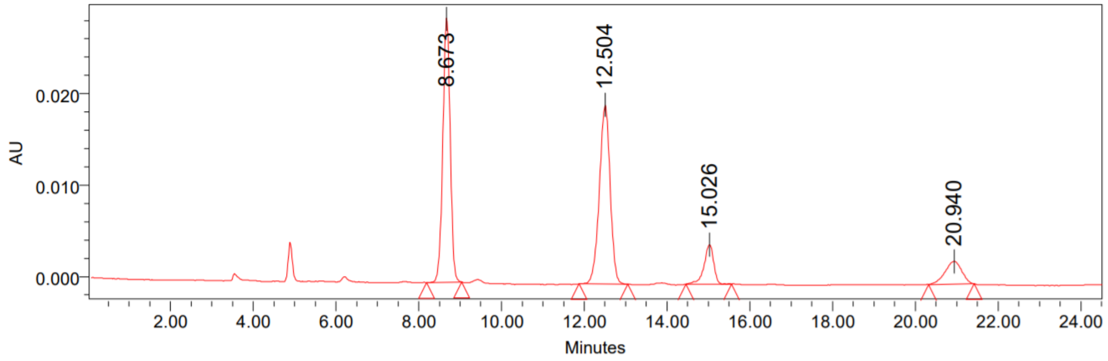

Processed Channel: PDA 297.5 nm

	Processed Channel	Retention Time (min)	Area	% Area	Height
1	PDA 297.5 nm	8.673	356296	41.88	28885
2	PDA 297.5 nm	12.504	355121	41.74	19575
3	PDA 297.5 nm	15.026	69384	8.15	4285
4	PDA 297.5 nm	20.940	70031	8.23	2492

Figure S139. Enantioenriched sample of (2*S*,3*R*,*S*$_a$)-2j: IA column, n-Hex/i-PrOH 95:5, T= 30°C, F= 1.0 mL/min.

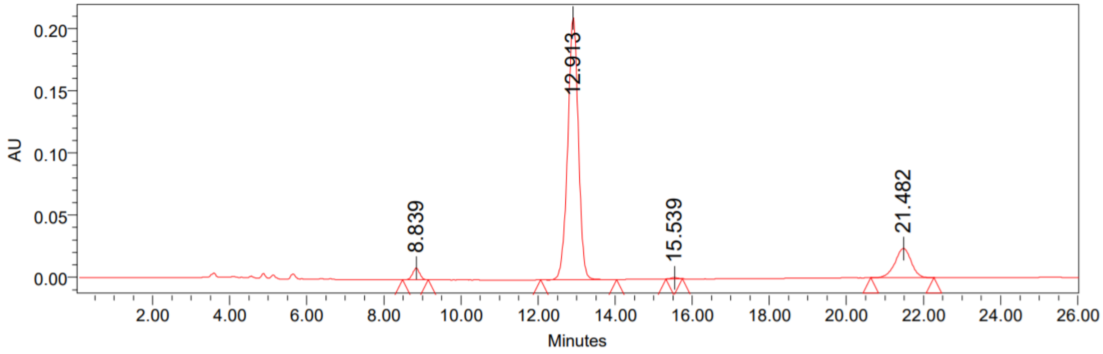

Processed Channel: PDA 253.3 nm

	Processed Channel	Retention Time (min)	Area	% Area	Height
1	PDA 253.3 nm	8.839	120108	2.48	9477
2	PDA 253.3 nm	12.913	4030855	83.10	211099
3	PDA 253.3 nm	15.539	11937	0.25	947
4	PDA 253.3 nm	21.482	687862	14.18	23556

Figure S140. ^{1}H-NMR (400 MHz, $CDCl_3$) of **(2*S*,3*R*,*S*$_a$)-2k**:

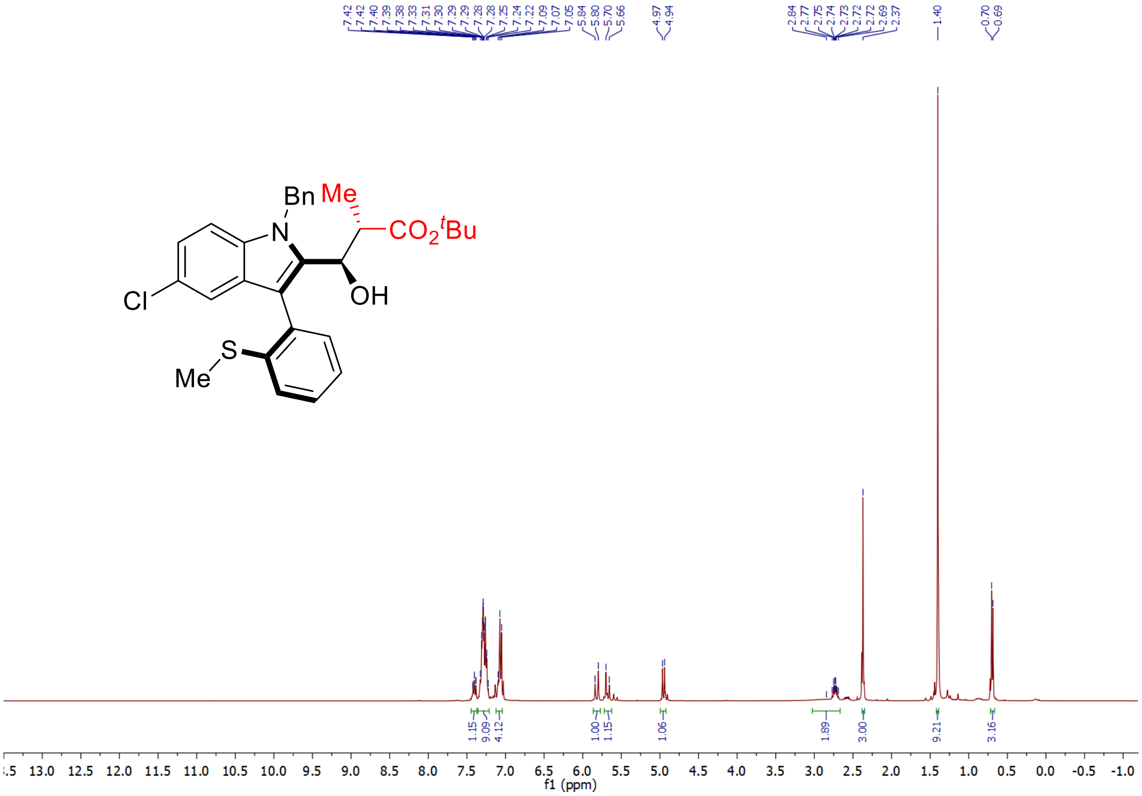

Figure S141. ^{13}C-NMR (100 MHz, $CDCl_3$) of **(2*S*,3*R*,*S*$_a$)-2k**:

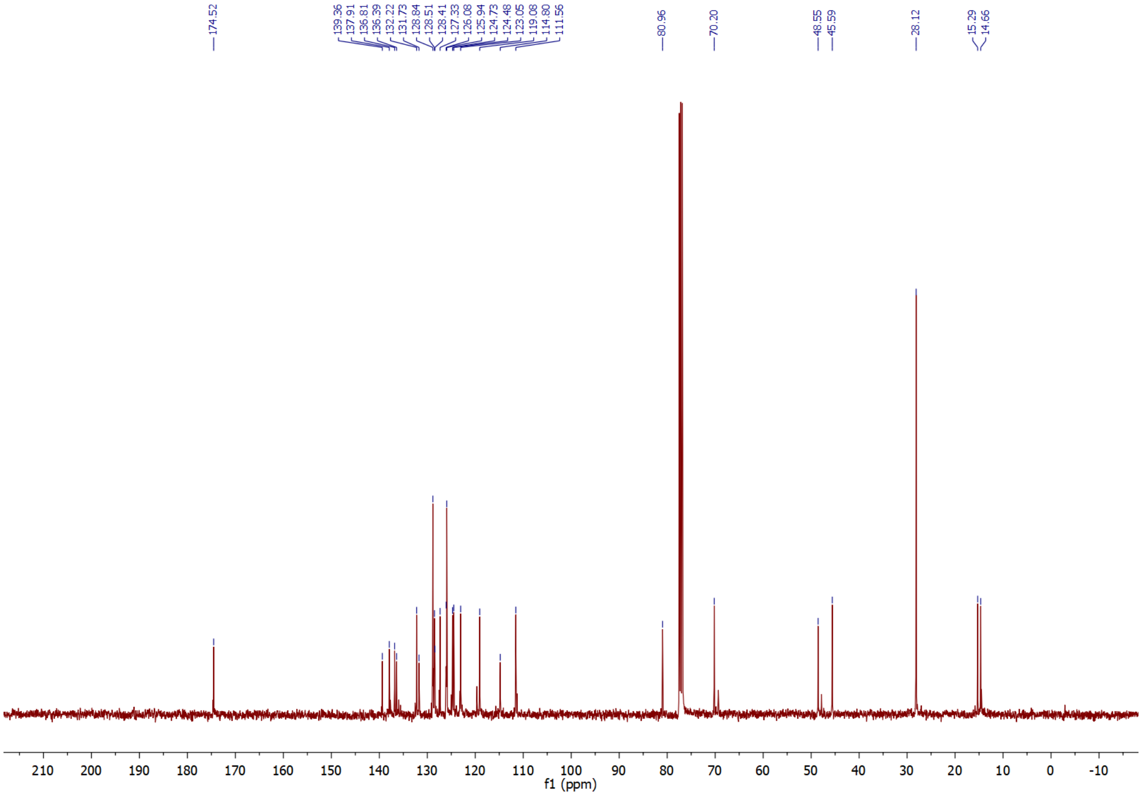

Figure S142. Racemic sample of 2k: ID column, n-Hex/i-PrOH 98:2, T= 30°C, F= 1.0 mL/min.

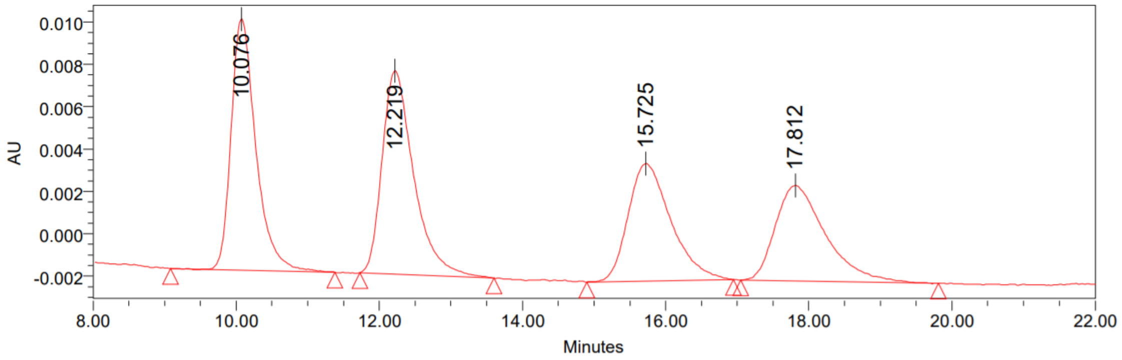

Processed Channel: PDA 272.7 nm

	Processed Channel	Retention Time (min)	Area	% Area	Height
1	PDA 272.7 nm	10.076	284582	27.70	11867
2	PDA 272.7 nm	12.219	294716	28.69	9609
3	PDA 272.7 nm	15.725	229385	22.33	5549
4	PDA 272.7 nm	17.812	218559	21.28	4511

Figure S143. Enantioenriched sample of (2*S*,3*R*,S_a)-2k: ID column, n-Hex/i-PrOH 98:2, T= 30°C, F= 1.0 mL/min.

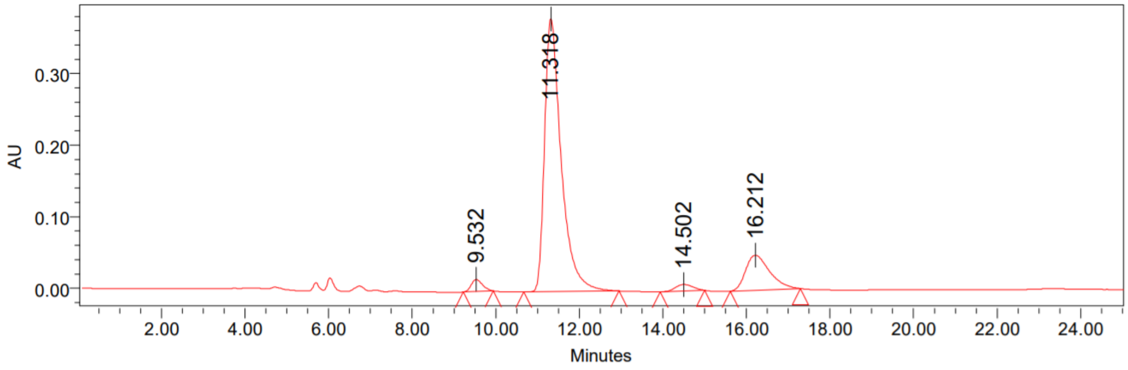

Processed Channel: PDA 244.1 nm

	Processed Channel	Retention Time (min)	Area	% Area	Height
1	PDA 244.1 nm	9.532	319626	2.44	16920
2	PDA 244.1 nm	11.318	10578178	80.63	381407
3	PDA 244.1 nm	14.502	264243	2.01	9224
4	PDA 244.1 nm	16.212	1957190	14.92	48945

Figure S144. ^{1}H-NMR (400 MHz, $CDCl_3$) of **(2*S*,3*R*,*S*$_a$)-2l**:

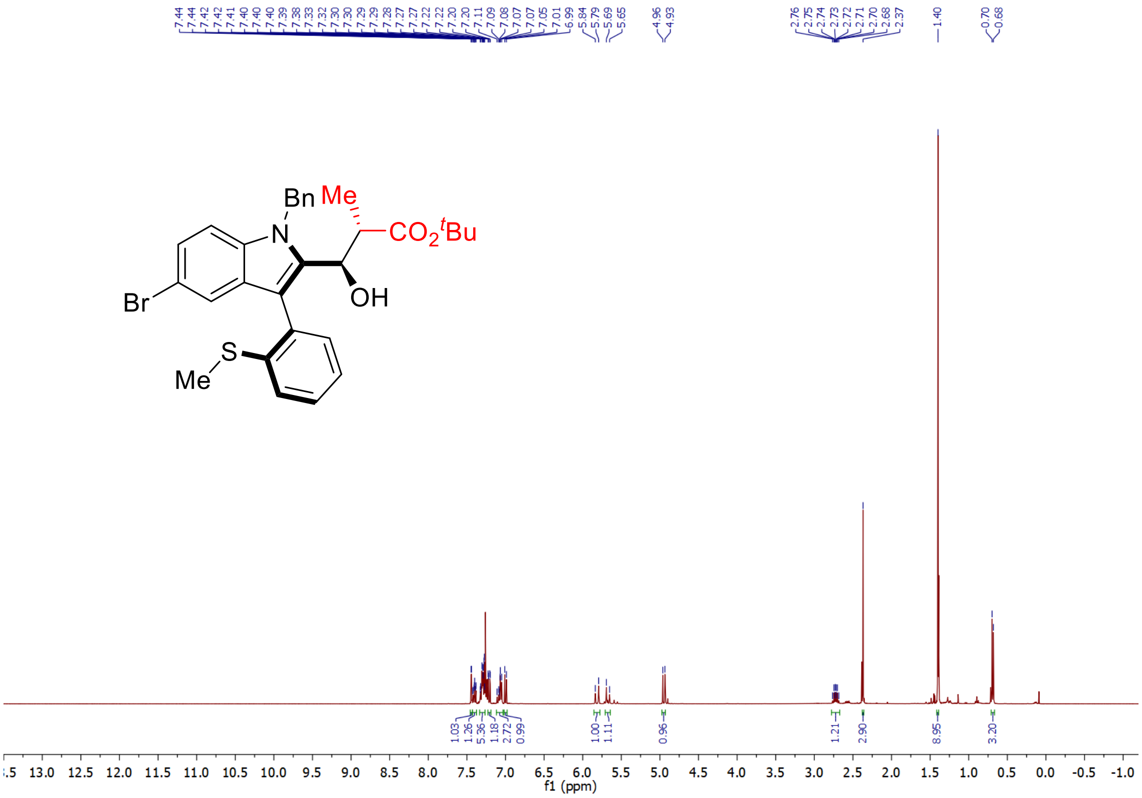

Figure S145. ^{13}C-NMR (100 MHz, $CDCl_3$) of **(2*S*,3*R*,*S*$_a$)-2l**:

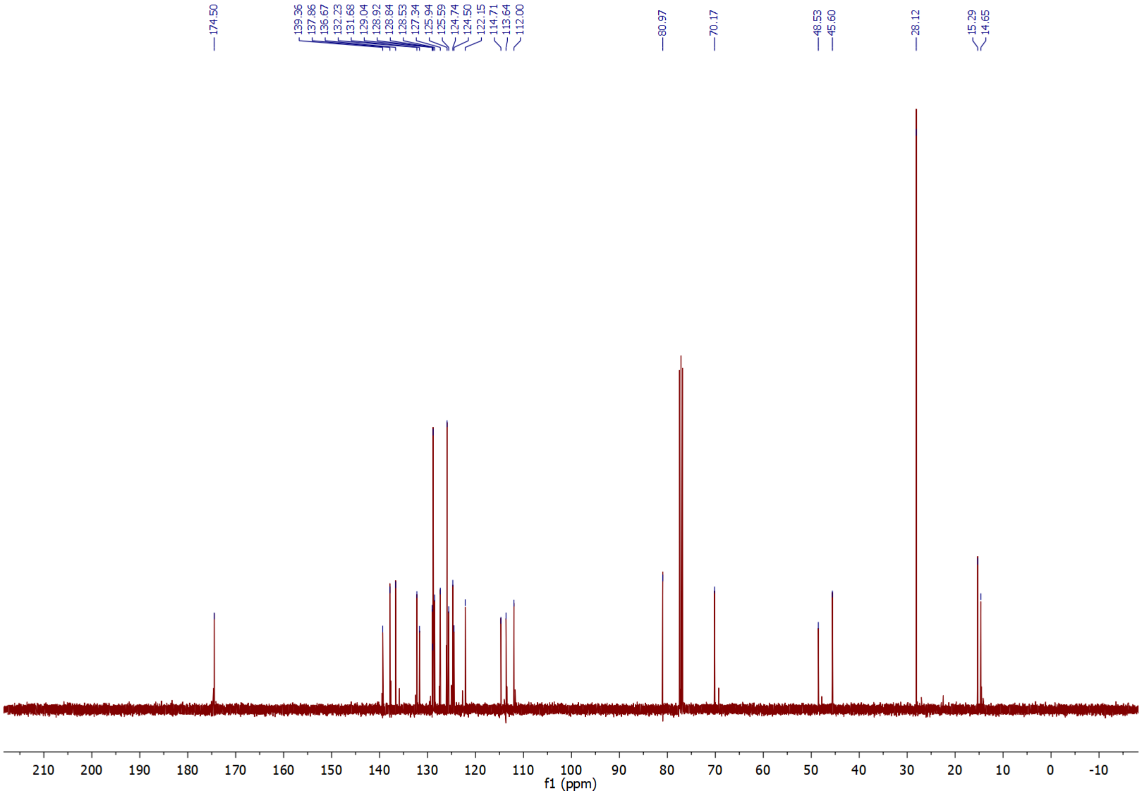

Figure S146. Racemic sample of 2l: IA column, n-Hex/i-PrOH 95:5, T= 30°C, F= 1.0 mL/min.

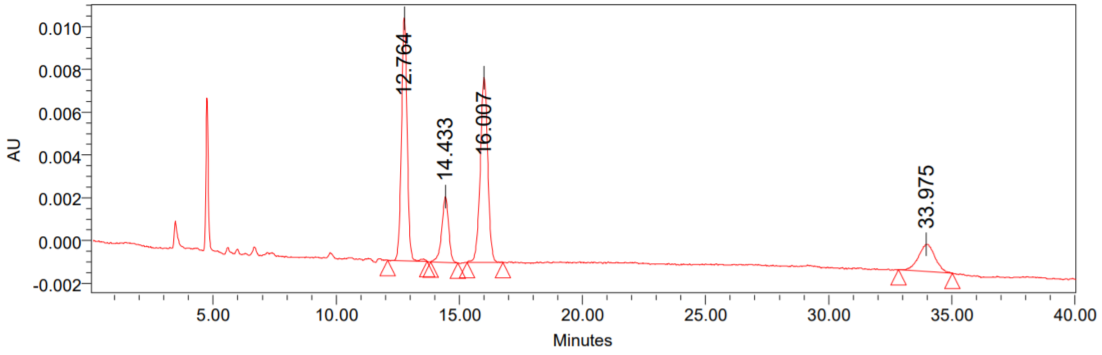

Processed Channel: PDA 274.8 nm

	Processed Channel	Retention Time (min)	Area	% Area	Height
1	PDA 274.8 nm	12.764	182064	37.63	11347
2	PDA 274.8 nm	14.433	59558	12.31	3068
3	PDA 274.8 nm	16.007	182880	37.80	8634
4	PDA 274.8 nm	33.975	59270	12.25	1274

Figure S147. Enantioenriched sample of (2*S*,3*R*,*S*$_a$)-2l: IA column, n-Hex/i-PrOH 95:5, T= 30°C, F= 1.0 mL/min.

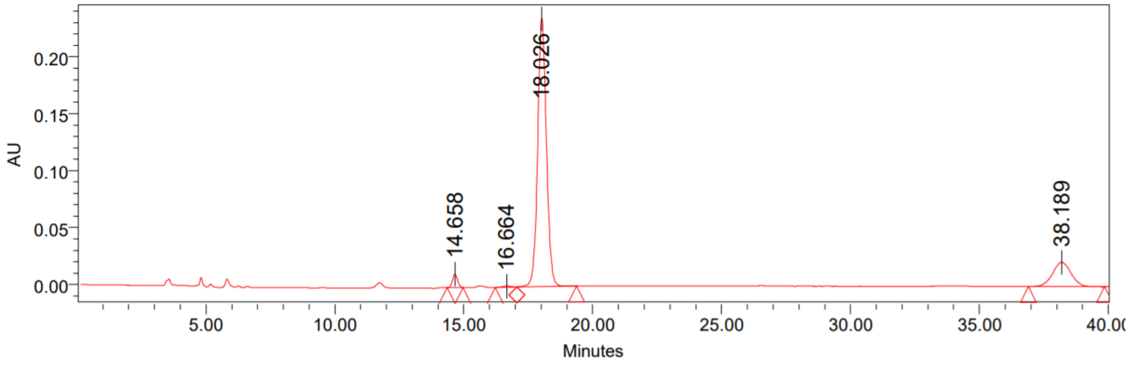

Processed Channel: PDA 255.3 nm

	Processed Channel	Retention Time (min)	Area	% Area	Height
1	PDA 255.3 nm	14.658	164749	2.43	11820
2	PDA 255.3 nm	16.664	20391	0.30	980
3	PDA 255.3 nm	18.026	5532249	81.47	235548
4	PDA 255.3 nm	38.189	1073376	15.81	21367

Figure S148. ^{1}H-NMR (400 MHz, $CDCl_3$) of **(2*S*,3*R*,*S*$_a$)-2m**:

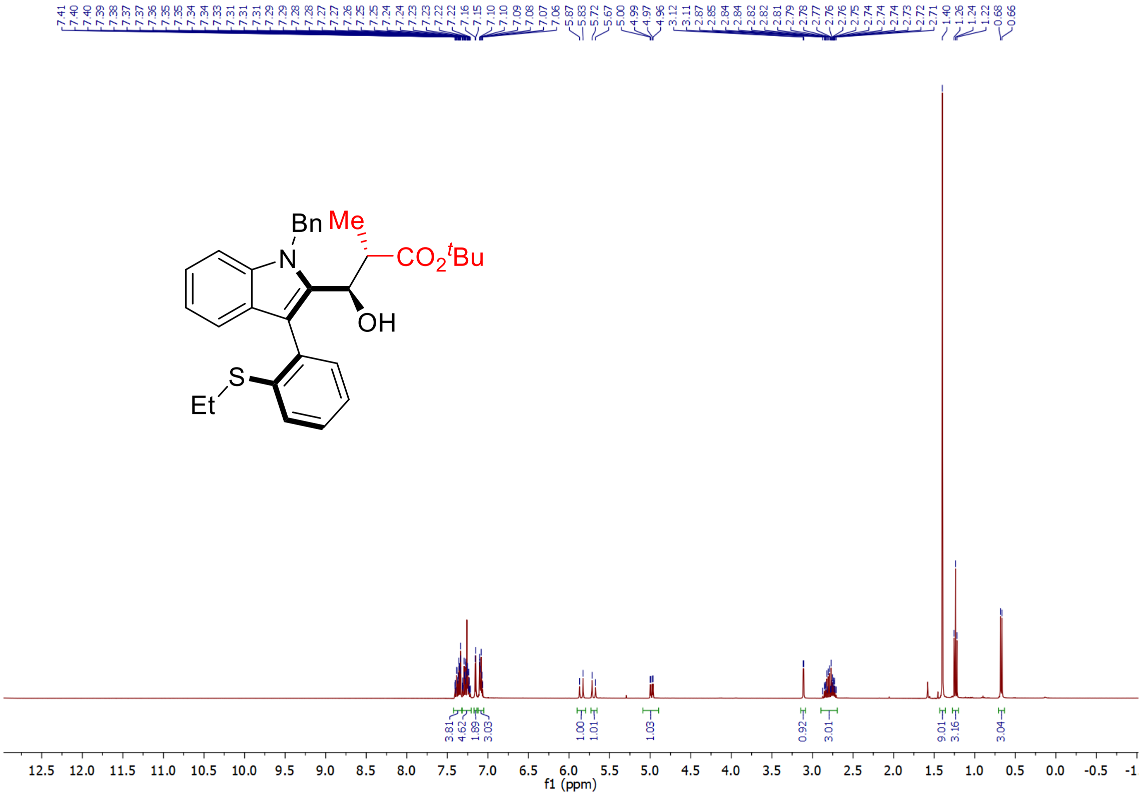

Figure S149. ^{13}C-NMR (100 MHz, $CDCl_3$) of **(2*S*,3*R*,*S*$_a$)-2m**:

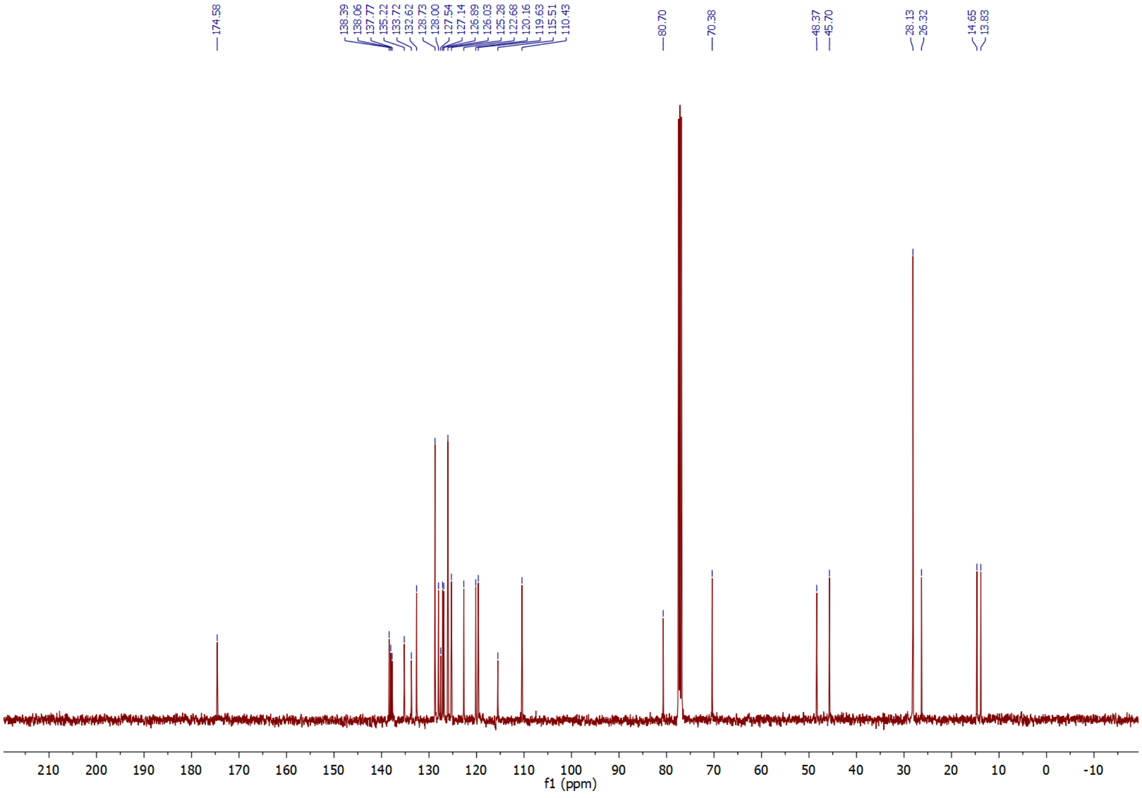

Figure S150. Racemic sample of 2m: IA column, n-Hex/i-PrOH 95:5, T= 30°C, F= 1.0 mL/min.

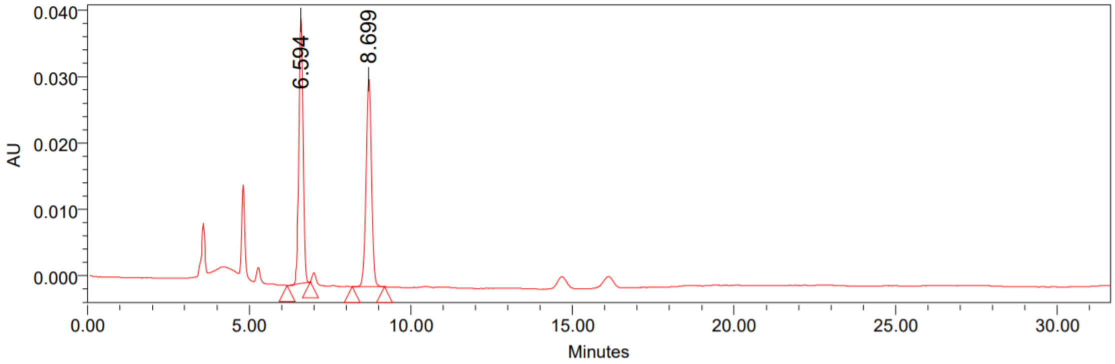

Processed Channel: PDA 259.9 nm

	Processed Channel	Retention Time (min)	Area	% Area	Height
1	PDA 259.9 nm	6.594	364487	49.46	39910
2	PDA 259.9 nm	8.699	372493	50.54	31367

Figure S151. Enantioenriched sample of (2*S*,3*R*,*S*$_a$)-2m: IA column, n-Hex/i-PrOH 95:5, T= 30°C, F= 1.0 mL/min.

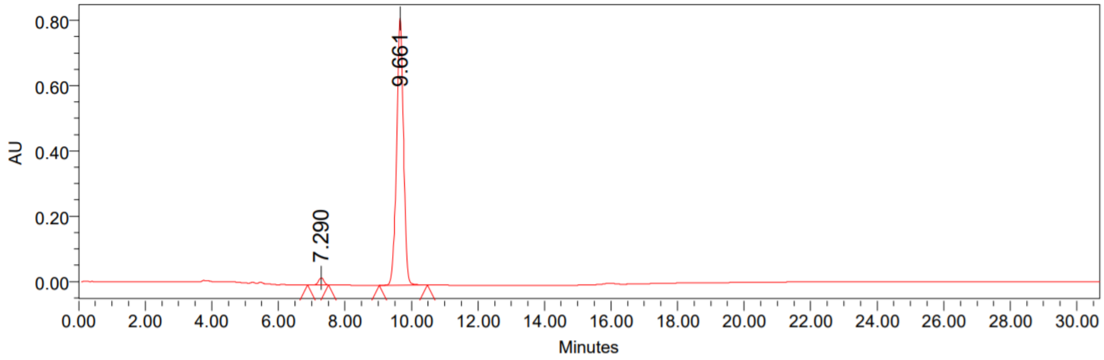

Processed Channel: PDA 234.6 nm

	Processed Channel	Retention Time (min)	Area	% Area	Height
1	PDA 234.6 nm	7.290	240578	1.99	21772
2	PDA 234.6 nm	9.661	11821016	98.01	817559

Figure S152. ^{1}H-NMR (400 MHz, $CDCl_3$) of **(2*S*,3*R*,*S*$_a$)-2n**:

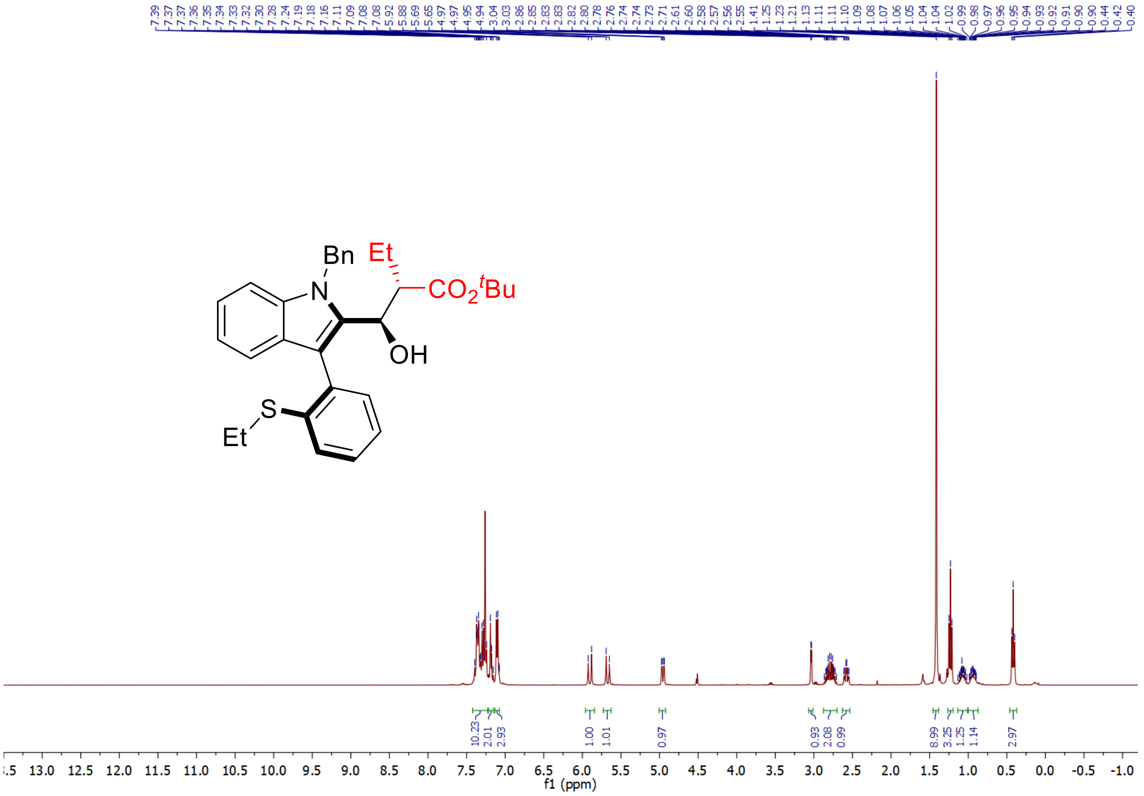

Figure S153. ^{13}C-NMR (100 MHz, $CDCl_3$) of **(2*S*,3*R*,*S*$_a$)-2n**:

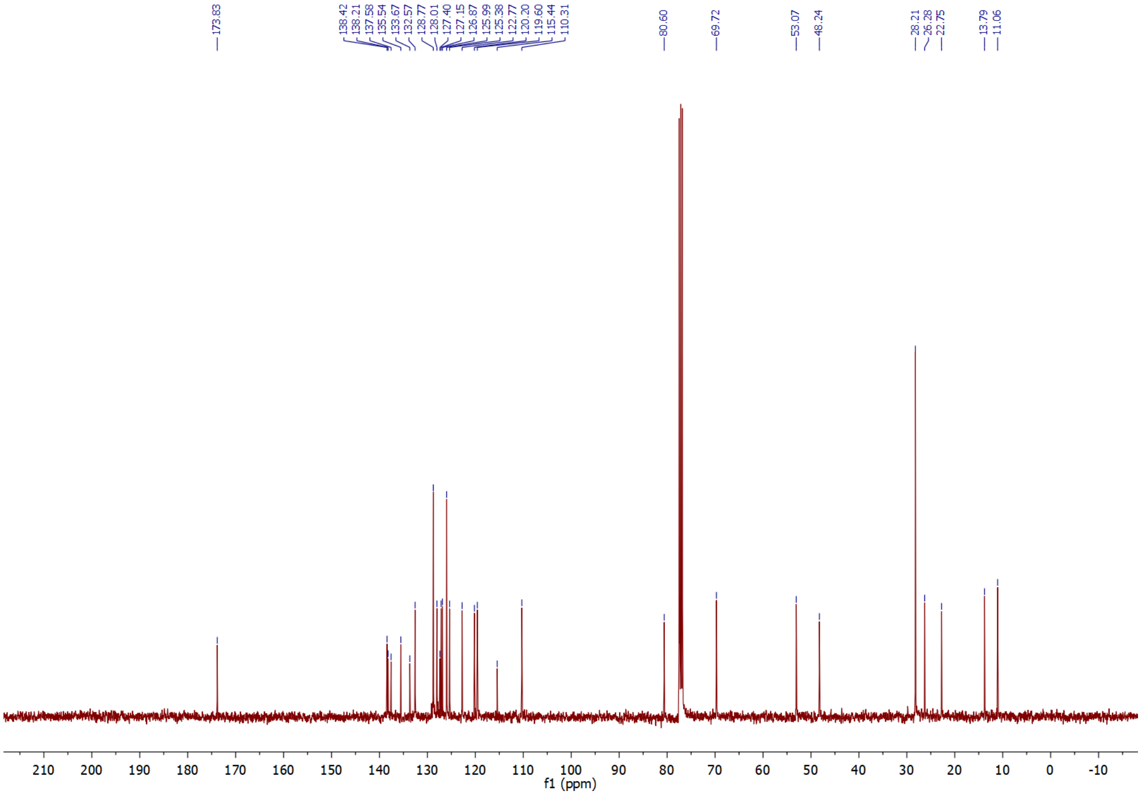

Figure S154. Enantioenriched sample of (2*S*,3*R*,*S*$_a$)-2n: IA column, n-Hex/i-PrOH 95:5, T= 30°C, F= 1.0 mL/min.

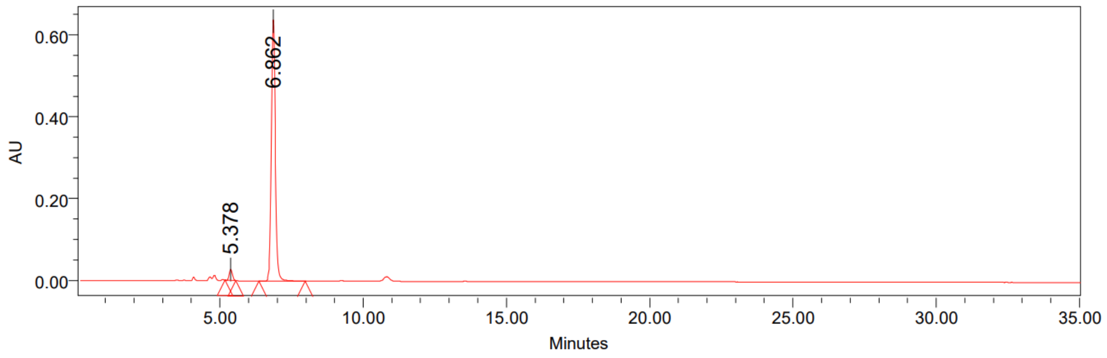

Processed Channel: PDA 277.7 nm

	Processed Channel	Retention Time (min)	Area	% Area	Height
1	PDA 277.7 nm	5.378	202938	3.20	26900
2	PDA 277.7 nm	6.862	6142826	96.80	638039

Figure S155. ^{1}H-NMR (400 MHz, $CDCl_3$) of **(2*S*,3*R*,*S*$_a$)-2o**:

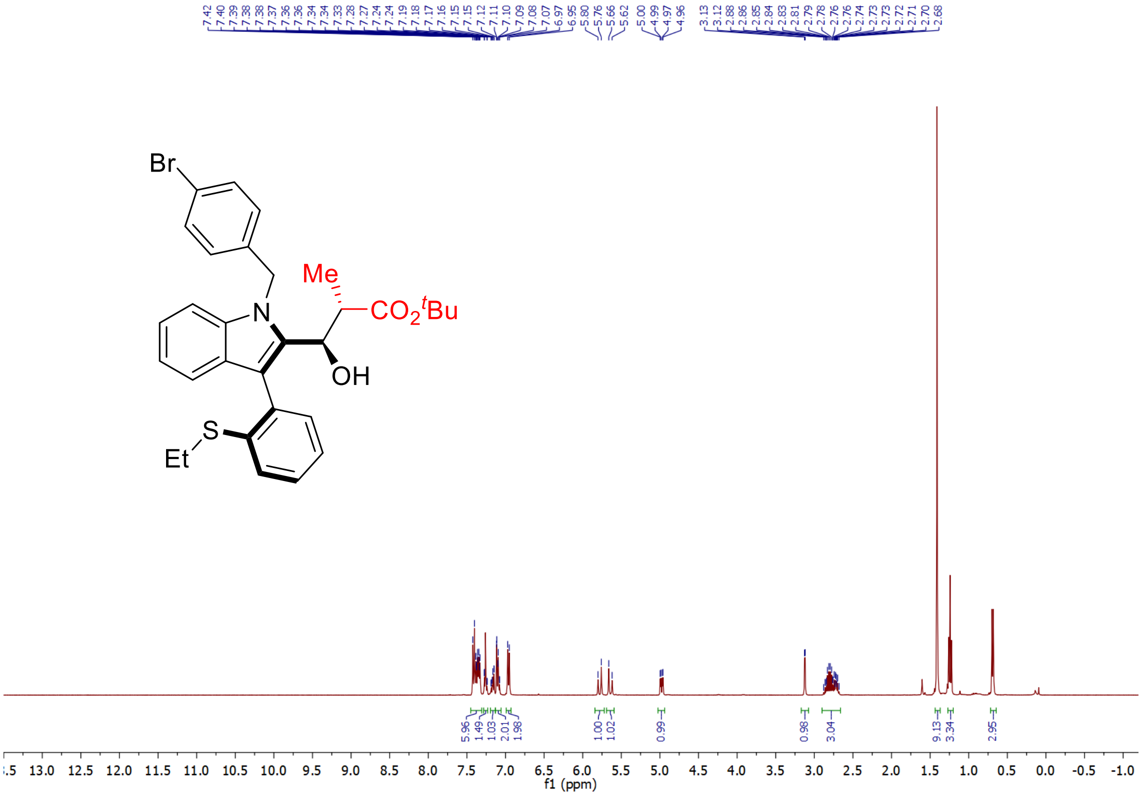

Figure S156. ^{13}C-NMR (100 MHz, $CDCl_3$) of **(2*S*,3*R*,*S*$_a$)-2o**:

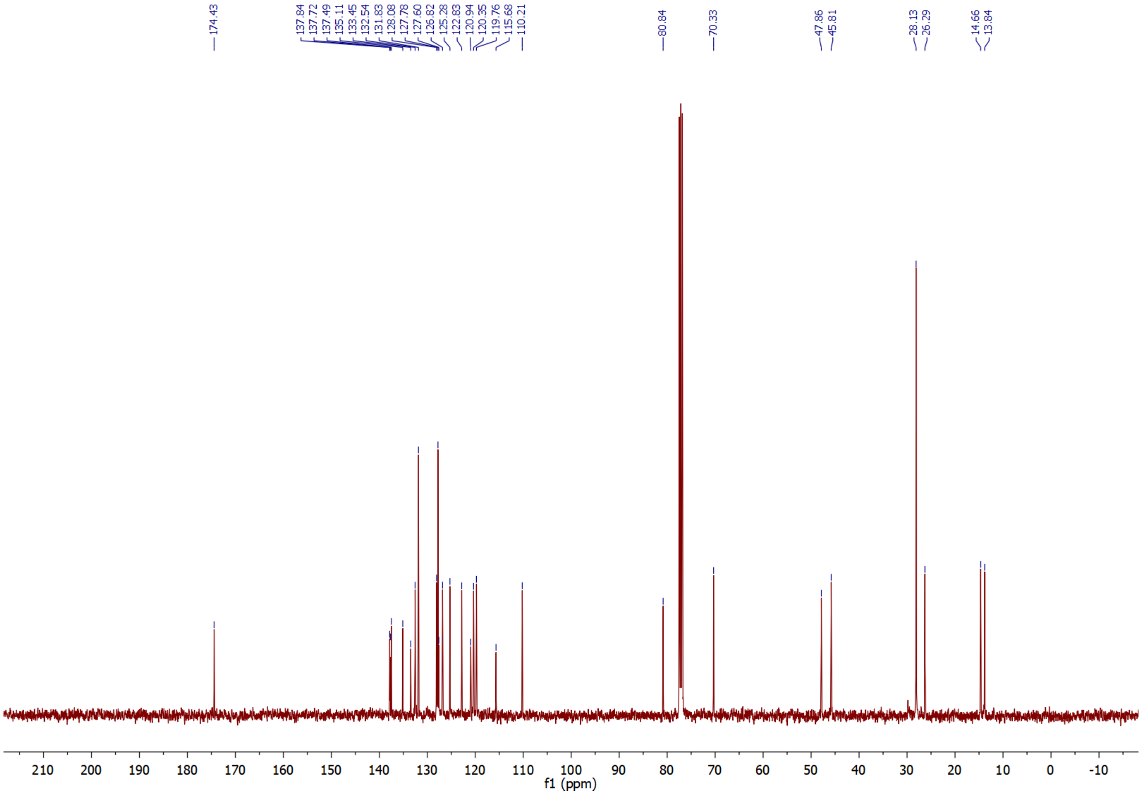

Figure S157. Racemic sample of 2o: IA column, n-Hex/i-PrOH 95:5, T= 30°C, F= 1.0 mL/min.

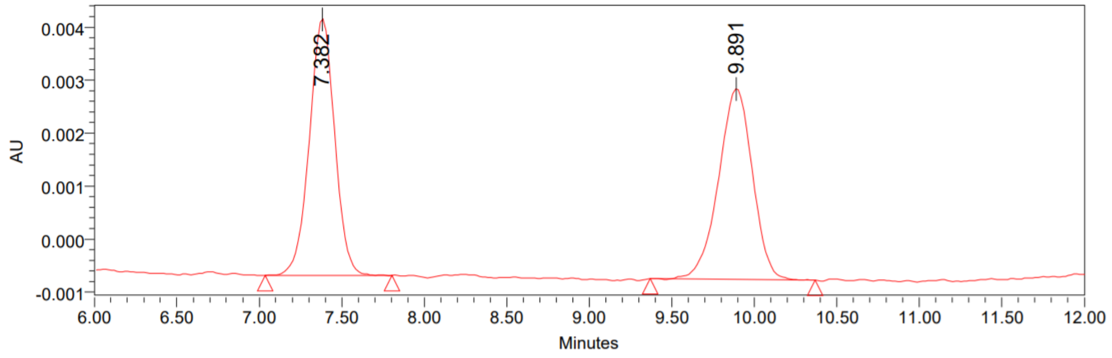

Processed Channel: PDA 290.2 nm

	Processed Channel	Retention Time (min)	Area	% Area	Height
1	PDA 290.2 nm	7.382	51787	50.07	4850
2	PDA 290.2 nm	9.891	51646	49.93	3605

Figure S158. Enantioenriched sample of (2*S*,3*R*,S_a)-2o: IA column, n-Hex/i-PrOH 95:5, T= 30°C, F= 1.0 mL/min.

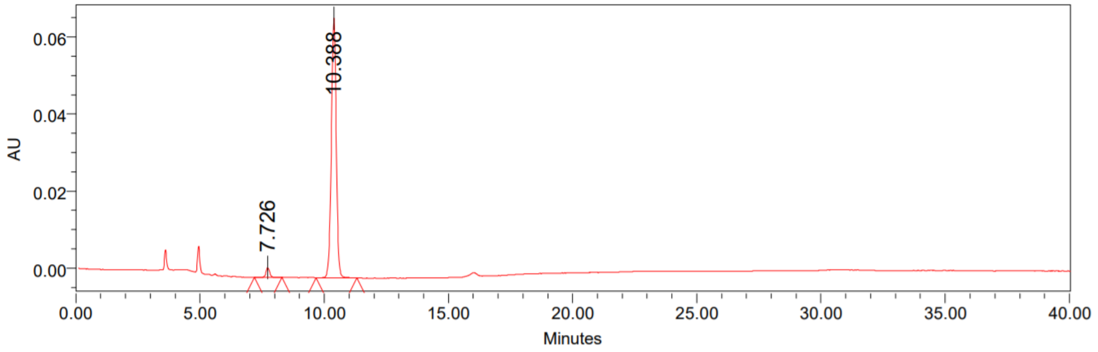

Processed Channel: PDA 252.2 nm

	Processed Channel	Retention Time (min)	Area	% Area	Height
1	PDA 252.2 nm	7.726	26260	2.77	2569
2	PDA 252.2 nm	10.388	921582	97.23	67756

Figure S159. ^{1}H-NMR (400 MHz, CDCl$_3$) of **(2*S*,3*R*,*S*$_a$)-2p**:

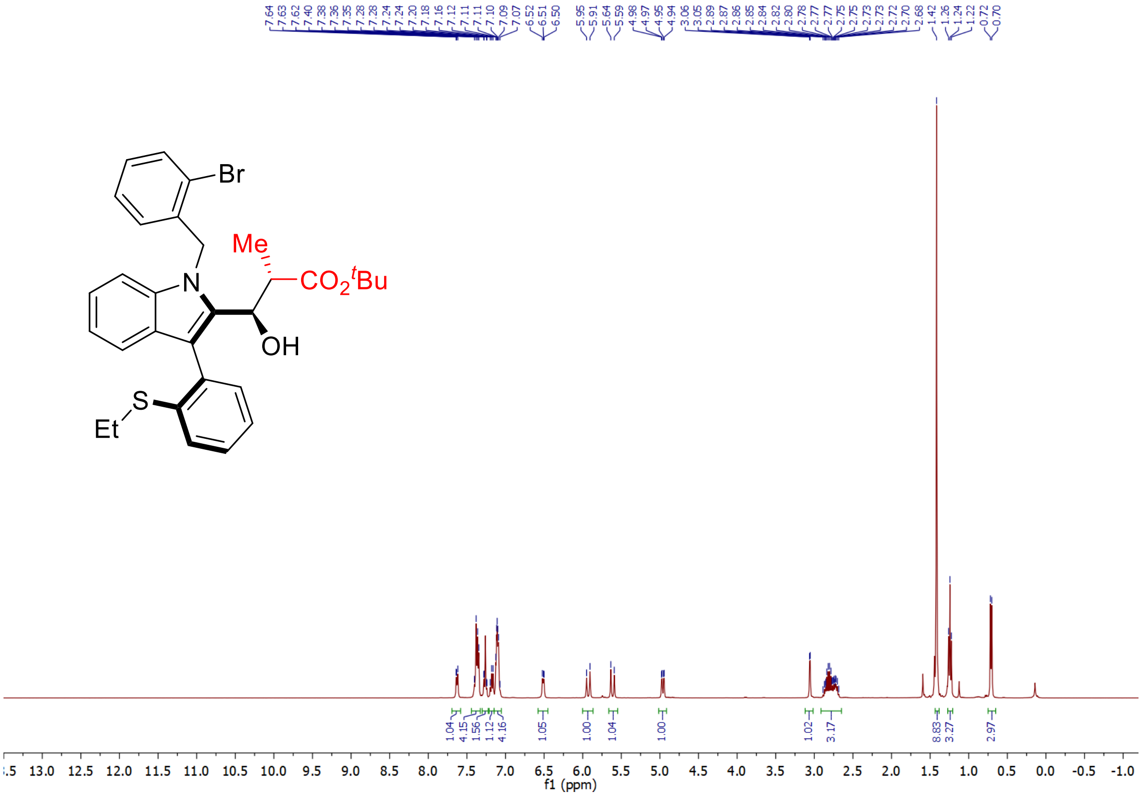

Figure S160. ^{13}C-NMR (100 MHz, CDCl$_3$) of **(2*S*,3*R*,*S*$_a$)-2p**:

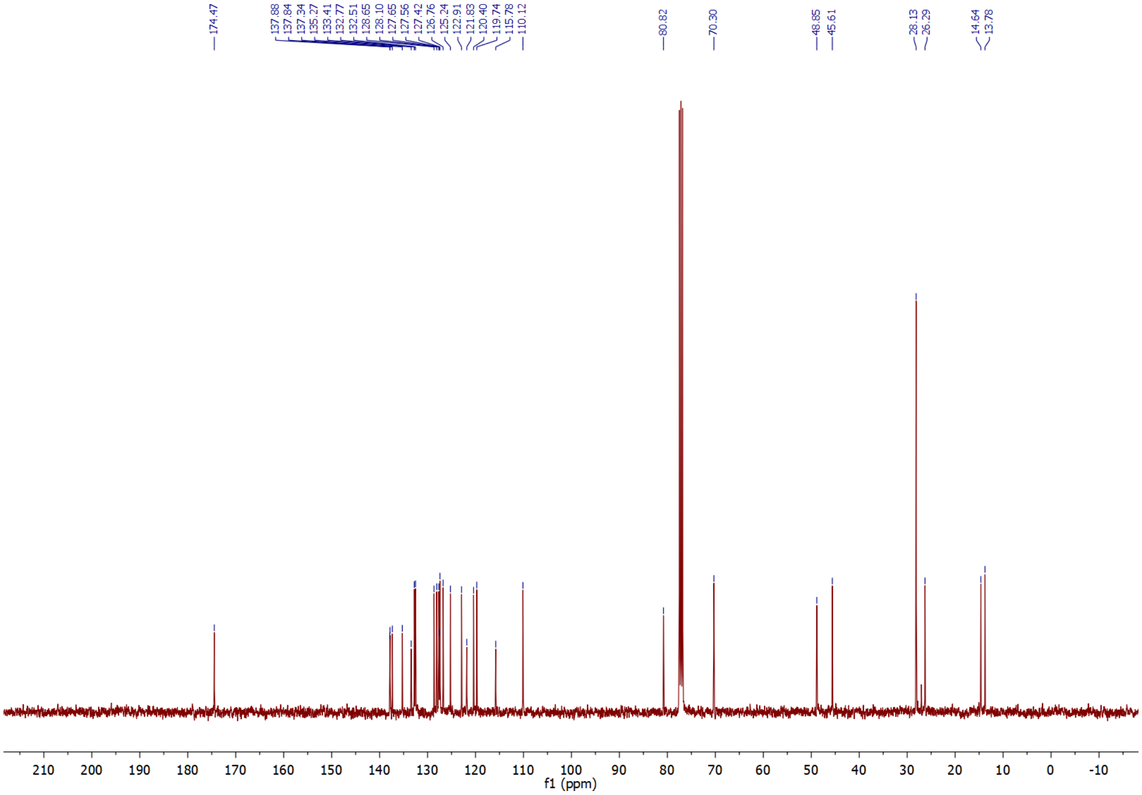

Figure S161. Racemic sample of 2p: IB column, n-Hex/i-PrOH 98:2, T= 30°C, F= 1.0 mL/min.

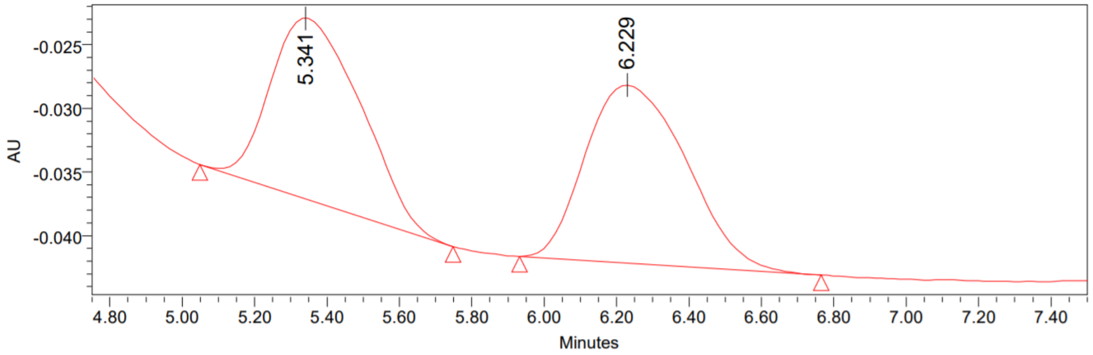

Processed Channel: PDA 218.0 nm

	Processed Channel	Retention Time (min)	Area	% Area	Height
1	PDA 218.0 nm	5.341	248662	47.82	14201
2	PDA 218.0 nm	6.229	271334	52.18	13975

Figure S162. Enantioenriched sample of (2*S*,3*R*,*S*$_a$)-2p: IB column, n-Hex/i-PrOH 98:2, T= 30°C, F= 1.0 mL/min.

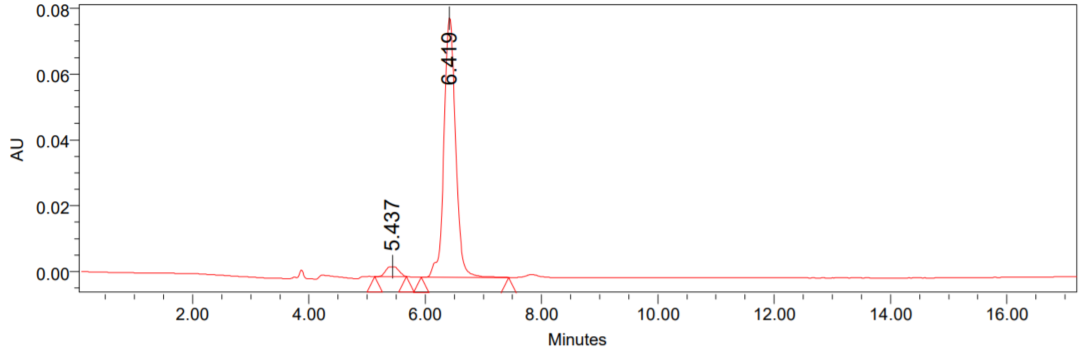

Processed Channel: PDA 263.5 nm

	Processed Channel	Retention Time (min)	Area	% Area	Height
1	PDA 263.5 nm	5.437	46532	4.07	2990
2	PDA 263.5 nm	6.419	1097629	95.93	78946

Figure S163. ^{1}H-NMR (400 MHz, $CDCl_3$) of **(2*S*,3*R*,*S*$_a$)-2q**:

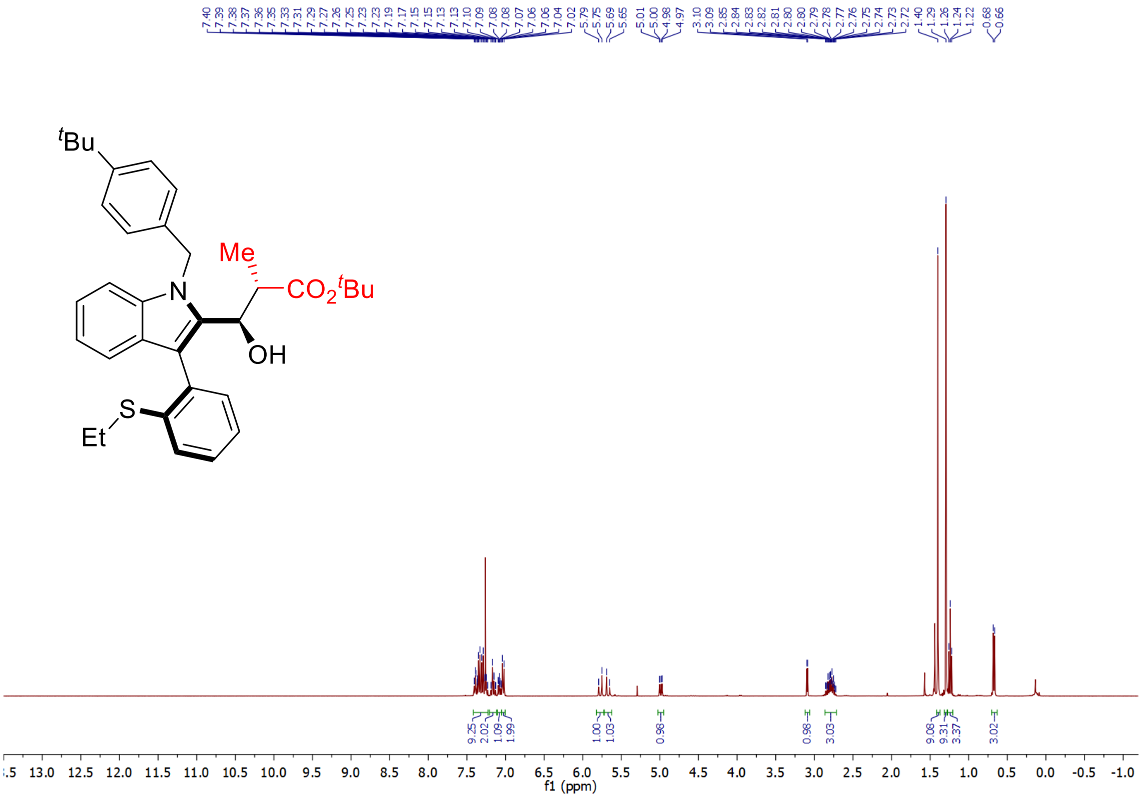

Figure S164. ^{13}C-NMR (100 MHz, $CDCl_3$) of **(2*S*,3*R*,*S*$_a$)-2q**:

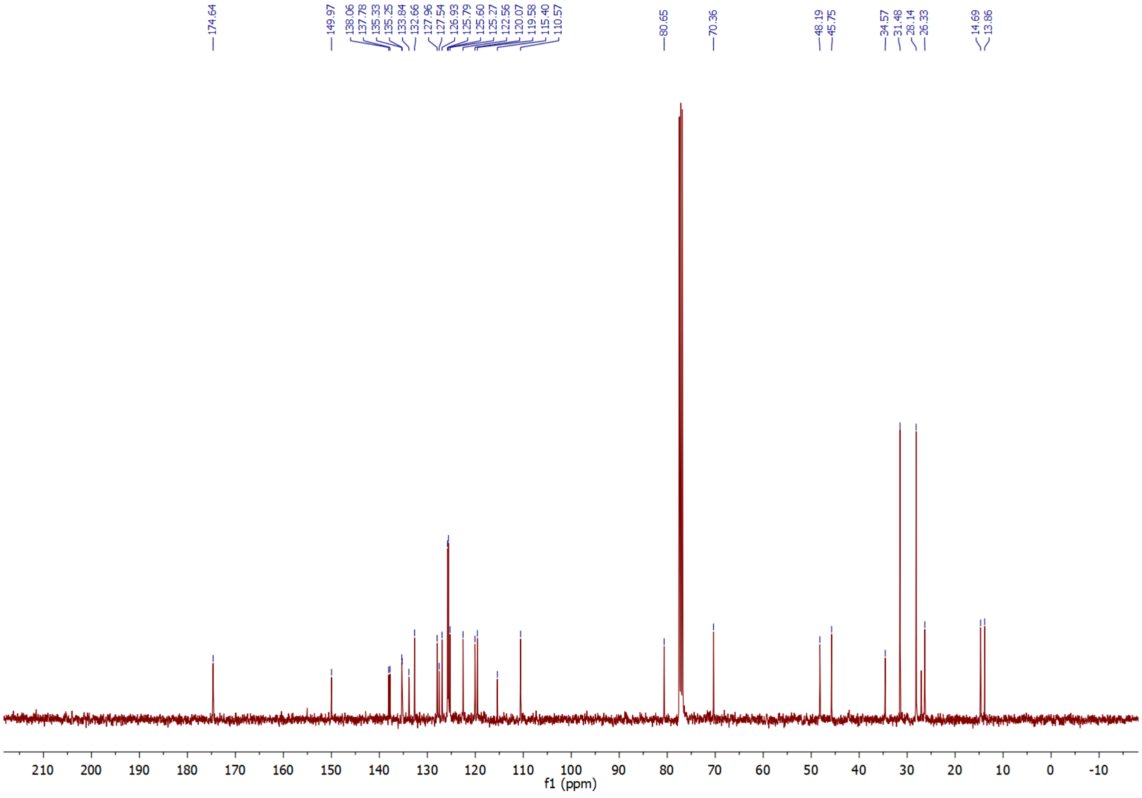

Figure S165. Racemic sample of 2q: IA column, n-Hex/i-PrOH 95:5, T= 30°C, F= 1.0 mL/min.

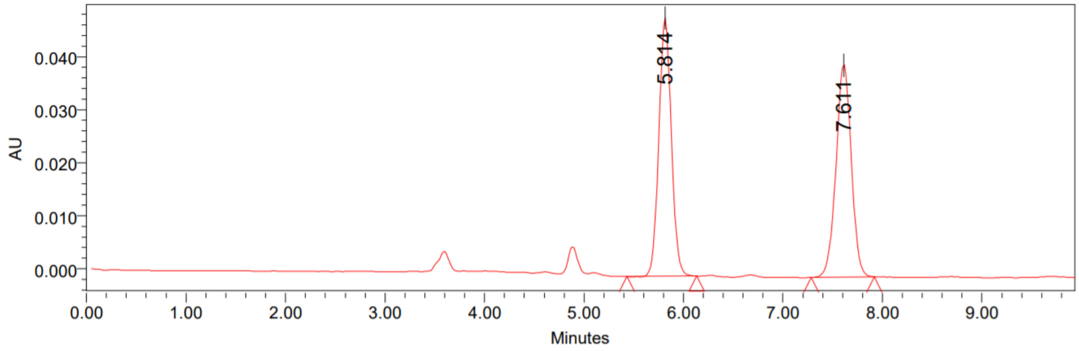

Processed Channel: PDA 259.3 nm

	Processed Channel	Retention Time (min)	Area	% Area	Height
1	PDA 259.3 nm	5.814	427164	49.97	48680
2	PDA 259.3 nm	7.611	427675	50.03	40078

Figure S166. Enantioenriched sample of (2*S*,3*R*,*S*$_a$)-2q: IA column, n-Hex/i-PrOH 95:5, T= 30°C, F= 1.0 mL/min.

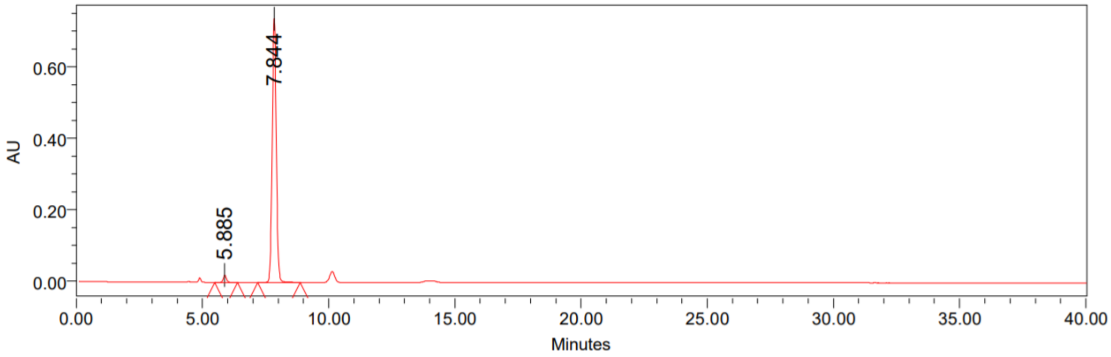

Processed Channel: PDA 272.1 nm

	Processed Channel	Retention Time (min)	Area	% Area	Height
1	PDA 272.1 nm	5.885	181081	2.14	20332
2	PDA 272.1 nm	7.844	8264566	97.86	738282

Figure S167. ^{1}H-NMR (400 MHz, $CDCl_3$) of **(2*S*,3*R*,*S*$_a$)-2r**:

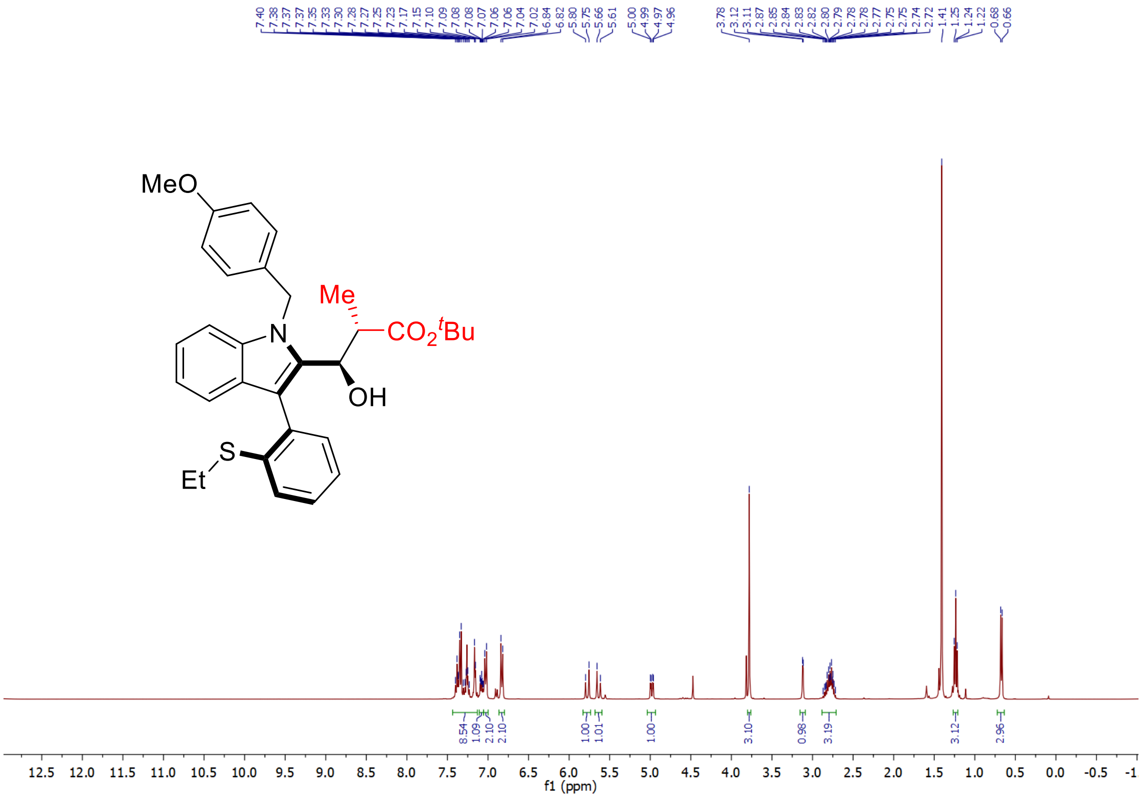

Figure S168. ^{13}C-NMR (100 MHz, $CDCl_3$) of **(2*S*,3*R*,*S*$_a$)-2r**:

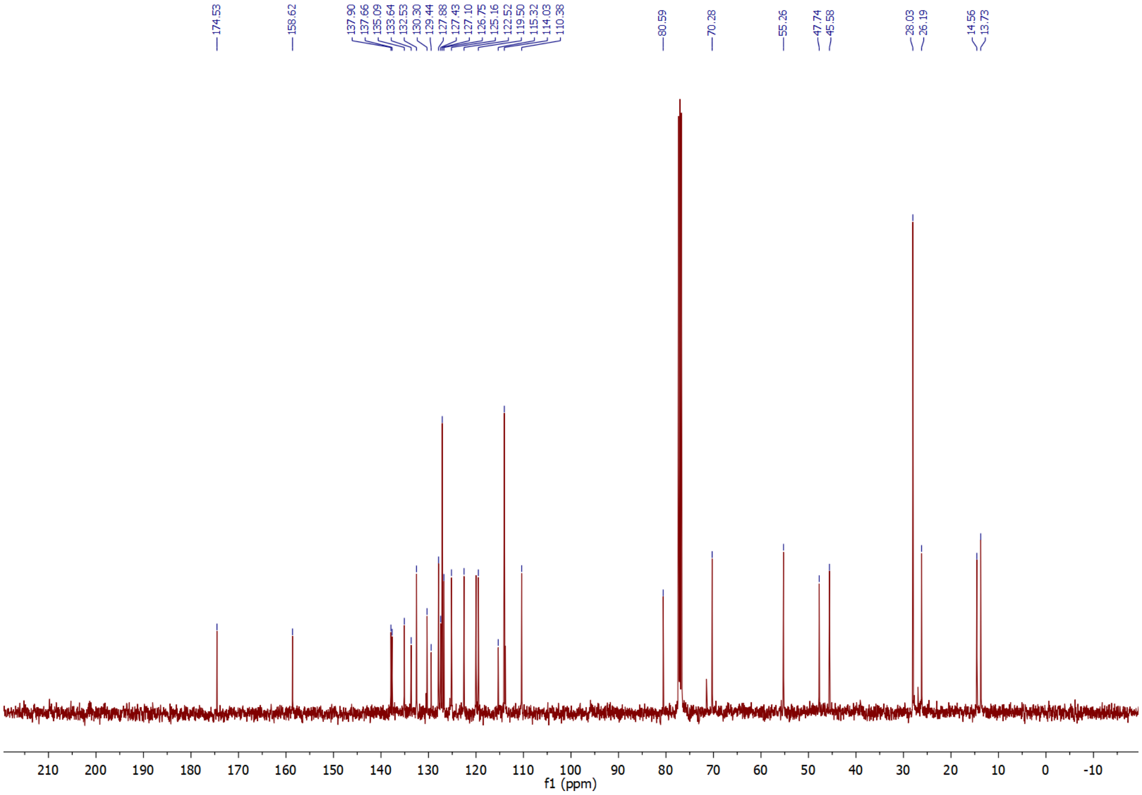

Figure S169. Racemic sample of 2r: IA column, n-Hex/i-PrOH 95:5, T= 30°C, F= 1.0 mL/min.

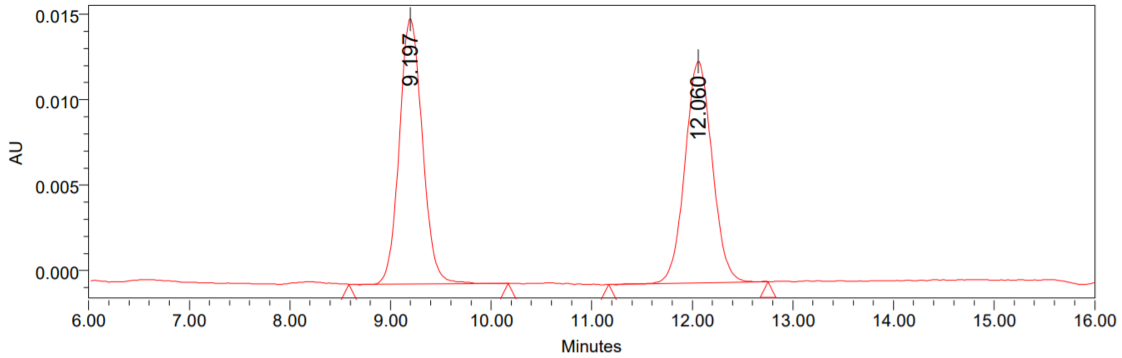

Processed Channel: PDA 270.5 nm

	Processed Channel	Retention Time (min)	Area	% Area	Height
1	PDA 270.5 nm	9.197	247431	50.40	15543
2	PDA 270.5 nm	12.060	243461	49.60	12979

Figure S170. Enantioenriched sample of (2*S*,3*R*,*S*$_a$)-2r: IA column, n-Hex/i-PrOH 95:5, T= 30°C, F= 1.0 mL/min.

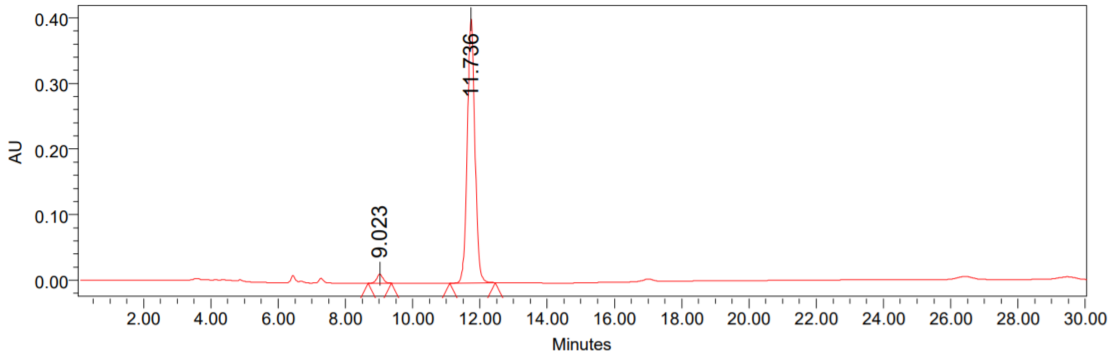

Processed Channel: PDA 244.3 nm

	Processed Channel	Retention Time (min)	Area	% Area	Height
1	PDA 244.3 nm	9.023	175648	2.69	13956
2	PDA 244.3 nm	11.736	6347683	97.31	402938

Figure S171. ^{1}H-NMR (400 MHz, $CDCl_3$) of **(2*S*,3*R*,*S*$_a$)-2s**:

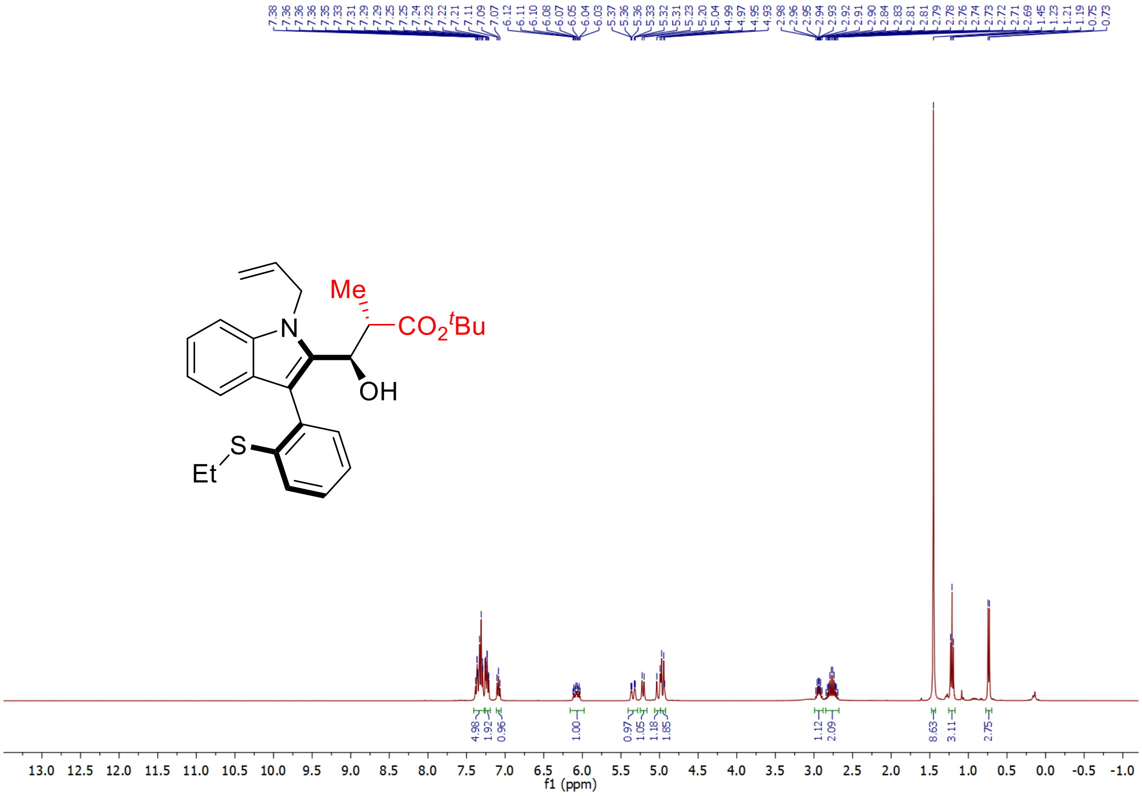

Figure S172. ^{13}C-NMR (100 MHz, $CDCl_3$) of **(2*S*,3*R*,*S*$_a$)-2s**:

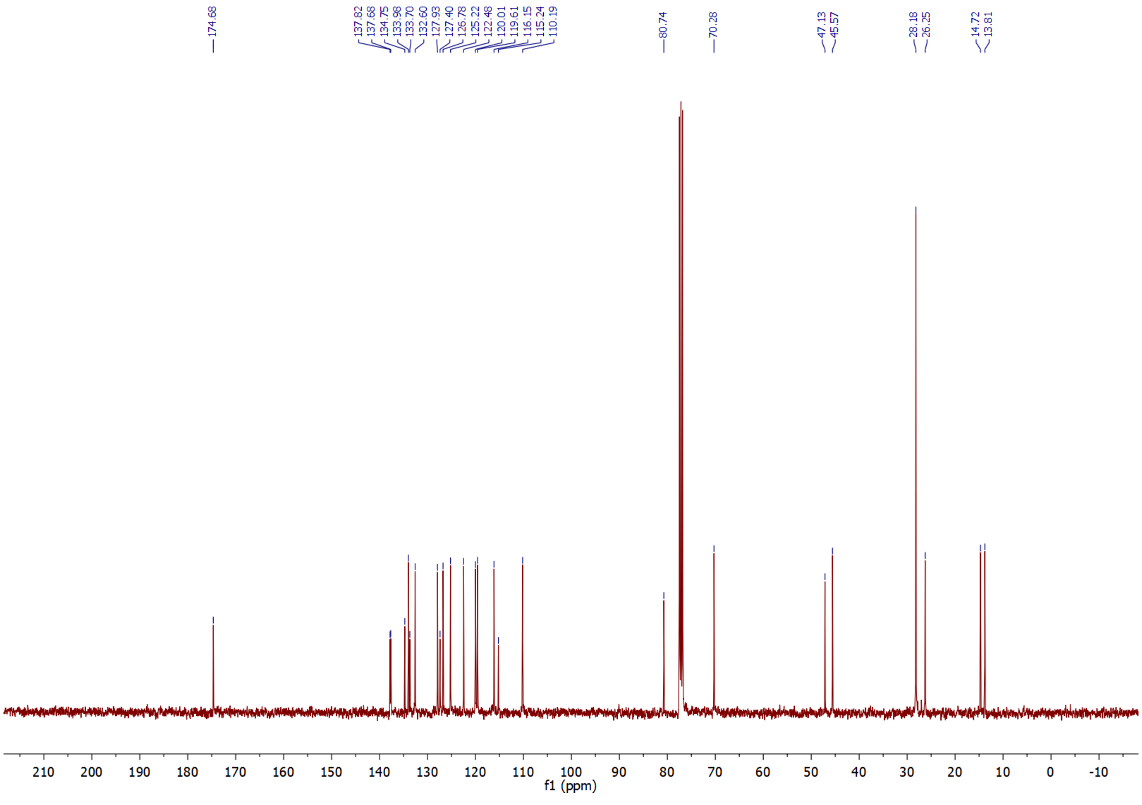

Figure S173. Racemic sample of 2s: IA column, n-Hex/i-PrOH 95:5, T= 30°C, F= 1.0 mL/min.

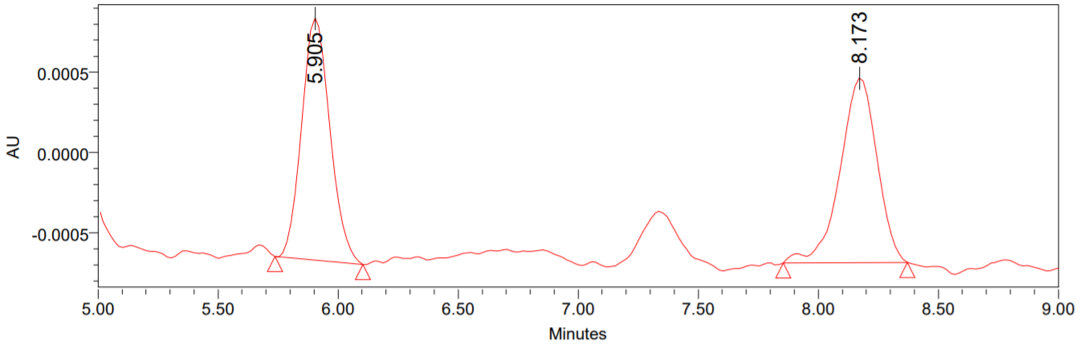

Processed Channel: PDA 296.7 nm

	Processed Channel	Retention Time (min)	Area	% Area	Height
1	PDA 296.7 nm	5.905	12507	50.54	1508
2	PDA 296.7 nm	8.173	12242	49.46	1150

Figure S174. Enantioenriched sample of (2*S*,3*R*,*S*$_a$)-2s: IA column, n-Hex/i-PrOH 95:5, T= 30°C, F= 1.0 mL/min.

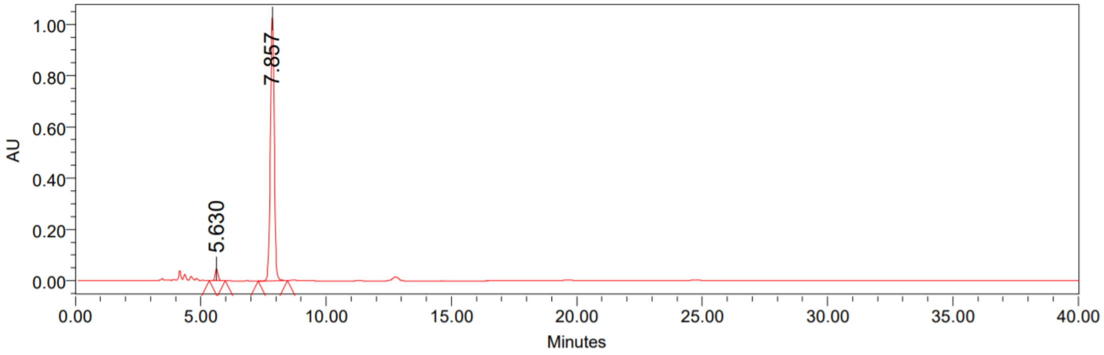

Processed Channel: PDA 262.0 nm

	Processed Channel	Retention Time (min)	Area	% Area	Height
1	PDA 262.0 nm	5.630	378273	3.38	47578
2	PDA 262.0 nm	7.857	10808283	96.62	1028166

Figure S175. ^{1}H-NMR (400 MHz, $CDCl_3$) of **(2*S*,3*R*,*S*$_a$)-2t**:

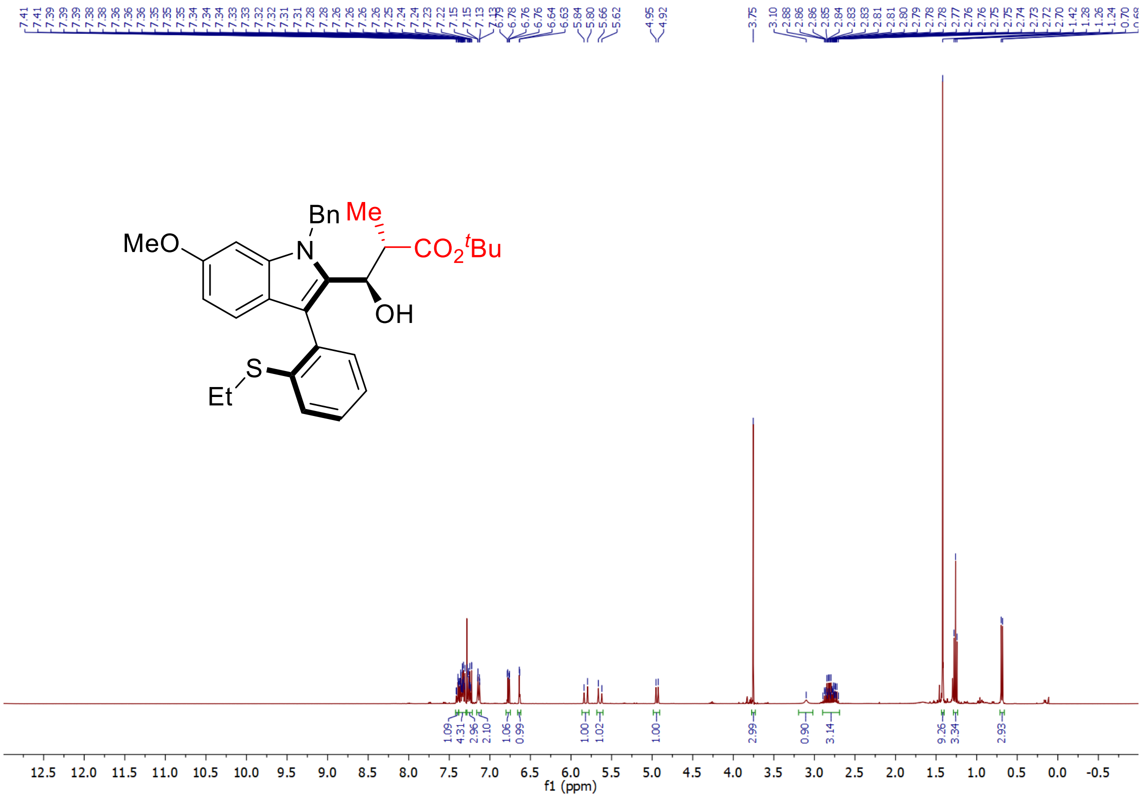

Figure S176. ^{13}C-NMR (100 MHz, $CDCl_3$) of **(2*S*,3*R*,*S*$_a$)-2t**:

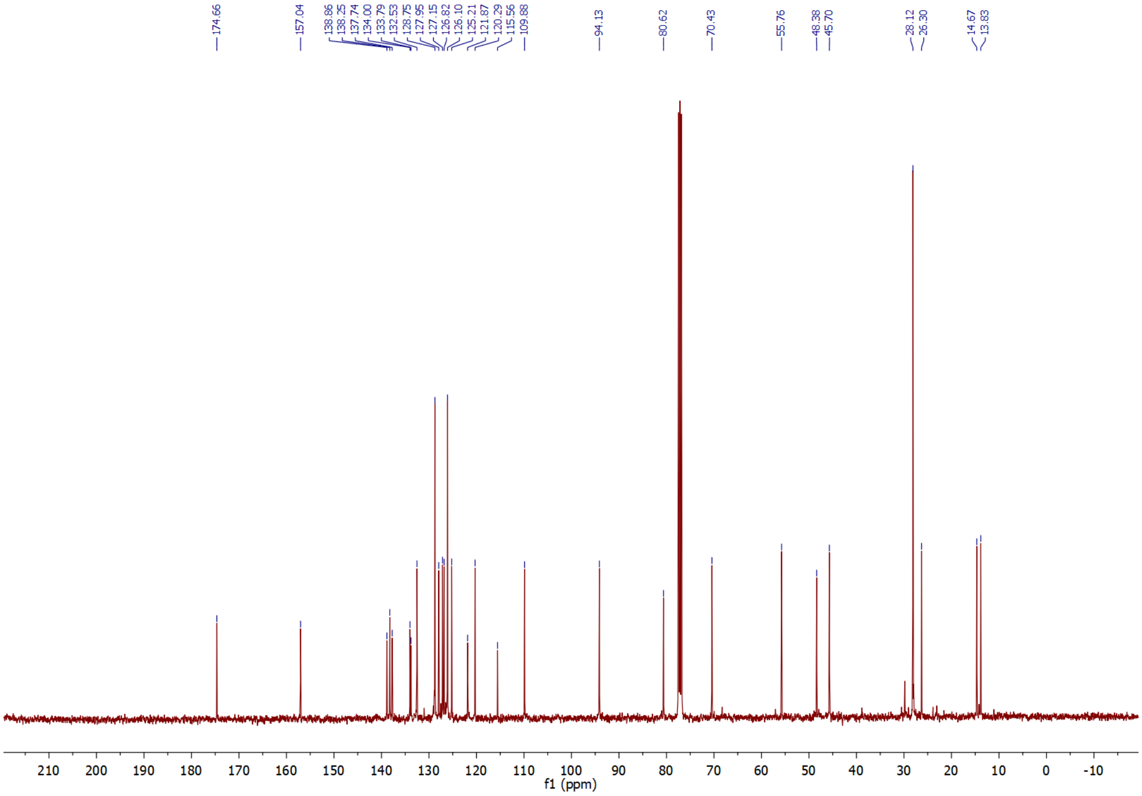

Figure S177. Racemic sample of 2t: IA column, n-Hex/i-PrOH 95:5, T= 30°C, F= 1.0 mL/min.

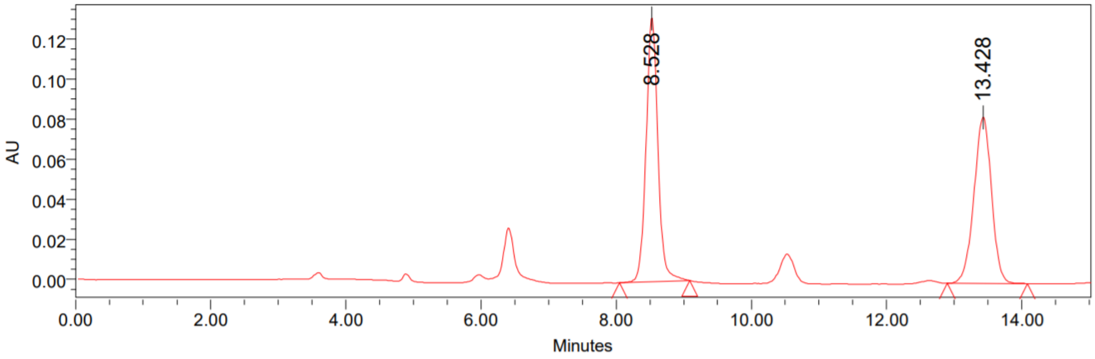

Processed Channel: PDA 252.2 nm

	Processed Channel	Retention Time (min)	Area	% Area	Height
1	PDA 252.2 nm	8.528	1615045	51.67	132276
2	PDA 252.2 nm	13.428	1510905	48.33	83352

Figure S178. Enantioenriched sample of (2*S*,3*R*,*S*$_a$)-2t: IA column, n-Hex/i-PrOH 95:5, T= 30°C, F= 1.0 mL/min.

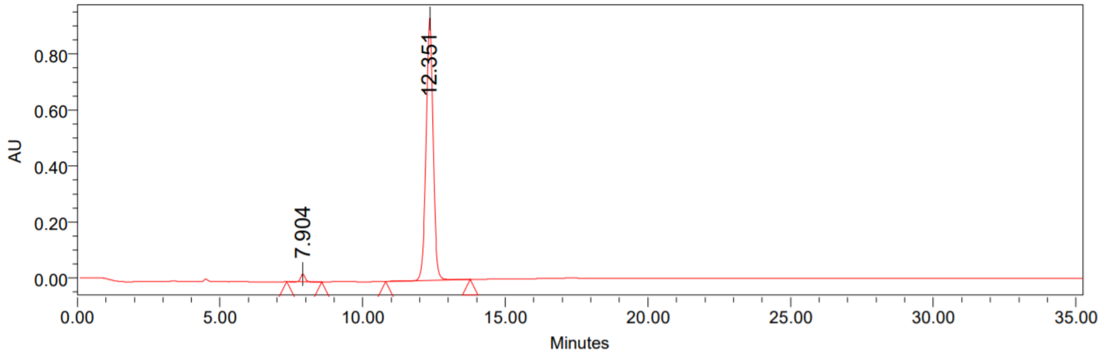

Processed Channel: PDA 232.5 nm

	Processed Channel	Retention Time (min)	Area	% Area	Height
1	PDA 232.5 nm	7.904	297920	1.82	27163
2	PDA 232.5 nm	12.351	16098007	98.18	936766

Figure S179. ^{1}H-NMR (400 MHz, $CDCl_3$) of **(2*S*,3*R*,*S*$_a$)-2u**:

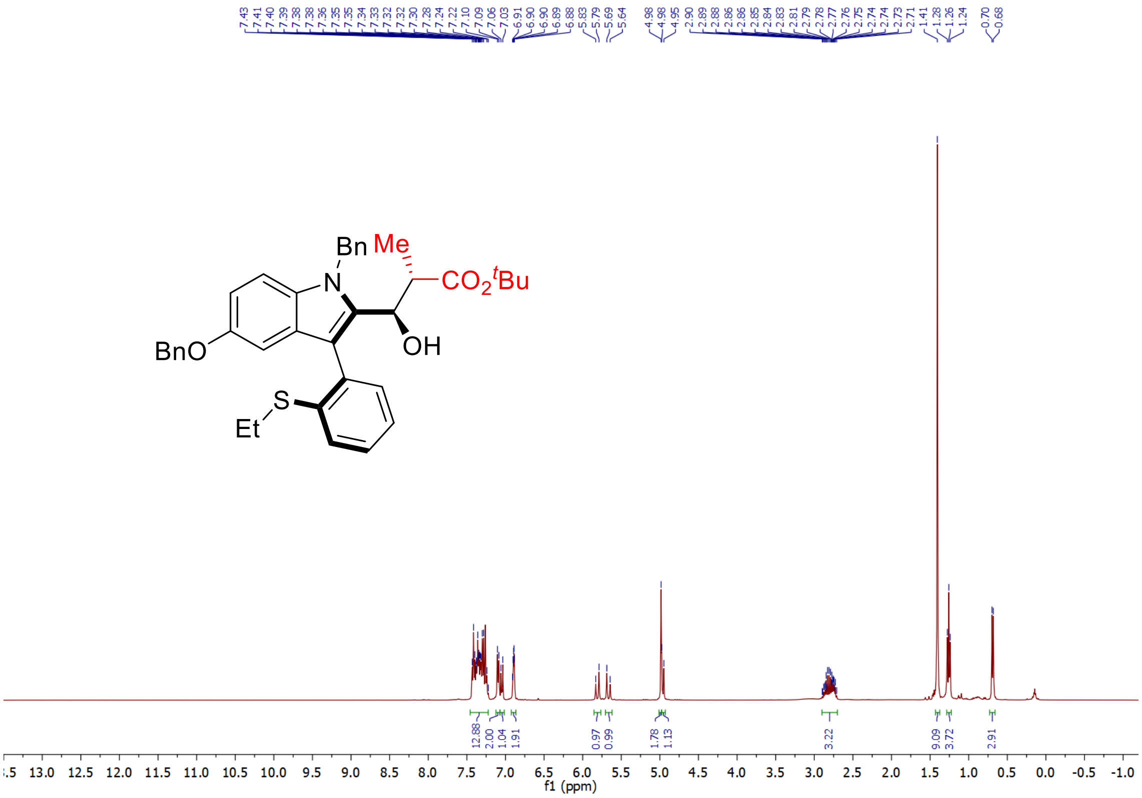

Figure S180. ^{13}C-NMR (100 MHz, $CDCl_3$) of **(2*S*,3*R*,*S*$_a$)-2u**:

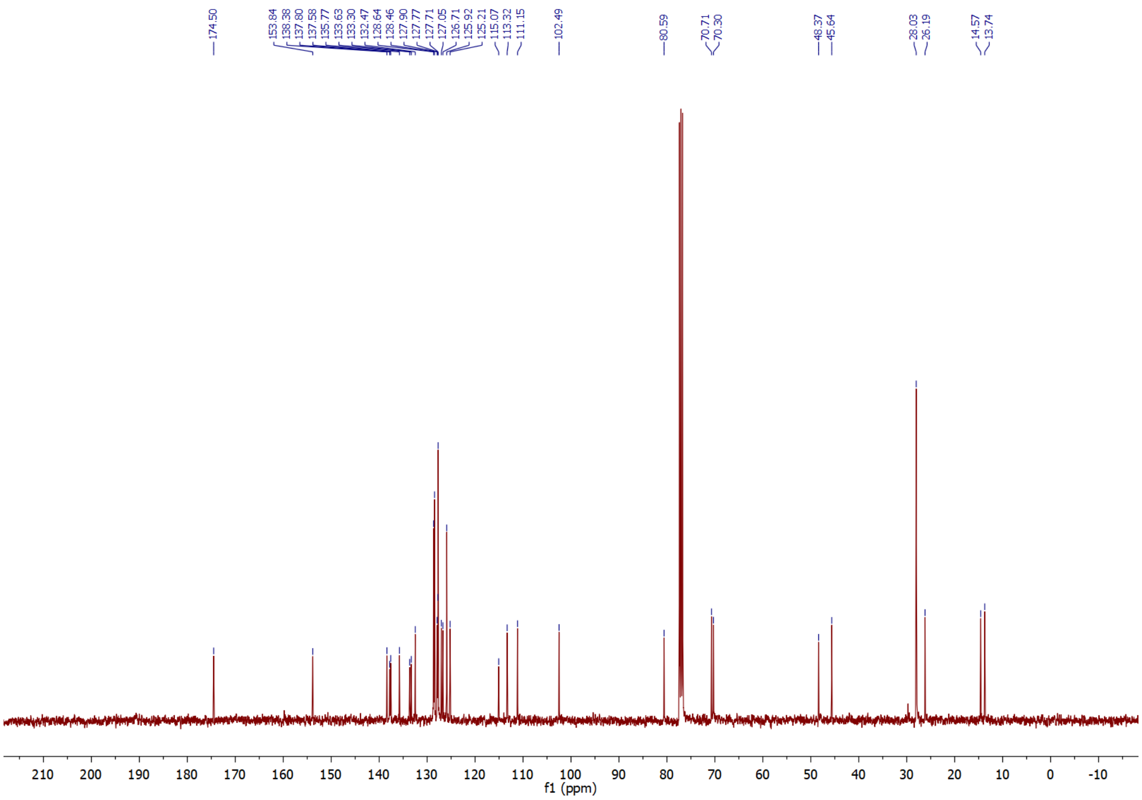

Figure S181. Racemic sample of 2u: IA column, n-Hex/i-PrOH 95:5, T= 30°C, F= 1.0 mL/min.

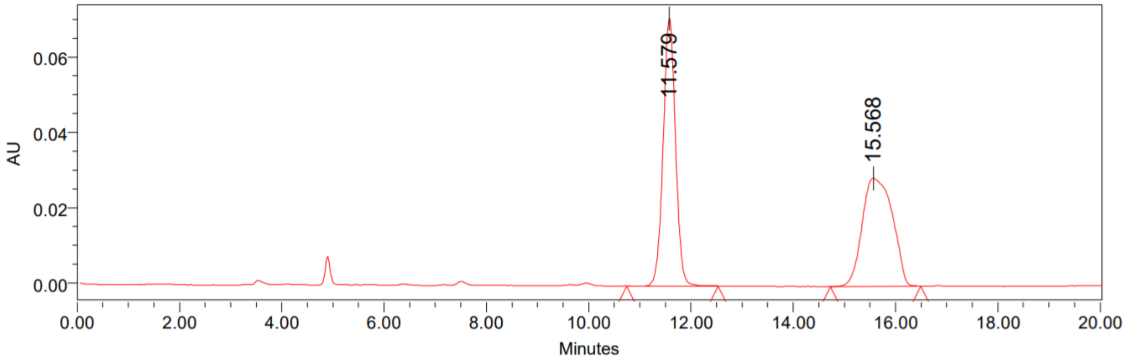

Processed Channel: PDA 274.6 nm

	Processed Channel	Retention Time (min)	Area	% Area	Height
1	PDA 274.6 nm	11.579	1189021	50.21	71023
2	PDA 274.6 nm	15.568	1179250	49.79	28734

Figure S182. Enantioenriched sample of (2*S*,3*R*,*S*$_a$)-2u: IA column, n-Hex/i-PrOH 95:5, T= 30°C, F= 1.0 mL/min.

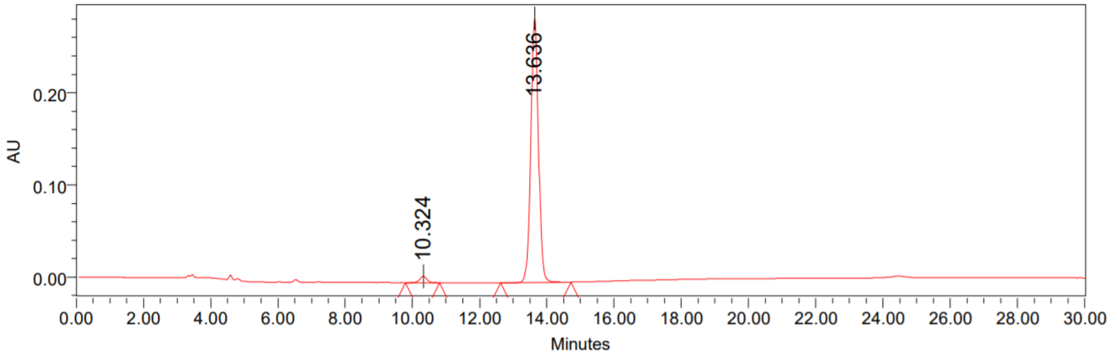

Processed Channel: PDA 243.2 nm

	Processed Channel	Retention Time (min)	Area	% Area	Height
1	PDA 243.2 nm	10.324	98673	2.13	6802
2	PDA 243.2 nm	13.636	4527686	97.87	286820

Figure S183. ^{1}H-NMR (400 MHz, $CDCl_3$) of **(2*S*,3*R*,*S*$_a$)-2v**:

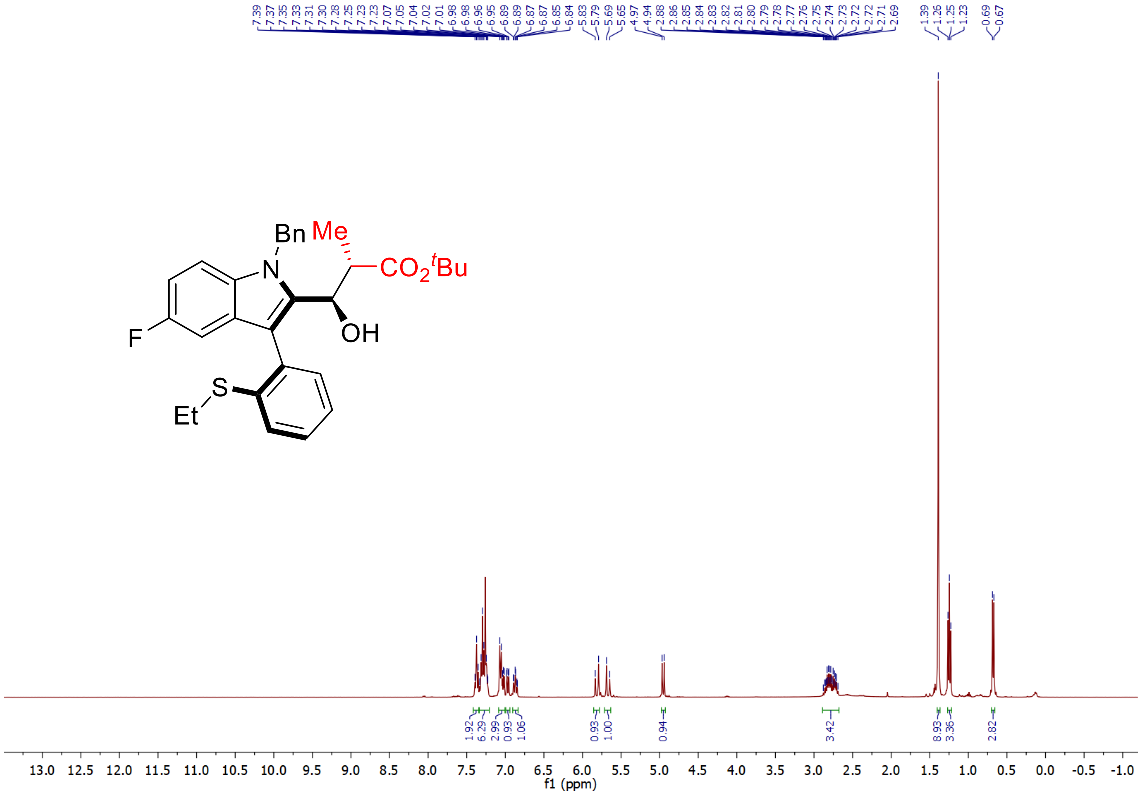

Figure S184. ^{13}C-NMR (100 MHz, $CDCl_3$) of **(2*S*,3*R*,*S*$_a$)-2v**:

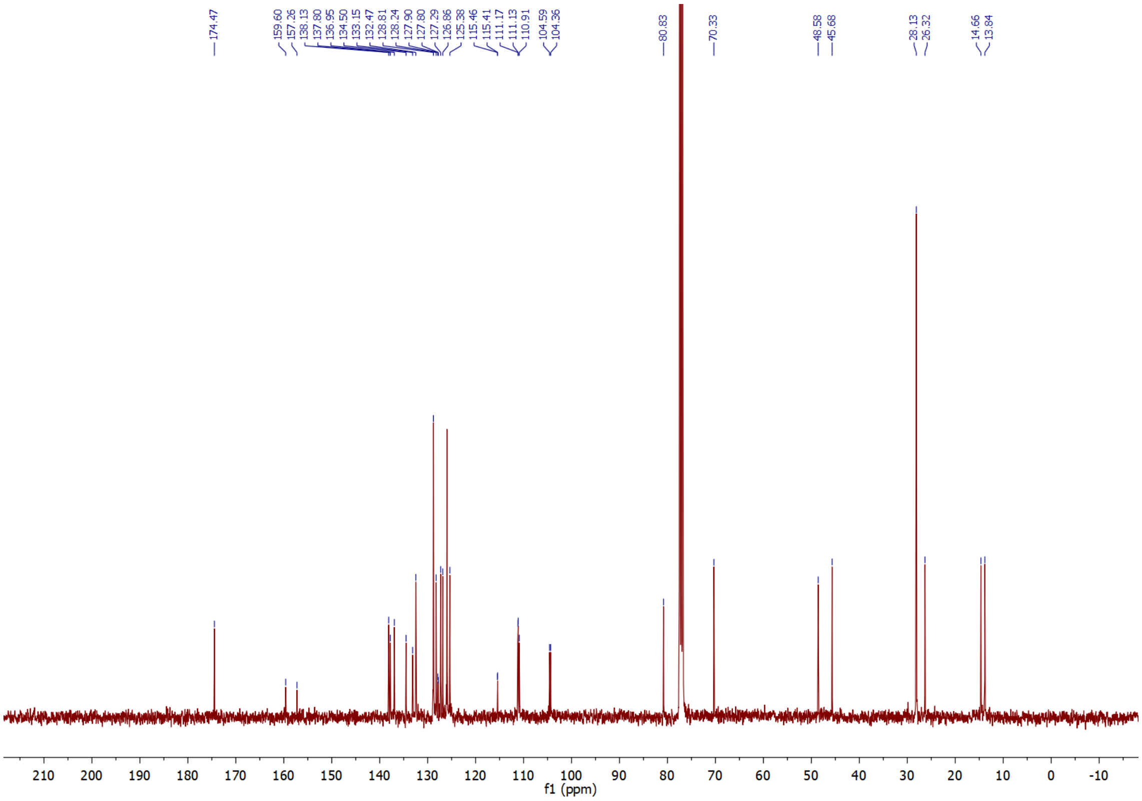

Figure S185. ^{19}F-NMR (376 MHz, $CDCl_3$) of **(2*S*,3*R*,*S**a*)-2v**:

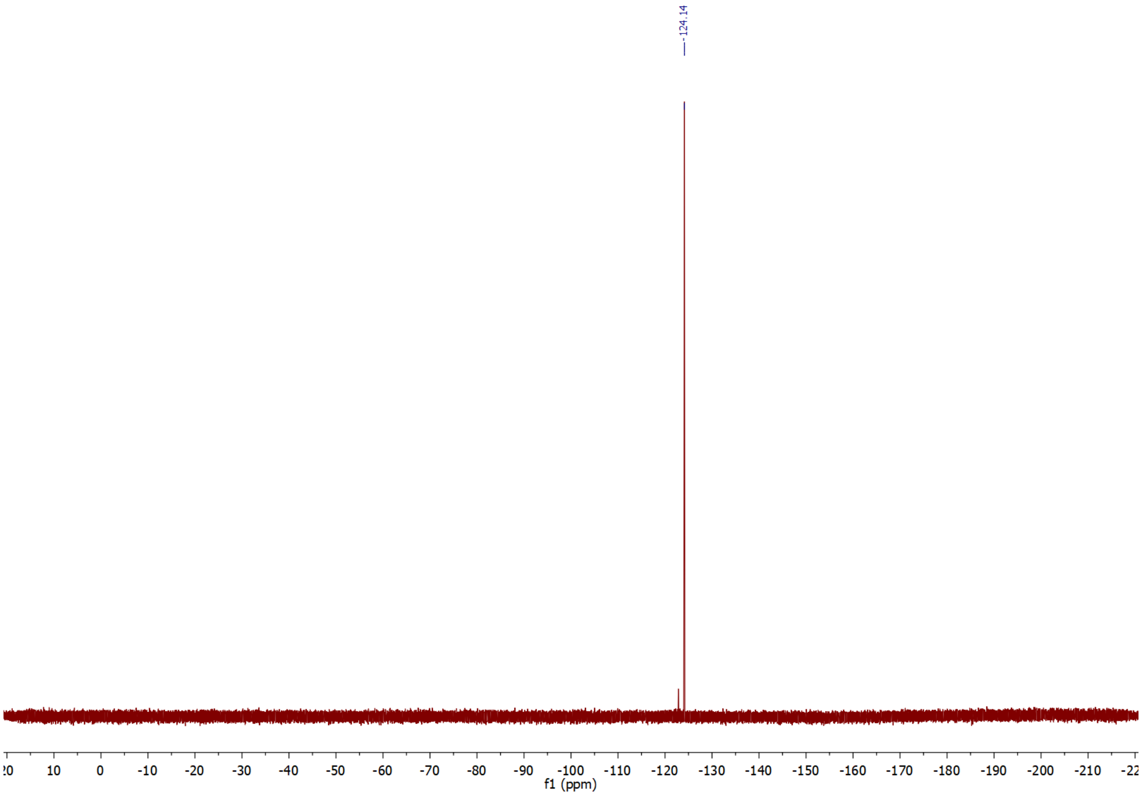

Figure S186. Racemic sample of 2v: IA column, n-Hex/i-PrOH 95:5, T= 30°C, F= 1.0 mL/min.

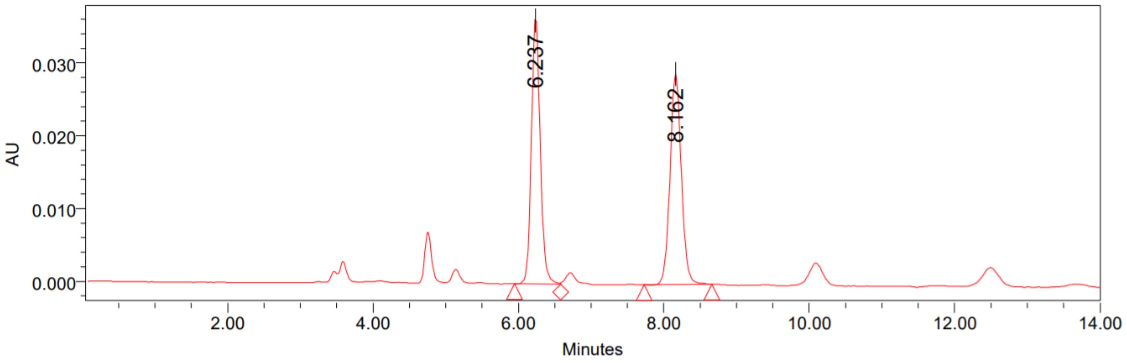

Processed Channel: PDA 268.1 nm

	Processed Channel	Retention Time (min)	Area	% Area	Height
1	PDA 268.1 nm	6.237	308213	49.68	36521
2	PDA 268.1 nm	8.162	312241	50.32	28914

Figure S187. Enantioenriched sample of (2*S*,3*R*,*S*$_a$)-2v: IA column, n-Hex/i-PrOH 95:5, T= 30°C, F= 1.0 mL/min.

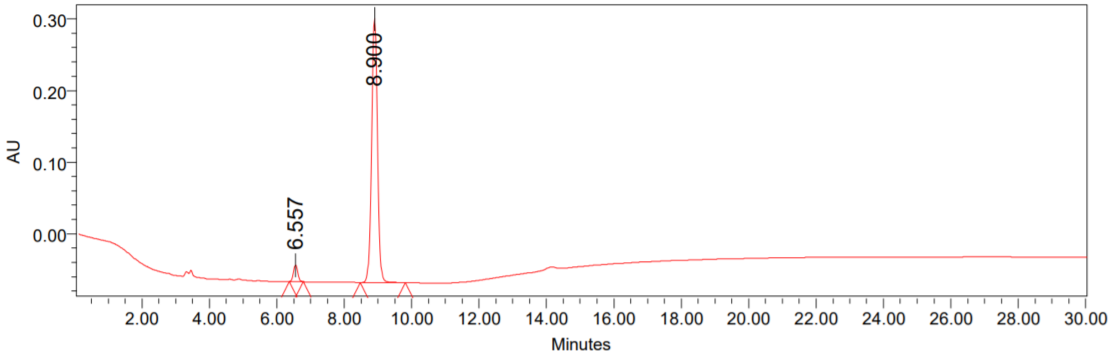

Processed Channel: PDA 221.0 nm

	Processed Channel	Retention Time (min)	Area	% Area	Height
1	PDA 221.0 nm	6.557	206552	4.45	23063
2	PDA 221.0 nm	8.900	4430994	95.55	369282

Figure S188. ^{1}H-NMR (400 MHz, $CDCl_3$) of **(2*S*,3*R*,*S*$_a$)-2x**:

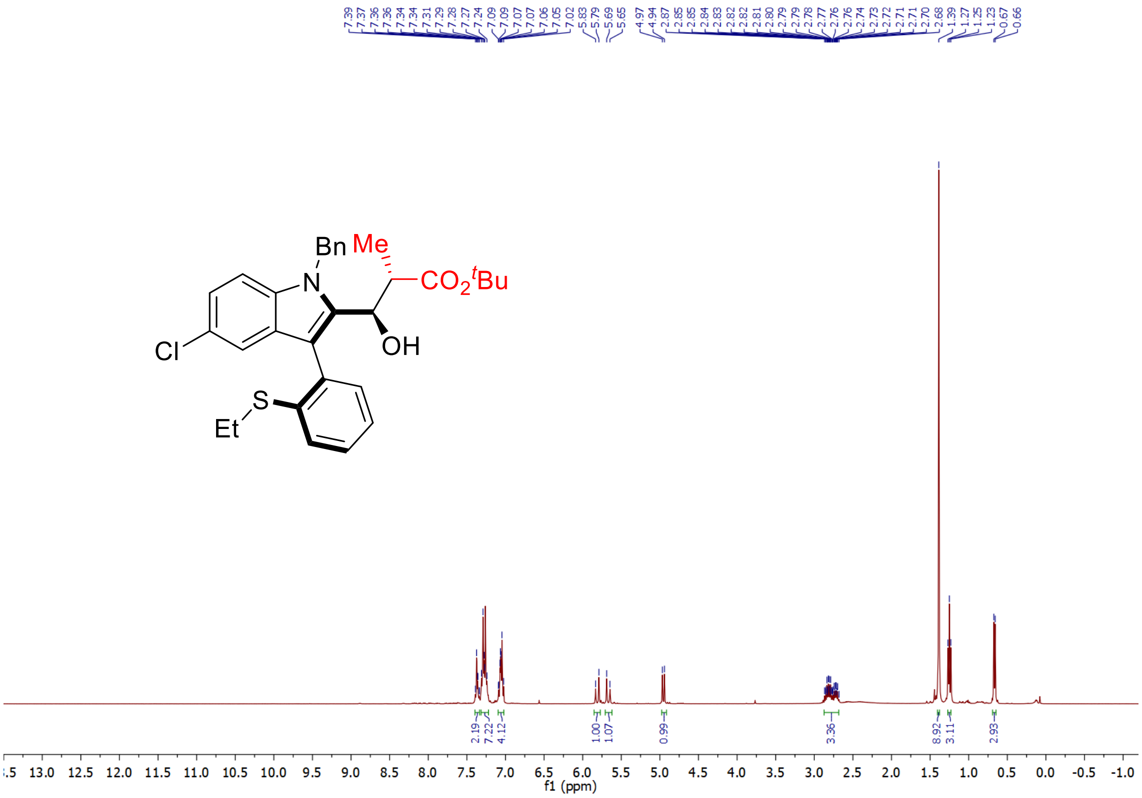

Figure S189. ^{13}C-NMR (100 MHz, $CDCl_3$) of **(2*S*,3*R*,*S*$_a$)-2x**:

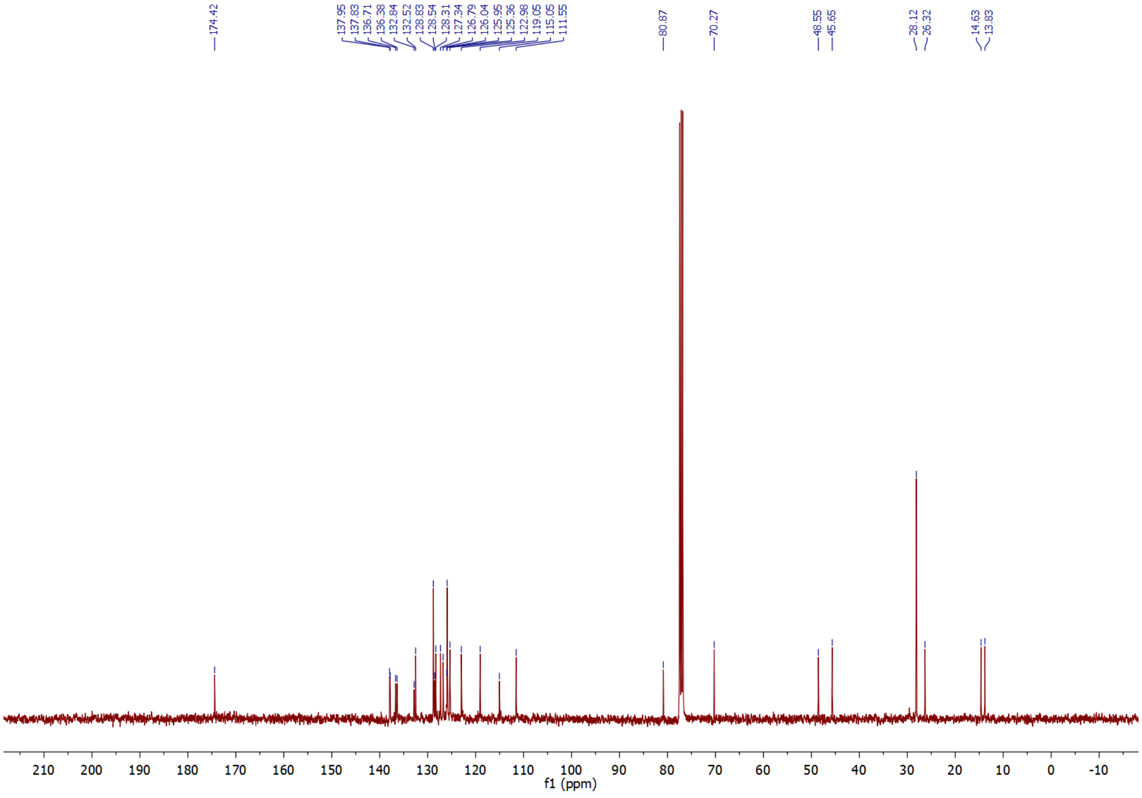

Figure S190. Racemic sample of 2x: IA column, n-Hex/i-PrOH 95:5, T= 30°C, F= 1.0 mL/min.

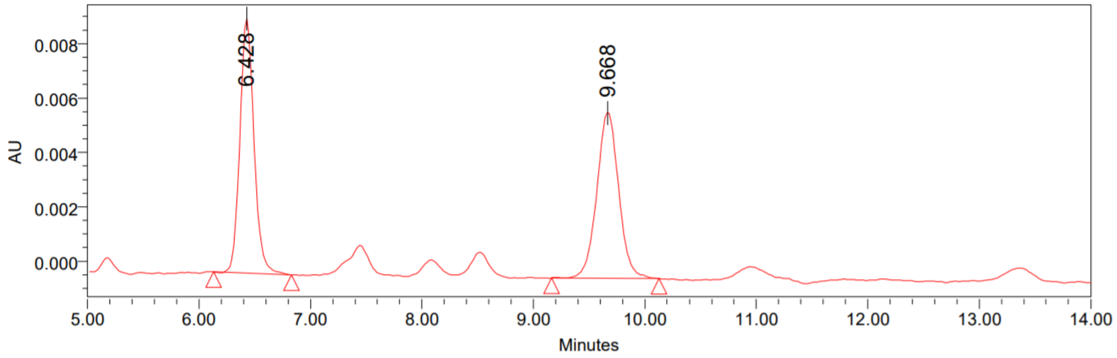

Processed Channel: PDA 285.8 nm

	Processed Channel	Retention Time (min)	Area	% Area	Height
1	PDA 285.8 nm	6.428	82506	49.75	9354
2	PDA 285.8 nm	9.668	83329	50.25	6113

Figure S191. Enantioenriched sample of (2*S*,3*R*,*S*$_a$)-2x: IA column, n-Hex/i-PrOH 95:5, T= 30°C, F= 1.0 mL/min.

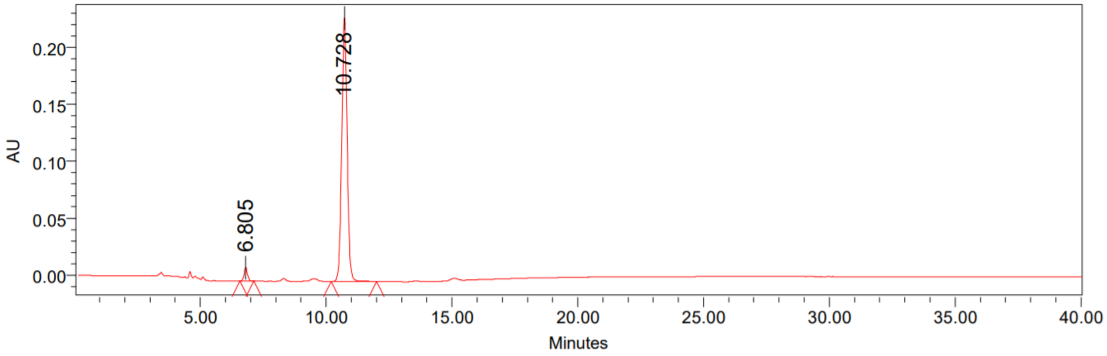

Processed Channel: PDA 244.4 nm

	Processed Channel	Retention Time (min)	Area	% Area	Height
1	PDA 244.4 nm	6.805	113666	3.19	11943
2	PDA 244.4 nm	10.728	3451362	96.81	231515

Figure S192. ^{1}H-NMR (400 MHz, $CDCl_3$) of **(2*S*,3*R*,*S*$_a$)-2y**:

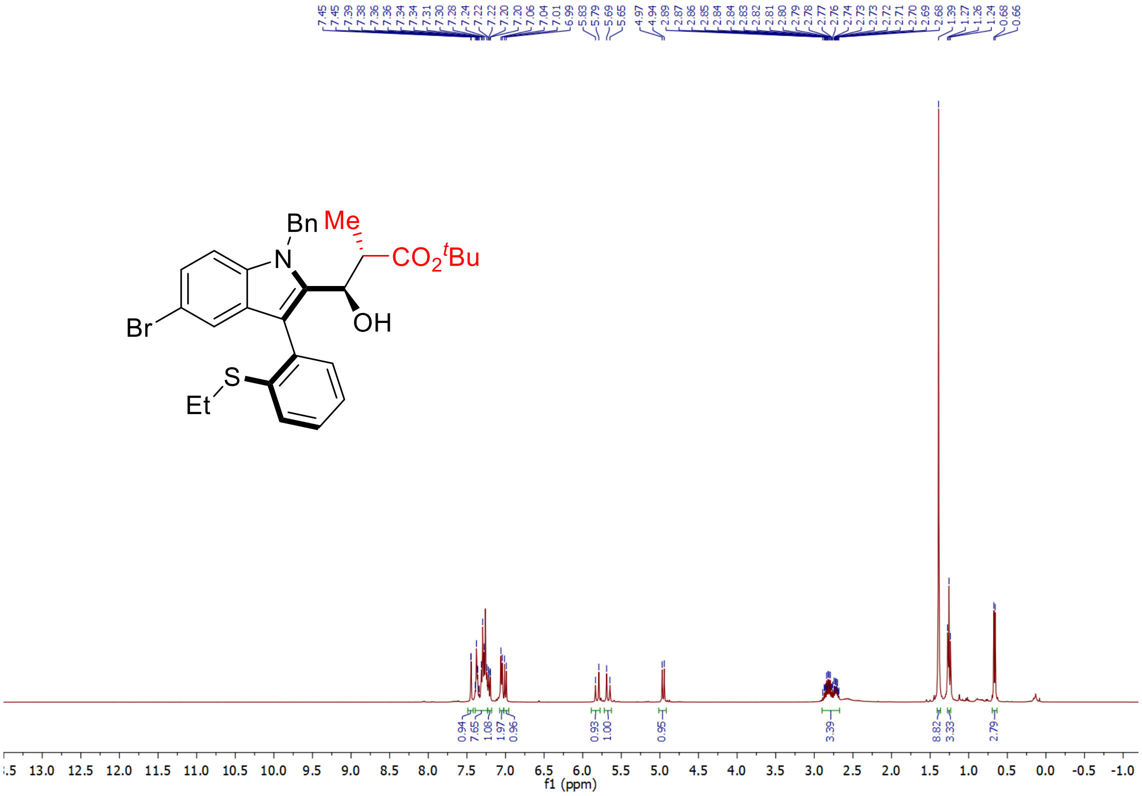

Figure S193. ^{13}C-NMR (100 MHz, $CDCl_3$) of **(2*S*,3*R*,*S*$_a$)-2y**:

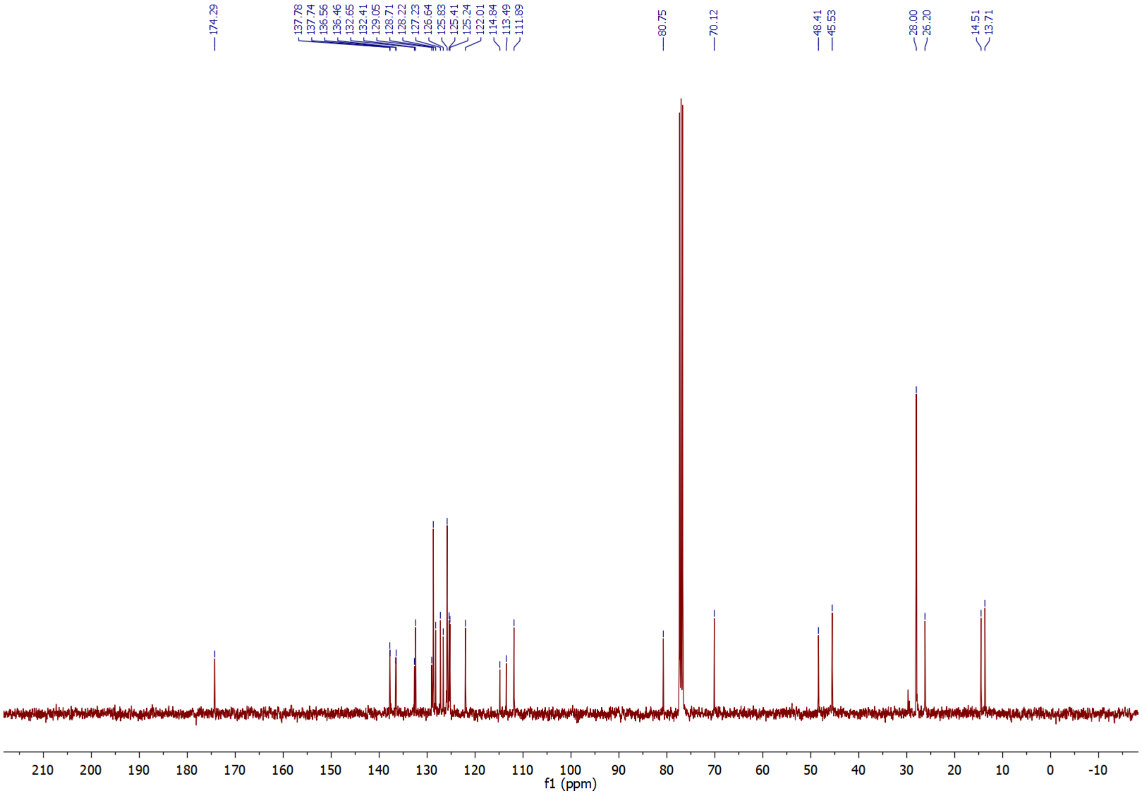

Figure S194. Racemic sample of 2y: IA column, n-Hex/i-PrOH 95:5, T= 30°C, F= 1.0 mL/min.

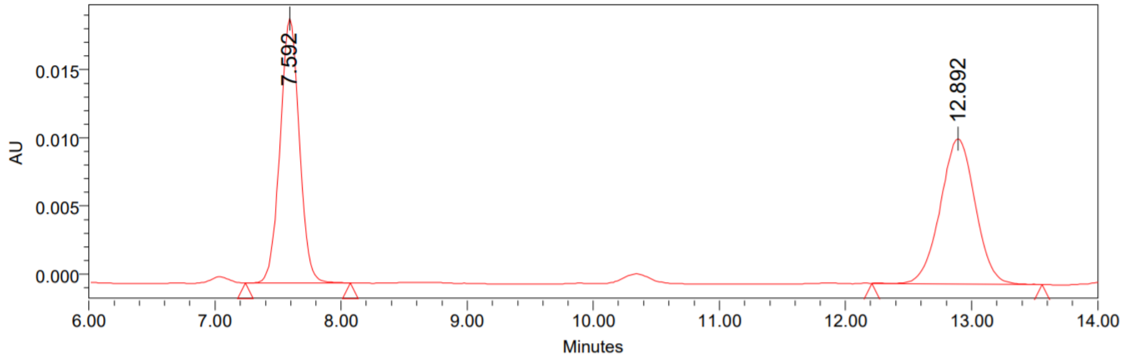

Processed Channel: PDA 267.0 nm

	Processed Channel	Retention Time (min)	Area	% Area	Height
1	PDA 267.0 nm	7.592	206223	50.21	19428
2	PDA 267.0 nm	12.892	204462	49.79	10660

Figure S195. Enantioenriched sample of (2*S*,3*R*,*S*$_a$)-2y: IA column, n-Hex/i-PrOH 95:5, T= 30°C, F= 1.0 mL/min.

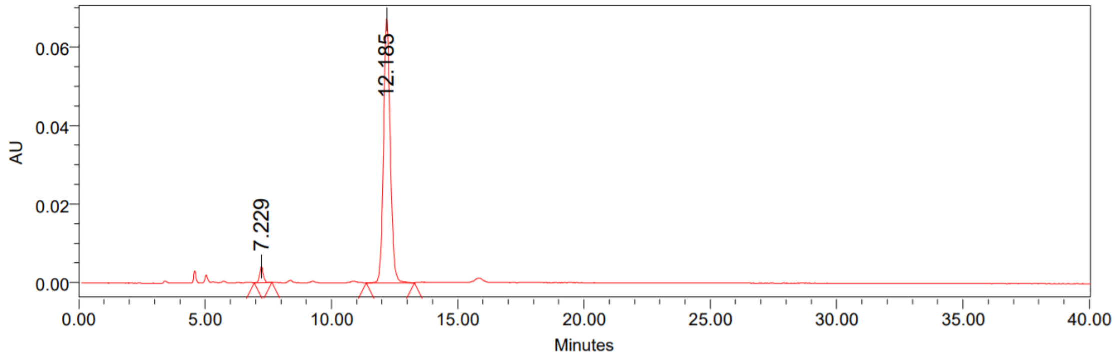

Processed Channel: PDA 312.7 nm

	Processed Channel	Retention Time (min)	Area	% Area	Height
1	PDA 312.7 nm	7.229	40542	3.17	4045
2	PDA 312.7 nm	12.185	1238889	96.83	67170

Figure S196. ^{1}H-NMR (400 MHz, $CDCl_3$) of **(2*S*,3*R*,*S*$_a$)-2z**:

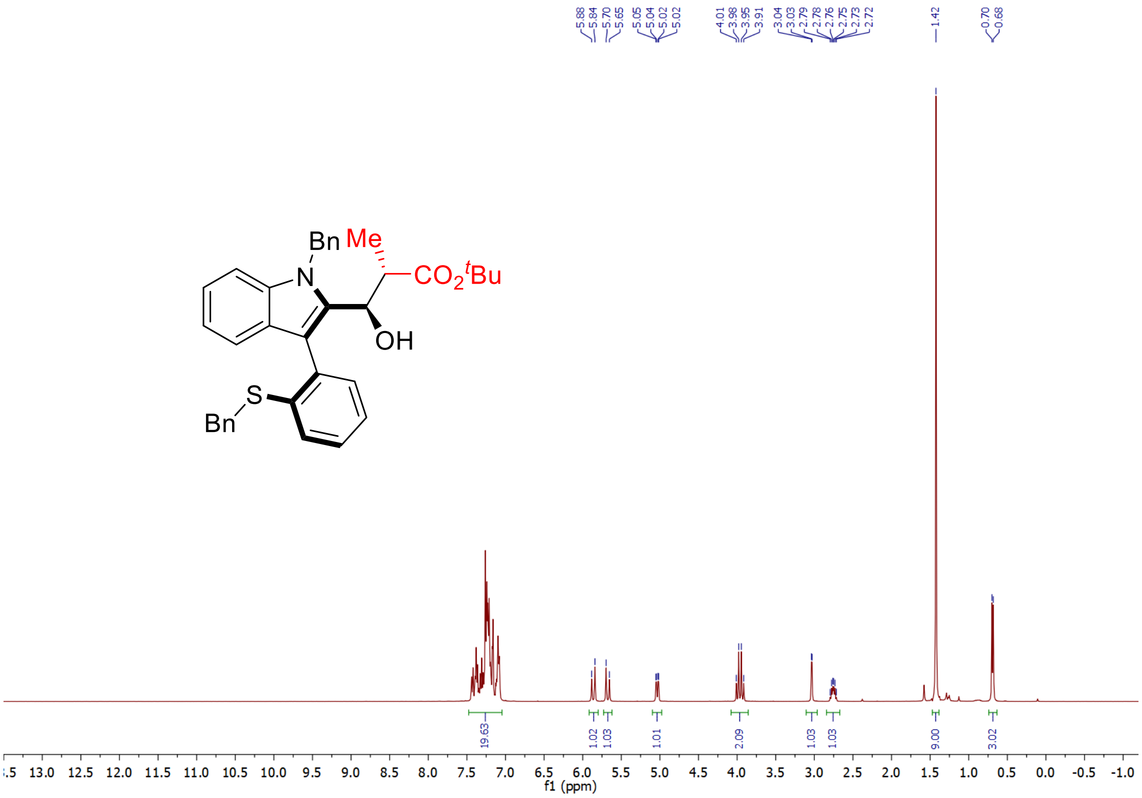

Figure S197. ^{13}C-NMR (100 MHz, $CDCl_3$) of **(2*S*,3*R*,*S*$_a$)-2z**:

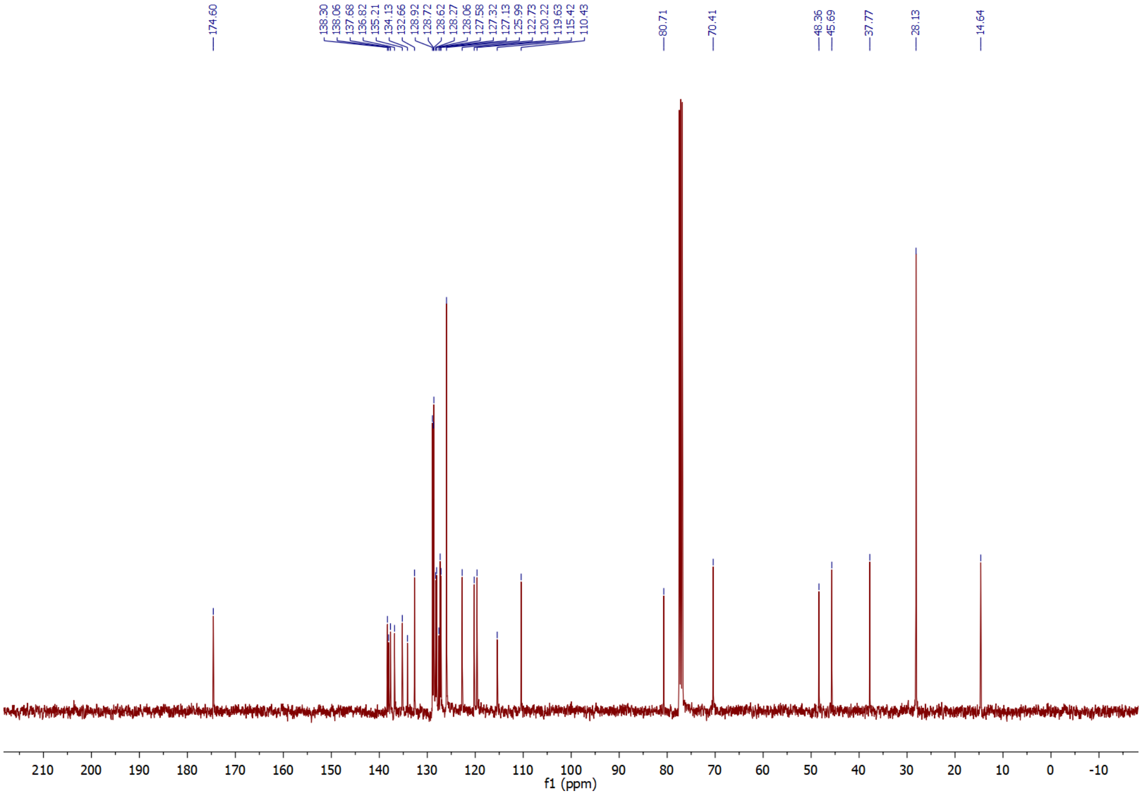

Figure S198. Racemic sample of 2z: IA column, n-Hex/i-PrOH 95:5, T= 30°C, F= 1.0 mL/min.

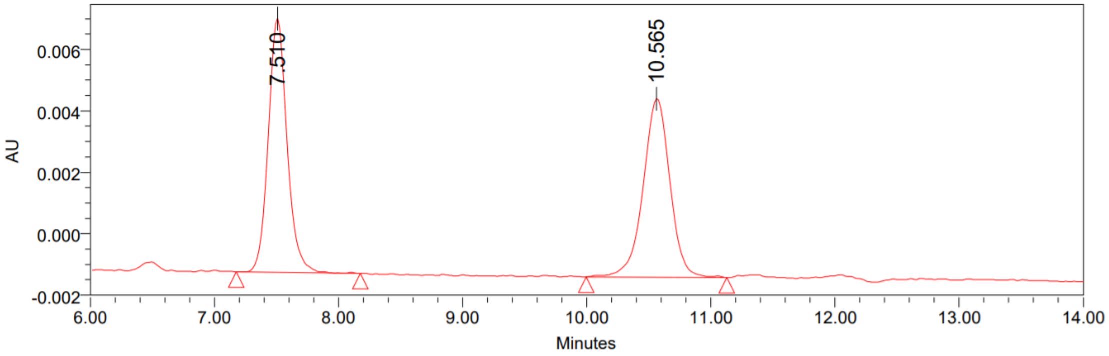

Processed Channel: PDA 282.7 nm

	Processed Channel	Retention Time (min)	Area	% Area	Height
1	PDA 282.7 nm	7.510	87298	49.96	8284
2	PDA 282.7 nm	10.565	87429	50.04	5823

Figure S199. Enantioenriched sample of (2*S*,3*R*,*S*$_a$)-2z: IA column, n-Hex/i-PrOH 95:5, T= 30°C, F= 1.0 mL/min.

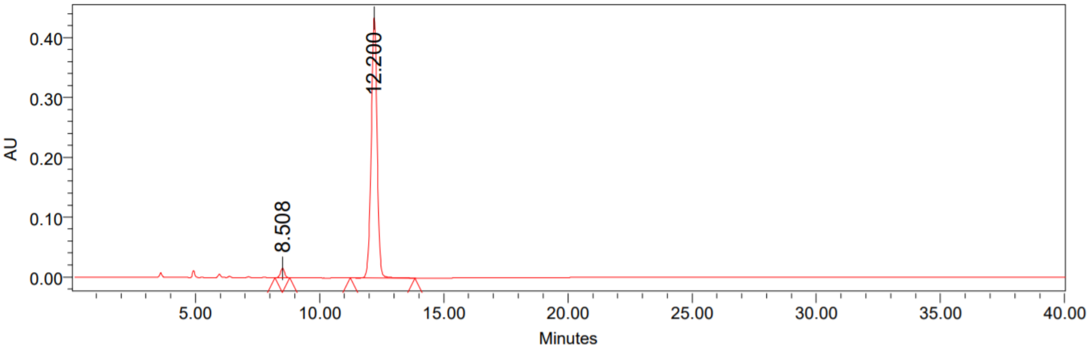

Processed Channel: PDA 256.4 nm

	Processed Channel	Retention Time (min)	Area	% Area	Height
1	PDA 256.4 nm	8.508	188208	2.56	16029
2	PDA 256.4 nm	12.200	7159187	97.44	434952

Figure S200. ^{1}H-NMR (400 MHz, $CDCl_3$) of **(2*S*,3*R*,*S*$_a$)-2aa**:

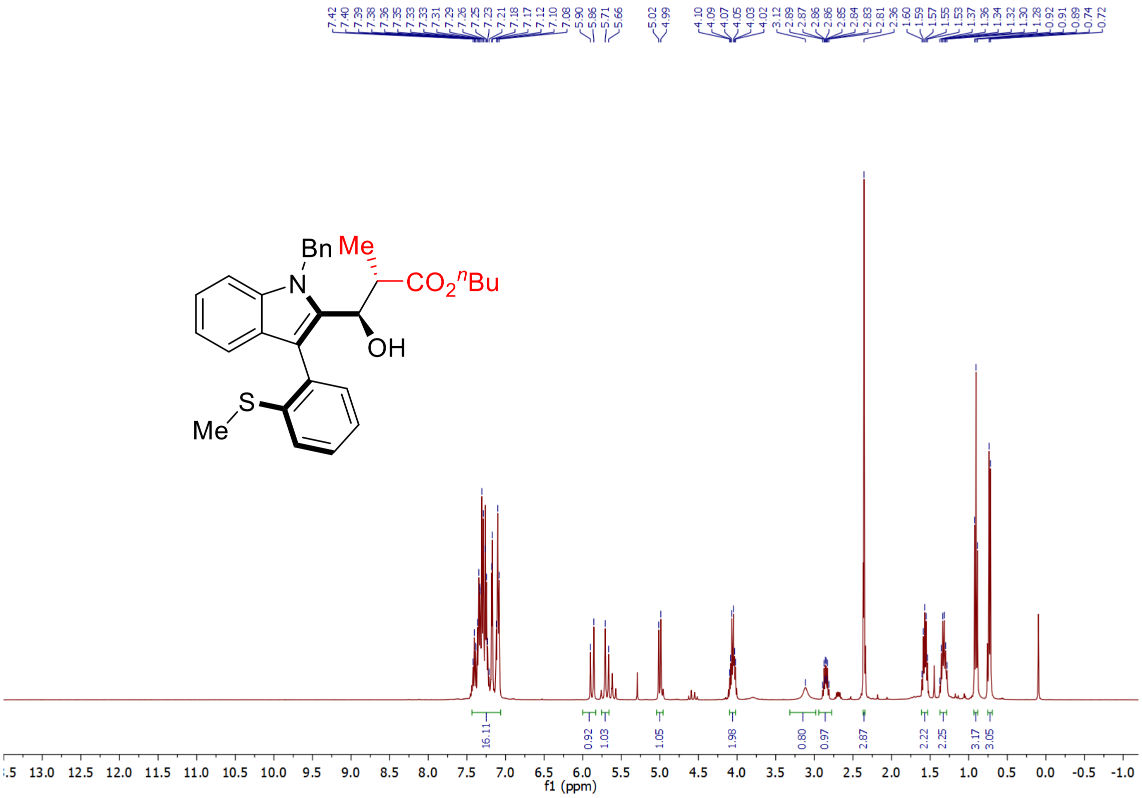

Figure S201. ^{13}C-NMR (100 MHz, $CDCl_3$) of **(2*S*,3*R*,*S*$_a$)-2aa**:

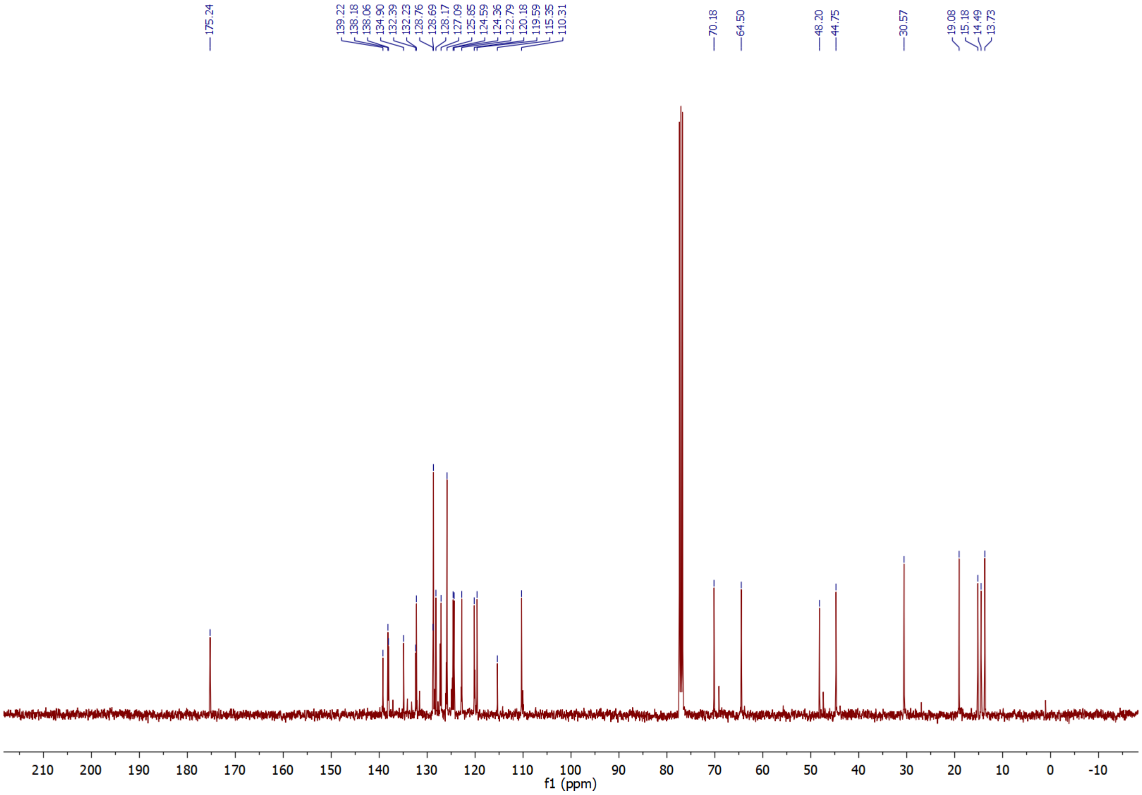

Figure S202. Enantioenriched sample of (2*S*,3*R*,*S*$_a$)-2aa: IA column, n-Hex/i-PrOH 98:2, T= 30°C, F= 1.0 mL/min.

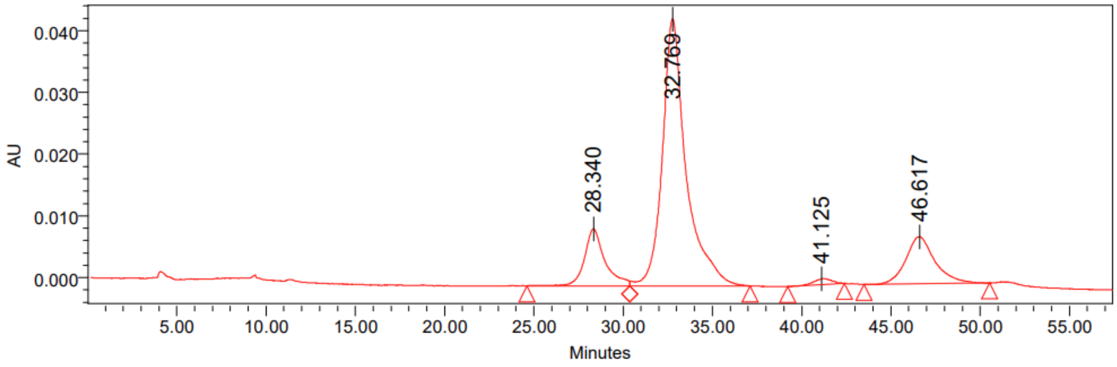

Processed Channel: PDA 272.4 nm

	Processed Channel	Retention Time (min)	Area	% Area	Height
1	PDA 272.4 nm	28.340	732061	12.91	9211
2	PDA 272.4 nm	32.769	4002187	70.56	43246
3	PDA 272.4 nm	41.125	69841	1.23	962
4	PDA 272.4 nm	46.617	867705	15.30	7628

Figure S203. ^{1}H-NMR (400 MHz, $CDCl_3$) of **(2*S*,3*R*,*S*$_a$)-2ab/(2*S*,3*R*,*R*$_a$)-2ab 1.7:1**:

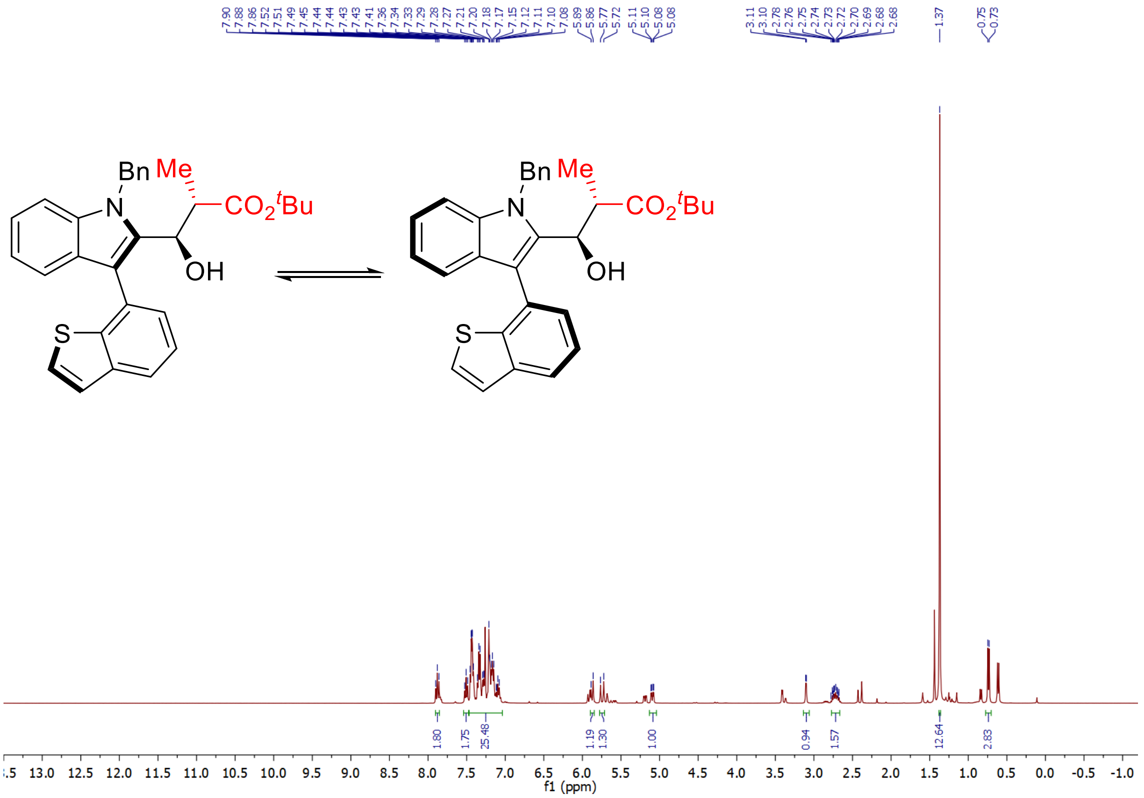

Figure S204. ^{13}C-NMR (100 MHz, $CDCl_3$) of **(2*S*,3*R*,*S*$_a$)-2ab/(2*S*,3*R*,*R*$_a$)-2ab 1.7:1**:

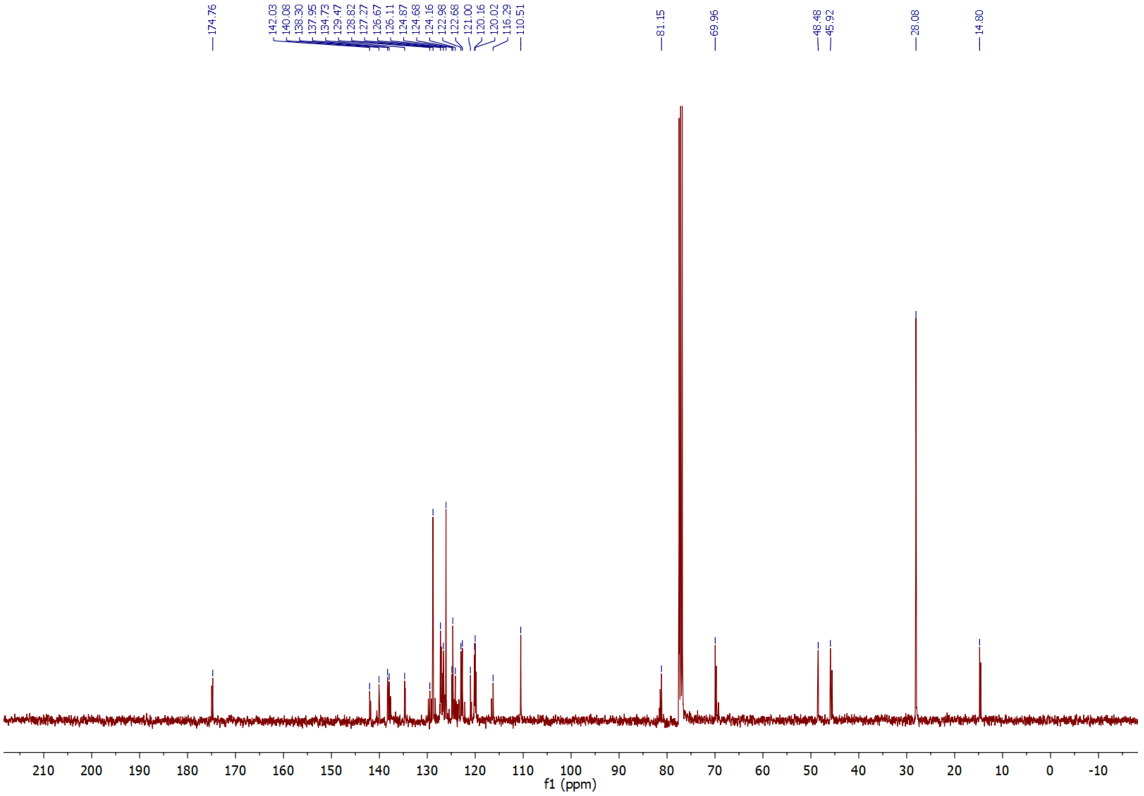

Figure S205. ^{1}H-NMR (400 MHz, $CDCl_3$) of **(2*S*,3*R*,*S*$_a$)-2ab/(2*S*,3*R*,*R*$_a$)-2ab 1.2:1**:

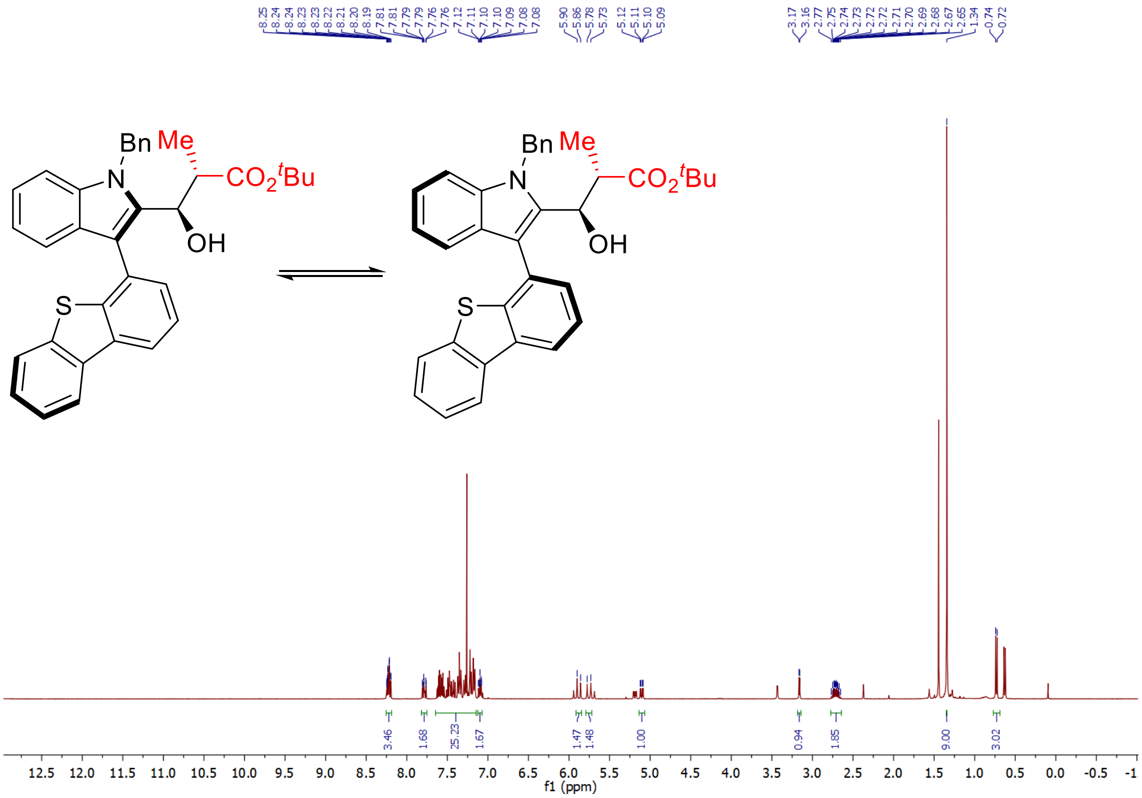

Figure S206. ^{13}C-NMR (100 MHz, $CDCl_3$) of **(2*S*,3*R*,*S*$_a$)-2ab/(2*S*,3*R*,*R*$_a$)-2ab 1.2:1**:

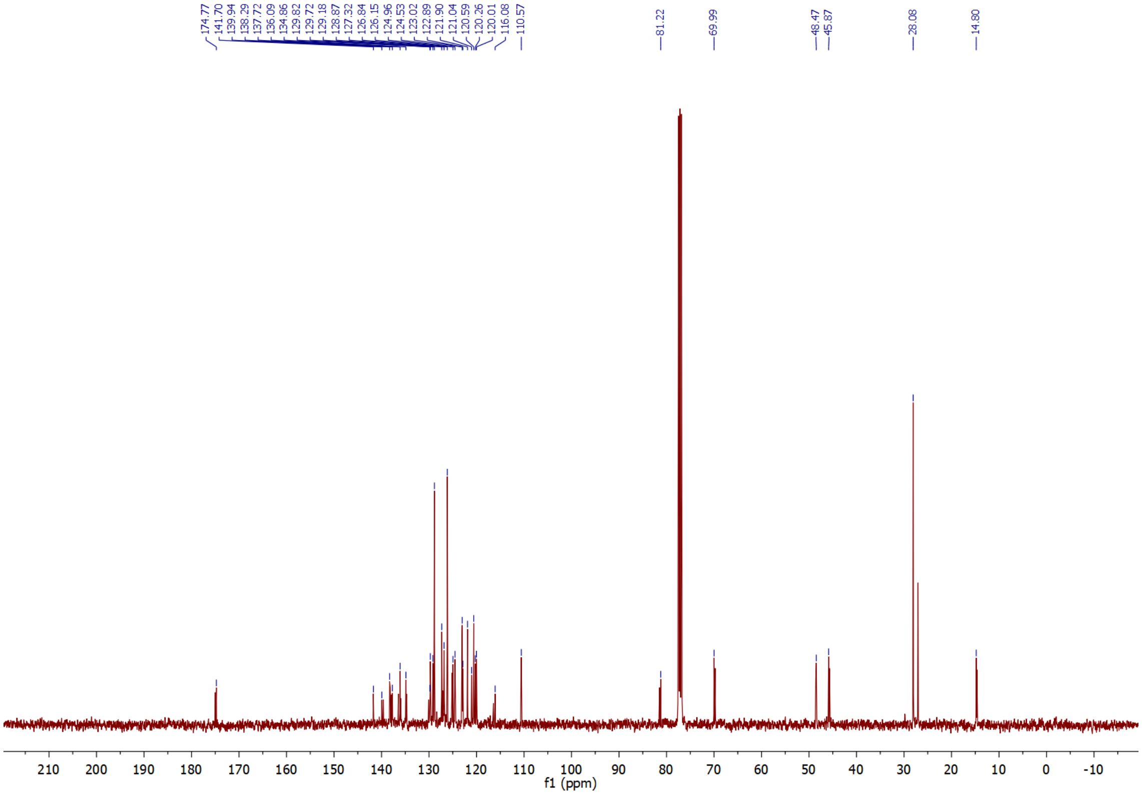

Figure S207. ^{1}H-NMR (400 MHz, CDCl$_3$) of **(1*R*,2*R*,*S$_a$*)-3**:

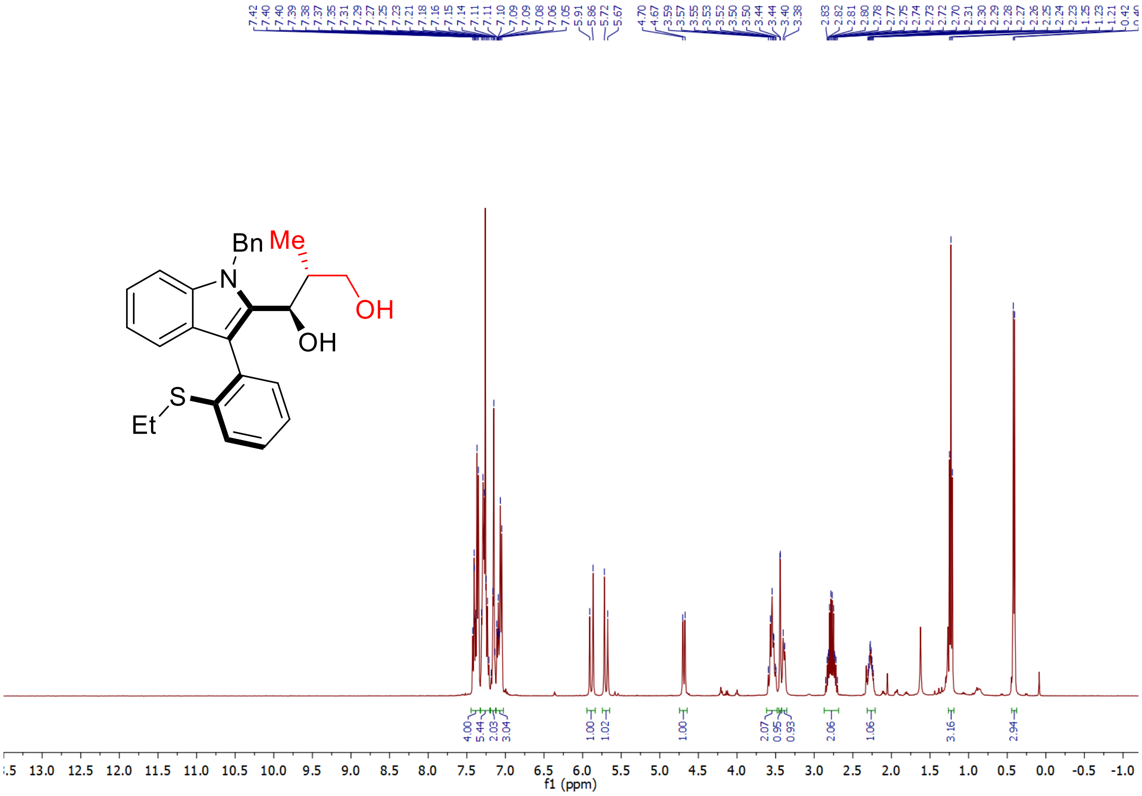

Figure S208. ^{13}C-NMR (100 MHz, CDCl$_3$) of **(1*R*,2*R*,*S$_a$*)-3**:

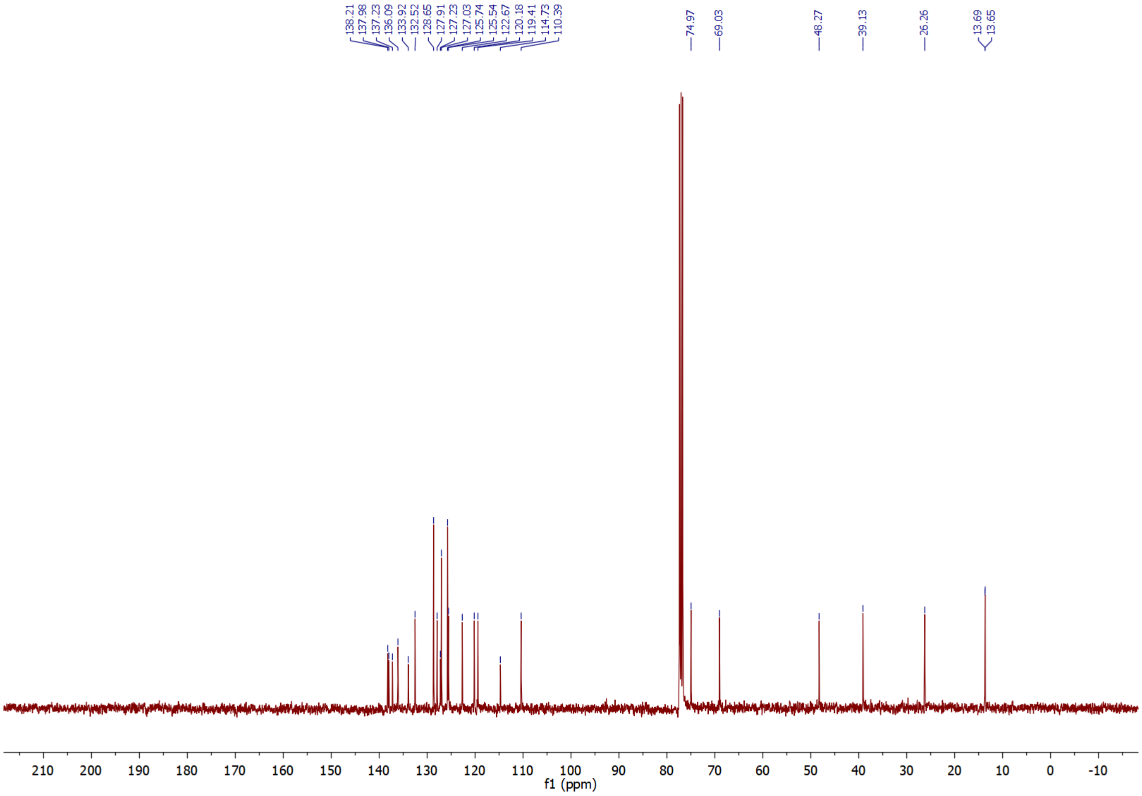

Figure S209. ^{1}H-NMR (400 MHz, CDCl$_3$) of **(4*R*,5*R*,*S*$_a$)-4**:

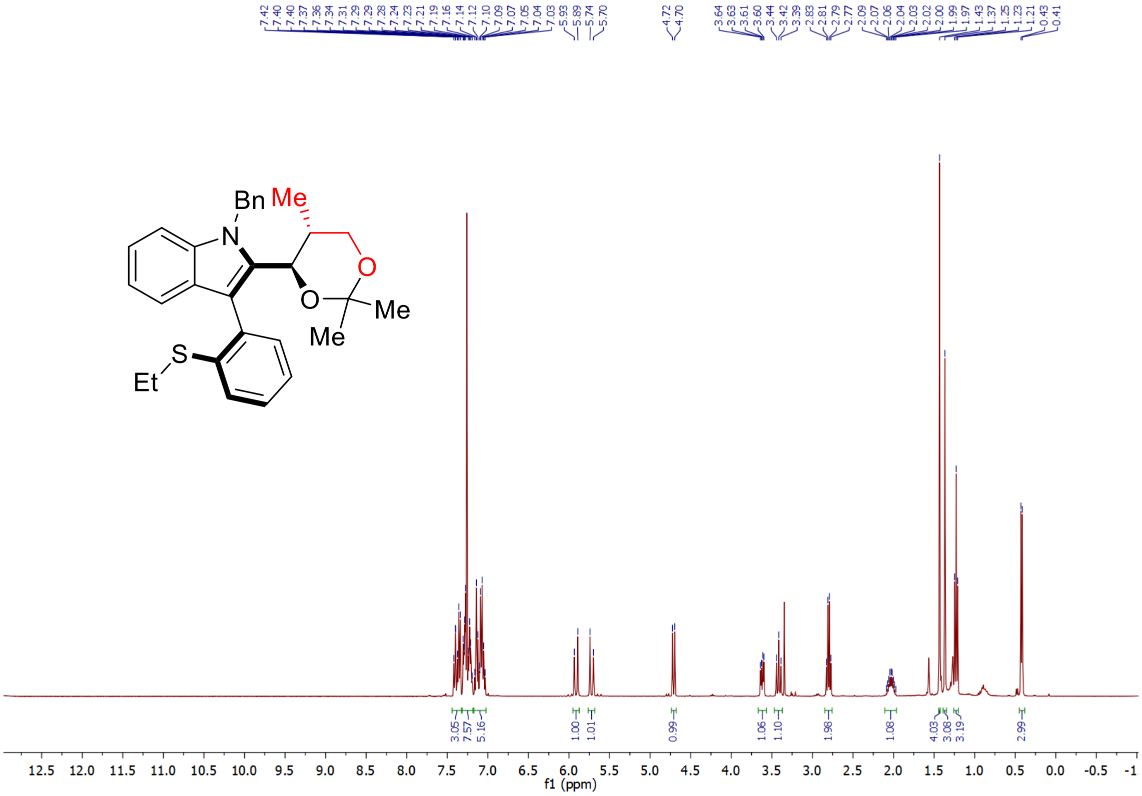

Figure S210. ^{13}C-NMR (100 MHz, CDCl$_3$) of **(4*R*,5*R*,*S*$_a$)-4**:

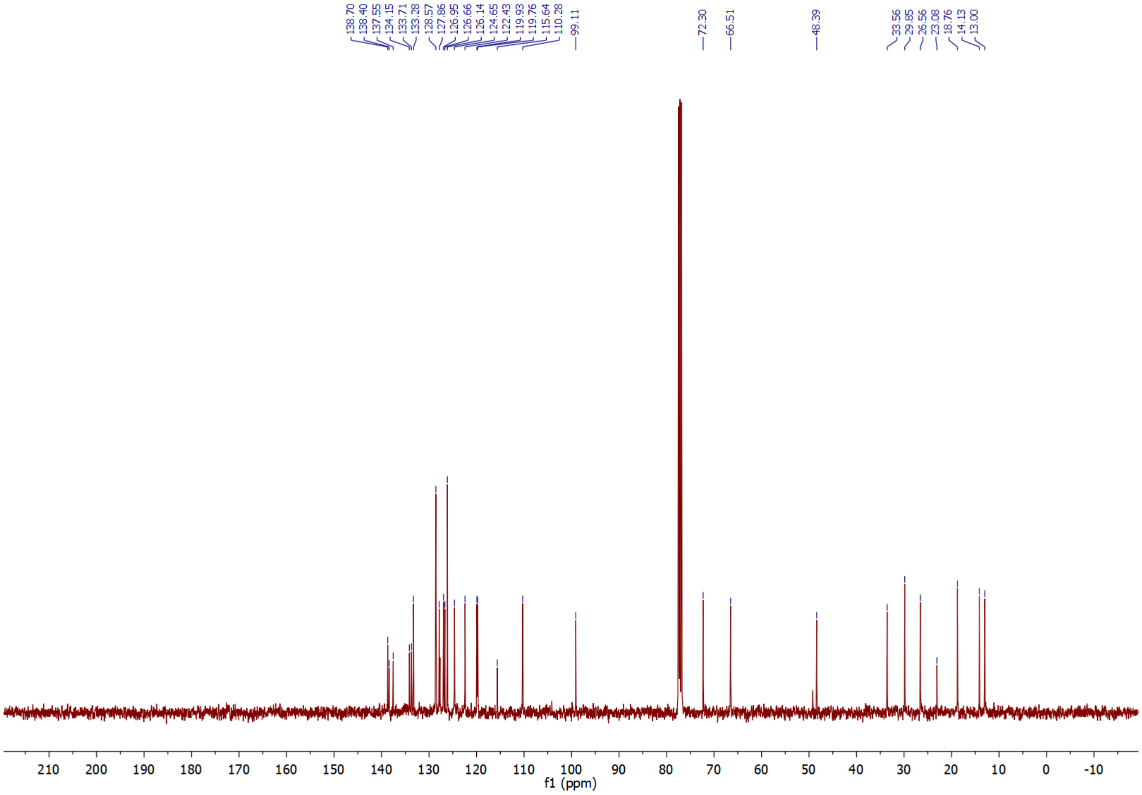

Figure S211. ^{1}H-NMR (400 MHz, $CDCl_3$) of **(2*S*,3*R*,*S*$_a$)-5**:

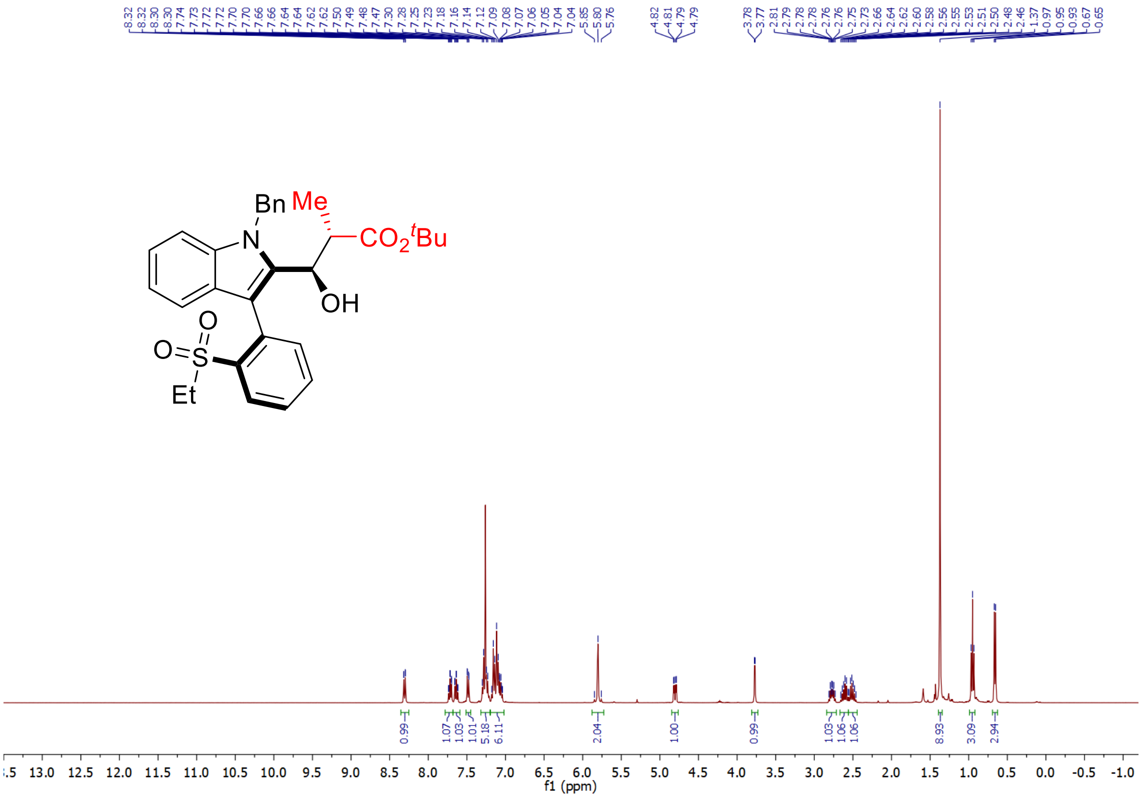

Figure S212. ^{13}C-NMR (100 MHz, $CDCl_3$) of **(2*S*,3*R*,*S*$_a$)-5**:

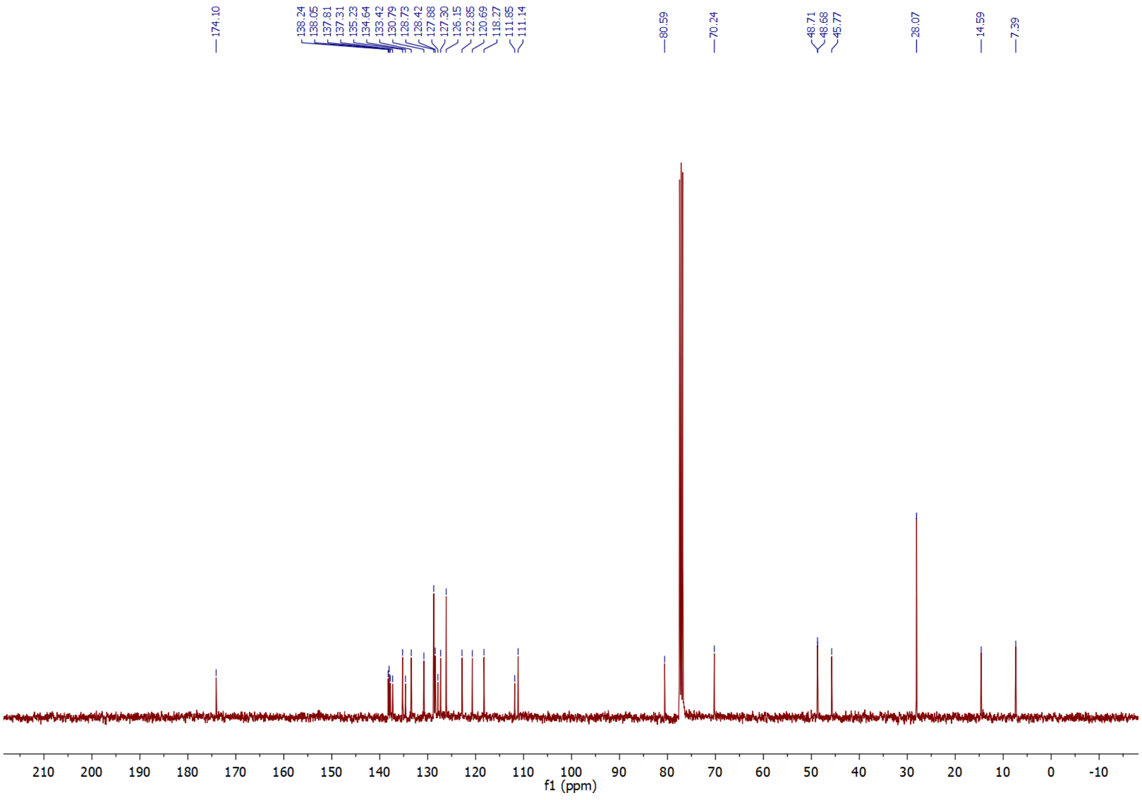

Figure S213. ^{1}H-NMR (400 MHz, $CDCl_3$) of **(2*S*,3*R*,*S*$_a$,*R*$_S$)-6**:

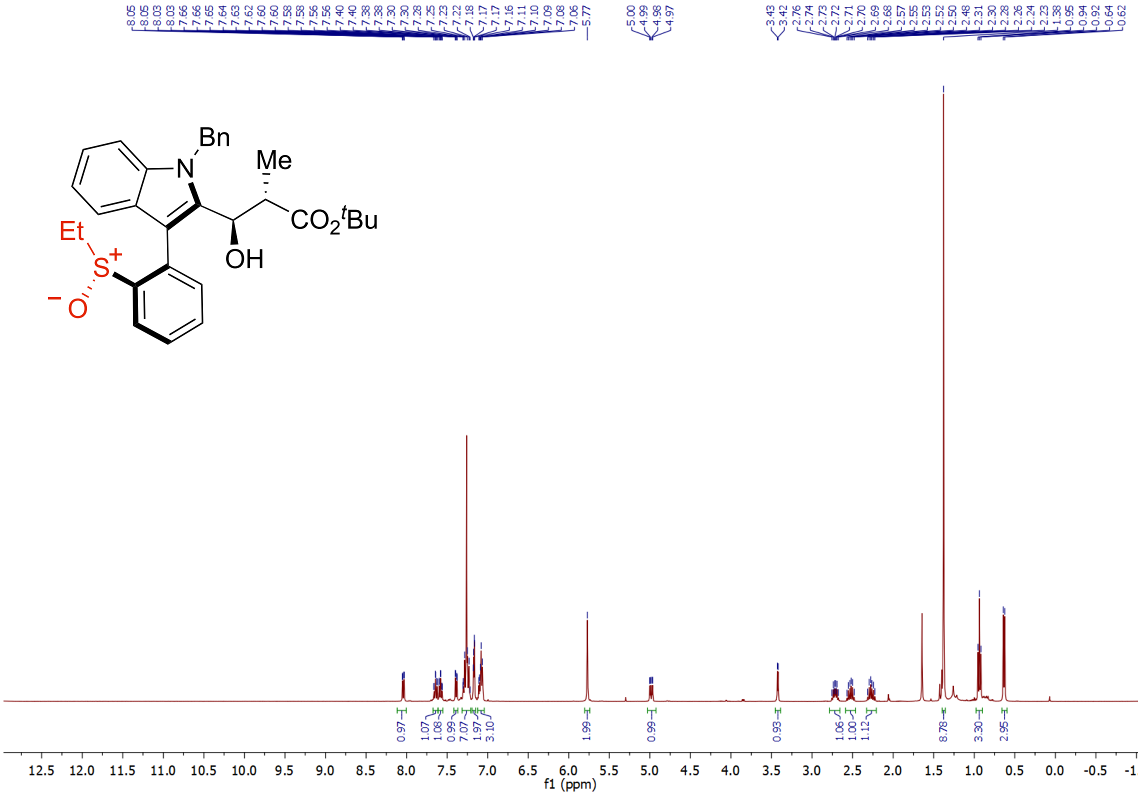

Figure S214. ^{13}C-NMR (100 MHz, $CDCl_3$) of **(2*S*,3*R*,*S*$_a$,*R*$_S$)-6**:

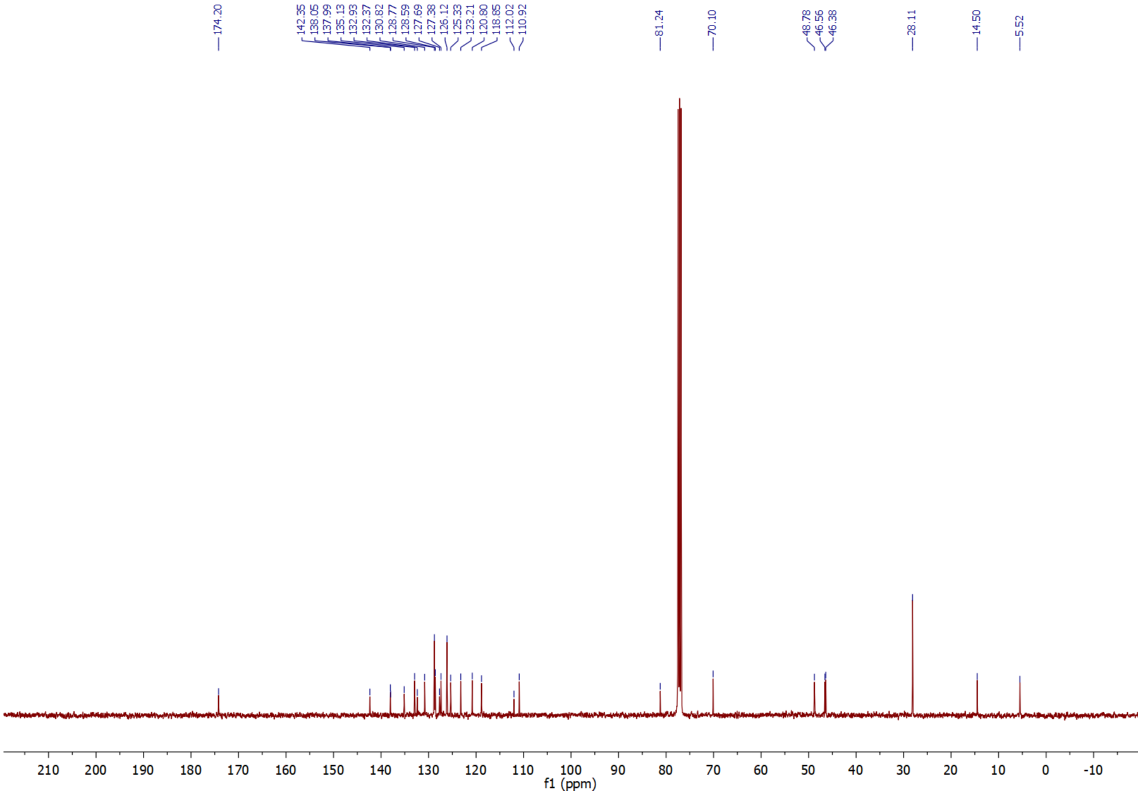

Supplement: Supplementary file 1 — cs3c03422_si_001.pdf [file cs3c03422_si_001.pdf]
